# Structurally Diverse Nitrogen-Rich Scaffolds via Continuous Photo-Click Reactions

**DOI:** 10.1021/acs.orglett.4c03953

**Published:** 2024-11-26

**Authors:** Davin Cronly, Megan Smyth, Thomas S. Moody, Scott Wharry, Julia Bruno-Colmenarez, Brendan Twamley, Marcus Baumann

**Affiliations:** † School of Chemistry, University College Dublin, O’Brien Centre for Science, Belfield, Dublin 4, Ireland; ‡ Technology Department, Almac Sciences, Craigavon, BT63 5QD, U.K.; § Arran Chemical Company, Monksland Industrial Estate, Roscommon N37 DN24, Ireland; ∥ School of Chemistry, Trinity College Dublin, Dublin 2, Ireland

## Abstract

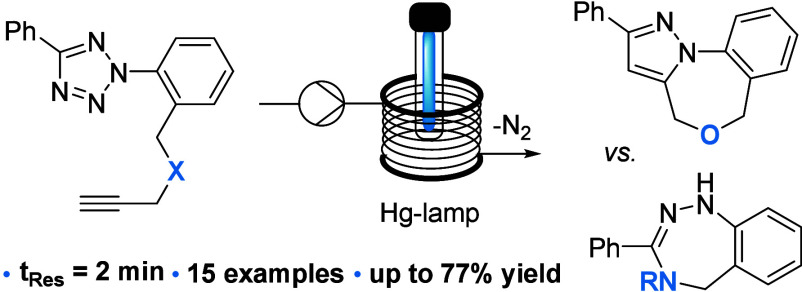

Continuous flow technology was exploited for the effective generation of nitrile imines via photolysis of substituted aryl tetrazoles. The resulting photo-click process rapidly affords advanced nitrogen-rich scaffolds upon the subsequent trapping of the reactive dipole with alkenes, alkynes, and benzylic amines. Crucially, this approach uncovers the differential reactivity for ether vs amine tethers, thus providing facile and scalable access to underexplored medicinally relevant heterocyclic entities.

Since their inception by Huisgen in the 1950s and the subsequent popularization by Sharpless and others, click reactions have become a main staple in the synthetic chemist’s toolbox.^1^ Click reactions benefit from operational simplicity, high atom and step economy, excellent reproducibility and a vast structural diversity of the resulting products making them popular tools for bioconjugations, polymer applications and materials chemistry.^2^ Although most applications are based on thermal or metal-catalyzed click reactions, a subset of studies exploit light to trigger these processes. Representative examples of photo-click reactions include thiol–ene reactions, photoinitiated Diels–Alder reactions as well as tetrazole–ene reactions.^3^ Photo-click reactions render further advantages as the use of specific wavelengths of light provide better selectivity in the absence of stoichiometric additives that normally require separation after the reaction. In parallel, photochemistry has witnessed a renaissance in the last 15 years with many new and mild reactions being discovered in academic and industrial laboratories.^4^ A further important development in this field is the use of miniaturized continuous flow reactors that increase reaction efficiency due to short path lengths of light and uniform irradiation of the reaction mixture that is pumped through narrow-dimension tubing.^5^ High spatiotemporal control thereby minimizes side reactions that otherwise result from overirradiation, and reaction automation as well as simple scale-up by continuous processing make photochemical flow reactions a very powerful tool for the effective generation of advanced building blocks.^6^ Nonetheless, the field of flow-based photo-click reactions remains underdeveloped despite the possibility of safely generating reactive intermediates *in situ* using light. Specifically, the continuous generation of nitrile imines from tetrazoles^7^ which is a powerful strategy to access a variety of azacyclic targets has only been reported by Jamieson en route to oxadiazoles^8^ prior to our own reports toward a variety of bioactive pyrazolines^9^ (Scheme 1). Based on our group’s interest in exploiting flow reactor technology as a tool for the discovery of new chemical reactivities,^10^ we wished to investigate the use of nitrile imines toward complex drug-like scaffolds.

**Scheme 1:**
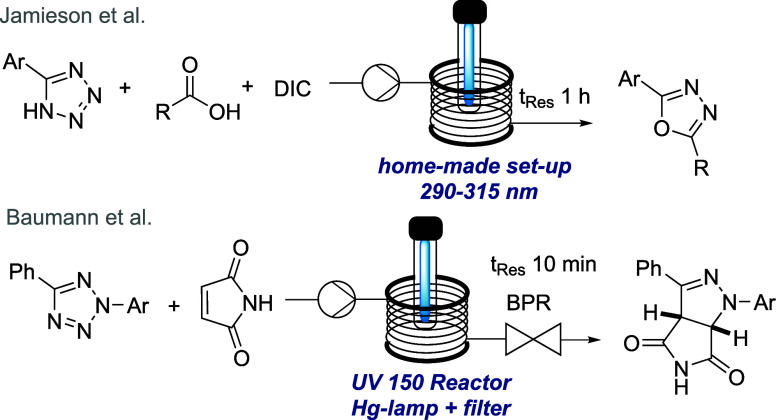
Tetrazoles in Continuous Photo-Click Reactions

Our study commenced with the preparation of the aryl tetrazole core that exploited the union of aryl diazonium salts with phenyl amidine species in the presence of iodine as an oxidant^11^ (see SI for details). Although tetrazole species may present safety concerns due to thermal instability, DSC measurements indicated that tetrazole **1** is stable at ambient temperatures (see ESI for details). As shown in Scheme 2, the first functionalization step required the bromination of the benzylic position which we envisaged could be achieved via a chromoselective photobromination using *N*-bromosuccinimide (NBS) in flow mode. Although photobromination reactions have previously been reported in flow mode,^12^ the presence of the photolabile tetrazole core warranted careful choice of light source and residence time to avoid side reactions. Passing solutions of the tetrazole substrate **1** (MeCN, 0.1 M, containing 1.5 equiv. NBS) through the UV-150 photoreactor of a Vapourtec E-Series flow reactor afforded the desired monobromination products in a residence time of 20 min. Crucially, the use of a high-power UV-A LED emitting at 365 nm (100 W input power) generated the desired products **2a**–**d** selectively without affecting the tetrazole core. In addition, the formation of geminal dibromide products was not observed, likely because of the high spatiotemporal control offered via this flow approach. However, when using an excess of the NBS reagent monobromination of both methyl groups in compound **1b** can be achieved to furnish product **2c**. Subsequent substitution of the bromide with alcohols and amines bearing alkene and alkyne moieties completed the substrate synthesis (see ESI for details).

**Scheme 2:**
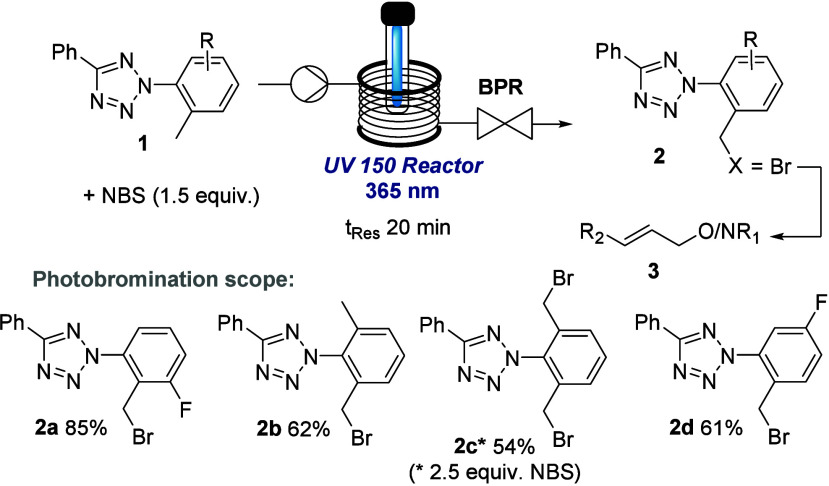
Access to Aryl Tetrazole Building Blocks **2** and **3**

Using tetrazole **3a** we next studied the photochemical reaction toward the tricyclic pyrazole **4a** whereby the initial formation of the nitrile imine dipole is followed by an intramolecular dipolar cycloaddition. An adjustable medium-pressure Hg-lamp (set to 137 W input power) with a low-pass filter was used in combination with the aforementioned Vapourtec photoflow reactor module. As indicated in Table 1, this study highlighted that the desired photoproduct can be obtained in high yields and short residence times using different solvents with toluene, xylene and ethyl acetate giving the best results (entries 1–5). For the latter solvent, the effect of higher concentrations (up to 100 mM) was evaluated showing that the initial drop in conversion can be compensated by extending the residence time from 2 to 5 min (entries 6–8) which is attractive for achieving increased productivity for the desired product. Moreover, under these conditions varying amounts of unreacted starting material were recovered that can be recycled.

**Table 1:**
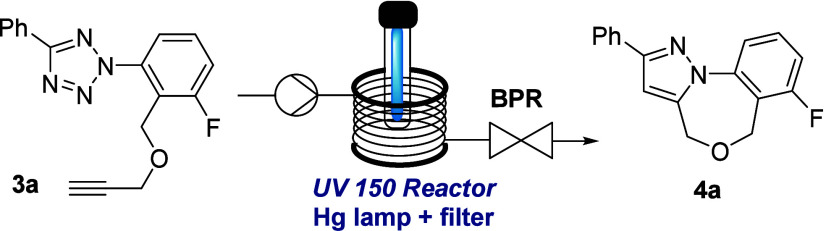
Reaction Optimization for Tricyclic Pyrazole **4a**

entry	solvent	*t*_Res_	**4a**	**3a**
**1**	MeCN (10 mM)	2 min	43%	50%
**2**	acetone (10 mM)	2 min	54%	18%
**3**	toluene (10 mM)	2 min	77%	20%
**4**	xylene (10 mM)	2 min	76%	13%
**5**	EtOAc (10 mM)	2 min	71%	20%
**6**	EtOAc (50 mM)	2 min	46%	44%
**7**	EtOAc (100 mM)	2 min	37%	57%
**8**	EtOAc (100 mM)	5 min	59%	32%

Next, we decided to evaluate the scope of this intramolecular photo-click process. Using the standard conditions (entry 5) showed that the desired tricyclic photoproducts are obtained in all cases (Figure 1). Products based on the aromatic pyrazole substructure were typically generated in the highest yields (i.e., **4a** etc.) unless a disubstituted alkyne is employed in which case significant decomposition was observed (**4d**). Moreover, related pyrazolines were accessed when using alkenyl ethers instead of propargyl ethers. These racemic scaffolds represent stable and often crystalline products that are easily separated from the remaining starting material. The embedded benzoxazine (**4a**–**d**,**f**,**h**) and benzoxazocine (**4e**) moieties add further value to these novel scaffolds in view of future medicinal chemistry applications due to their nonplanar conformation which, along with the hydrogen-bond acceptors, may impart increased solubility. In fact, simpler benzoxazine systems are found in drugs such as loxapine^13^ and nefopam^14^ which are used in the treatment of neurological disorders and analgesia, indicating the potential value of these new scaffolds in related contexts.

**Figure 1:**
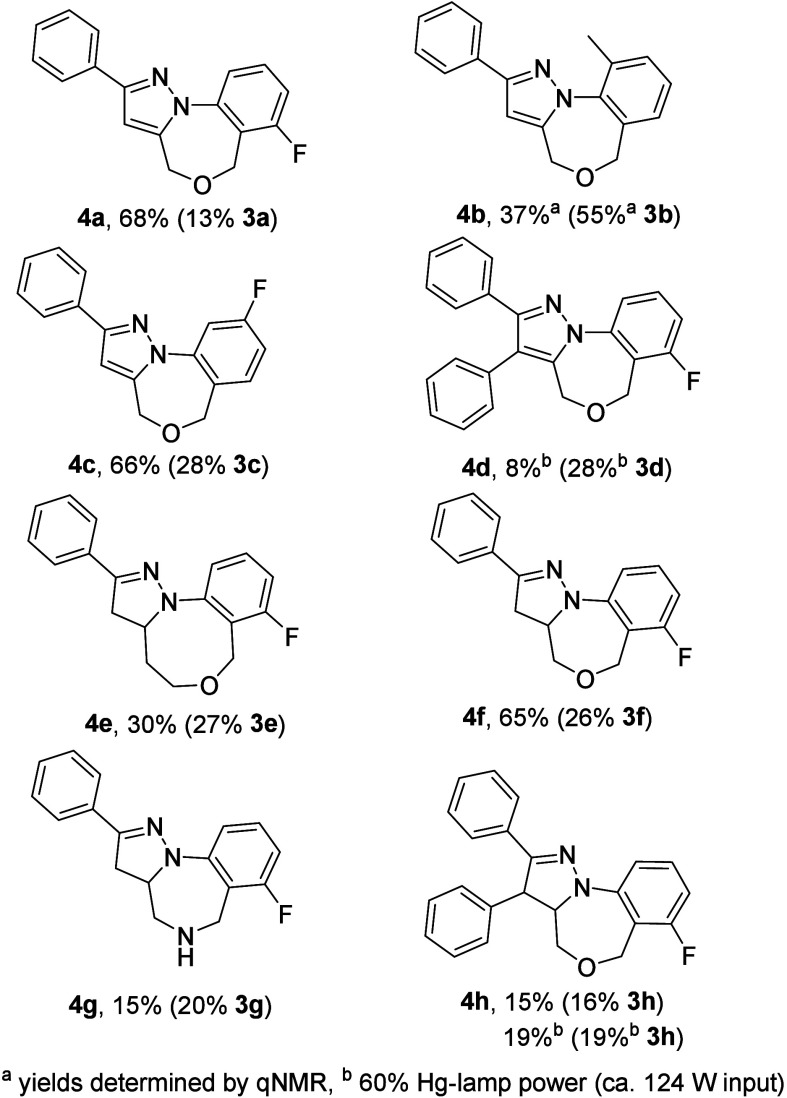
Product scope toward tricyclic pyrrole and pyrazoline products using the photo-click approach.

In addition, we exploited single crystal X-ray diffraction experiments to unambiguously establish the connectivity for pyrazole **4c** and pyrazoline **4f** (Figure 2). These structures furthermore highlight the distinct three-dimensional character of these scaffolds due to the conformation of the oxazine moiety.

**Figure 2:**
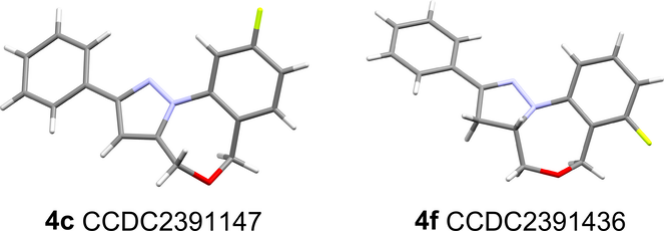
X-ray structures of products **4c** and **4f**.

Next, we wished to investigate the analogous amine tethered tetrazole scaffolds and subjected a tetrazole substrate bearing a tethered allyl amine moiety to the photo-click conditions. Unexpectedly, a new product was isolated in a yield of 36% in addition to pyrazoline **4g** (15%) and unreacted substrate (20%). Spectroscopic characterization by ^1^H NMR revealed that for this new product, the allyl group was unaffected while HRMS indicated the expected loss of dinitrogen. Based on this data the structure of this new product was assigned to be based on a dihydro-1*H*-benzo[f][1,2,4]triazepine scaffold which was subsequently confirmed by single crystal X-ray diffraction as shown in Scheme 3.

**Scheme 3:**
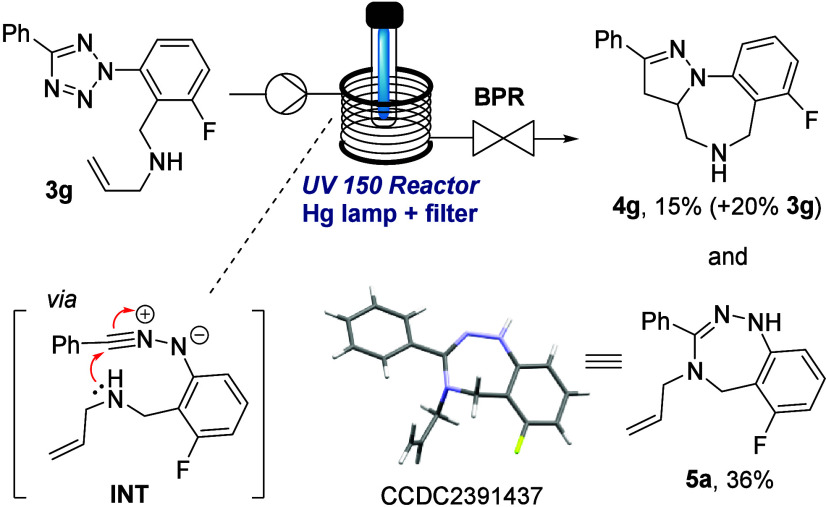
Synthesis of Novel Benzotriazepine Scaffold **5a**

The generation of this benzotriazepine ring system is noteworthy as this scaffold is only known in previous reports as an oxidized analog.^15^ While this finding warrants further mechanistic investigations, we surmise that the photochemically generated nitrile imine intermediate which can exist in multiple canonical forms^16^ reacts via a different resonance form compared to the benzylic ether analogs (Scheme 3). A plausible mechanism may be based on a propargylic form of the nitrile imine being nucleophilically attacked by the secondary amine followed by proton transfer. Crucially, the availability of the N–H proton and the increased nucleophilicity of the amine nitrogen compared to the ether oxygen appears to facilitate the overall process.

To establish whether this photo-click approach provides a general route to these intriguing scaffolds we subjected a small set of tetrazole substrates to the analogous reaction conditions which pleasingly afforded the desired products in good chemical yields (Scheme 4). This furthermore highlighted that alkyl, ester and alkene appendages on the benzylic amine are well tolerated. In the case of *N*-aryl appendages the desired products (i.e., **5e**,**f**) were not observed indicating that either their reduced nucleophilicity or competitive absorption hampers the transformation. In general, the underlying flow process thereby provides fast (t_Res_ = 2 min) and facile access to these unprecedented scaffolds with productivities of 1.68 mmol/h which will enable further synthetic and medicinal explorations.

**Scheme 4:**
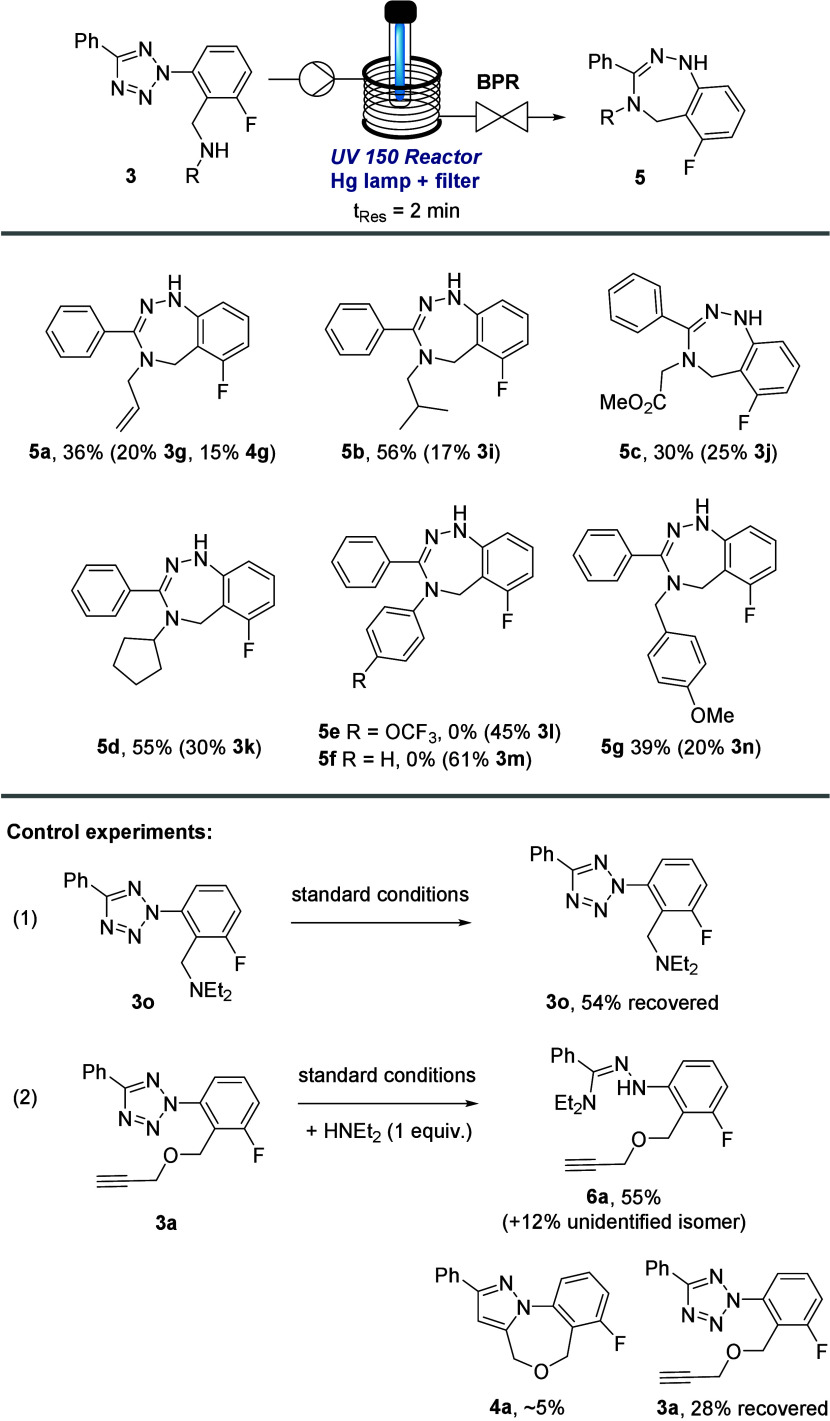
Reaction Scope toward Benzotriazepines via the Photo-Click Approach in Flow Mode (*t*_Res_ = 2 min)

In addition, control experiments were performed to test the reactivity of the tetrazole scaffold in the presence of amines. When a tertiary amine was attached to the benzylic position (i.e., substrate **3o** in Scheme 4, reaction (1)) no productive reaction was observed under standard conditions (i.e., ca. 46% substrate decomposition by qNMR). Alternatively, when substrate **3a** and an external amine such as HNEt_2_ were subjected to the standard conditions two new adducts were isolated that were tentatively assigned as structures **6a** and an unidentified isomer based on NMR and HRMS data. Only small amounts of substrate **3a** and cycloadduct **4a** were observed in this case (Scheme 4, reaction (2)) indicating that the intramolecular cycloaddition is not favored over intermolecular reactions between the nitrile imine intermediate and an external amine.

In conclusion, we report the synthesis of medicinally relevant nitrogen-rich ring systems exploiting a robust photo-click approach. Aryl tetrazole substrates bearing ether or amine appendages are thereby subjected to photolysis conditions generating the reactive nitrile imine intermediate. Tricyclic pyrazole and pyrazoline scaffolds are obtained via a dipolar cycloaddition process when using propargyl or allyl ethers as tethers. In contrast, a new reactivity was discovered when employing secondary amines instead of ethers affording a facile and unprecedented entry to previously elusive benzotriazepines in high chemical yields. Both transformations exploit the use of a photochemical flow reactor setup which selectively yields the targets in short residence times and with high productivities. Overall, this study demonstrates the streamlined generation of new heterocyclic scaffolds exploiting the unique reactivity of nitrile imines generated via photochemical click reactions.

## Data Availability

The data underlying this study are available in the published article and its Supporting Information.

## Supporting Information Available

Complete experimental procedures, compound characterization, and pictures of the flow equipment (PDF)
FAIR data, including the primary NMR FID files, for compounds **1a**–**c**, **2a**–**e**, **3a**–**n**, **4a**–**h**, **5a**–**d**, and **5g** (ZIP)
