# Supplementary material for: Structurally Diverse Nitrogen-Rich Scaffolds via Continuous Photo-Click Reactions
Source: Org Lett. 2024 Nov 26;26(49):10559–63. doi: 10.1021/acs.orglett.4c03953 (PMC11650770; doi:10.1021/acs.orglett.4c03953)
Supplement: Supplementary file 1 — ol4c03953_si_001.pdf [file ol4c03953_si_001.pdf]

Structurally Diverse Nitrogen-rich Scaffolds *via* Continuous Photo-Click Reactions

Davin Cronly,[a] Megan Smyth,[b] Thomas S. Moody,[b,c] Scott Wharry,[b] Julia Bruno-Colmenarez,[a] Brendan Twamley,[d] Marcus Baumann*[a]

[a] School of Chemistry, University College Dublin, O'Brien Centre for Science, Belfield, Dublin 4, Dublin, Ireland

[b] Almac Sciences, Technology Department, Craigavon, BT63 5QD, UK

[c] Arran Chemical Company, Monksland Industrial Estate, Roscommon N37 DN24, Ireland

[d] School of Chemistry, Trinity College Dublin, Dublin 2, Ireland

Email: marcus.baumann@ucd.ie

Table of Contents

General materials and methods 3
Reactor setups 4
Differential scanning calorimetry (DSC) for compounds **1b** and **1c** 5
Photo-Click reaction Optimisation. 6
General procedure for the synthesis of tetrazole compounds (**GP1**) 7
Characterisation data of compound **1a-c** 7
General procedure for the bromination of tetrazole substrates (**GP2**) 8
Characterisation data of compounds **2a-e** 8
General Procedure for the synthesis of ether alkyne and alkene substrates (**GP3**) 10
General Procedure for the synthesis of secondary amine substrates (**GP4**) 10
Characterisation data of compounds **3a-n** 10
General Procedure for Photo-click reaction (**GP5**)....17
Characterisation data of compound **4a-h** 18
Characterisation data of compounds **5a-h** 21
Control experiment 25
X-Ray Data for **4c**, **4f** and **5a** 27
NMR Spectra 35
References. 85

General materials and methods

Unless otherwise stated, all solvents were purchased from Fisher Scientific and used without further purification. Also, unless otherwise stated, all substrates and reagents were purchased from Fluorochem, Sigma-Aldrich or TCI and used as received. ^{1}H NMR spectra were recorded on 400 MHz instruments and are reported relative to the residual solvent: $CHCl_3$ (δ 7.26 ppm). ^{13}C{1H} NMR spectra were recorded on the same instruments (100 and 125 MHz) and are reported relative to $CHCl_3$ (δ 77.0 ppm) or DMSO-d_6 (δ 39.52 ppm). ^{19}F NMR were recorded at 376 MHz.

Data for ^{1}H NMR are reported as follows: chemical shift (δ/ ppm) (integration, multiplicity, coupling constant (Hz)). Multiplicities are reported as follows: s = singlet, d = doublet, t = triplet, q = quartet, p = pentet, m = multiplet, br s = broad singlet, app = apparent. Data for ^{13}C{^{1}H} NMR are reported in terms of chemical shift (δ/ppm) and multiplicity (C, ArCH, CH, CH_2, or CH_3). Data for ^{19}F NMR are reported in terms of chemical shift (δ/ppm). COSY, HSQC and HMBC experiments were used in the structural assignment.

High-resolution mass spectrometry was performed using the indicated techniques on a micromass LCT orthogonal time-of-flight mass spectrometer with leucine-enkephalin (TyrGlyPhe-Leu) as an internal lock mass. IR spectra were obtained by use of a Bruker Platinum spectrometer (neat, ATR sampling) with the intensities of the characteristic signals being reported as weak (w, <21% of the tallest signal). signal), medium (m, 21–70% of the tallest signal), or strong (s, >71% of the tallest signal).

Continuous flow experiments were performed on a Vapourtec E-Series system equipped with peristaltic pumps and a dynamic BPR achieved through utilization of a peristaltic pump in a reverse direction (1-9 bar, Vapourtec). For photochemical experiments the UV-150 module (Vapourtec) was used in combination with a high-power LED (365 nm, purchased from Vapourtec) tuned at the desired W (0-100 Watt) or a medium-pressure Hg-lamp and cooled to 25-30 °C by passing a stream of compressed air through the reactor unit. A low-pass filter was used in combination with the Hg lamp, no filter was used with the 365 nm lamp. Reactor coils were made of PFA (perfluoroalkoxy polymer) tubing (i.d. 1/16 inch) with a volume of 10 mL.

TLC was performed on Merck pre-coated Silica gel 60 F254 aluminium plates with realisation by UV irradiation at 254 nm, $KMnO_4$ and vanillin stain. Flash chromatography was performed using Macherey-Nagel silica gel 60 M, with a particle range of 0.04 - 0.063 mm

Reactor setups

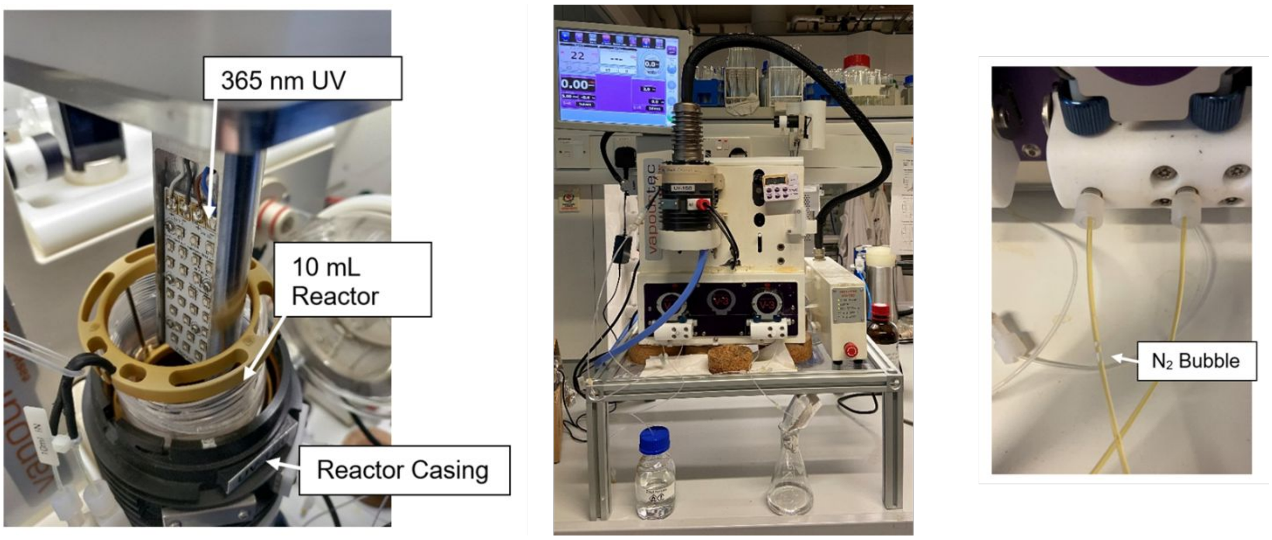

Figure S1: Images of Photoreactor Coil, LED and Pump Details.

Differential scanning calorimetry (DSC) for compounds **1b** and **1c**

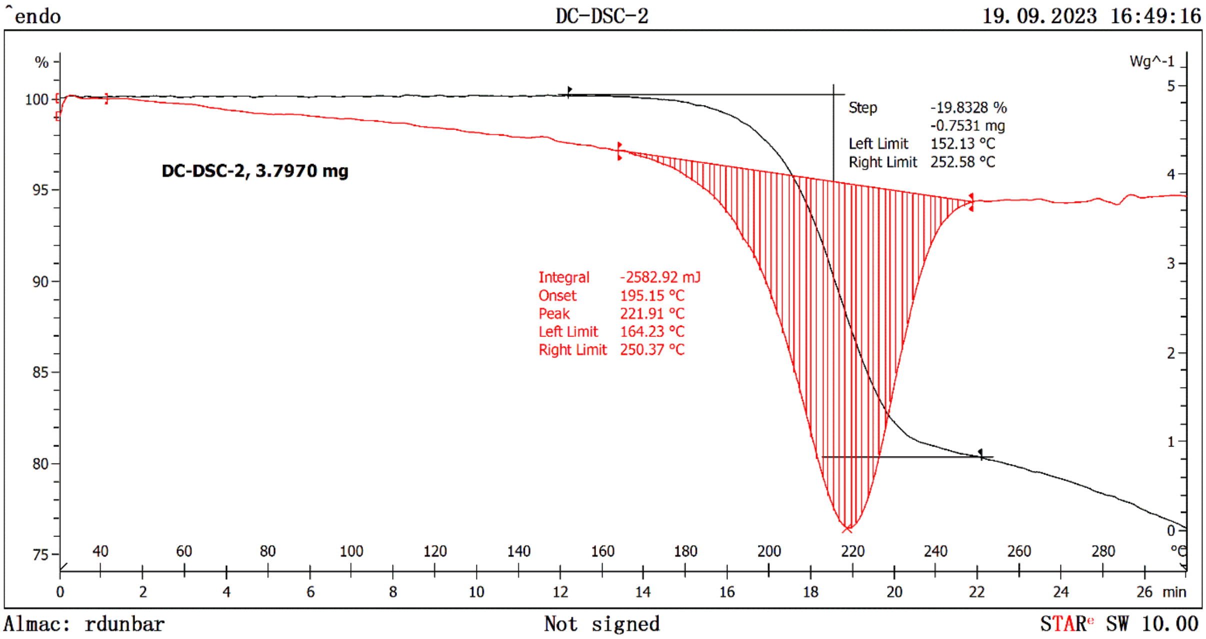

Figure SI1: DSC analysis of compound **1b.**

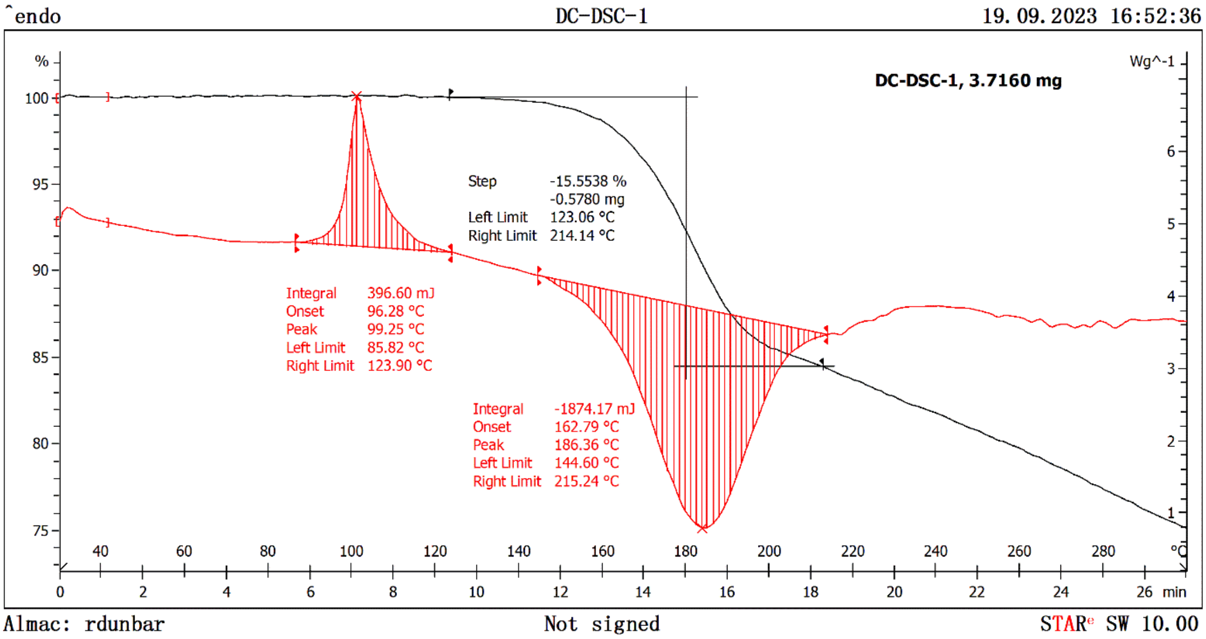

Figure SI2: DSC analysis of compound **1c**.

Photo-Click reaction Optimisation.

Table 1: Optimisation of reaction conditions for Photo-Click reaction. Yields were determined by internal standard ^{1}H NMR.

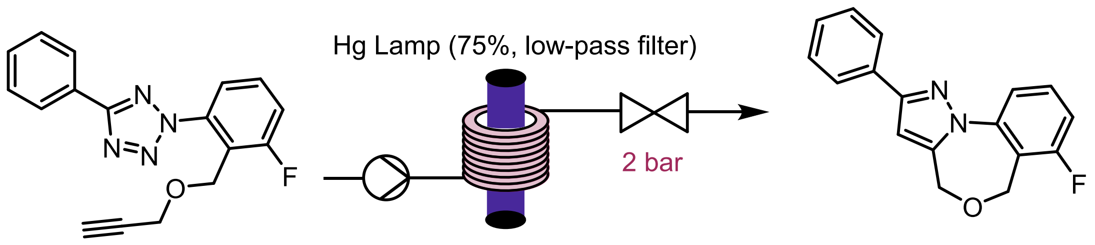

Entry	Solvent	Concentration (mM)	Residence time (min)	Product Yield	Starting material
**1**	MeCN	10	2	43%	50%
**2**	Acetone	10	2	54%	18%
**3**	Acetone	10	1	53%	42%
**4**	Toulene	10	2	77%	20%
**5**	Xylene	10	2	76%	13%
**6**	EtOAc	10	2	71%	20%
**7**	EtOAc	20	2	54%	27%
**8**	EtOAc	30	2	51%	36%
**9**	EtOAc	40	2	48%	42%
**10**	EtOAc	50	2	46%	44%
**11**	EtOAc	70	2	41%	50%
**12**	EtOAc	100	2	37%	57%
**13**	EtOAc	100	3	52%	47%
**14**	EtOAc	100	4	55%	41%
**15**	EtOAc	100	5	59%	32%

General procedure for the synthesis of tetrazole compounds (**GP1**)

A solution of aniline (1 equiv. 4M) was prepared by drop-wise addition to HCl (12 M) at 0 °C and stirred for 5 minutes. $NaNO_2$ (1 equiv. in H_2O, 4 M) was slowly added to the suspension and left to stir at 0 °C for 10 minutes. $NaBF_4$ (1.6 equiv. in H_2O, 5 M) was added to the solution and stirred for 10 minutes. The solid suspension was isolated by vacuum filtration and washed with dilute $NaBF_4$ (5% w/w) to afford the crude diazonium salt.

The crude diazonium salt was added in portions to a solution of benzamidine hydrochloride (1 equiv.) and K_2CO_3 (5 equiv.) in DMSO (0.2 M). The solution was left to stir for 1 hour at room temperature. A solution of I_2 (1.2 equiv.) and NaI (1.5 equiv.) in DMSO was added to the stirring solution and left to stir for an additional 1 hour at room temperature. The reaction as quenched using a saturated $Na_2S_2O_3$ solution. The aqueous solution was extracted with EtOAc (3 x 30 mL). Purification by column chromatography (5% EtOAc: pentane) yielded the desired product over two steps.[1, 2]

Characterisation data of compound **1a-c**

2-(3-Fluoro-2-methylphenyl)-5-phenyl-2*H*-tetrazole (**1a**)

Following the procedure outline in **GP1**. 2-Methyl-3-fluoroaniline (3.0 g, 24 mmol) was used to obtain **1a** as a white solid in 53% (3.26 g 12.8 mmol) over two steps.

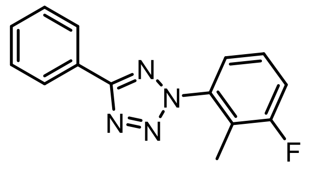

Chemical Formula: $C_{14}H_{11}FN_4$
Exact Mass: 254.0968

Appearance: White Solid

Yield: 53%, 3.26 g (12.8 mmol)

^{1}H NMR (400 MHz, $CDCl_3$) δ 8.30 – 8.20 (m, 2H), 7.58 – 7.46 (m, 4H), 7.38 (tdd, *J* = 8.0, 5.7, 0.7 Hz, 1H), 7.30 – 7.23 (m, 1H), 2.35 (dd, *J* = 2.3, 0.5 Hz, 3H). **^{13}C NMR (101 MHz, $CDCl_3$)** δ 163.9 (d, *J* = 240 Hz, ArCF), 130.7 (ArCH), 129.0 (2 ArCH), 127.4 (d, *J* = 9 Hz, ArCH), 127.0 (2 ArCH),127.0 (C), 121.5 (C), 121.0 (d, *J* = 4 Hz, ArC), 117.1 (d, *J* = 23 Hz, ArCH), 10.6 (d, *J* = 5 Hz, CH_3). **IR (solid)** 1618 (w), 1588 (w), 1530 (w), 1475 (m), 1450 (m), 1248 (m), 1172 (m), 1150 (m), 1008 (m), 992 (m), 860 (m), 783 (m), 726 (s), 688 (s), 494 (w). **HRMS** (QTOF) m/z: $[M+H]^+$ Calcd for $C_{14}H_{12}FN_4^+$ 255.1042; Found 255.1041.

2-(2,6-Dimethylphenyl)-5-phenyl-2*H*-tetrazole (**1b**)

Following the procedure outlined in **GP1**. 2,6-Dimethylaniline (1.45, 12 mmol) was used to obtain **1b** as an orange oil in 35% (1.04 g, 4.2 mmol) over two steps.

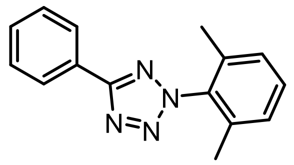

Chemical Formula: $C_{15}H_{14}N_4$
Exact Mass: 250.1218

Appearance: Orange oil

Yield: 35%, 1.04 g (4.2 mmol)

^{1}H NMR (400 MHz, $CDCl_3$) δ 8.28 – 8.24 (m, 2H), 7.58 – 7.45 (m, 3H), 7.39 (dd, *J* = 8.1, 7.1 Hz, 1H), 7.26 – 7.20 (m, 2H), 2.07 (s, 6H). **^{13}C NMR (101 MHz, $CDCl_3$)** δ 165.2 (C), 136.4 (C), 135.6 (2C), 130.9 (ArCH), 130.6 (ArCH), 129.1 (2ArCH), 128.7 (2ArCH), 127.4 (C), 127.1 (2ArCH), 17.5 (2CH_3). **IR (neat, oil)** 3070 (w), 2926 (w), 1527 (m), 1464 (s), 1448 (s), 1361 (m), 1208 (w), 1012 (s), 994 (m), 923 (w), 773 (m), 730 (s), 691 (s), 507 (m) **HRMS** (QTOF) m/z: $[M+H]^+$ Calcd for $C_{15}H_{15}N_4^+$ 251.1291; Found 251.1292.

2-(5-Fluoro-2-methylphenyl)-5-phenyl-2*H*-tetrazole (**1c**)

Following the procedure outlined in **GP1**. 2-Methyl-5-fluoroaniline (3.00 g, 24 mmol) was used to obtain compound **1c** as a white solid in 29% (1.75g, 6.88 mmol) over two steps.

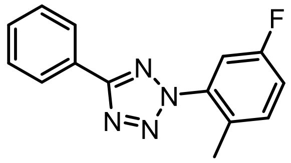

Chemical Formula: $C_{14}H_{11}FN_4$
Exact Mass: 254.0968

Appearance: White Solid

Yield: 25%, 761 mg (2.99 mmol)

^{1}H NMR (400 MHz, $CDCl_3$) δ 8.30 – 8.20 (m, 2H), 7.59 – 7.46 (m, 4H), 7.39 (ddd, *J* = 8.7, 5.8, 0.9 Hz, 1H), 7.18 (td, *J* = 8.2, 2.7 Hz, 1H), 2.45 (t, *J* = 1.0 Hz, 3H). **^{13}C NMR (101 MHz, $CDCl_3$)** δ 165.1 (C), 160.9 (d, *J* = 247 Hz, ArCF), 136.8 (d, *J* = 9 Hz C), 133.3 (d, *J* = 9 Hz, ArCH), 130.8 (ArCH), 129.1 (2ArCH), 128.6 (d, *J* = 4 Hz, C), 127.1 (2ArCH), 127.1 (C), 117.3 (d, *J* = 21 Hz, ArCH), 112.5 (d, *J* = 26 Hz, ArCH), 18.7 (CH_3). **^{19}F NMR (376 MHz, $CDCl_3$)** δ -114.64 (m). **IR (solid)** 3079 (w), 3054 (w), 1899 (w), 1609 (m), 1505 (s), 1451 (s), 1225 (m), 1183 (m), 1125 (w), 1018 (s), 996 (m), 889 (m), 873 (m), 813 (s), 729 (s), 691 (s), 478 (m). **HRMS** (QTOF) m/z: $[M+H]^+$ Calcd for $C_{14}H_{12}FN_4^+$ 255.1041; Found 255.1040.

General procedure for the bromination of tetrazole substrates (**GP2**)

To a solution of tetrazole substrate (1 equiv.) in MeCN (0.1M), *N*-bromosuccinimide (1.5 equiv) was added. The solution was passed with a flow rate of 0.5 mL min^{-1} through the Vapourtec E-Series UV150 reactor (10 mL volume, 2 minute residence time) irradiating with 365 nm (100 W) light. Excess bromine was quenched using a saturated $Na_2S_2O_3$ solution, the solution was concentrated *in vacuo* and the extracted using EtOAc and water. The organic layer was dried over anhydrous Na_2SO_4 and concentrated *in vacuo*. The product was isolated and purified using column chromatography (5% EtOAc: pentane).

Characterisation data of compounds **2a-e**

2-(2-(Bromomethyl)-3-fluorophenyl)-5-phenyl-2*H*-tetrazole (**2a**)

Following the procedure outlined in **GP2**. **1a** (2.1 g, 8.25 mmol) was used to obtain **2a** as an orange solid in 85% (2.32 g, 6.69 mmol).

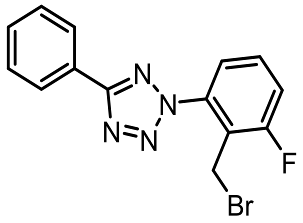

Chemical Formula: $C_{14}H_{10}BrFN_4$
Exact Mass: 332.0073

Appearance: Orange solid

Yield: 85%, 2.32 g (6.69 mmol)

^{1}H NMR (400 MHz, $CDCl_3$) δ 8.32 – 8.22 (m, 2H), 7.72 (dt, *J* = 8.2, 1.2 Hz, 1H), 7.59 – 7.48 (m, 4H), 7.31 (ddd, *J* = 9.4, 8.4, 1.2 Hz, 1H), 4.89 (d, *J* = 1.7 Hz, 2H). **^{13}C NMR (101 MHz, $CDCl_3$)** δ 165.4 (C), 161.2 (d, *J* = 252 Hz, ArCF) , 136.6 (d, *J* = 5 Hz, C), 130.9 (ArCH), 130.6 (d, *J* = 10 Hz, ArCH), 129.1 (2 x ArCH), 127.2 (2 x ArCH), 126.7 (C), 120.9 (d, *J* = 4 Hz, ArCH), 120.7 (d, *J* = 18 Hz, C), 117.6 (d, *J* = 23 Hz, ArCH), 20.3 (d, *J* = 6 Hz, CH_2). **^{19}F NMR (376 MHz, $CDCl_3$)** δ -112.23 (m). **IR (neat, solid)** 3067(w), 1613 (w), 1588 (w), 1470 (m), 1447 (m), 1251 (m), 1226 (m), 1184 (m), 1067 (m), 1019 (w), 909 (s), 795 (s), 724 (s), 684 (s), 614 (s), 587 (m), 493 (m) **HRMS** (QTOF) m/z: $[M+H]^+$ Calcd for $C_{14}H_{10}BrFN_4^+$ 333.0146; Found 333.0146.

2-(2,6-bis(bromomethyl)phenyl)-5-phenyl-2*H*-tetrazole (**2b**)

Following the procedure outlined in **GP2. 1b** (276 mg, 1.103 mmol) was used to obtain **2b** as a pale-yellow oil in 62% (225 mg, 0.68 mmol).

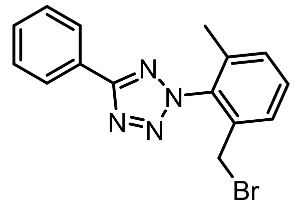

Chemical Formula: $C_{15}H_{13}BrN_4$
Exact Mass: 328.0324

Appearance: Pale-yellow oil

Yield: 62% 225 mg (0.68 mmol)

^{1}H NMR (400 MHz, CDCl$_3$) δ 8.33 – 8.23 (m, 2H), 7.58 – 7.50 (m, 3H), 7.50 – 7.45 (m, 2H), 7.38 (ddd, *J* = 6.4, 2.9, 0.8 Hz, 1H), 4.28 (s, 2H), 2.13 (d, *J* = 0.7 Hz, 3H). **^{13}C NMR (101 MHz, CDCl$_3$)** δ 165.3 (C), 136.4 (C), 135.5 (C), 134.7 (C), 131.6 (ArCH), 131.2 (ArCH), 130.7 (ArCH), 129.0 (2 ArCH), 128.8 (ArCH), 127.1 (2 ArCH), 127.0 (C), 27.5 (CH_2), 17.7 (CH_3). **IR (neat, oil)** 3040 (w), 1527 (m), 1475 (m), 1445 (m), 1360 (w), 1221 (m), 1163 (w), 1071 (w), 1013 (s), 790 (m), 770 (w), 749 (m), 730 (s), 691 (s), 619 (m) 605 (m), 513 (m), 493 (m). **HRMS** (QTOF) m/z: $[M+H]^+$ Calcd for $C_{15}H_{14}BrN_4^+$ 329.0396; Found 329.0397.

2-(2,6-Bis(bromomethyl)phenyl)-5-phenyl-2*H*-tetrazole (**2c**)

Following the procedure outlined in **GP2** deviations were 2.5 equivalents of NBS. **1b** (291 mg, 1.16 mmol) was used to obtain **2c** as an orange solid in 54% (256 mg, 0.63 mmol).

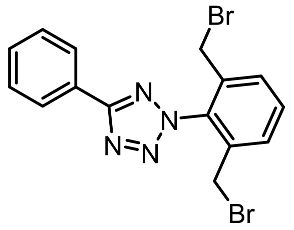

Chemical Formula: $C_{15}H_{12}Br_2N_4$
Exact Mass: 405.9429

Appearance: Orange solid

Yield: 54% 256 mg (0.63 mmol)

^{1}H NMR (400 MHz, CDCl$_3$) δ 8.33 – 8.25 (m, 2H), 7.65 – 7.49 (m, 6H), 4.38 (s, 4H). **^{13}C NMR (101 MHz, CDCl$_3$)** δ 165.6 (C), 135.5 (C), 135.1 (2 C), 131.7 (2 ArCH), 131.7 (ArCH), 130.9 (ArCH), 129.2 (2 ArCH), 127.3 (2 ArCH), 126.9 (C), 27.4 (2 CH_2). **IR (solid)** 2919 (w), 1447 (m), 1208 (m), 1012 (m), 982 (m), 867 (m), 800 (m), 751 (s), 728 (s), 687 (s), 589 (s), 503 (m) **HRMS** (QTOF) m/z: $[M+H]^+$ Calcd for $C_{15}H_{13}Br_2N_4^+$ 406.9501; Found 406.9501.

2-(2-(Bromomethyl)-5-fluorophenyl)-5-phenyl-2*H*-tetrazole (**2d**)

Following the procedure outlined in **GP2**. **1c** (309 mg, 1.22 mmol) was used to obtain **2d** as an orange solid in 61% (253 mg, 0.759 mmol).

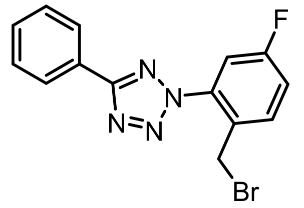

Chemical Formula: $C_{14}H_{10}BrFN_4$
Exact Mass: 332.0073

Appearance: orange solid.

Yield: 61%, 253 mg (0.759 mmol)

^{1}H NMR (400 MHz, CDCl$_3$) δ 8.29 – 8.25 (m, 2H), 7.70 – 7.60 (m, 2H), 7.58 – 7.51 (m, 3H), 7.25 (ddd, *J* = 8.7, 7.5, 2.7 Hz, 1H), 4.87 (s, 2H). **^{13}C NMR (101 MHz, CDCl$_3$)** δ 165.4 (C), 162.4 (d, *J* = 252 Hz, ArCF), 136.3 (C), 133.8 (d, *J* = 9 Hz ArCH), 130.9 (ArCH), 129.0 (2 ArCH), 127.5 (d, *J* = 4 Hz, ArCH)), 127.2 (2 ArCH), 126.7 (C), 117.5 (d, *J* = 21 Hz, ArCH), 112.7 (d, *J* = 26 Hz, ArCH), 28.7 ($BrCH_2$). **^{19}F NMR (376 MHz, CDCl$_3$)** δ -109.23 (m). **IR (solid)** 2963 (m), 1611 (m), 1530 (s), 1508 (m), 1467 (m), 1451 (s), 1260 (m), 1231 (m), 1016 (s), 900 (s), 868 (m), 799 (m), 730 (s), 691 (s), 612 (m), 487 (w). **HRMS** (QTOF) m/z: $[M+H]^+$ Calcd for $C_{14}H_{11}BrFN_4^+$ 333.0146; Found 333.0143.

3-Phenylprop-2-yn-1-ol (**2e**)

Following a previously reported procedure the compound **2e** was obtained in 32% (790 mg, 6 mmol) as a pale-yellow oil. Matches published data.[3]

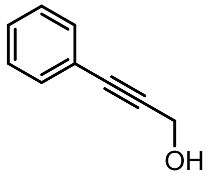

Chemical Formula: C_9H_8O
Exact Mass: 132.0575

Appearance: pale yellow oil

Yield: 32% 790 mg (6.0 mmol)

^{1}H NMR (400 MHz, $CDCl_3$) δ 7.49 – 7.39 (m, 2H), 7.37 – 7.26 (m, 3H), 4.50 (s, 2H), 1.77 (s, 1H).

General Procedure for the synthesis of ether alkyne and alkene substrates (**GP3**)

A solution of allyl/alkyl alcohol (1.2 equiv.) and KO*t*-Bu (1.2 equiv.) in dry THF (0.1 M) under N_2 was prepared at 0 °C and stirred for 10 minutes. To this solution brominated substrate (**2a-d**) (1 equiv.) was added and the reaction solution was stirred overnight. The reaction solution was evaporated under reduced pressure and the crude material was redissolved in EtOAc. The organic layer was washed with water and dried over anhydrous $Na_2S_2O_3$. Purification by column chromatography yielded the products when necessary.

General Procedure for the synthesis of secondary amine substrates (**GP4**)

A solution of amine (1.2 equiv.) and K_2CO_3 (2 equiv.) in MeCN (0.1 M) under N_2 was stirred for 10 minutes. To this solution **2a** (1 equiv.) was added and the reaction solution was stirred overnight. The reaction solution was concentrated *in vacuo* and redissolved in EtOAc. The organic layer was washed with water and dried over anhydrous $Na_2S_2O_3$. Purification by column chromatography yielded the products when necessary.

Characterisation data of compounds **3a-n**

2-(3-Fluoro-2-((prop-2-yn-1-yloxy)methyl)phenyl)-5-phenyl-2*H*-tetrazole (**3a**)

Following the procedure outlined in **GP3**.Propargyl alcohol (95 mg, 1.63 mmol) and **2a** (453 mg, 1.36 mmol) was used to obtain **3a** as a colourless oil in 80% (336 mg, 1.08 mmol) following column chromatography (10% EtOAc: pentane).

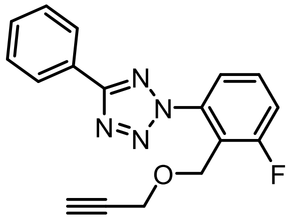

Chemical Formula: $C_{17}H_{13}FN_4O$
Exact Mass: 308.1073

Appearance: Colourless oil

Yield: 80%

^{1}H NMR (400 MHz, $CDCl_3$) δ 8.29 – 8.21 (m, 2H), 7.64 – 7.46 (m, 5H), 7.33 (ddd, *J* = 9.4, 8.2, 1.4 Hz, 1H), 4.90 (d, *J* = 1.8 Hz, 2H), 4.06 (d, *J* = 2.4 Hz, 2H), 2.27 (t, *J* = 2.4 Hz, 1H). **^{13}C NMR (101 MHz, $CDCl_3$)** δ 165.2 (C), 161.8 (d, *J* = 251 Hz, ArCF), 137.7 (d, *J* = 6 Hz, C), 130.7 (ArCH), 130.5 (d, *J* = 10 Hz, ArCH), 129.0 (2 ArCH), 127.1 (2 ArCH), 127.0 (C), 121.4 (d, *J* = 4 Hz, ArCH), 120.3

(d, *J* = 19 Hz, C), 117.7 (d, *J* = 23 Hz, ArCH), 78.8 (C), 74.9 (CH), 60.6 (d, *J* = 5 Hz, CH_2), 57.8 (CH_2). **^{19}F NMR (376 MHz, $CDCl_3$)** δ -113.11 (m). **IR (neat, oil)** 3297 (m, br), 3071 (w), 2948 (w), 1617 (m), 1530 (m), 1471 (s), 1449 (s), 1360 (m), 1245 (s), 1079 (s, br), 1021 (s), 868 (s), 789 (s), 730 (s), 691 (s), 635 (m, br), 497 (m). **HRMS** (QTOF) m/z: $[M+H]^+$ Calcd for $C_{17}H_{14}FN_4O^+$ 309.1146; Found 309.1147.

2-(2-Methyl-6-((prop-2-yn-1-yloxy)methyl)phenyl)-5-phenyl-2*H*-tetrazolet (**3b**)

Following the procedure outlined in **GP3**. Propargyl alcohol (58 mg, 1 mmol) and **2b** (274 mg, 0.83 mmol) was used to obtain **3b** as a colourless oil in 51% (130 mg, 0.43 mmol) following column chromatography (5% EtOAc: pentane).

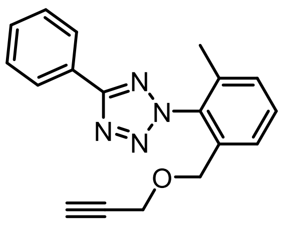

Chemical Formula: $C_{18}H_{16}N_4O$
Exact Mass: 304.1324

Appearance: Colourless oil

Yield: 51%, 130 mg (0.43 mmol)

^{1}H NMR (400 MHz, $CDCl_3$) δ 8.31 – 8.21 (m, 2H), 7.58 – 7.46 (m, 5H), 7.41 – 7.33 (m, 1H), 4.38 (s, 2H), 4.02 (d, *J* = 2.4 Hz, 2H), 2.28 (t, *J* = 2.4 Hz, 1H), 2.11 (d, *J* = 0.7 Hz, 3H). **^{13}C NMR (101 MHz, $CDCl_3$)** δ 165.1(C), 135.7 (C), 135.3 (C), 134.8 (C), 131.0 (ArCH), 130.7 (ArCH), 130.6 (ArCH), 129.0 (2 ArCH), 127.2 (ArCH), 127.0 (2 ArCH),127.0 (C), 78.9 (C), 74.9 (CH), 67.1 (CH_2), 57.5 (CH_2), 17.4 (CH_3). **IR (neat, oil)** 3293 (m, br), 3071(w), 2855 (w), 1528 (w), 1448 (m), 1361 (m), 1081 (s), 1013 (s), 788 (m), 731 (s), 692 (s), 509 (w). **HRMS** (QTOF) m/z: $[M+H]^+$ Calcd for $C_{18}H_{17}N_4O^+$ 305.1397; Found 305.1406.

2-(5-Fluoro-2-((prop-2-yn-1-yloxy)methyl)phenyl)-5-phenyl-2*H*-tetrazole (**3c**)

Following the procedure outlined in **GP3**. Propargyl alcohol (70 mg, 1.2 mmol) and **2c** (334 mg, 1.0 mmol) was used to obtain **3c** following organic extraction as a white solid in 96% (297 mg, 0.96 mmol).

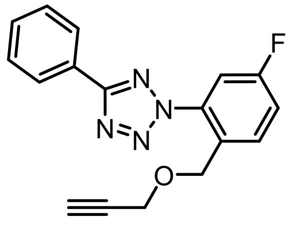

Chemical Formula: $C_{17}H_{13}FN_4O$
Exact Mass: 308.1073

Appearance: White Solid

Yield: 96%, 297 mg (0.96 mmol)

^{1}H NMR (400 MHz, $CDCl_3$) δ 8.30 – 8.19 (m, 2H), 7.75 (dd, *J* = 8.7, 5.9 Hz, 1H), 7.63 (dd, *J* = 8.8, 2.6 Hz, 1H), 7.57 – 7.47 (m, 3H), 7.32 – 7.21 (m, 1H), 4.87 (s, 2H), 4.18 (d, *J* = 2.4 Hz, 2H), 2.39 (t, *J* = 2.4 Hz, 1H). **^{13}C NMR (101 MHz, $CDCl_3$)** δ 165.2 (C), 162.0 (d, *J* = 249 Hz, ArCF), 135.8 (d, *J* = 10 Hz, C), 131.6 (d, *J* = 8 Hz, ArCH), 130.9 (ArCH), 129.1 (2 ArCH), 127.8 (d, *J* = 3 Hz, C), 127.2 (2 ArCH), 126.9 (C), 117.3 (d, *J* = 21 Hz, ArCH), 112.0 (d, *J* = 26 Hz, ArCH), 79.1 (C), 75.2 (CH), 67.6 (CH_2), 58.0 (CH_2). **^{19}F NMR (376 MHz, $CDCl_3$)** δ -111.64 (m). **IR (solid)** 3288 (s), 3063 (w), 2902 (w), 2858 (w), 2116 (w), 1608 (m), 1506 (s), 1449 (s), 1364 (m), 1213 (s), 1178 (s), 1090 (s), 1015 (s), 954 (m), 837 (m), 705 (s), 680 (s), 656 (s). **HRMS** (QTOF) m/z: $[M+H]^+$ Calcd for $C_{17}H_{14}FN_4O^+$ 309.1146; Found 309.1146.

2-(3-Fluoro-2-(((3-phenylprop-2-yn-1-yl)oxy)methyl)phenyl)-5-phenyl-2*H*-tetrazole (**3d**)

Following the procedure outlined in **GP3**. **2e** (159 mg, 1.2 mmol) and **2a** (345 mg, 1.06 mmol) was used to obtain **3e** as a pale-yellow oil in 25% (101 mg, 026 mmol) following column chromatography (5% EtOAc: pentane).

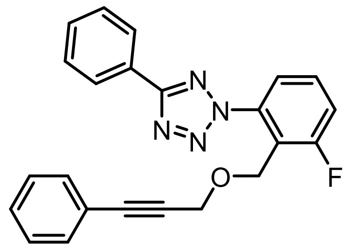

Chemical Formula: $C_{23}H_{17}FN_4O$
Exact Mass: 384.1386

Appearance: pale-yellow oil

Yield: 25%, 101 mg (0.26 mmol)

^{1}H NMR (400 MHz, $CDCl_3$) δ 8.27 – 8.16 (m, 2H), 7.60 (dt, *J* = 8.0, 1.2 Hz, 1H), 7.54 (td, *J* = 8.1, 5.6 Hz, 1H), 7.49 – 7.45 (m, 3H), 7.40 – 7.18 (m, 6H), 4.97 (d, *J* = 1.8 Hz, 2H), 4.29 (s, 2H). **^{13}C NMR (126 MHz, $CDCl_3$)** δ 165.3 (C), 161.9 (d, *J* = 250 Hz, ArCF), 137.8 (d, *J* = 6 Hz, C), 131.7 (2 ArCH), 130.6 (ArCH), 130.5 (d, *J* = 10 Hz, ArCH), 129.0 (2 ArCH), 128.3 (ArCH), 128.1 (2 ArCH), 127.1 (2 ArCH), 126.9 (C), 122.4 (C), 121.4 (d, *J* = 4 Hz, ArCH), 120.6 (d, *J* = 18 Hz, C), 117.7 (d, *J* = 23 Hz, ArCH), 86.8 (C), 84.2 (C), 60.6 (d, *J* = 5 Hz, CH_2), 58.6 (CH_2). **^{19}F NMR (470 MHz, $CDCl_3$)** δ -113.03 (m). **IR (neat, oil)** 3060 (w), 2888 (w), 2848 (w), 1728 (w), 1617 (m), 1590 (w), 1530 (w), 1471 (s), 1449 (s), 1353 (m), 1246 (m), 1073 (s), 1021 (m), 870 (m), 790 (s), 756 (s), 690 (s), 587 (w), 525 (w). **HRMS** (QTOF) m/z: $[M+H]^+$ Calcd for $C_{23}H_{18}FN_4O^+$ 385.1459; Found 385.1459.

2-(2-((But-3-en-1-yloxy)methyl)-3-fluorophenyl)-5-phenyl-2*H*-tetrazole (**3e**)

Following the procedure outlined in **GP3**. 3-buten-1-ol (87 mg, 1.2 mmol) and **2a** (333 mg, 1.0 mmol) was used to obtain **3e** following organic extraction as a pale-yellow oil in 92% (300 mg, 0.92 mmol).

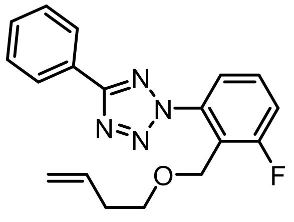

Chemical Formula: $C_{18}H_{17}FN_4O$
Exact Mass: 324.1386

Appearance: pale yellow oil

Yield: 92% 300 mg (0.92 mmol)

^{1}H NMR (400 MHz, $CDCl_3$) δ 8.28 – 8.22 (m, 2H), 7.60 – 7.47 (m, 5H), 7.31 (ddd, *J* = 9.4, 8.3, 1.4 Hz, 1H), 5.58 (ddt, *J* = 17.0, 10.2, 6.7 Hz, 1H), 4.94 – 4.83 (m, 2H), 4.80 (d, *J* = 1.8 Hz, 2H), 3.38 (t, *J* = 6.7 Hz, 2H), 2.12 (qt, *J* = 6.7, 1.4 Hz, 2H). **^{13}C NMR (101 MHz, $CDCl_3$)** δ 165.1 (C), 161.5 (d, *J* = 250 Hz, ArCF), 137.7 (d, *J* = 6 Hz, C), 134.7 (CH), 130.6 (ArCH), 130.1 (d, *J* = 10 Hz, ArCH), 129.0 (2 ArCH),127.0 (C), 127.0 (2 ArCH), 121.4 (d, *J* = 4 Hz, ArCH), 121.2 (d, *J* = 18 Hz, C), 117.7 (d, *J* = 23 Hz, ArCH), 116.3 (CH_2), 70.2 (CH_2), 61.8 (d, *J* = 5 Hz, CH_2), 33.9 (CH_2). **^{19}F NMR (376 MHz, $CDCl_3$)** δ -113.79 (m). **IR (neat, oil)** 3074 (w), 2978 (w), 2871 (w), 1641 (m), 1617 (m), 1589 (m), 1530 (w), 1472 (s), 1448 (s), 1362 (m), 1245 (s), 1209 (w), 1095 (s), 990 (m), 873 (m), 789 (s), 730 (s), 691 (s). **HRMS** (QTOF) m/z: $[M+H]^+$ Calcd for $C_{18}H_{17}FN_4O^+$ 325.1459; Found 325.1460.

2-(2-((Allyloxy)methyl)-3-fluorophenyl)-5-phenyl-2*H*-tetrazole (**3f**)

Following the procedure outlined in **GP3**. Allyl alcohol (70 mg, 1.2 mmol) and **2a** (334 mg, 1.0 mmol) was used to obtain **3f** following organic extraction as a pale-yellow oil in 87% (270 mg, 0.87 mmol).

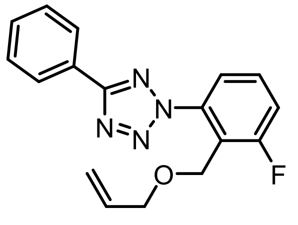

Chemical Formula: $C_{17}H_{15}FN_4O$
Exact Mass: 310.1230

Appearance: pale yellow oil

Yield: 87%, 270 mg (0.87 mmol)

^{1}H NMR (400 MHz, CDCl$_3$) δ 8.30 – 8.20 (m, 2H), 7.61 – 7.47 (m, 5H), 7.32 (ddd, *J* = 9.4, 8.2, 1.4 Hz, 1H), 5.71 (ddt, *J* = 17.2, 10.3, 5.8 Hz, 1H), 5.14 – 5.00 (m, 2H), 4.79 (d, *J* = 1.8 Hz, 2H), 3.88 (dt, *J* = 5.8, 1.4 Hz, 2H). **^{13}C NMR (101 MHz, CDCl$_3$)** δ 165.1 (C), 161.6 (d, *J* = 250 Hz, ArCF), 137.7 (d, *J* = 6 Hz, ArCHz), 134.0 (CH), 130.6 (ArCH), 130.2 (d, *J* = 10 Hz, ArCH), 129.0 (2 ArCH), 127.0 (2 ArCH), 127.0 (C), 121.4 (d, *J* = 4 Hz, ArCH), 121.2 (d, *J* = 18 Hz, C), 117.7 (d, *J* = 23 Hz, ArCH), 117.5 (CH_2), 71.8 (CH_2), 61.0 (d, *J* = 5 Hz, CH_2). **^{19}F NMR (376 MHz, CDCl$_3$)** δ -113.64 (m). **IR (neat, oil)** 3074 (w), 2851 (w), 1618 (m), 1589 (m), 1530 (w), 1471 (s), 1449 (s), 1395 (m), 1246 (s), 1207 (w), 1124 (m), 1071 (s, br), 1023 (m), 989 (s), 924 (m), 869 (m), 789 (s), 730 (s), 691 (s), 558 (w), 499 (w). **HRMS** (QTOF) m/z: $[M+H]^+$ Calcd for $C_{17}H_{16}FN_4O^+$ 311.1303; Found 311.1305.

N-(2-Fluoro-6-(5-phenyl-2*H*-tetrazol-2-yl)benzyl)prop-2-en-1-amine (**3g**)

Following the procedure outlined in **GP4**. Allyl amine (67 mg, 1.2 mmol) and **2a** (333 mg, 1 mmol) was used to obtain **3g** following an organic extraction as a colourless oil in 91% (282 mg, 0.91 mmol)

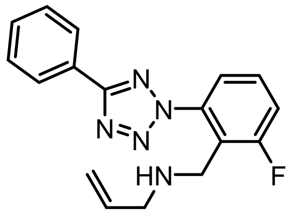

Chemical Formula: $C_{17}H_{16}FN_5$
Exact Mass: 309.1390

Appearance: Colourless oil

Yield: 91%, 282 mg (0.91 mmol)

^{1}H NMR (400 MHz, cdcl$_3$) δ 8.28 – 8.18 (m, 2H), 7.61 (dt, *J* = 8.1, 1.1 Hz, 1H), 7.56 – 7.51 (m, 3H), 7.48 (td, *J* = 8.2, 5.7 Hz, 1H), 7.31 (ddd, *J* = 9.4, 8.3, 1.2 Hz, 1H), 5.87 (ddt, *J* = 17.2, 10.2, 6.0 Hz, 1H), 5.19 – 5.02 (m, 1H), 3.88 (d, *J* = 1.9 Hz, 2H), 3.27 (dtd, *J* = 6.0, 1.5, 0.7 Hz, 2H), 1.93 (s, 1H). **^{13}C NMR (101 MHz, CDCl$_3$)** δ 165.3 (C), 161.9 (d, *J* = 248 Hz, ArCF), 137.5 (d, *J* = 6 Hz, C), 136.5 (CH), 130.8 (ArCH), 129.2 (d, *J* = 10 Hz, ArCH), 129.1 (2 ArCH), 127.0 (2 ArCH), 126.8 (C), 123.1 (d, *J* = 20 Hz, C), 121.0 (d, *J* = 3 Hz, ArCH), 117.5 (d, *J* = 23 Hz, ArCH), 116.1 (CH_2), 51.8 (CH_2), 42.2 (d, *J* = 4 Hz, CH_2). **^{19}F NMR (376 MHz, CDCl$_3$)** δ -113.46 (m). **IR (neat, oil)** 3675 (w), 3326 (w), 3074 (w), 2912 (w), 1643 (w), 1587 (m), 1530 (m), 1469 (s), 1449 (s), 1364 (m), 1248 (m), 1072 (s), 1020 (m), 995 (m), 919 (s), 864 (m), 787 (s), 729 (s), 690 (s), 559 (w), 493 (w). **HRMS** (QTOF) m/z: $[M+H]^+$ Calcd for $C_{17}H_{17}FN_5^+$ 310.1463; Found 310.1460.

2-(2-((Cinnamyloxy)methyl)-3-fluorophenyl)-5-phenyl-2*H*-tetrazole (**3h**)

Following the procedure outlined in **GP3**. Cinnamyl alcohol (161 mg, 1.2 mmol) and **2a** (333 mg, 1.0 mmol) was used to obtain **3h** as a colourless oil in 56% (216 mg, 0.56 mmol) following column chromatography (10% EtOAc: pentane).

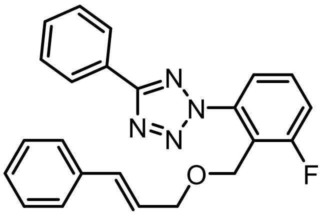

Chemical Formula: $C_{23}H_{19}FN_4O$
Exact Mass: 386.1543

Appearance: Colourless Oil

Yield: 56%, 216 mg (0.56 mmol)

^{1}H NMR (400 MHz, $CDCl_3$) δ 8.29 – 8.19 (m, 2H), 7.62 – 7.57 (m, 1H), 7.55 – 7.47 (m, 4H), 7.32 (td, *J* = 8.8, 1.3 Hz, 1H), 7.25 – 7.14 (m, 5H), 6.41 (dt, *J* = 15.9, 1.5 Hz, 1H), 6.07 (dt, *J* = 16.0, 6.2 Hz, 1H), 4.85 (d, *J* = 1.8 Hz, 2H), 4.05 (dd, *J* = 6.2, 1.4 Hz, 2H). **^{13}C NMR (101 MHz, $CDCl_3$)** δ 165.3 (C), 161.7 (d, *J* = 250 Hz, ArCF), 137.8 (d, *J* = 6 Hz, C), 136.5 (C), 133.0 (CH), 130.7 (ArCH), 130.3 (d, *J* = 10 Hz, ArCH), 129.1 (2ArCH), 128.5 (2ArCH), 127.7 (ArCH), 127.2 (2 ArCH), 127.1 (C), 126.6 (2 ArCH), 125.4 (CH), 121.6 (d, *J* = 4 Hz, ArCH), 121.4 (d, *J* = 18 Hz, C), 117.8 (d, *J* = 23 Hz, ArCH), 71.5 (CH_2), 61.2 (d, *J* = 5 Hz, CH_2). **^{19}F NMR (376 MHz, $CDCl_3$)** δ -113.42 (m). **IR (neat, oil)** 3059 (w), 2850 (w), 1589 (m), 1470 (s), 1448 (s), 1361 (m), 1246 (s), 1072 (s), 966 (s), 870 (m), 789 (s), 729 (s), 690 (s). **HRMS** (QTOF) m/z: $[M+H]^+$ Calcd for $C_{23}H_{20}FN_4O^+$ 387.1616; Found 387.1616.

N-(2-Fluoro-6-(5-phenyl-2*H*-tetrazol-2-yl)benzyl)-2-methylpropan-1-amine (**3i**)

Following the procedure outlined in **GP4**. Isobutyl amine (131 mg, 1.8 mmol) and **2a** (502 mg, 1.5 mmol) was used to obtain **3i** following organic extraction as a pale-yellow oil in 93% (432 mg, 1.4 mmol).

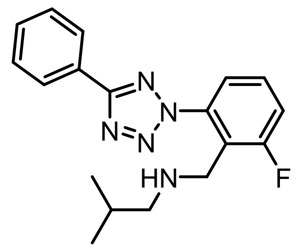

Chemical Formula: $C_{18}H_{20}FN_5$
Exact Mass: 325.1703

Appearance: pale-yellow oil

Yield: 93%, 432 mg (1.4 mmol)

^{1}H NMR (400 MHz, $CDCl_3$) δ 8.28 – 8.18 (m, 2H), 7.59 (dt, *J* = 8.0, 1.1 Hz, 1H), 7.56 – 7.50 (m, 3H), 7.47 (td, *J* = 8.2, 5.7 Hz, 1H), 7.30 (ddd, *J* = 9.4, 8.3, 1.2 Hz, 1H), 3.88 (d, *J* = 1.9 Hz, 2H), 2.41 (dd, *J* = 6.8, 0.8 Hz, 2H), 1.94 (s, 1H),f 1.70 (dh, *J* = 13.4, 6.7 Hz, 1H), 0.86 (d, *J* = 6.6 Hz, 6H). **^{13}C NMR (101 MHz, $CDCl_3$)** δ 165.3 (C), 161.8 (d, *J* = 248 Hz, ArCF), 137.5 (d, *J* = 6 Hz, C), 130.8 (ArCH), 129.1 (d, *J* = 10 Hz, ArCH), 129.0 (2 ArCH), 127.1 (2 ArCH), 126.8 (C), 123.2 (d, *J* = 18 Hz, C), 121.1 (d, *J* = 4 Hz, ArCH), 117.5 (d, *J* = 24 Hz, ArCH), 57.2 (CH_2), 42.9 (d, *J* = 4 Hz, CH_2), 28.3 (CH), 20.6 (2 CH_3). **^{19}F NMR (376 MHz, $CDCl_3$)** δ -113.6 (m). **IR (neat, oil)** 2955 (m, br), 2870 (w), 1615 (w), 1587 (w), 1529 (m), 1469 (s), 1364 (m), 1248 (m), 1109 (m), 866 (s), 788 (s), 730 (s), 691 (s). **HRMS** (QTOF) m/z: $[M+H]^+$ Calcd for $C_{18}H_{21}FN_5^+$ 326.1776; Found 326.1773.

Methyl (2-fluoro-6-(5-phenyl-2*H*-tetrazol-2-yl)benzyl)glycinate (**3j**)

Following the procedure outlined in **GP4**. Methyl ester glycine hydrochloride (151 mg, 1.2 mmol) and **2a** (334 mg, 1 mmol) was used to obtain **3j** as a brown solid in 46% (158 mg, 0.46 mmol) following column chromatography (10% EtOAc: pentane).

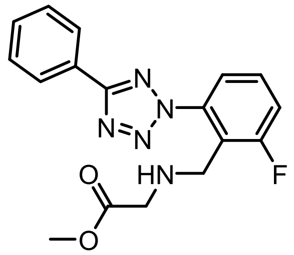

Chemical Formula: $C_{17}H_{16}FN_5O_2$
Exact Mass: 341.1288

Appearance: brown solid

Yield: 46%, 158 mg (0.46 mmol)

^{1}H NMR (400 MHz, CDCl$_3$) δ 8.30 – 8.20 (m, 2H), 7.64 (dt, *J* = 8.1, 1.2 Hz, 1H), 7.59 – 7.44 (m, 4H), 7.35 – 7.28 (m, 1H), 3.97 (d, *J* = 2.0 Hz, 2H), 3.65 (s, 3H), 3.46 (d, *J* = 0.8 Hz, 2H).**^{13}C NMR (101 MHz, CDCl$_3$)** δ 172.4 (C=O), 165.3 (C), 161.9 (d, *J* = 248 Hz, ArCF), 137.4 (C), 130.8 (ArCH), 129.5 (d, *J* = 10 Hz, ArCH), 129.0 (2 ArCH), 128.5 (C), 127.1 (2 ArCH), 126.8 (C), 122.3 (d, *J* = 20 Hz, C), 121.0 (d, *J* = 4 Hz, ArCH), 117.5 (d, *J* = 24 Hz, ArCH), 51.8 (CH_3), 50.0 (CH_2), 42.2 (d, *J* = 4 Hz, CH_2). **^{19}F NMR (376 MHz, CDCl$_3$)** δ -113.51(m). **IR (solid)** 3358 (m), 2958 (m), 1734 (s), 1610 (m), 1583 (m), 1461 (s), 1437 (s), 1373 (m), 1212 (s), 1180 (s), 1141 (m), 1071 (m), 1021 (m), 992 (m), 936 (s), 850 (m). 825(m), 785 (s), 726 (s), 688 (s), 593 (m), 496 (m). **HRMS** (QTOF) m/z: $[M+H]^+$ Calcd for $C_{17}H_{17}FN_5O_2^+$ 342.1361; Found 342.1362.

N-(2-Fluoro-6-(5-phenyl-2*H*-tetrazol-2-yl)benzyl)cyclopentanamine (**3k**)

Following the procedure outlined in **GP4**. Cyclopentylamine (102 mg, 1.2 mmol) and **2a** (333 mg, 1.0 mmol) was used to obtain **3k** following organic extraction as an orange oil in 98% (334 mg, 0.98 mmol)

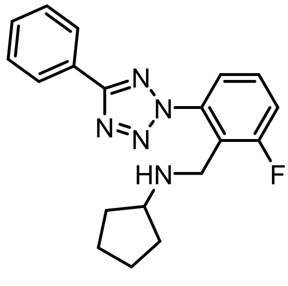

Chemical Formula: $C_{19}H_{20}FN_5$
Exact Mass: 337.1703

Appearance: orange oil

Yield: 98%, 334 mg (0.98 mmol)

^{1}H NMR (400 MHz, CDCl$_3$) δ 8.28 – 8.19 (m, 2H), 7.60 – 7.50 (m, 4H), 7.46 (td, *J* = 8.2, 5.7 Hz, 1H), 7.30 (ddd, *J* = 9.5, 8.3, 1.2 Hz, 1H), 3.84 (d, *J* = 1.8 Hz, 2H), 3.07 (p, *J* = 6.4 Hz, 1H), 1.85 – 1.29 (m, 9H). **^{13}C NMR (101 MHz, CDCl$_3$)** δ 165.3 (C), 161.9 (d, *J* = 248 Hz, ArCF), 137.5 (d, *J* = 6 Hz, C), 130.8 (ArCH),129.0 (2 ArCH), 129.0 (d, *J* = 10 Hz, ArCH), 127.0 (2 ArCH), 126.8 (C), 123.7 (d, *J* = 20 Hz, C), 121.0 (d, *J* = 4 Hz, ArCH), 117.6 (d, *J* = 23 Hz, ArCH), 59.2 (CH), 41.6 (d, *J* = 4 Hz, CH_2), 33.1 (2 CH_2), 24.1 (2 CH_2). **^{19}F NMR (376 MHz, CDCl$_3$)** δ -113.73 (m). **IR (neat, oil)** 2951 (m, br), 2865 (m), 1615 (m), 1588 (m), 1530 (m), 1469 (s), 1449 (s), 1362 (m), 1242 (m), 1122 (w), 1072 (m). 1021 (m), 866 (m), 788 (s), 729 (s), 690 (s). **HRMS** (QTOF) m/z: $[M+H]^+$ Calcd for $C_{19}H_{21}FN_5^+$ 338.1776; Found 338.1776.

N-(2-Fluoro-6-(5-phenyl-2*H*-tetrazol-2-yl)benzyl)-4-(trifluoromethoxy)aniline (**3l**)

Following the procedure outlined in **GP4**. 4-(trifluoromethoxy)-aniline (159 mg, 0.9 mmol) and **2a** (252 mg, 0.75 mmol) was used to obtain **3l** as a crystalline solid in 85% (275 mg, 0.64 mmol) following column chromatography (10% EtOAc: pentane).

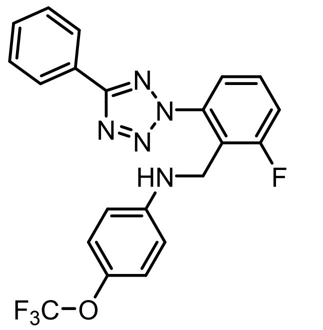

Chemical Formula: $C_{21}H_{15}F_4N_5O$
Exact Mass: 429.1213

Appearance: crystalline solid

Yield: 85%, 275 mg (0.64 mmol)

^{1}H NMR (400 MHz, $CDCl_3$) δ 8.23 – 8.13 (m, 2H), 7.69 (dt, *J* = 8.1, 1.2 Hz, 1H), 7.57 – 7.44 (m, 4H), 7.33 (ddd, *J* = 9.4, 8.3, 1.2 Hz, 1H), 7.04 – 6.95 (m, 2H), 6.66 – 6.56 (m, 2H), 4.53 (d, *J* = 4.0 Hz, 2H), 4.43 (s, 1H). **^{13}C NMR (101 MHz, $CDCl_3$)** δ 165.5 (C), 161.6 (d, *J* = 248 Hz, ArCF), 146.2 (C), 141.0 (q, *J* = 2 Hz, C), 137.3 (d, *J* = 6 Hz, C), 130.9 (ArCH), 129.8 (d, *J* = 10 Hz, ArCH), 129.1 (2 ArCH), 127.0 (ArCH), 126.5 (C), 122.3 (2 ArCH), 121.4 (d, *J* = 19 Hz, C), 121.0 (d, *J* = 4 Hz, C), 120.7 (q, *J* = 256 Hz, CF_3), 117.7 (d, *J* = 24 Hz, ArCH), 113.8 (d, *J* = 1 Hz, 2 ArCH), 38.3 (d, *J* = 5 Hz, CH_2). **^{19}F NMR (376 MHz, $CDCl_3$)** δ -58.44, -112.84 – -112.92 (m). **IR (solid)** 3359 (m), 3044 (w, br), 2883 (w), 1590 (m), 1510 (s), 1473 (s), 1451 (m), 1226 (s, br), 1138 (s), 1075 (m), 987 (m), 865 (m), 834 (m), 783 (s), 729 (m), 691 (m), 569 (m), 537 (m), 485 (m), 431 (w). **HRMS** (QTOF) m/z: $[M+H]^+$ Calcd for $C_{21}H_{16}F_4N_5O^+$ 430.1285; Found 430.1288.

N-(2-Fluoro-6-(5-phenyl-2*H*-tetrazol-2-yl)benzyl)aniline (**3m**)

Following the procedure outlined in **GP4**. Aniline (110 mg, 1.16 mmol) and **2a** (354 mg, 1.06 mmol) was used to obtain **3m** as an orange solid in 86% (316 mg, 0.91 mmol) following column chromatography (5% EtOAc: pentane)

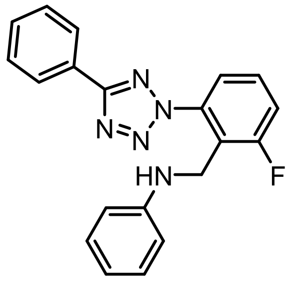

Chemical Formula: $C_{20}H_{16}FN_5$
Exact Mass: 345.1390

Appearance: Orange solid

Yield: 86%, 316 mg (0.91 mmol)

^{1}H NMR (400 MHz, $CDCl_3$) δ 8.24 – 8.13 (m, 2H), 7.66 (dt, *J* = 8.1, 1.1 Hz, 1H), 7.58 – 7.44 (m, 4H), 7.32 (ddd, *J* = 9.5, 8.3, 1.2 Hz, 1H), 7.19 – 7.09 (m, 2H), 6.71 (tt, *J* = 7.3, 1.1 Hz, 1H), 6.68 – 6.63 (m, 2H), 4.55 (s, 2H), 4.33 (s, 1H). **^{13}C NMR (101 MHz, $CDCl_3$)** δ 165.4 (C), 161.7 (d, *J* = 249 Hz, ArCF), 147.4 (C), 137.4 (d, *J* = 6 Hz, C), 130.8 (ArCH), 129.6 (d, *J* = 10 Hz, ArCH), 129.1 (2 ArCH), 127.1 (2 ArCH), 126.6 (2 ArCH), 122.1 (C), 122.0 (C), 121.0 (d, *J* = 4 Hz, ArCH), 118.3 (ArCH), 117.7 (d, *J* = 24 Hz, ArCH), 113.6 (2 ArCH), 38.2 (d, *J* = 4 Hz, CH_2). **^{19}F NMR (376 MHz, $CDCl_3$)** δ -112.86 (m). **IR (solid)** 3432 (m), 3077 (w), 3046 (w), 2958 (w), 1601 (m), 1506 (m), 1461 (s), 1450 (s), 1337 (m), 1286 (m), 1236 (m), 1216 (m), 1182 (m), 1160 (m), 1122 (w), 1065 (w), 1020 (w), 992 (m), 967 (m), 900 (s), 860 (m), 817 (m), 782 (s), 745 (s), 717 (s), 690 (s), 633 (w), 503 (s), 444 (m). **HRMS** (QTOF) m/z: $[M+H]^+$ Calcd for $C_{20}H_{17}FN_5^+$ 346.1463; Found 346.1462.

N-(2-Fluoro-6-(5-phenyl-2*H*-tetrazol-2-yl)benzyl)-1-(4-methoxyphenyl)methanamine (**3n**)

Following the procedure outlined in **GP4**. 4-methoxybenzylamine (123 mg, 0.12 mmol) and **2a** (250 mg, 0.75 mmol) was used to obtain **3n** as a pale-yellow oil in 93% (273 mg, 0.70 mmol) following column chromatography (20% EtOAc: pentane).

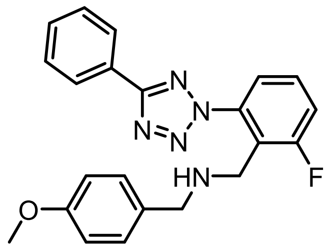

Chemical Formula: $C_{22}H_{20}FN_5O$
Exact Mass: 389.1652

Appearance: pale-yellow oil

Yield: 93%, 273 mg (0.70 mmol)

^{1}H NMR (400 MHz, CDCl$_3$) δ 8.25 – 8.16 (m, 2H), 7.58 (dt, *J* = 8.1, 1.1 Hz, 1H), 7.55 – 7.50 (m, 3H), 7.46 (td, *J* = 8.2, 5.7 Hz, 1H), 7.30 (td, *J* = 8.7, 1.2 Hz, 1H), 7.21 – 7.13 (m, 2H), 6.79 – 6.71 (m, 2H), 3.89 (d, *J* = 1.9 Hz, 2H), 3.73 (s, 2H), 3.72 (d, *J* = 0.6 Hz, 3H), 2.10 (s, 1H). **^{13}C NMR (101 MHz, CDCl$_3$)** δ 165.2 (C), 161.9 (d, *J* = 248 Hz, ArCH), 158.6 (C), 137.5 (d, *J* = 6 Hz, C), 131.9 (C), 130.8 (ArCH), 129.2 (2 ArCH), 129.2 (d, *J* = 10 Hz, ArCH), 129.0 (2 ArCH), 127.1 (2 ArCH), 126.8 (C), 123.4 (d, *J* = 20 Hz, C), 121.1 (d, *J* = 4 Hz, ArCH), 117.6 (d, *J* = 23 Hz, C), 113.7 (2 ArCH), 55.2 (CH_3), 52.7 (CH_2), 42.2 (d, *J* = 4 Hz, CH_2). **^{19}F NMR (376 MHz, CDCl$_3$)** δ -113.50 (m). **IR (neat, oil)** 3322 (w), 2932 (w), 2834 (w), 1612 (m), 1586 (m), 1510 (s), 1464 (s), 1363 (w), 1300 (w), 1245 (s), 1174 (m), 1123 (m), 1101 (m), 1021 (s), 926 (m), 868 (m), 788 (s), 730 (s), 691 (s), 575 (m), 514 (m). **HRMS** (QTOF) m/z: $[M+H]^+$ Calcd for $C_{22}H_{21}FN_5O^+$ 390.1725; Found 390.1724.

N-ethyl-*N*-(2-fluoro-6-(5-phenyl-2*H*-tetrazol-2-yl)benzyl)ethanamine (**3o**)

Following the procedure outlined in **GP4**. Diethylamine (88mg, 1.2 mmol) and **2a** (360 mg, 1.08 mmol) was used to obtain **3o** following organic extractions as a yellow oil in 92% (324 mg, 1.00 mmol).

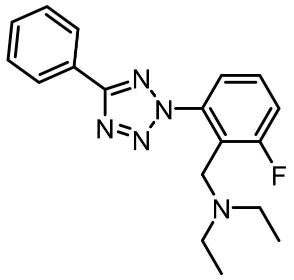

Chemical Formula: $C_{18}H_{20}FN_5$
Exact Mass: 325.1703

Appearance: Yellow oil

Yield: 92%, 324 mg (1.00 mmol)

^{1}H NMR (400 MHz, CDCl$_3$) δ 8.29 – 8.18 (m, 2H), 7.56 – 7.49 (m, 3H), 7.46 – 7.42 (m, 2H), 7.31 – 7.27 (m, 1H), 3.88 (d, J = 1.7 Hz, 2H), 2.25 (q, J = 7.1 Hz, 4H), 0.72 (t, J = 7.1 Hz, 6H). **^{13}C NMR (101 MHz, CDCl$_3$)** δ 164.8, 161.7 (d, J = 248 Hz), 138.2, 130.4, 129.0, 128.7 (d, J = 10 Hz), 127.3, 127.0, 124.0 (d, J = 17 Hz), 122.1 (d, J = 4 Hz), 117.6 (d, J = 24 Hz), 46.2, 46.1 (d, J = 3 Hz), 11.19.**^{19}F NMR (376 MHz, CDCl$_3$)** δ -113.75 – -113.86 (m). **IR (oil)** 2970 (w), 2965 (w), 2805 (w), 1616 (w), 1588 (w), 1469 (s), 1364 (w), 1244 (m), 1200 (w), 1023(w), 921 (w), 867 (m), 788 (m), 772 (m), 730 (s), 691 (s), 502 (w). **HRMS** (QTOF) m/z: $[M+H]^+$ Calcd for $C_{18}H_{21}FN_5^+$; 326.1776 Found 326.1775.

General Procedure for Photo-click reaction (**GP5**)

A solution of substrate (**3a-m**) in EtOAc (0.01 M) was pumped at a flow rate of 5 mL min^{-1} through the Vapourtec E-Series UV150 reactor (10 mL volume, 2-minute residence time) equipped with a medium pressure Hg lamp (137 W input, 42 W output, low pass filter) light under a 2-bar back pressure. The collected reaction mixture was concentrated *in vacuo* and the crude material was purified by column chromatography to yield the product.

Characterisation data of compound **4a-h**

7-Fluoro-2-phenyl-4*H*,6*H*-benzo[e]pyrazolo[5,1-c][1,4]oxazepine (**4a**)

Following the procedure outlined in **GP5**. **3a** (1.02 g, 3.31 mmol) was used to obtain **4a** as an orange solid in 60% (0.56 g, 2 mmol) following column chromatography (5% EtOAc : pentane).

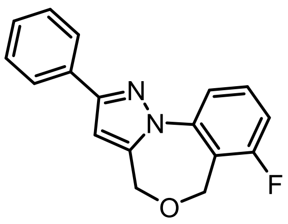

Chemical Formula: $C_{17}H_{13}FN_2O$
Exact Mass: 280.1012

Appearance: orange solid

Yield: 60%, 0.56 g (2 mmol)

^{1}H NMR (400 MHz, $CDCl_3$) δ 7.94 – 7.85 (m, 2H), 7.76 (dt, *J* = 8.1, 1.0 Hz, 1H), 7.50 (td, *J* = 8.2, 5.9 Hz, 1H), 7.47 – 7.41 (m, 3H), 7.41 – 7.32 (m, 1H), 7.11 (ddd, *J* = 9.4, 8.3, 1.1 Hz, 1H), 4.70 (d, *J* = 1.8 Hz, 2H), 4.66 (s, 2H). **^{13}C NMR (101 MHz, $CDCl_3$)** δ 160.6 (d, *J* = 248 Hz, ArCF), 153.1 (C), 141.8 (d, *J* = 6 Hz, C), 141.2 (C), 132.6 (C), 130.8 (d, *J* = 10 Hz, ArCH), 128.8 (2 ArCH), 128.4 (ArCH), 125.9 (2 ArCH), 117.7 (d, *J* = 4 Hz, ArCH), 116.7 (d, *J* = 20 Hz, C), 113.9 (d, *J* = 23 Hz, ArCH), 104.6 (CH), 60.0 (CH_2), 59.9 (d, *J* = 5 Hz, CH_2). **^{19}F NMR (376 MHz, $CDCl_3$)** δ -116.79 (m). **IR (solid)** 3068 (w), 2981 (w), 1614 (m), 1588 (m), 1527 (w), 1470 (s), 1447 (s), 1251 (m), 1226 (m), 1185 (m), 1068 (m), 1019 (s), 909 (s), 837 (w), 795 (s), 725 (s), 684 (s), 614 (m), 588 (m), 493 (m). **HRMS** (QTOF) m/z: $[M+H]^+$ Calcd for $C_{17}H_{14}FN_2O^+$ 281.1085; Found 281.1084.

10-Methyl-2-phenyl-4H,6*H*-benzo[e]pyrazolo[5,1-c][1,4]oxazepine (**4b**)

Following the procedure outlined in **GP5**. **3b** (103 mg, 0.338 mmol) was used to obtain **4b** with a qNMR yield of 37% as **4b** and **3b** were inseparable *via* column chromatography.

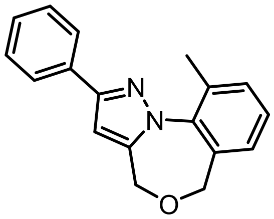

Chemical Formula: $C_{18}H_{16}N_2O$
Exact Mass: 276.1263

Appearance: Colourless oil

Yield: N/a

^{1}H NMR (400 MHz, $CDCl_3$) δ 7.95 – 7.88 (m, 2H), 7.47 – 7.38 (m, 3H), 7.38 – 7.26 (m, 3H), 6.75 (s, 1H), 4.54 (s, 2H), 4.40 (s, 2H), 2.63 (s, 3H).

9-Fluoro-2-phenyl-4*H*,6*H*-benzo[e]pyrazolo[5,1-c][1,4]oxazepine (**4c**)

Following the procedure outlined in **GP5**. **3c** (101 mg, 0.32 mmol) was used to obtain **4c** as a yellow crystalline solid in 66% (60 mg, 0.214 mmol) following column chromatography (5% EtOAc : pentane).

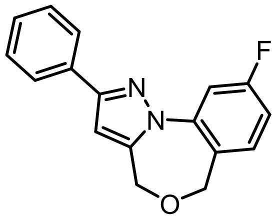

Chemical Formula: $C_{17}H_{13}FN_2O$
Exact Mass: 280.1012

Appearance: yellow crystalline solid

Yield: 66%, 60 mg (0.214 mmol)

^{1}H NMR (400 MHz, $CDCl_3$) δ 7.94 – 7.86 (m, 2H), 7.69 (dd, *J* = 9.4, 2.6 Hz, 1H), 7.48 – 7.32 (m, 4H), 7.06 (td, *J* = 8.3, 2.6 Hz, 1H), 6.75 (d, *J* = 0.6 Hz, 1H), 4.66 (d, *J* = 0.5 Hz, 2H), 4.56 (s, 2H). **^{13}C NMR (101 MHz, $CDCl_3$)** δ 163.5 (d, *J* = 249 Hz CF), 153.2 (C), 141.7 (d, *J* = 11 Hz, C), 141.3 (C), 132.6 (C), 131.9 (d, *J* = 10 Hz ArCH), 128.8 (ArCH), 128.4 (2 ArCH), 125.9 (2 ArCH), 124.7 (d, *J* = 3 Hz,

C), 114.1 (d, J = 22 Hz, ArCH), 109.5 (d, J = 26 Hz, ArCH), 104.5(CH), 67.5 (CH_2), 59.9 (CH_2). **^{19}F NMR (376 MHz, $CDCl_3$)** δ -110.17 (m). **IR (neat, solid)** 2922 (w), 1721 (w), 1603 (m), 1552 (m), 1496 (s), 1457 (s), 1353 (m), 1270 (m), 1215 (m), 1152 (m), 1075 (s), 950 (m), 890 (s), 845 (s), 811 (s), 762 (s), 688 (s), 575 (m). **HRMS** (QTOF) m/z: $[M+H]^+$ Calcd for $C_{17}H_{14}FN_2O^+$ 281.1085; Found 281.1088.

7-Fluoro-2,3-diphenyl-4*H*,6*H*-benzo[e]pyrazolo[5,1-c][1,4]oxazepine (**4d**)

Following the procedure outlined in **GP5** with a deviation of using an input lamp power of 124 W. **3d** (288 mg, 0.75 mmol) was used to obtain **4d** as a yellow waxy solid in 8% (20 mg, 0.06 mmol) following column chromatography (1% EtOAc: pentane).

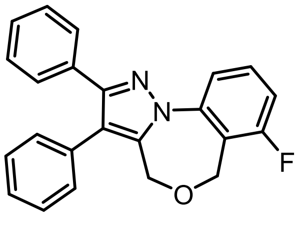

Chemical Formula: $C_{23}H_{17}FN_2O$
Exact Mass: 356.1325

Appearance: yellow waxy solid.

Yield: 8%, 20 mg (0.06 mmol).

^{1}H NMR (400 MHz, $CDCl_3$) δ 7.81 (dt, J = 8.2, 1.0 Hz, 1H), 7.58 – 7.50 (m, 3H), 7.44 – 7.28 (m, 8H), 7.14 (td, J = 8.7, 1.1 Hz, 1H), 4.79 (d, J = 1.8 Hz, 2H), 4.55 (s, 2H). **^{13}C NMR (101 MHz, $CDCl_3$)** δ 160.6 (d, J = 249 Hz, ArCF), 151.1 (C), 141.8 (d, J = 5.5, C), 138.5 (C), 132.5 (C), 132.0 (C), 130.9 (d, J = 10 Hz, ArCH), 130.1 (2 ArCH), 128.7 (2 ArCH), 128.3 (2 ArCH), 128.3 (2 ArCH), 128.0 (ArCH), 127.4 (ArCH), 121.2 (C), 117.7 (d, J = 4 Hz, ArCH), 116.5 (C), 114.1 (d, J = 23 Hz, ArCH), 60.0 (d, J = 5 Hz, CH_2), 58.9 (CH_2). **^{19}F NMR (376 MHz, $CDCl_3$)** δ -116.57 (m). **IR (solid)** 2921 (w), 1771 (w), 1700 (w), 1617 (w), 1588 (m), 1482 (s), 1450 (m), 1433 (m), 1376 (w), 1233 (s), 1178 (m), 1121 (m), 1076 (s), 910 (s), 787 (s), 732 (s), 696 (s), 563 (m). **HRMS** (QTOF) m/z: $[M+H]^+$ Calcd for $C_{23}H_{18}FN_2O^+$ 357.1398; Found 357.1398.

8-Fluoro-2-phenyl-3,3a,4,5-tetrahydro-7*H*-benzo[c]pyrazolo[1,5-e][1,5]oxazocine (**4e**)

Following the procedure outlined in **GP5**. **3e** (167 mg, 0.515 mmol) was used to obtain **4e** as an orange solid in 30% (45 mg, 0.152 mmol) following column chromatography (5% EtOAc: pentane).

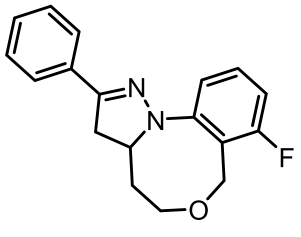

Chemical Formula: $C_{18}H_{17}FN_2O$
Exact Mass: 296.1325

Appearance: Orange solid

Yield: 30%, 45 mg (0.152 mmol)

^{1}H NMR (400 MHz, $CDCl_3$) δ 7.79 – 7.68 (m, 2H), 7.44 – 7.30 (m, 4H), 7.21 (dt, J = 8.2, 1.0 Hz, 1H), 6.95 (ddt, J = 9.0, 7.9, 1.1 Hz, 1H), 4.99 (dd, J = 11.9, 1.3 Hz, 1H), 4.86 (dd, J = 11.9, 2.9 Hz, 1H), 4.01 – 3.91 (m, 1H), 3.66 – 3.53 (m, 1H), 3.50 – 3.35 (m, 2H), 2.93 (dd, J = 16.0, 14.1 Hz, 1H), 2.35 – 2.23 (m, 2H). **^{13}C NMR (101 MHz, $CDCl_3$)** δ 162.2 (d, J = 248 Hz, ArCF), 152.7 (C), 150.9 (d, J = 7 Hz, C), 132.5 (C), 130.6 (d, J = 10 Hz, ArCH), 129.2 (ArCH), 128.6 (2 ArCH), 125.9 (2 ArCH), 117.7 (d, J = 3 Hz, ArCH), 116.8 (d, J = 18 Hz ArC), 112.1 (d, J = 22 Hz, ArCH), 71.1 (CH), 64.1 (CH_2), 58.3 (d, J = 5 Hz, CH_2), 41.0 (CH_2), 33.9 (CH_2). **^{19}F NMR (376 MHz, $CDCl_3$)** δ -116.37 (m). **IR (solid)** 2934 (w, br), 1613 (m), 1581 (s), 1465 (s), 1376 (m), 1279 (s), 1109 (s), 1050 (s), 991 (m), 912 (w), 869 (m), 853 (m), 786 (m), 759 (s), 736 (m), 690 (s), 665 (s), 637 (m), 554 (m), 480 (m). **HRMS** (QTOF) m/z: $[M+H]^+$ Calcd for $C_{18}H_{18}FN_2O^+$ 297.1398; Found 297.1400.

7-Fluoro-2-phenyl-3a,4-dihydro-3*H*,6*H*-benzo[e]pyrazolo[5,1-c][1,4]oxazepine (**4f**)

Following the procedure outlined in **GP5**. **3f** (101 mg, 0.326 mmol) was used to obtain **4f** as a yellow crystalline solid in 65% (60 mg, 0.212 mmol) following column chromatography (5% EtOAc: pentane).

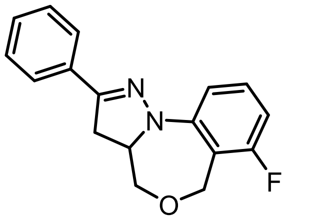

Chemical Formula: $C_{17}H_{15}FN_2O$
Exact Mass: 282.1168

Appearance: yellow crystalline solid

Yield: 65%, 60 mg (0.21 mmol)

^{1}H NMR (400 MHz, $CDCl_3$) δ 7.76 – 7.73 (m, 2H), 7.45 – 7.36 (m, 4H), 7.22 (td, J = 8.2, 6.2 Hz, 1H), 6.76 (ddd, J = 9.4, 8.2, 1.1 Hz, 1H), 5.36 (d, J = 13.5 Hz, 1H), 4.36 – 4.27 (m, 2H), 3.91 – 3.82 (m, 1H), 3.77 (dd, J = 11.7, 9.8 Hz, 1H), 3.40 (dfd, J = 16.3, 10.0 Hz, 1H), 2.84 (dd, J = 16.3, 13.3 Hz, 1H).**^{13}C NMR (101 MHz, $CDCl_3$)** δ 160.5 (d, *J* = 245 Hz, ArCF), 151.2 (C), 150.3 (d, *J* = 6 Hz, ArCH), 132.2 (C), 129.4 (ArCH), 129.3 (d, *J* = 11 Hz, ArCH), 128.6 (2 ArCH), 126.1 (2 ArCH), 116.3 (d, *J* = 17 Hz, C), 113.8 (d, *J* = 3 Hz, ArCH), 109.4 (d, *J* = 24 Hz, ArCH), 74.3 (CH_2), 67.6 (CH), 65.0 (d, *J* = 8 Hz, CH_2), 36.4 (CH_2). **^{19}F NMR (376 MHz, $CDCl_3$)** δ -119.67 (m). **IR (solid)** 2958 (w), 2857(w), 1725 (w, br), 1613 (s), 1579 (s), 1469 (s), 1371 (m), 1293 (m), 1236 (m), 1176 (m), 1093 (s), 1072 (s), 1028 (m), 922 (s), 831 (m), 778 (m), 759 (s), 716 (m), 691 (s), 599 (m), 561 (m), 541 (s), 502 (m), 422 (m). **HRMS** (QTOF) m/z: $[M+H]^+$ Calcd for $C_{17}H_{15}FN_2O^+$ 283.1241; Found 283.1242 .

7-Fluoro-2-phenyl-3a,4,5,6-tetrahydro-3*H*-benzo[f]pyrazolo[1,5-a][1,4]diazepine (**4g**)

Following the procedure outlined in **GP5**. **3g** (93 mg, 0.30 mmol) was used to obtain **4g** as a in 15% (14 mg, 0.05 mmol) following column chromatography (5-40% EtOAc: pentane).

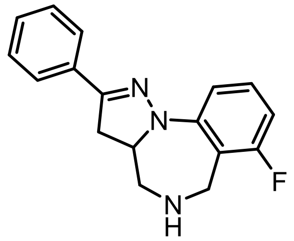

Chemical Formula: $C_{17}H_{16}FN_3$
Exact Mass: 281.1328

Appearance: yellow solid

Yield: 15%, 14 mg (0.05 mmol)

^{1}H NMR (400 MHz, $CDCl_3$) δ 7.74 (ddt, *J* = 6.4, 2.3, 1.0 Hz, 2H), 7.44 – 7.33 (m, 4H), 7.21 – 7.11 (m, 1H), 6.75 (dd, *J* = 9.5, 8.2 Hz, 1H), 4.58 (d, *J* = 14.7 Hz, 1H), 3.68 (dtd, *J* = 12.8, 10.1, 2.5 Hz, 1H), 3.57 (ddd, *J* = 13.4, 6.4, 2.3 Hz, 2H), 3.50 – 3.39 (m, 1H), 3.11 (dd, *J* = 13.3, 10.1 Hz, 1H), 2.88 (dd, *J* = 16.4, 13.0 Hz, 1H). **^{13}C NMR (101 MHz, $CDCl_3$)** δ 161.6 (d, *J* = 246 Hz, ArCF), 151.2 (C), 150.5 (d, *J* = 6 Hz, C), 132.4 (C), 129.2 (ArCH), 128.6 (2 ArCH), 128.3 (d, *J* = 10 Hz, ArCH), 126.0 (2 ArCH),125.7 (d, *J* = 20 Hz, C), 114.3 (d, *J* = 3 Hz, ArCH), 109.5 (d, *J* = 24 Hz, ArCH), 69.0 (CH), 55.0 (CH_2), 43.8 (d, *J* = 8 Hz, CH_2), 38.3 (CH_2). **^{19}F NMR (376 MHz, $CDCl_3$)** δ -119.59 – -119.69 (m). **IR (solid)** 3321 (w), 2970 (w). 2915 (w), 2489 (w), 1606 (m), 1572 (m), 1446 (s. br), 1367 (s), 1321 (w), 1283.6 (m), 1227 (m), 1172 (m), 1082 (s), 1062 (s, br), 966 (m), 908 (w), 840 (m), 826 (m), 787 (s), 757 (s), 688 (s), 665 (s), 541 (m), 504 (w). **HRMS** (QTOF) m/z: $[M+H]^+$ Calcd for $C_{17}H_{17}FN_3$ 282.1401; Found 282.1403.

7-Fluoro-2,3-diphenyl-3a,4-dihydro-3*H*,6*H*-benzo[e]pyrazolo[5,1-c][1,4]oxazepine (**4h**)

Following the procedure outlined in **GP5**. **3h** (91 mg, 0.235 mmol) was used to obtain **4h** as a yellow waxy solid in 15% (13 mg, 0.035 mmol) following column chromatography (5% EtOAc: pentane)

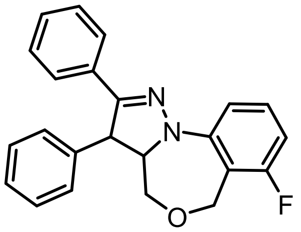

Chemical Formula: $C_{23}H_{19}FN_2O$
Exact Mass: 358.1481

Appearance: Yellow waxy solid

Yield: 15%, 13 mg (0.035 mmol)

^{1}H NMR (400 MHz, CDCl$_3$) δ 7.58 – 7.52 (m, 3H), 7.33 – 7.28 (m, 3H), 7.28 – 7.19 (m, 7H), 6.78 (ddd, *J* = 9.5, 8.1, 1.1 Hz, 1H), 5.34 (d, *J* = 13.5 Hz, 1H), 4.36 – 4.22 (m, 3H), 3.89 (dd, *J* = 11.8, 9.7 Hz, 1H), 3.73 (ddd, *J* = 12.5, 9.7, 2.6 Hz, 1H). **^{13}C NMR (101 MHz, CDCl$_3$)** δ 160.5 (d, *J* = 245 Hz, ArCF), 151.5 (C), 149.7 (d, *J* = 6 Hz, C), 139.5 (C), 131.5 (C), 129.4 (d, *J* = 10 Hz, ArCH), 129.2 (2 ArCH), 128.8 (ArCH), 128.4 (2 ArCH), 128.3 (2 ArCH), 127.7 (ArCH), 127.0 (2 ArCH), 116.4 (d, *J* = 17 Hz, C), 113.8 (d, *J* = 3 Hz, ArCH), 109.3 (d, *J* = 24 Hz, ArCH), 77.1 (CH) 73.2 (CH$_2$), 65.1 (d, *J* = 8 Hz, CH$_2$), 56.4 (CH). **^{19}F NMR (376 MHz, CDCl$_3$)** δ -119.37 (m). **IR (solid)** 2852 (w), 1692 (w), 1613 (m), 1579 (m), 1467 (s), 1363 (m), 1238 (m), 1097 (s), 1058 (s). 930 (m), 760 (s), 691 (s), 625 (m), 530 (w), 488 (w). **HRMS** (QTOF) m/z: [M+H]$^+$ Calcd for $C_{23}H_{19}FN_2O$ 359.1554; Found 359.1557.

Characterisation data of compounds **5a-h**

4-Allyl-6-fluoro-3-phenyl-4,5-dihydro-1*H*-benzo[f][1,2,4]triazepine (**5a**)

Following the procedure outlined in **GP5**. **3g** (93 mg, 0.30 mmol) was used to obtain **5a** as a white solid in 36% (30 mg, 0.108 mmol) following column chromatography (5-40% EtOAc: pentane).

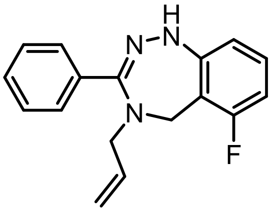

Chemical Formula: $C_{17}H_{16}FN_3$
Exact Mass: 281.1328

Appearance: White solid

Yield: 36%, 30 mg (0.108 mmol)

^{1}H NMR (400 MHz, CDCl$_3$) δ 7.54 – 7.46 (m, 2H), 7.36 – 7.29 (m, 3H), 7.09 (td, *J* = 8.1, 6.2 Hz, 1H), 6.63 – 6.50 (m, 3H), 5.86 – 5.71 (m, 1H), 5.32 – 5.19 (m, 2H), 4.50 (s, 2H), 3.40 (d, *J* = 5.9 Hz, 2H). **^{13}C NMR (101 MHz, CDCl$_3$)** δ 160.4 (d, *J* = 244 Hz, ArCH), 151.3 (d, *J* = 7 Hz, C), 151.0 (C), 137.8 (C), 134.0 (CH), 129.0 (ArCH), 129.0 (d, *J* = 10 Hz, ArCH), 128.2 (2 ArCH), 127.7 (2 ArCH), 118.7 (d, *J* = 20 Hz, C), 118.3 (CH$_2$), 112.4 (d, *J* = 3 Hz, ArCH), 107.5 (d, *J* = 23 Hz, ArCH), 56.0 (CH$_2$), 41.9 (d, *J* = 4 Hz, CH$_2$).**^{19}F NMR (376 MHz, CDCl$_3$)** δ -121.40 (m). **IR (solid)** 3298 (s, br), 2986 (w), 1609 (s), 1505 (m), 1474 (s), 1441 (s), 1369 (m), 1277 (s), 1244 (s), 1127 (s), 1066 (s), 1047 (m), 926 (m), 843 (m), 803 (m), 774 (s), 733 (s), 700 (s), 636 (m), 556 (w), 503 (m), 474 (m). **HRMS** (QTOF) m/z: [M+H]$^+$ Calcd for $C_{17}H_{17}FN_3^+$ 282.1401; Found 282.1403.

6-Fluoro-4-isobutyl-3-phenyl-4,5-dihydro-1*H*-benzo[*f*][1,2,4]triazepine (**5b**)

Following the procedure outlined in **GP5**. **3i** (76 mg, 0.248 mmol) was used to obtain **5b** as a white solid in 56% (41 mg, 0.138 mmol) following purification by column chromatography (10% EtOAc: pentane).

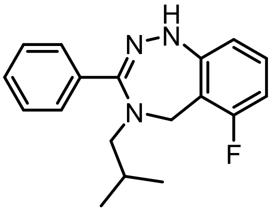

Chemical Formula: $C_{18}H_{20}FN_3$
Exact Mass: 297.1641

Appearance: white solid

Yield: 56%, 41 mg (0.138 mmol)

^{1}H NMR (400 MHz, CDCl$_3$ δ 7.52 – 7.42 (m, 2H), 7.38 – 7.29 (m, 3H), 7.04 (td, *J* = 8.1, 6.2 Hz, 1H), 6.88 (d, *J* = 2.5 Hz, 1H), 6.53 (ddd, *J* = 9.1, 8.3, 1.0 Hz, 1H), 6.42 (d, *J* = 8.0 Hz, 1H), 4.55 (d, *J* = 0.9 Hz, 2H), 2.74 (d, *J* = 7.5 Hz, 2H), 1.96 (dp, *J* = 13.7, 6.9 Hz, 1H), 0.76 (d, *J* = 6.7 Hz, 6H). **^{13}C NMR (101 MHz, CDCl$_3$)** δ 160.1 (d, *J* = 243 Hz, ArCF), 151.9 (C), 150.7 (d, *J* = 7 Hz, C), 137.8 (C), 128.9 (d, *J* = 10 Hz, ArCH), 128.8 (ArCH), 128.4 (2 ArCH), 128.2 (2 ArCH), 118.0 (d, *J* = 20 Hz, C), 112.0 (d, *J* = 3 Hz, ArCH), 106.9 (d, *J* = 23 Hz, ArCH), 61.1 (CH_2), 44.1 (d, *J* = 5 Hz, CH_2), 26.7 (CH), 20.0 (2 CH_3). **^{19}F NMR (376 MHz, CDCl$_3$)** δ -121.78 (m). **IR (solid)** 3251 (m, br), 3055 (w), 2958 (s), 2863 (m), 1727 (w), 1644 (m), 1611 (s), 1515 (s), 1463 (s), 1402 (m), 1350 (m), 1259 (m), 1228 (s), 1148 (s), 1110 (m), 1053 (m), 769 (s), 725 (m), 698 (s), 607 (m), 507 (m), 445 (w). **HRMS** (QTOF) m/z: $[M+H]^+$ Calcd for $C_{18}H_{21}FN_3^+$ 297.1636; Found 297.1629.

Methyl 2-(6-fluoro-3-phenyl-1,5-dihydro-4*H*-benzo[f][1,2,4]triazepin-4-yl)acetate (**5c**)

Following the procedure outlined in **GP5**. **3j** (65 mg, 0.19 mmol) was used to obtain **5c** as a yellow solid in 30% (18 mg, 0.057 mmol) following column chromatography (5% EtOAc: pentane).

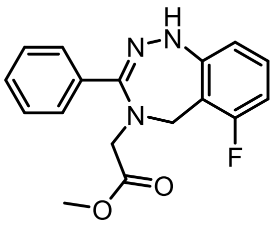

Chemical Formula: $C_{17}H_{16}FN_3O_2$
Exact Mass: 313.1227

Appearance: yellow solid

Yield: 30%, 18 mg (0.057 mmol)

^{1}H NMR (400 MHz, CDCl$_3$) δ 7.57 – 7.48 (m, 2H), 7.37 – 7.27 (m, 3H), 7.09 (td, *J* = 8.2, 6.2 Hz, 1H), 6.78 (d, *J* = 2.5 Hz, 1H), 6.58 (td, *J* = 8.5, 1.0 Hz, 1H), 6.51 (dt, *J* = 8.0, 0.8 Hz, 1H), 4.58 (s, 2H), 3.65 (s, 3H), 3.60 (s, 2H). **^{13}C NMR (101 MHz, CDCl$_3$)** δ 170.6 (C=O), 160.2 (d, *J* = 245 Hz, ArCF), 150.8 (d, *J* = 7 Hz, C), 149.6 (C), 136.9 (C), 129.3 (d, *J* = 10 Hz, ArCH), 129.2 (ArCH), 128.5 (2 ArCH), 127.7 (2 ArCH), 117.9 (d, *J* = 20 Hz, C), 112.5 (d, *J* = 3 Hz, ArCH), 107.6 (d, *J* = 23 Hz, ArCH), 54.4 (CH_2), 52.0 (CH_3), 44.4 (d, *J* = 4 Hz, CH_2). **^{19}F NMR (376 MHz, CDCl$_3$)** δ -122.30 (m). **IR (solid)** 3306 (w), 3141 (w), 2917 (w), 2883 (w), 1752 (s), 1612 (m), 1515 (m), 1472 (m), 1414 (m), 1364 (m), 1270 (m), 1232 (m), 1204 (s), 1134 (s), 1050 (m), 989 (s), 846 (m), 778 (s), 735 (s), 704 (s), 615 (m), 578 (m), 526 (m), 502 (m), 428 (m). **HRMS** (QTOF) m/z: $[M+H]^+$ Calcd for $C_{17}H_{17}FN_3O_2^+$ 314.1299; Found 314.1302.

4-Cyclopentyl-6-fluoro-3-phenyl-4,5-dihydro-1*H*-benzo[*f*][1,2,4]triazepine (**5d**)

Following the procedure outlined in **GP5**. **3k** (98 mg, 0.29 mmol) was used to obtain **5d** as a white solid in 55% (50 mg, 0.16 mmol) following column chromatography (5% EtOAc: pentane).

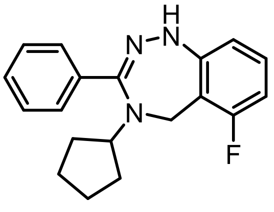

Chemical Formula: $C_{19}H_{20}FN_3$
Exact Mass: 309.1641

Appearance: white solid

Yield: 55%, 50 mg (0.16 mmol)

^{1}H NMR (400 MHz, CDCl$_3$) δ 7.53 – 7.42 (m, 2H), 7.41 – 7.28 (m, 3H), 7.03 (td, *J* = 8.1, 6.2 Hz, 1H), 6.81 (s, br., 1H), 6.50 (ddd, *J* = 9.2, 8.3, 1.0 Hz, 1H), 6.40 (dt, *J* = 8.0, 0.8 Hz, 1H), 4.48 (s, 2H), 3.68 – 3.56 (m, 1H), 1.70 – 1.55 (m, 6H), 1.38 (tdd, *J* = 7.9, 5.4, 3.1 Hz, 2H). **^{13}C NMR (101 MHz, CDCl$_3$)** δ 159.5 (d, *J* = 243 Hz, ArCF), 151.2 (C), 150.1 (d, *J* = 7 Hz, C), 138.4 (C), 128.7 (d, *J* = 10 Hz, ArCH), 128.6 (ArCH), 128.2 (2 ArCH), 127.8 (2 ArCH), 118.4 (d, *J* = 19 Hz, C), 111.5 (d, *J* = 3 Hz, ArCH), 106.4 (d, *J* = 23 Hz, ArCH), 62.2 (CH), 39.6 (d, *J* = 5 Hz, CH$_2$), 28.8 (2 CH$_2$), 23.9 (2 CH$_2$). **^{19}F NMR (376 MHz, CDCl$_3$)** δ -120.97 (m). **IR (solid)** 3253 (m, br), 3052 (w), 2951 (m, br), 2867 (m), 1612 (s), 1520 (s), 1479 (m), 1407 (m), 1345 (m), 1268 (s), 1229 (m), 1169 (m), 1146 (m), 1054 (m), 1028 (m), 922 (w), 821 (w), 754 (s), 697 (s), 508 (m), 478 (w). **HRMS** (QTOF) m/z: [M+H]$^+$ Calcd for $C_{19}H_{21}FN_3^+$ 310.1714; Found 310.1715.

6-Fluoro-3-phenyl-4-(4-(trifluoromethoxy)phenyl)-4,5-dihydro-1*H*-benzo[*f*][1,2,4]triazepine (**5e**)

Following the procedure outlined in **GP5**. Using **3l** **5e** was not obtained.

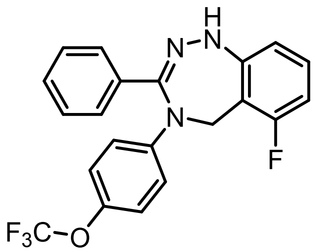

Chemical Formula: $C_{21}H_{15}F_4N_3O$
Exact Mass: 401.1151

6-Fluoro-3,4-diphenyl-4,5-dihydro-1*H*-benzo[*f*][1,2,4]triazepine (**5f**)

Following the procedure outlined in **GP5**. Using **3m** **5f** was not obtained.

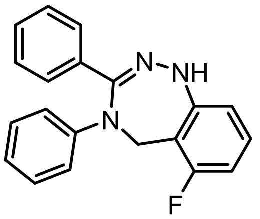

Chemical Formula: $C_{20}H_{16}FN_3$
Exact Mass: 317.1328

6-Fluoro-4-(4-methoxybenzyl)-3-phenyl-4,5-dihydro-1*H*-benzo[*f*][1,2,4]triazepine (**5g**)

Following the procedure outlined in **GP5**. **3n** (167 mg, 0.43 mmol) was used to obtain **5g** as a white solid in 39% (60 mg, 0.17 mmol) following column chromatography (10% EtOAc: pentane).

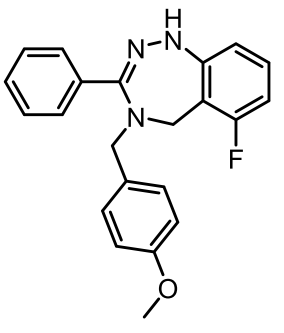

Chemical Formula: $C_{22}H_{20}FN_3O$
Exact Mass: 361.1590

Appearance: White Solid

Yield: 39%, 60 mg (0.17 mmol)

1H NMR (400 MHz, $CDCl_3$) δ 7.64 – 7.55 (m, 2H), 7.40 – 7.29 (m, 3H), 7.26 – 7.17 (m, 2H), 7.06 (td, *J* = 8.2, 6.2 Hz, 1H), 6.92 – 6.82 (m, 2H), 6.69 (d, *J* = 2.5 Hz, 1H), 6.58 – 6.47 (m, 2H), 4.44 (s, 2H), 3.97 (s, 2H), 3.81 (s, 3H). **^{13}C NMR (101 MHz, $CDCl_3$)** δ 160.36 (d, *J* = 244.5 Hz), 158.92, 151.32, 151.08, 137.93, 129.32, 129.24, 128.96, 128.86, 128.32, 127.96, 118.31 (d, *J* = 20.0 Hz), 112.35 (d, *J* = 3.0 Hz), 107.57 (d, *J* = 23.0 Hz), 56.29, 55.26, 42.27 (d, *J* = 3.9 Hz). **^{19}F NMR (376 MHz, $CDCl_3$)** δ -120.34 (m). **IR (solid)** 3303(w), 3249 (w), 2930 (w), 2833 (w), 1611 (s), 1511 (m), 1489 (m), 1473 (m), 1440 (m), 1362 (w), 1289 (w), 1272 (m), 1248 (m), 1165 (w), 1119 (m), 1040 (s), 1022 (s), 925 (w), 775 (s), 730 (m), 700 (m), 618 (w), 514 (w), 503 (w). **HRMS** (QTOF) m/z: $[M+H]^+$ Calcd for $C_{22}H_{21}FN_3O^+$ 362.1663; Found 362.1673.

Control experiment

3a (313 mg, 1.02 mmol) was run following the procedure outlined in **GP5** with an addition of 1 equiv. of diethylamine (75 mg, 1.02 mmol). It was found from this that the yield of product **4a** was lowered and the formation of two new products were observed in NMR. The products were isolated as regio-isomers and assigned the structures shown for **6a** and **6b** based on NMR and HRMS data. The shown yields were determined by qNMR.

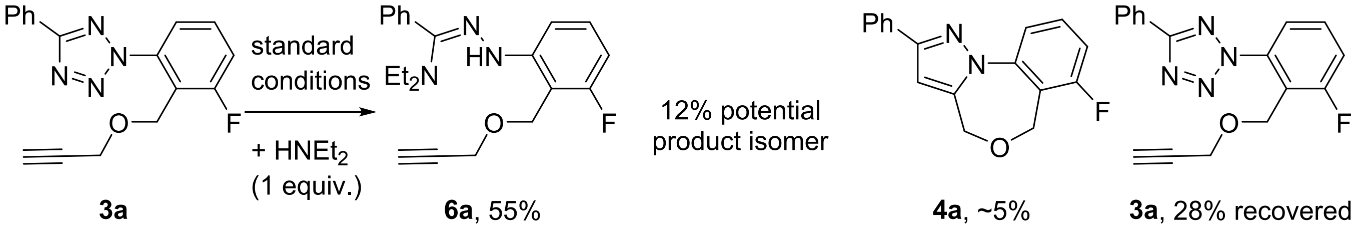

3a | **6a**, 55% | **4a**, ~5% | **3a**, 28% recovered

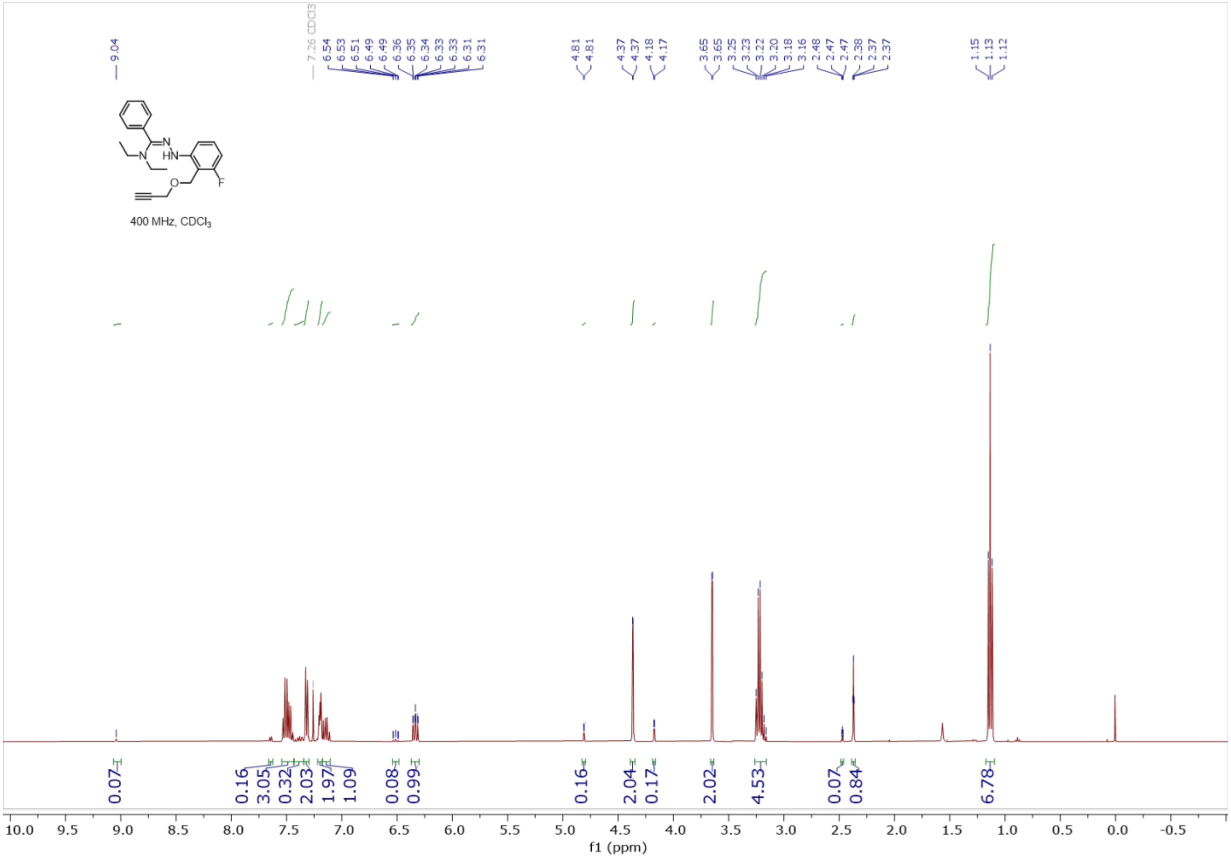

Figure SI4: ^{1}H NMR of isolated isomers **6a**.

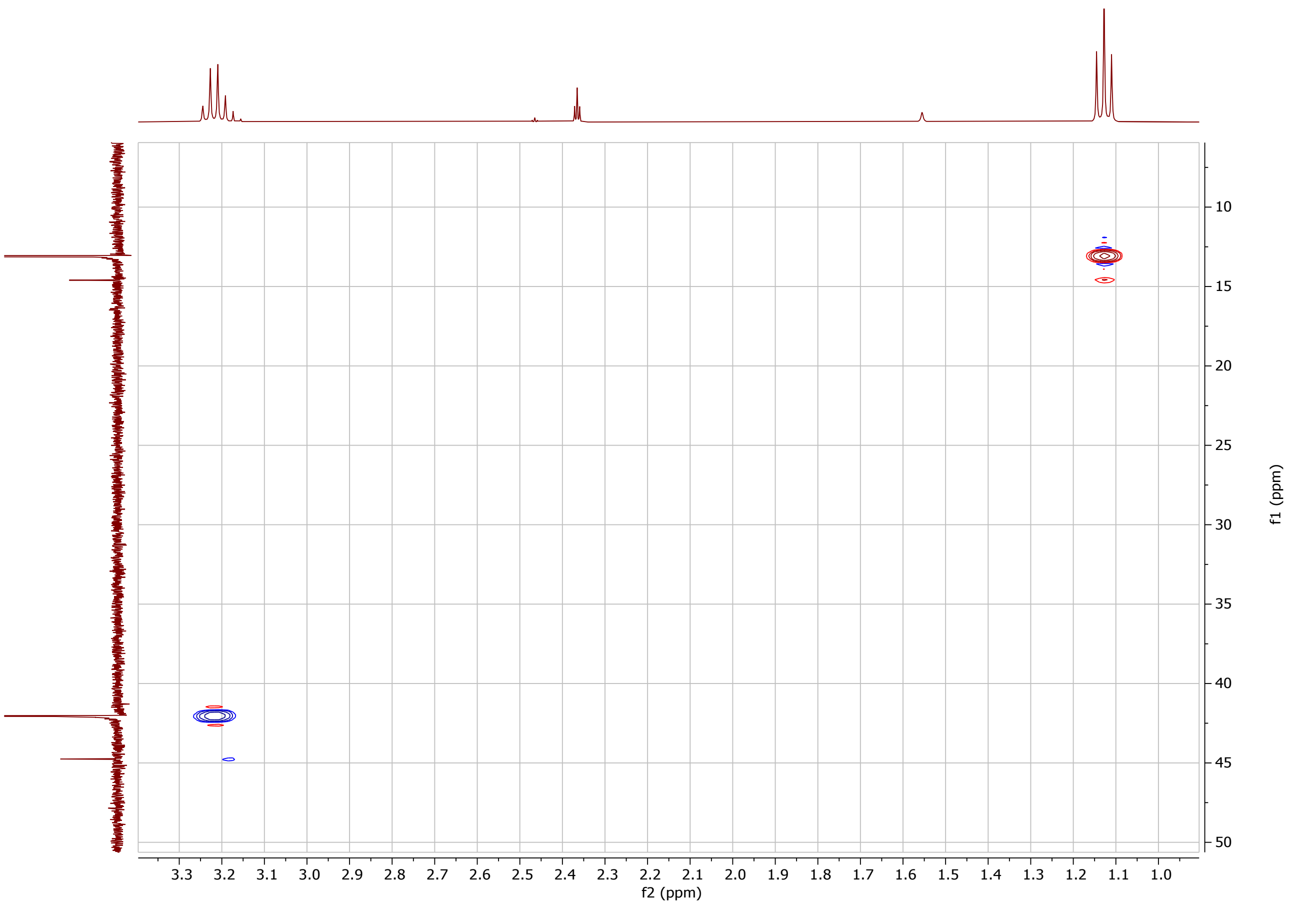

Figure SI5: HSQC showing overlapping peaks from ethyl chains of isomers.

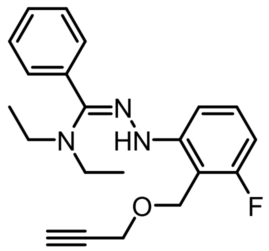

Chemical Formula: $C_{21}H_{24}FN_3O$
Exact Mass: 353.1903

HRMS (QTOF) m/z: $[M+H]^+$ Calcd for $C_{21}H_{25}FN_3O^+$ 354.1976; Found 354.1977.

X-Ray Data for **4c**, **4f** and **5a**

Sample preparation

Crystals of compound **4c** were prepared through slow evaporation of chloroform and resuspended in minimal volume of pentane. Compound **4f** and **5a** were dissolved in minimal volume of a 4:1 solution of DCM: cyclohexane. The solvent was allowed to slowly evaporate to afford the crystals as suspension in cyclohexane.

X-Ray Diffraction Experiment for Compound 4c

The structure of compound **4c** was measured at 102(4) K using a SuperNova, dual four-circle diffractometer with an Atlas detector and a low-temperature device. Equipped with a micro-focus sealed X-ray tube, the setup utilized Cu Kα radiation (λ = 1.54184 Å) and a mirror as the monochromator. Data processing was carried out using Crysalispro, followed by a multi-scan absorption correction with SCALE3 ABSPACK.[1]

To solve the structure, we used dual methods with SHELXT and refined it through full-matrix least-squares methods against F2, implemented in SHELXL within the Olex2 environment.[2-4] All non-hydrogen atoms were refined anisotropically, while hydrogen atoms were refined isotropically using calculated positions in a riding model. Uiso values for terminal sp3 carbons were constrained to 1.5 times, and for all other carbons to 1.2 times, the Ueq of their pivot atoms.

Table 1. Crystal data and structure refinement for Compound **4c**.

**CCDC number**	**2391147**
Empirical formula	$C_{17}H_{13}FN_2O$
Formula weight	280.29
Temperature [K]	102(4)
Crystal system	monoclinic
Space group (number)	$P2_1/n$ (14)
a [Å]	12.9798(10)
b [Å]	7.383(2)
c [Å]	13.777(4)
α [°]	90
β [°]	93.795(10)
γ [°]	90
Volume [Å^3]	1317.3(5)
Z	4

ρ_{calc} [gcm^{-3}]	1.413
μ [mm^{-1}]	0.815
F(000)	584
Crystal size [mm^3]	0.394×0.139×0.095
Crystal colour	translucent light colourless
Crystal shape	block
Radiation	Cu K_α (λ=1.54184 Å)
2θ range [°]	9.07 to 152.91 (0.79 Å)
Index ranges	−16 ≤ h ≤ 16<br>−7 ≤ k ≤ 9<br>−17 ≤ l ≤ 17
Reflections collected	22415
Independent reflections	2749<br>R_{int} = 0.0979<br>R_{sigma} = 0.0356
Completeness to<br>θ = 67.684°	100.0 %
Data / Restraints / Parameters	2749/0/190
Absorption correction<br>T_{min}/T_{max} (method)	0.64069/1.00000<br>(multi-scan)
Goodness-of-fit on F^2	1.064
Final R indexes<br>[I≥2σ(I)]	R_1 = 0.0472<br>wR_2 = 0.1230
Final R indexes<br>[all data]	R_1 = 0.0546<br>wR_2 = 0.1321
Largest peak/hole [eÅ^{-3}]	0.27/−0.37

Hydrogen Bonds in the structure.

D-H A	D-H (Å)	H…A (Å)	D…A (Å)	D-H…A (°)	Symmetry	Graph
C9-H9…F1	0.9500	2.4800	3.3260(19)	148.00	1/2+x,1/2-y,-1/2+z	C(8)
C17-H17…O1	0.9500	2.5500	3.415(2)	152.00	1-x,1-y,2-z	$R^2_2(6)$

In compound **4c**, two weak hydrogen bond interactions, C9–H9···F1 and C17–H17···O1, contribute significantly to the crystal organization. The C17–H17···O1 interaction, with an H···O distance of 2.55 Å and an angle of 152°, forms a dimer with an $R^2_2(6)$ motif, linking molecules in pairs via a C–H···O hydrogen bond (symmetry 1 - x, 1 - y, 2 - z). This dimer motif stabilizes the crystal packing.

Meanwhile, the C9–H9···F1 interaction, with an H···F distance of 2.48 Å and an angle of 148°, creates an infinite **C(8)** chain, generating a fishbone structure along the crystal (symmetry 1/2 + x, 1/2 - y, -1/2 + z). Although these C–H···F and C–H···O interactions are weaker than classical hydrogen bonds, they enhance the stability of the crystal lattice and promote a unique supramolecular arrangement that influences the structure and properties of the material.

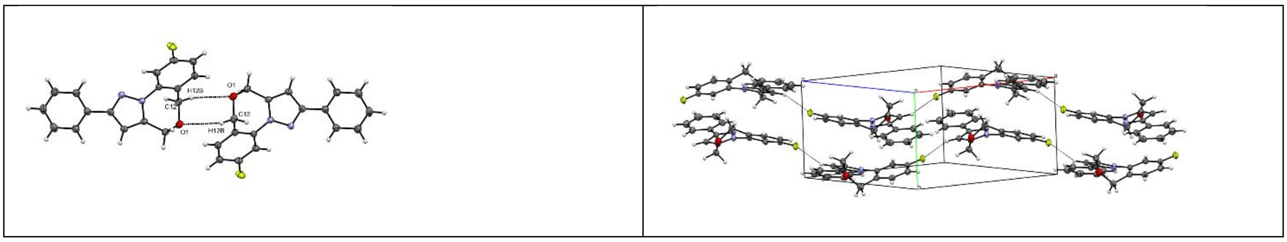

Figure SI6: Hydrogen bonding interactions in compound 4C: (a) C-H…O hydrogen bonds resulting in the formation of $R^2_2(6)$ dimers; (b) C-H…F interaction which forms infinite chain C(8).

References:

[1] Crysalispro, *1.171.42.92*, **2023**, Rigaku OD.

[2] G. M. Sheldrick, *Acta Cryst.* **2015**, *A71*, 3–8, doi:10.1107/S2053273314026370.

[3] G. M. Sheldrick, *Acta Cryst.* **2015**, *C71*, 3–8, doi:10.1107/S2053229614024218.

[4] O. V. Dolomanov, L. J. Bourhis, R. J. Gildea, J. A. K. Howard, H. Puschmann, *J. Appl. Cryst.* **2009**, *42*, 339-341, doi:10.1107/S0021889808042726.

Crystals of **4f** and **5a** were mounted on a MiTeGen micromount with NVH immersion oil. Data were collected from a shock-cooled single crystal at 100(2) K on a Bruker D8 Quest ECO three-circle diffractometer with a sealed X-ray tube using a graphite monochromator and a Bruker PHOTON III C7 detector. The diffractometer was equipped with an Oxford Cryostream 800 low temperature device and used Mo K_α radiation (λ = 0.71073 Å). All data were integrated with SAINT and a multi-scan absorption correction using SADABS was applied.[1,2]

Structures were solved by dual methods using SHELXT and refined by full-matrix least-squares methods against F^2 by SHELXL using Olex2.[3-5] All non-hydrogen atoms were refined with anisotropic displacement parameters. All hydrogen atoms were refined with isotropic displacement parameters. Some of their coordinates were refined freely and some on calculated positions using a riding model with their U_{iso} values constrained to 1.5 times the U_{eq} of their pivot atoms for terminal sp^3 carbon atoms and 1.2 times for all other carbon atoms.

Crystallographic data for the structures reported here have been deposited with the Cambridge Crystallographic Data Centre.[6] CCDC 2391436-2391437 contain the supplementary crystallographic data for this paper. These data can be obtained free of charge from The Cambridge Crystallographic Data Centre via www.ccdc.cam.ac.uk/structures.

[1] Bruker, *SAINT*, Bruker AXS Inc., Madison, Wisconsin, USA.

[2] L. Krause, R. Herbst-Irmer, G. M. Sheldrick, D. Stalke, *J. Appl. Cryst.* **2015**, *48*, 3–10, doi:10.1107/S1600576714022985.

[3] G. M. Sheldrick, *Acta Cryst.* **2015**, *A71*, 3–8, doi:10.1107/S2053273314026370.

[4] G. M. Sheldrick, *Acta Cryst.* **2015**, *C71*, 3–8, doi:10.1107/S2053229614024218.

[5] O. V. Dolomanov, L. J. Bourhis, R. J. Gildea, J. A. K. Howard, H. Puschmann, *J. Appl. Cryst.* **2009**, *42*, 339-341, doi:10.1107/S0021889808042726.

[6] C. R. Groom, I. J. Bruno, M. P. Lightfoot, S. C. Ward, *Acta Cryst.* **2016**, *B72*, 171–179, doi:10.1107/S2052520616003954.

Structure Tables

	4f	5a
CCDC number	2391436	2391437
Empirical formula	$C_{17}H_{15}FN_2O$	$C_{17}H_{16}FN_3$
Formula weight	282.31	281.33
Temperature [K]	100(2)	100(2)
Crystal system	triclinic	triclinic
Space group (number)	$P\bar{1}$ (2)	$P\bar{1}$ (2)
a [Å]	8.32230(10)	9.38760(10)
b [Å]	12.2966(2)	12.1698(2)
c [Å]	14.0525(2)	13.2417(2)
α [°]	80.3537(6)	105.4493(7)
β [°]	87.4430(7)	91.0864(7)
γ [°]	71.0392(6)	98.0181(7)
Volume [Å^3]	1340.74(3)	1441.39(4)
Z	4	4
ρ_{calc} [gcm^{-3}]	1.399	1.296
μ [mm^{-1}]	0.098	0.088
F(000)	592	592
Crystal size [mm^3]	0.385×0.193×0.175	0.473×0.105×0.076
Crystal colour	colourless	colourless
Crystal shape	block	block
Radiation	Mo K_α (λ=0.71073 Å)	Mo K_α (λ=0.71073 Å)
2Θ range [°]	5.76 to 61.01 (0.70 Å)	4.39 to 62.06 (0.69 Å)
Index ranges	$-11 \le h \le 11$<br>$-17 \le k \le 17$<br>$-20 \le l \le 20$	$-13 \le h \le 13$<br>$-17 \le k \le 17$<br>$-19 \le l \le 19$
Reflections collected	52985	46628
Independent reflections	8165<br>R_{int} = 0.0267<br>R_{sigma} = 0.0172	9199<br>R_{int} = 0.0443<br>R_{sigma} = 0.0380
Completeness	99.7 %	99.9 %
Data / Restraints / Parameters	8165/0/379	9199/2/388
Goodness-of-fit on F^2	1.023	1.009
Final *R* indexes [$I \ge 2\sigma(I)$]	R_1 = 0.0382<br>wR_2 = 0.1020	R_1 = 0.0462<br>wR_2 = 0.1039
Final *R* indexes [all data]	R_1 = 0.0451<br>wR_2 = 0.1077	R_1 = 0.0692<br>wR_2 = 0.1172
Largest peak/hole [eÅ^{-3}]	0.44/-0.21	0.43/-0.25
Flack X parameter	-	-
Extinction coefficient		0.0087(15)

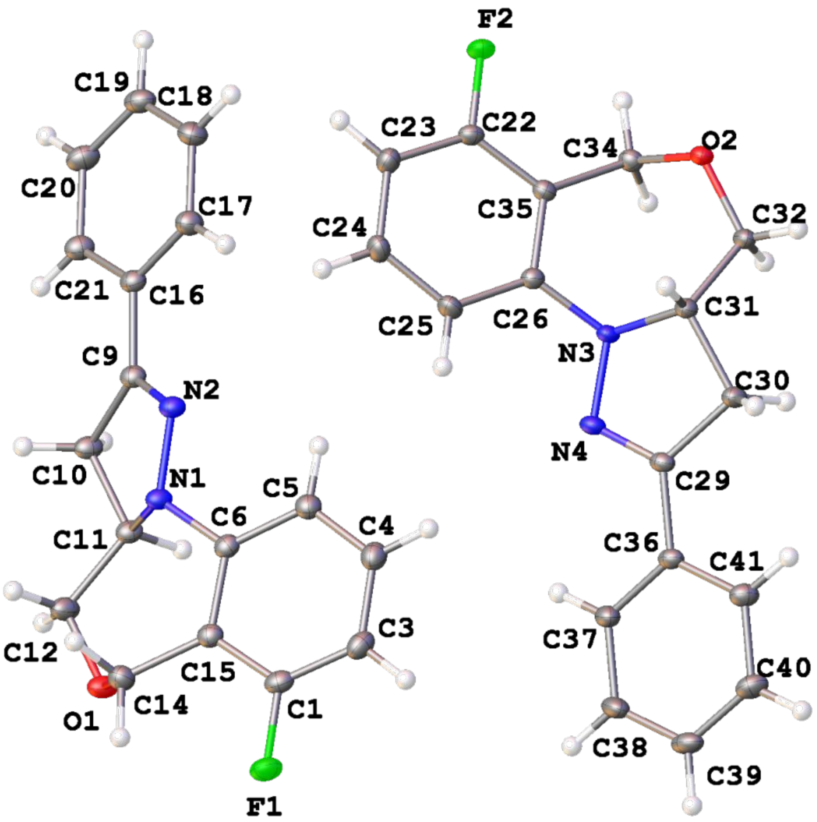

Fig. SI7. Molecular structure of **4f** with fully labelled atoms. Displacement parameters shown at 50% probability.

4f Hydrogen bonding

D–H···A [Å]	d(D–H) [Å]	d(H···A) [Å]	d(D···A) [Å]	<(DHA) [°]
C19–H19···O1$^{\#1}$	0.95	2.51	3.3986(12)	157
C39–H39···O2$^{\#2}$	0.95	2.45	3.3850(11)	167

Symmetry transformations used to generate equivalent atoms:
#1: +X, -1+Y, +Z;   #2: +X, 1+Y, +Z;

In **4f** each of the two independent molecules in the asymmetric unit displays the same primary hydrogen bonding motif, C(10), an infinite chain parallel to the b-axis.

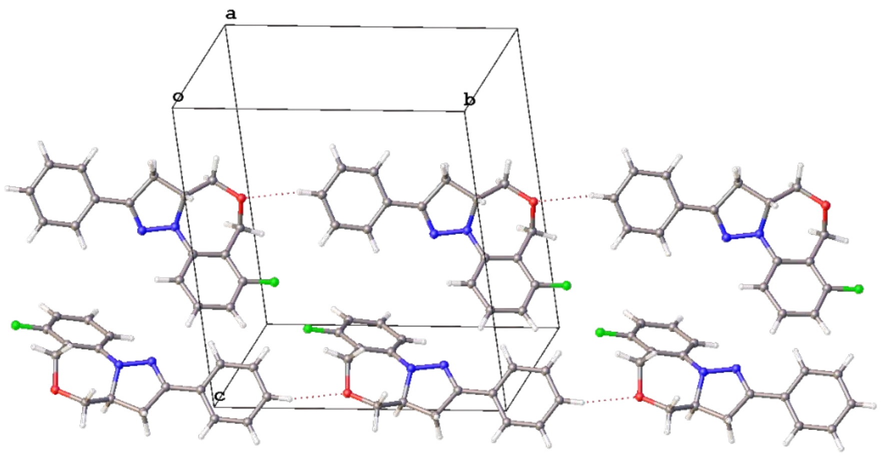

Fig. SI8. Hydrogen bonding in **4f** with the CH...O interaction forming an infinite chain.

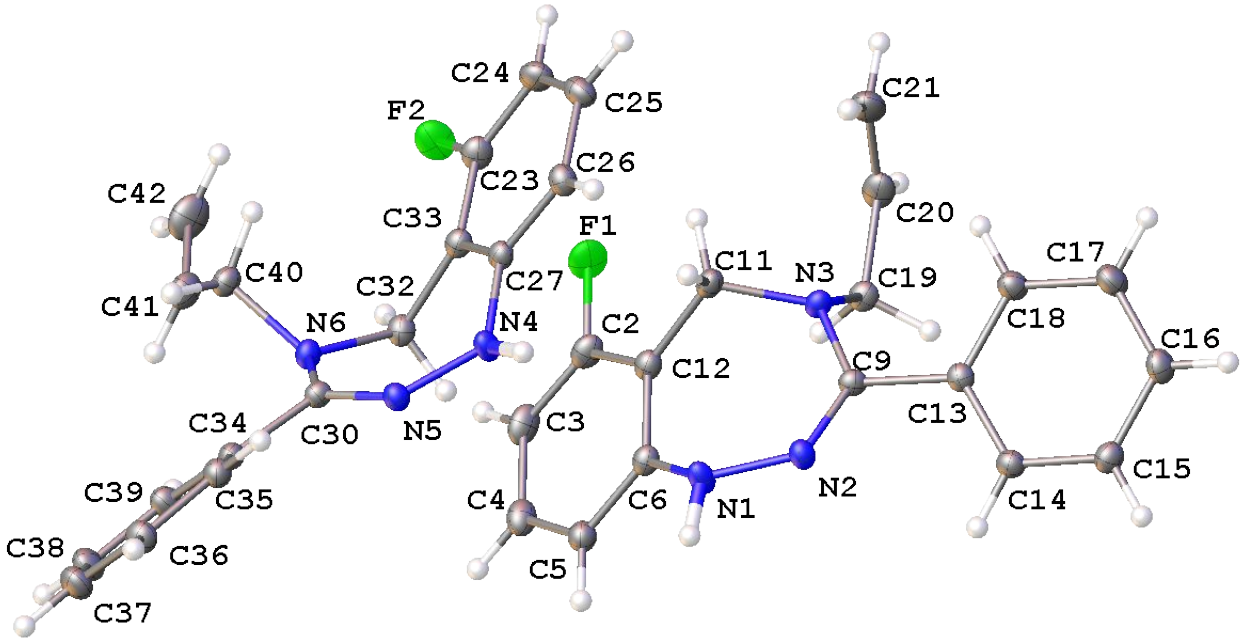

Fig. SI9. Molecular structure of **5a** with fully labelled atoms. Displacement parameters shown at 50% probability.

5a Hydrogen bonding

D–H···A [Å]	d(D–H) [Å]	d(H···A) [Å]	d(D···A) [Å]	<(DHA) [°]
C20–H20···F1#1	0.95	2.47	3.2966(15)	145
N4–H4A···N5#2	0.880(9)	2.238(12)	3.0120(14)	146.6(15)
N1–H1···N2#3	0.877(9)	2.189(11)	2.9861(14)	151.0(15)

Symmetry transformations used to generate equivalent atoms:
#1: 1-X, 2-Y, 2-Z;   #2: 2-X, 1-Y, 1-Z;   #3: 1-X, 1-Y, 1-Z;

In **5a**, the N-H of the triazepine in each of the independent molecules in the asymmetric unit from a hydrogen bonded ring with the neighbouring triazepine with a $R_2^2(6)$ motif. This, as seen in **4c**, stabilizes the packing. However, the C20-H20...F1 interactions forms another ring motif $R_2^2(16)$ which extends one chain as a ribbon. The other independent molecule in the asymmetric unit with F2 has a much longer intermolecular C24H24...F2 (2-x, 2-y, 2-z) distance of 3.4518(17) Å, considerably longer than that seen between the other molecule with F1.

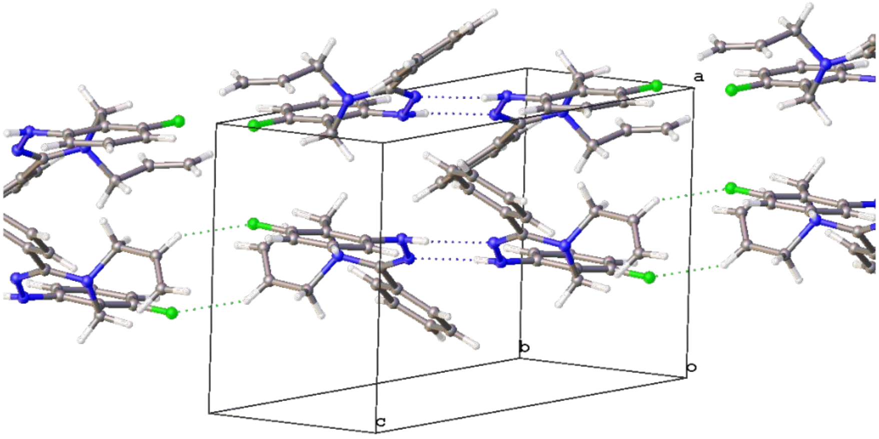

Fig. SI10: Hydrogen bonding in **5a** showing both types of H bonding interaction (dotted lines).

NMR Spectra

2-(3-Fluoro-2-methylphenyl)-5-phenyl-2*H*-tetrazole (**1a**)

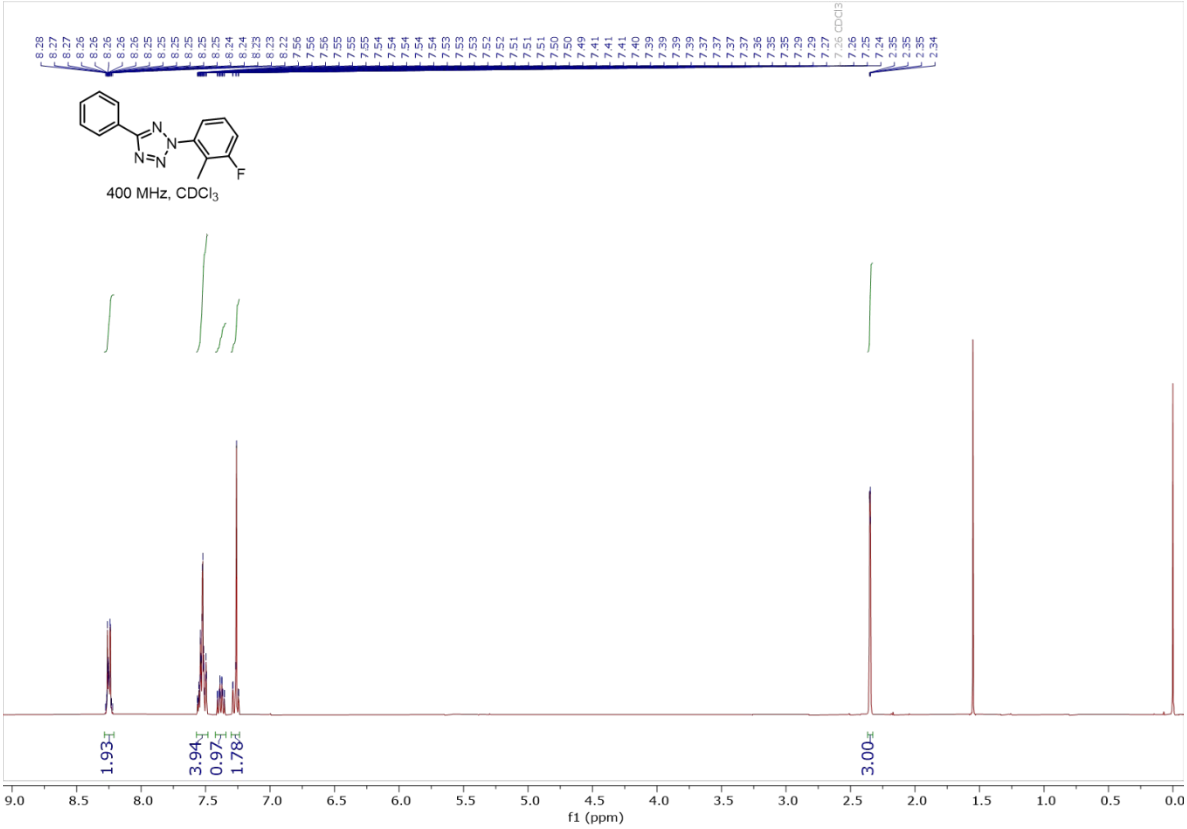

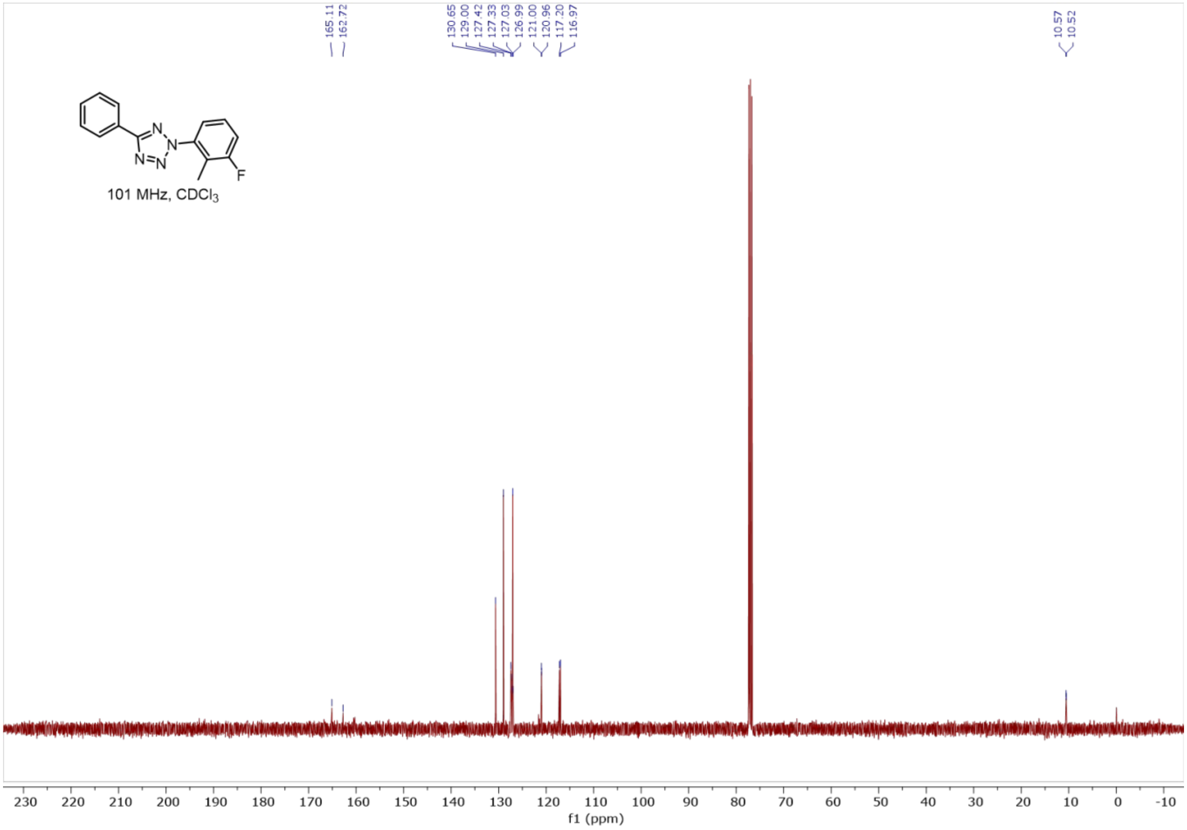

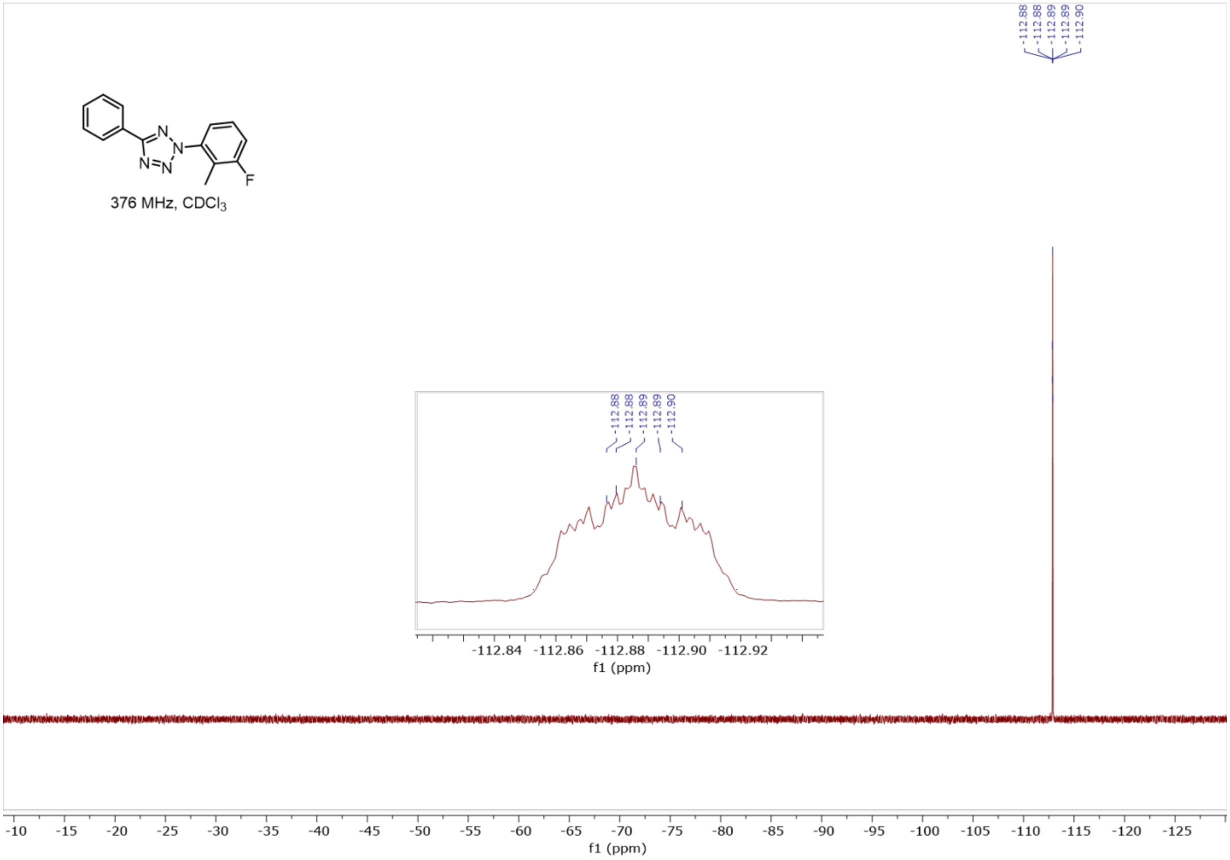

2-(2,6-Dimethylphenyl)-5-phenyl-2H-tetrazole (**1b**)

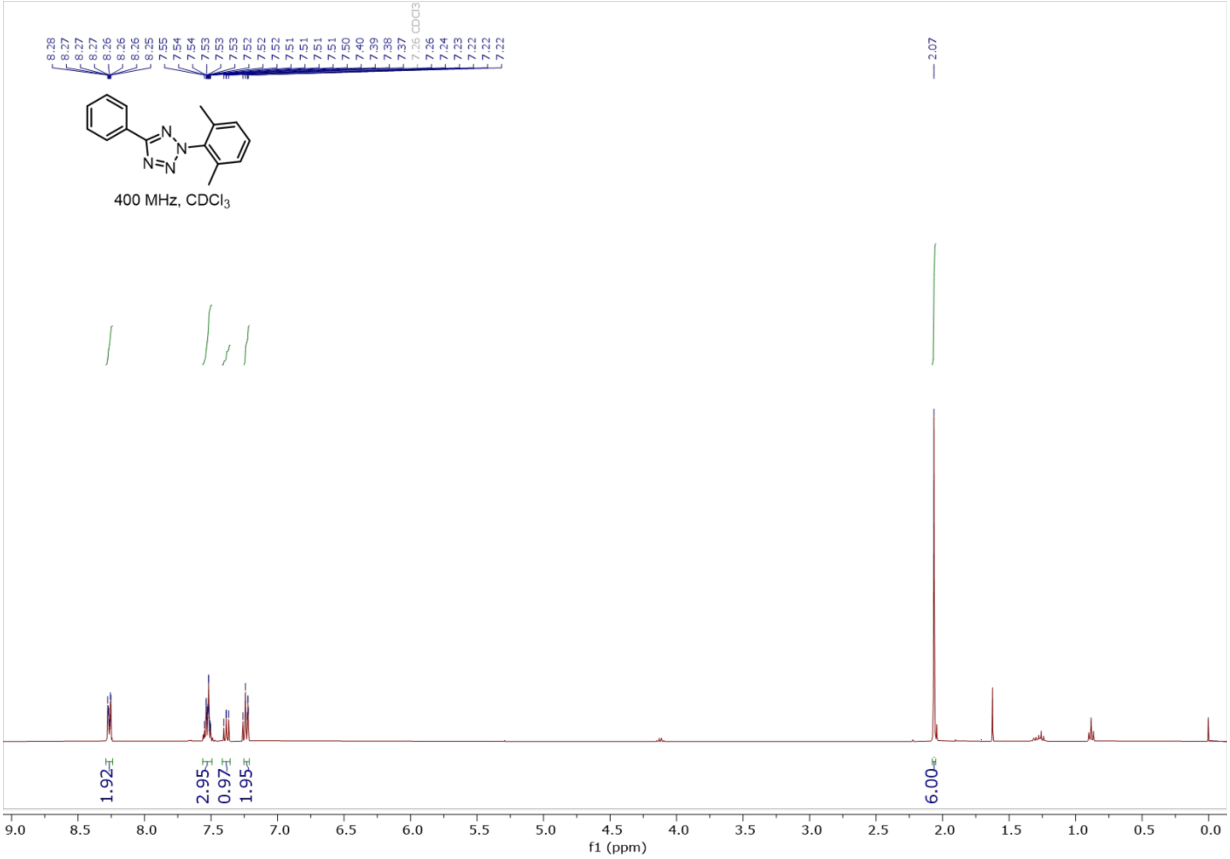

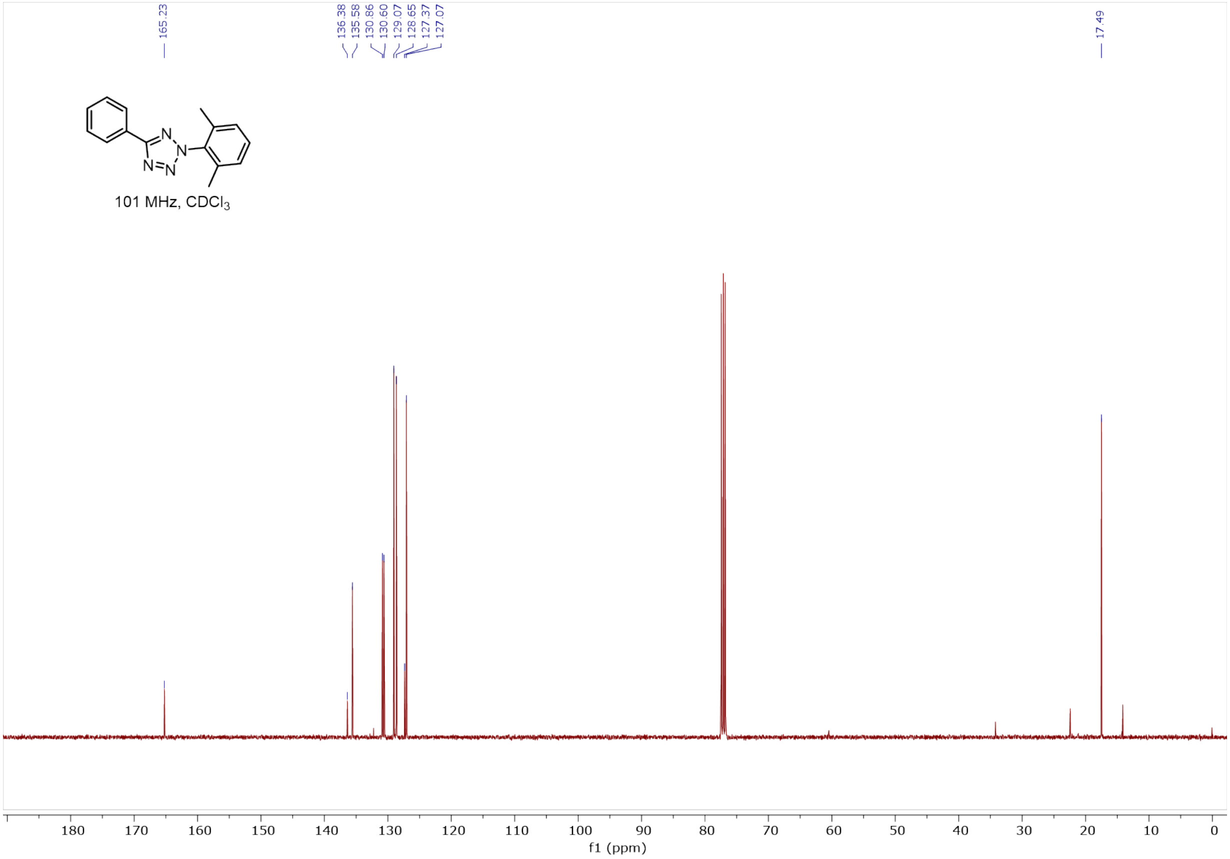

2-(5-Fluoro-2-methylphenyl)-5-phenyl-2*H*-tetrazole (**1c**)

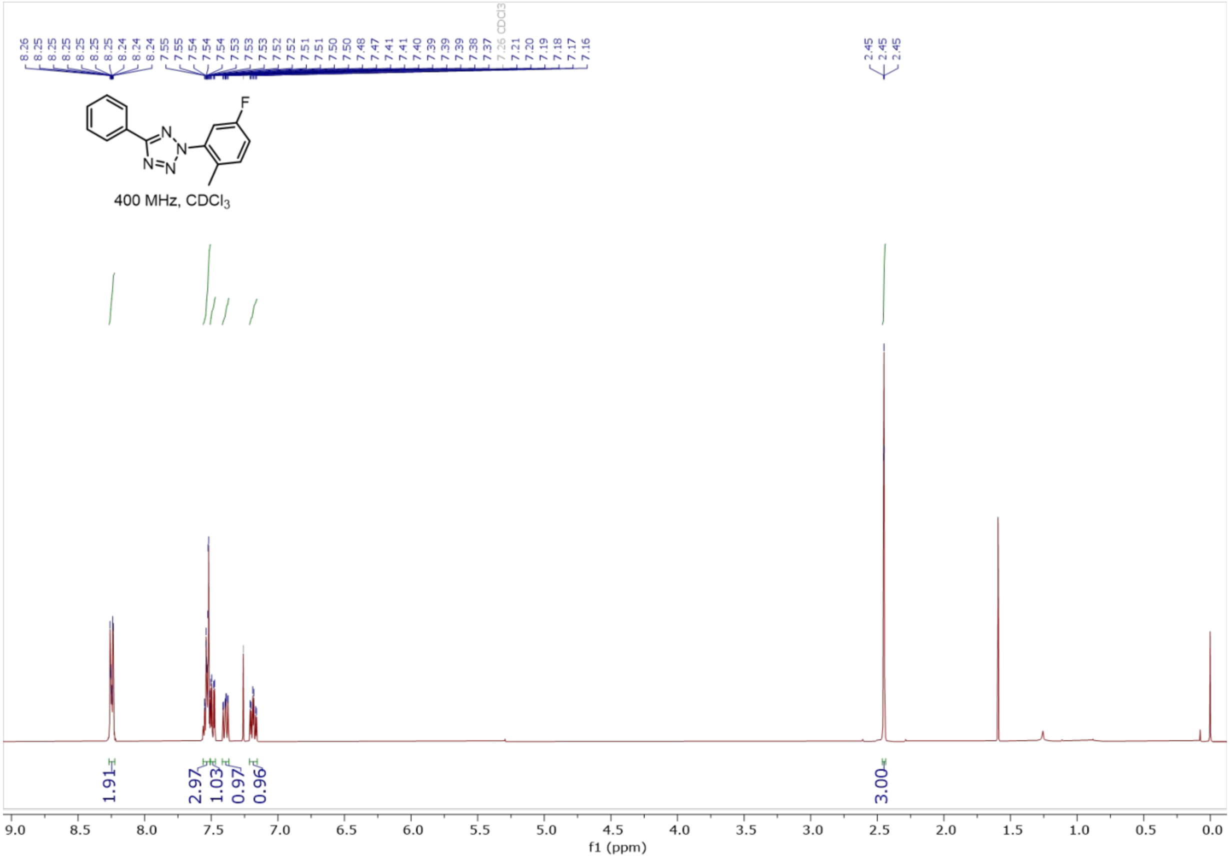

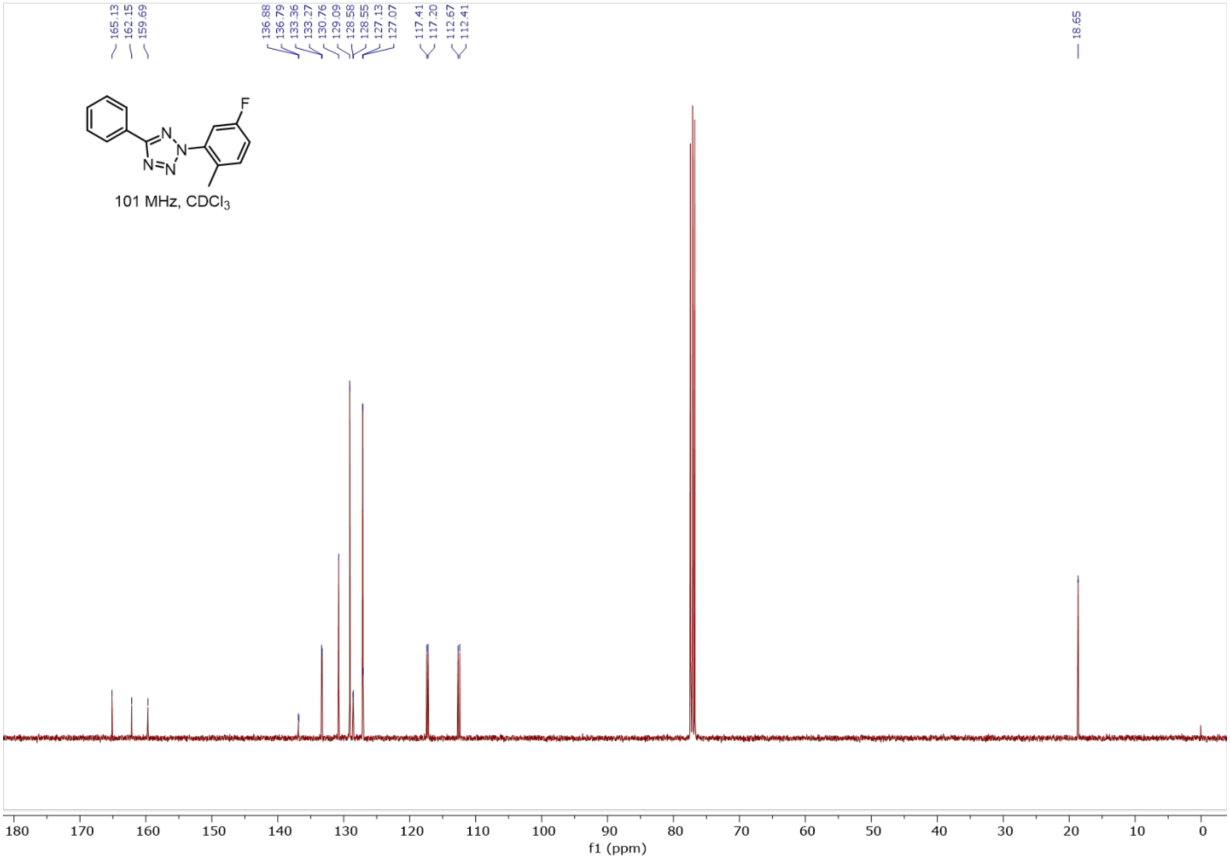
165.13
162.15
159.69
136.88
136.79
133.36
133.27
130.76
129.09
128.58
128.55
127.13
127.07
117.41
117.20
112.67
112.41
18.65
F
N
N
N
N
101 MHz, CDCl3
180 170 160 150 140 130 120 110 100 90 80 70 60 50 40 30 20 10 0
f1 (ppm)

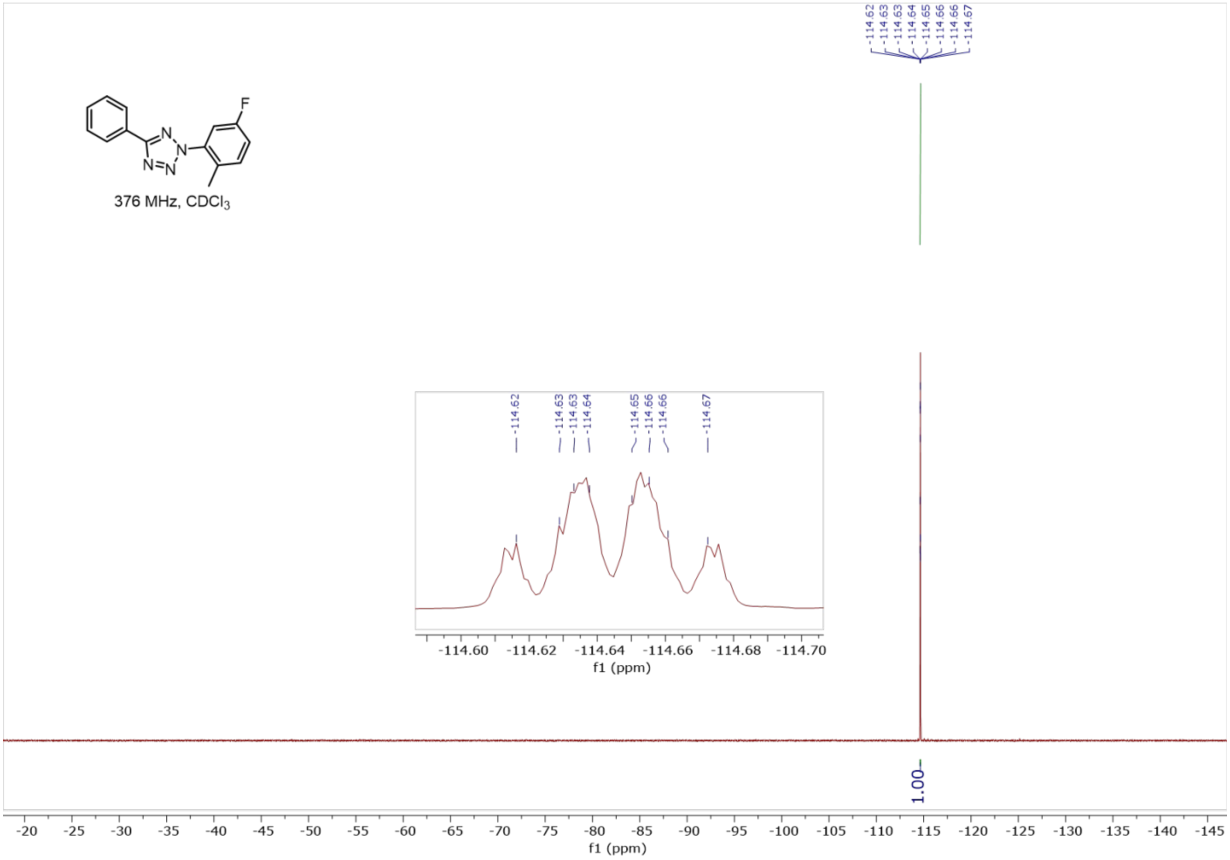
-114.62
-114.63
-114.63
-114.64
-114.65
-114.66
-114.66
-114.67
F
N
N
N
N
376 MHz, CDCl3
-114.60 -114.62 -114.64 -114.66 -114.68 -114.70
f1 (ppm)
1.00
-20 -25 -30 -35 -40 -45 -50 -55 -60 -65 -70 -75 -80 -85 -90 -95 -100 -105 -110 -115 -120 -125 -130 -135 -140 -145
f1 (ppm)

2-(2-(Bromomethyl)-3-fluorophenyl)-5-phenyl-2*H*-tetrazole (**2a**)

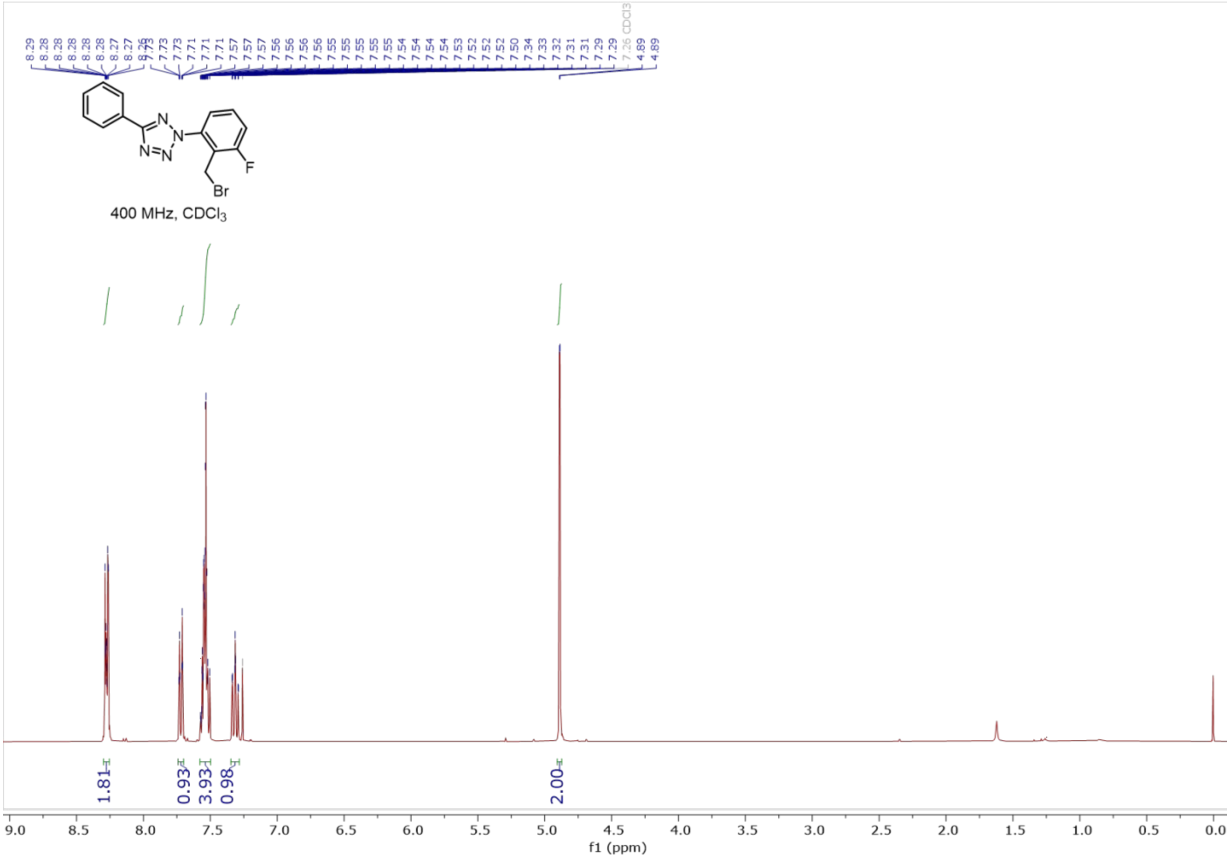

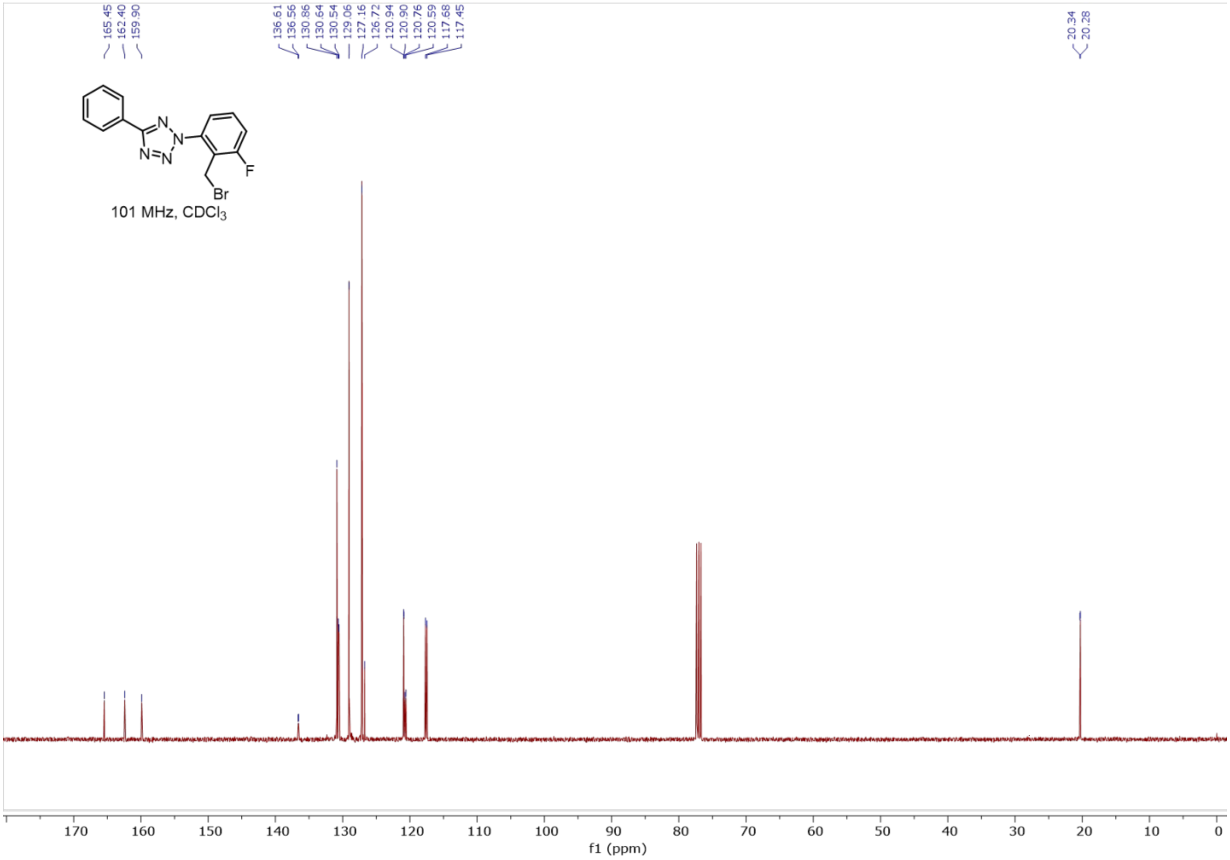

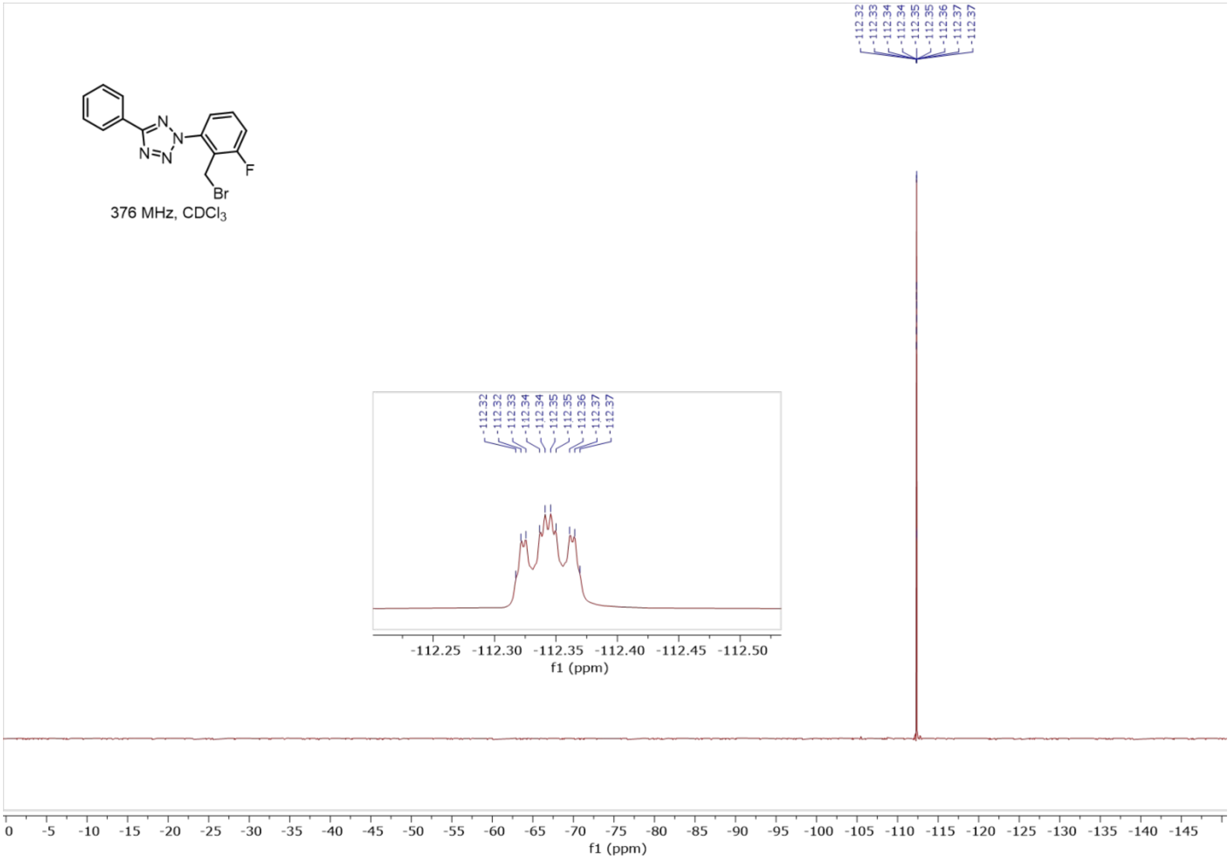

2-(2,6-Bis(bromomethyl)phenyl)-5-phenyl-2*H*-tetrazole (**2b**)

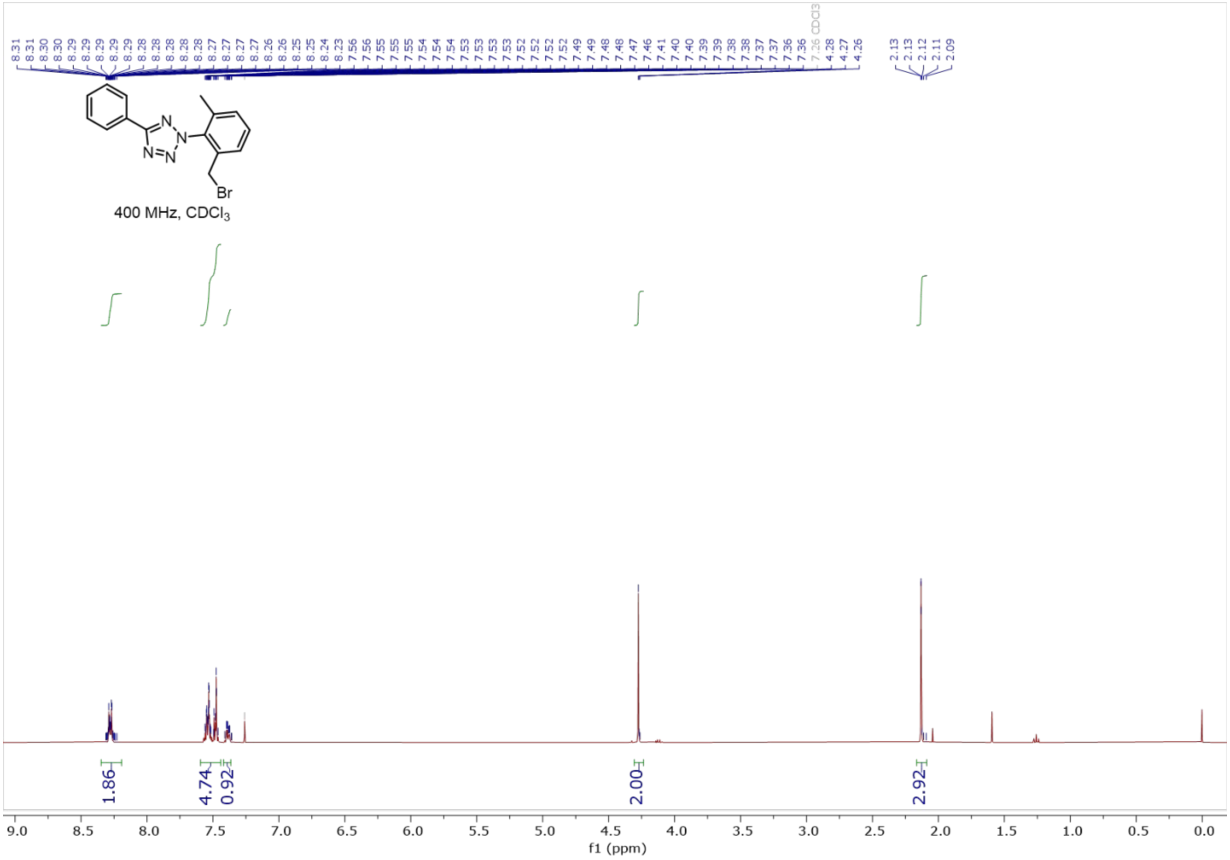

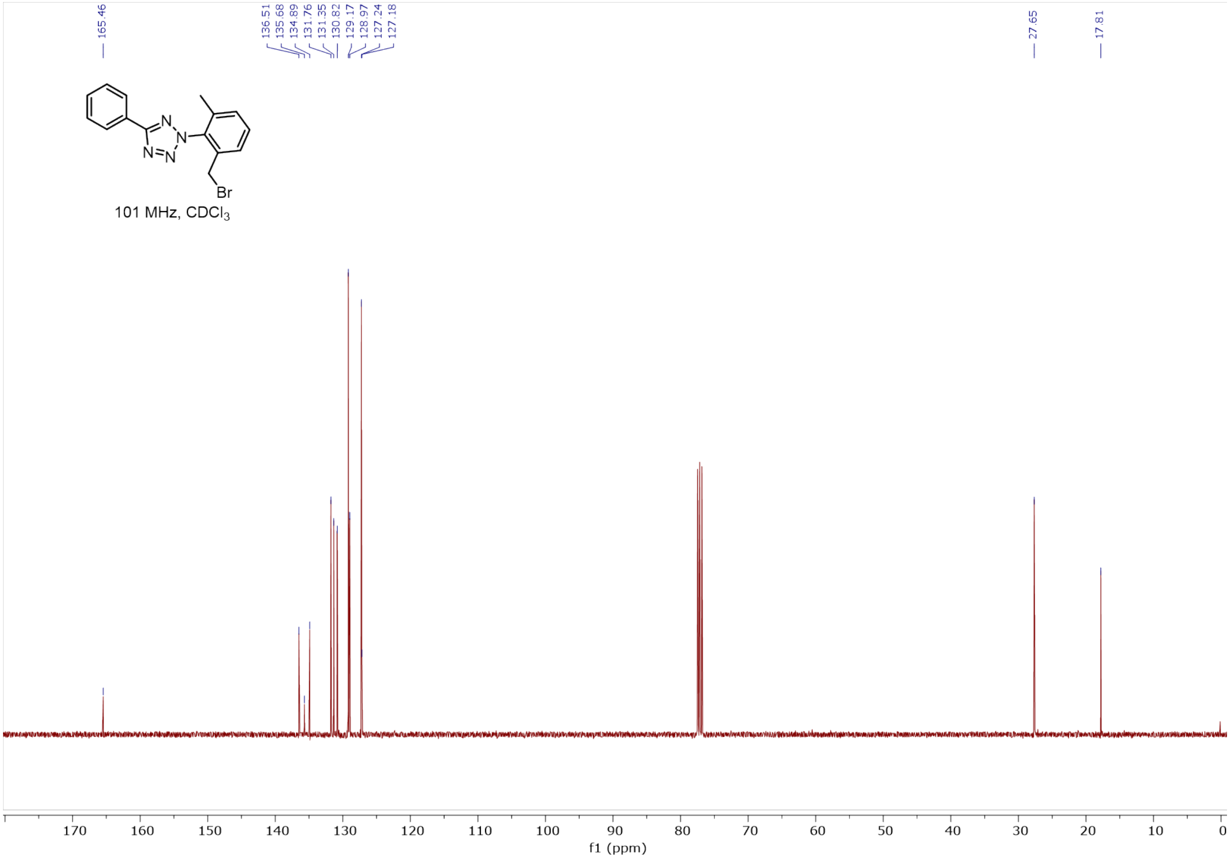

2-(2,6-Bis(bromomethyl)phenyl)-5-phenyl-2*H*-tetrazole (**2c**)

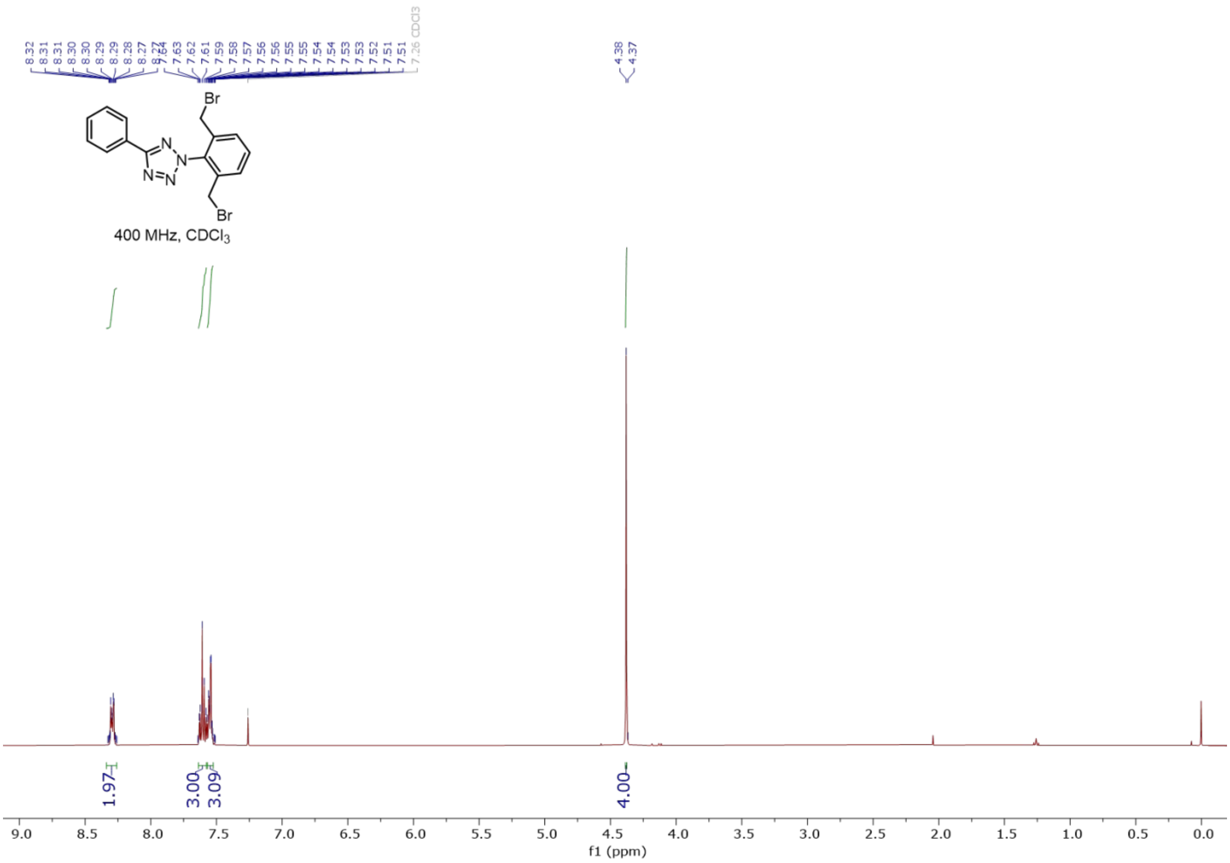

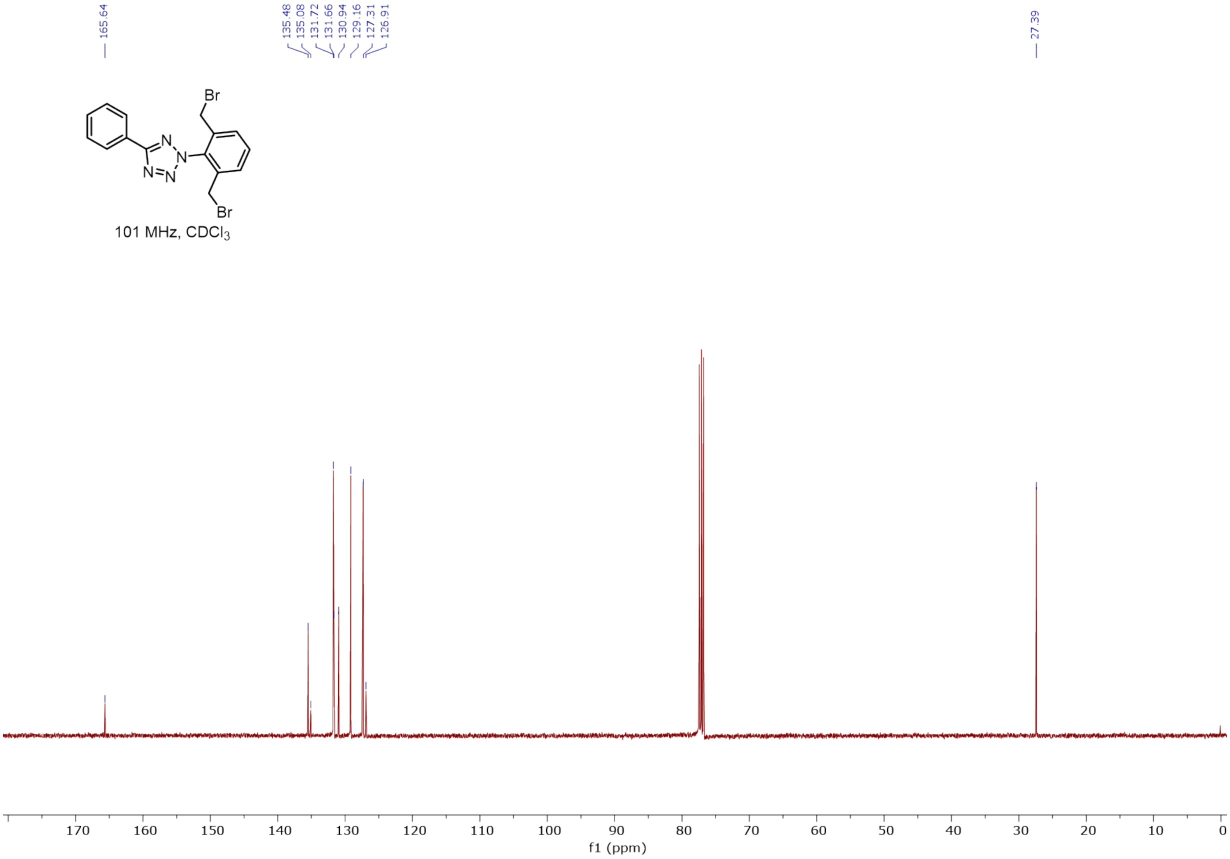

2-(2-(Bromomethyl)-5-fluorophenyl)-5-phenyl-2*H*-tetrazole (**2d**)

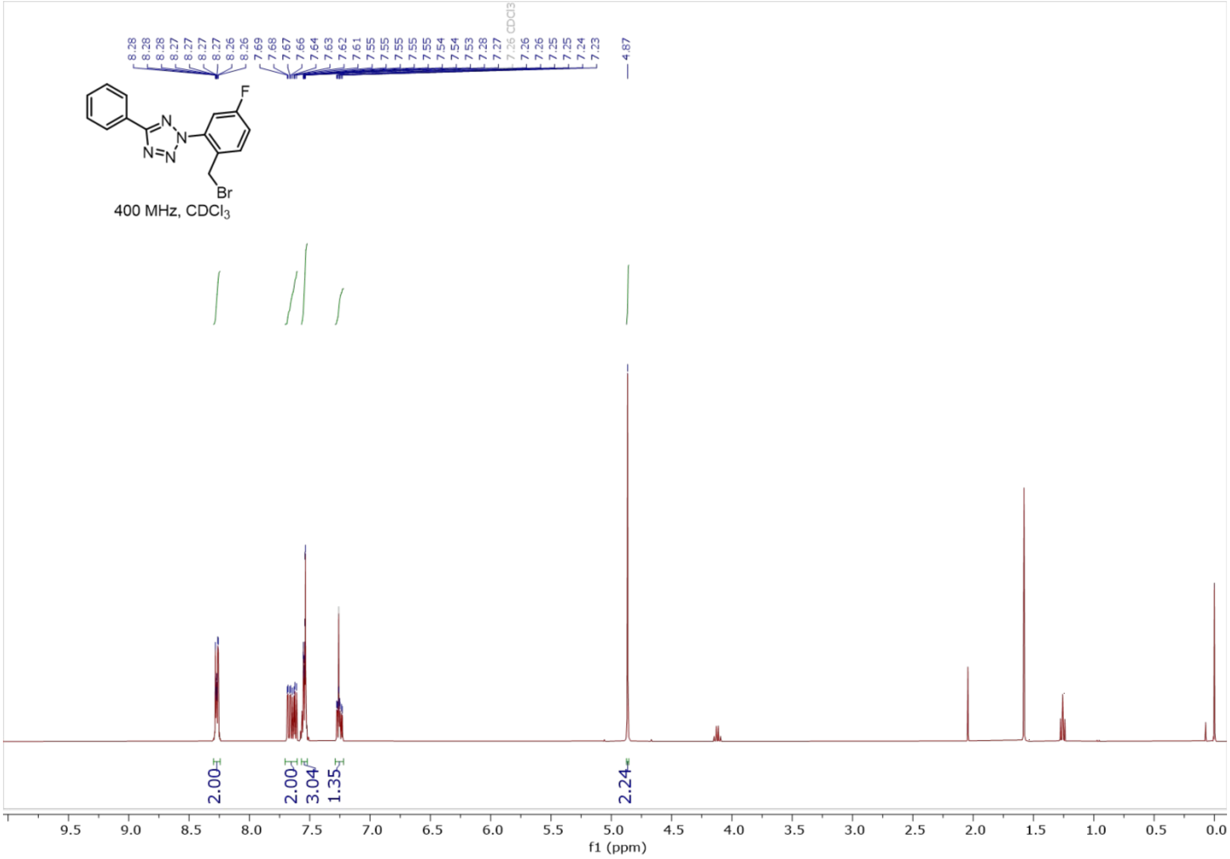

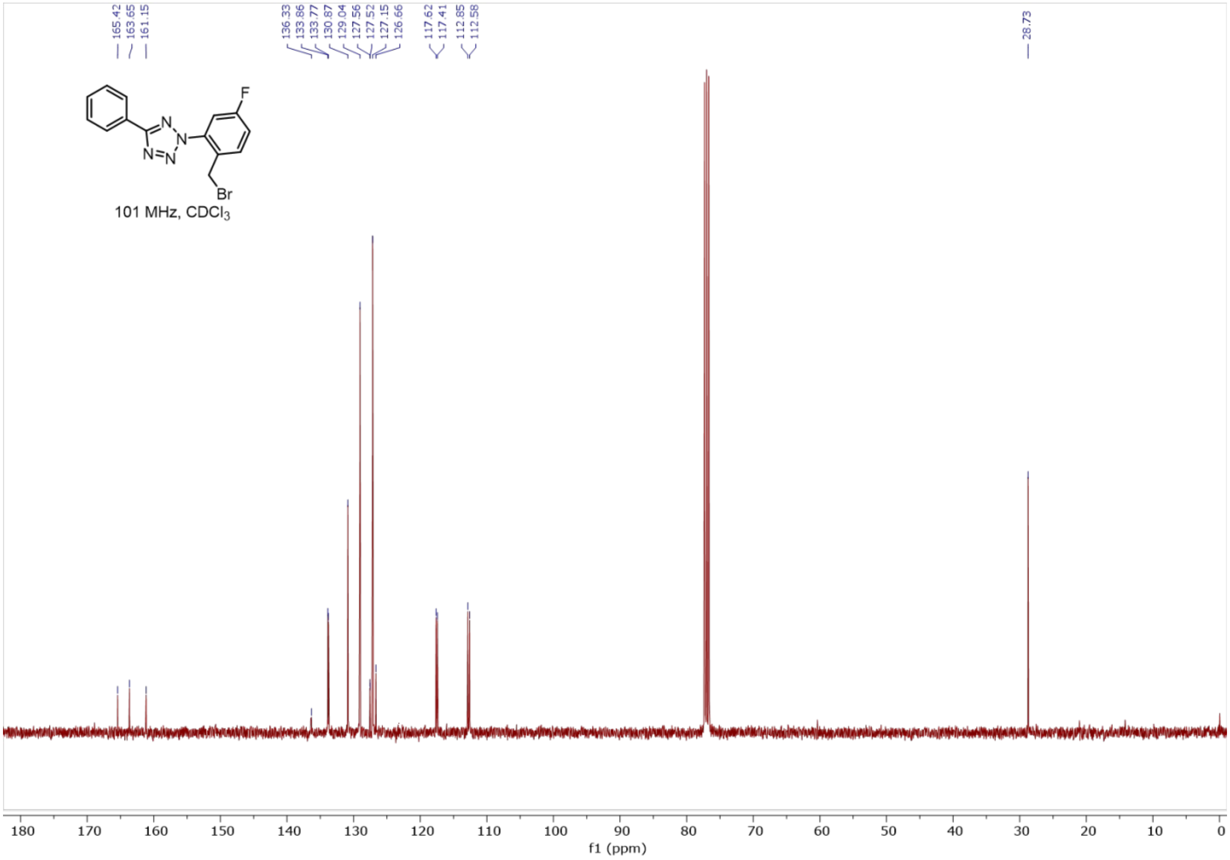
165.42
163.65
161.15
136.33
133.86
133.77
130.87
129.04
127.56
127.52
127.15
126.66
117.62
117.41
112.85
112.58
28.73
F
N
Br
101 MHz, CDCl3
f1 (ppm)

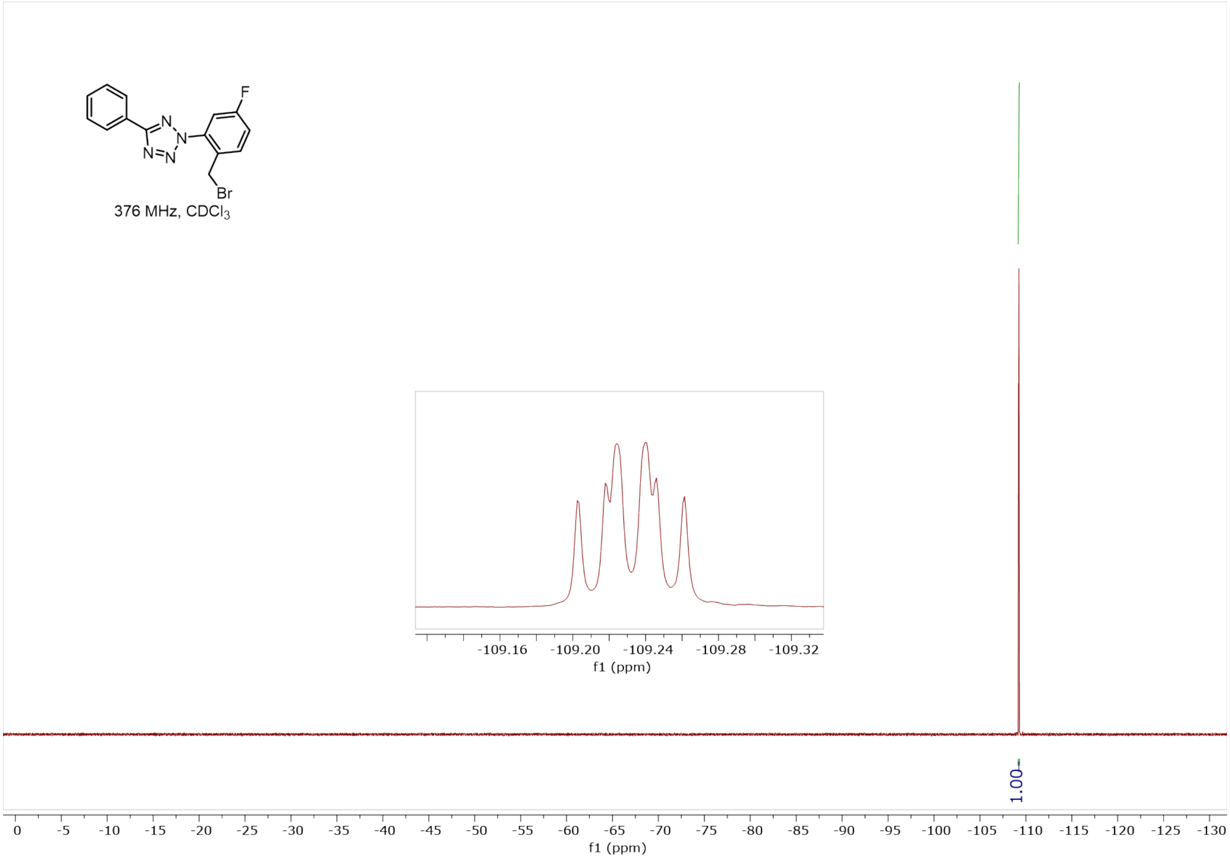
F
N
Br
376 MHz, CDCl3
-109.16
-109.20
-109.24
-109.28
-109.32
f1 (ppm)
1.00
f1 (ppm)

3-Phenylprop-2-yn-1-ol (**2e**)

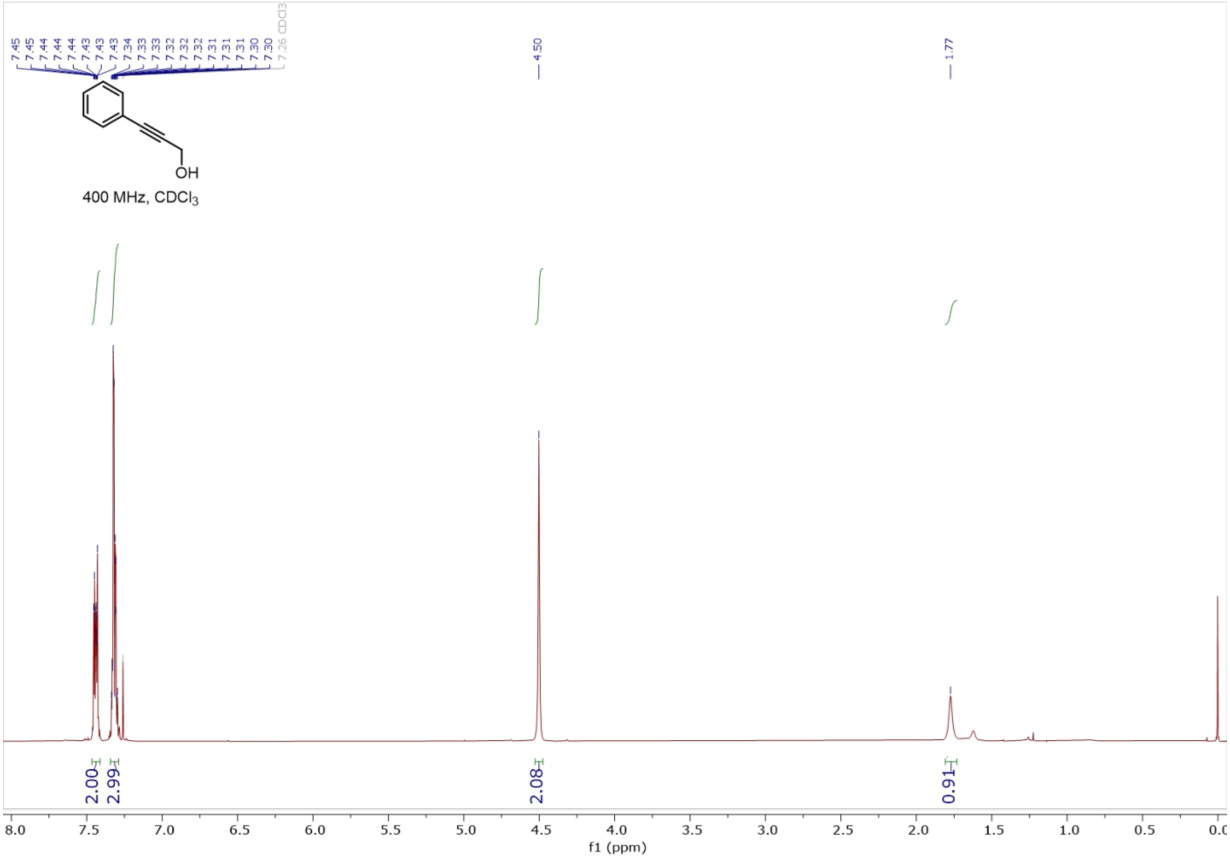

2-(3-Fluoro-2-((prop-2-yn-1-yloxy)methyl)phenyl)-5-phenyl-2*H*-tetrazole (**3a**)

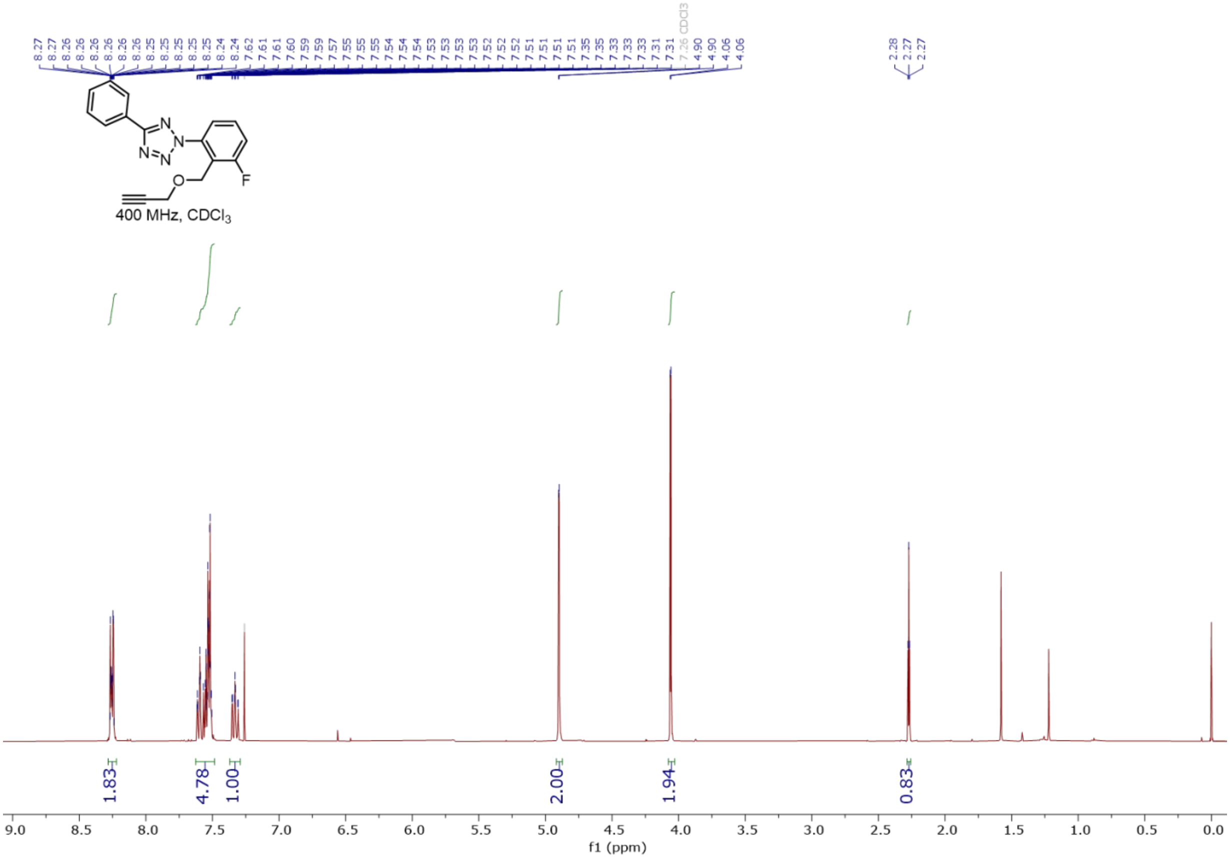

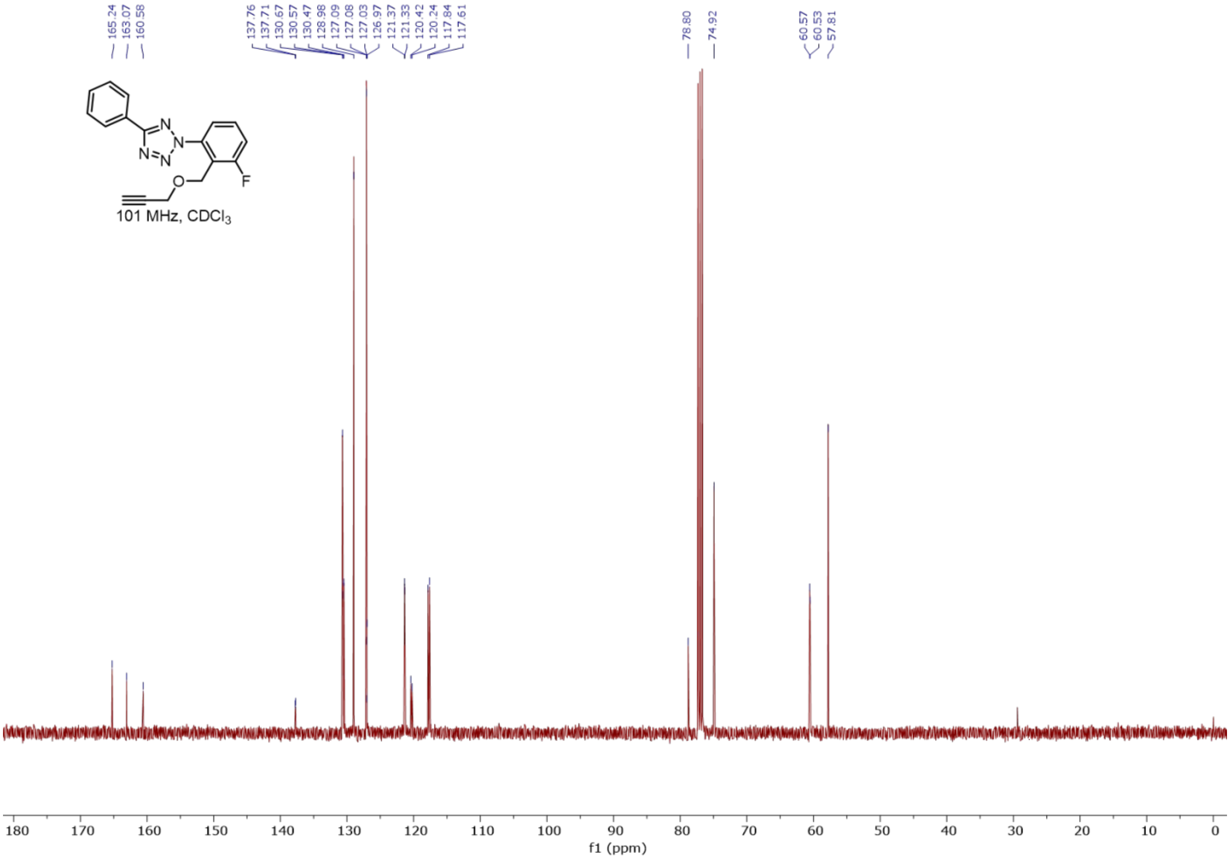
165.24
163.07
160.58
137.76
137.71
130.67
130.57
130.47
128.98
127.09
127.08
127.03
126.97
121.37
121.33
120.42
120.24
117.84
117.61
78.80
74.92
60.57
60.53
57.81
101 MHz, CDCl3
f1 (ppm)

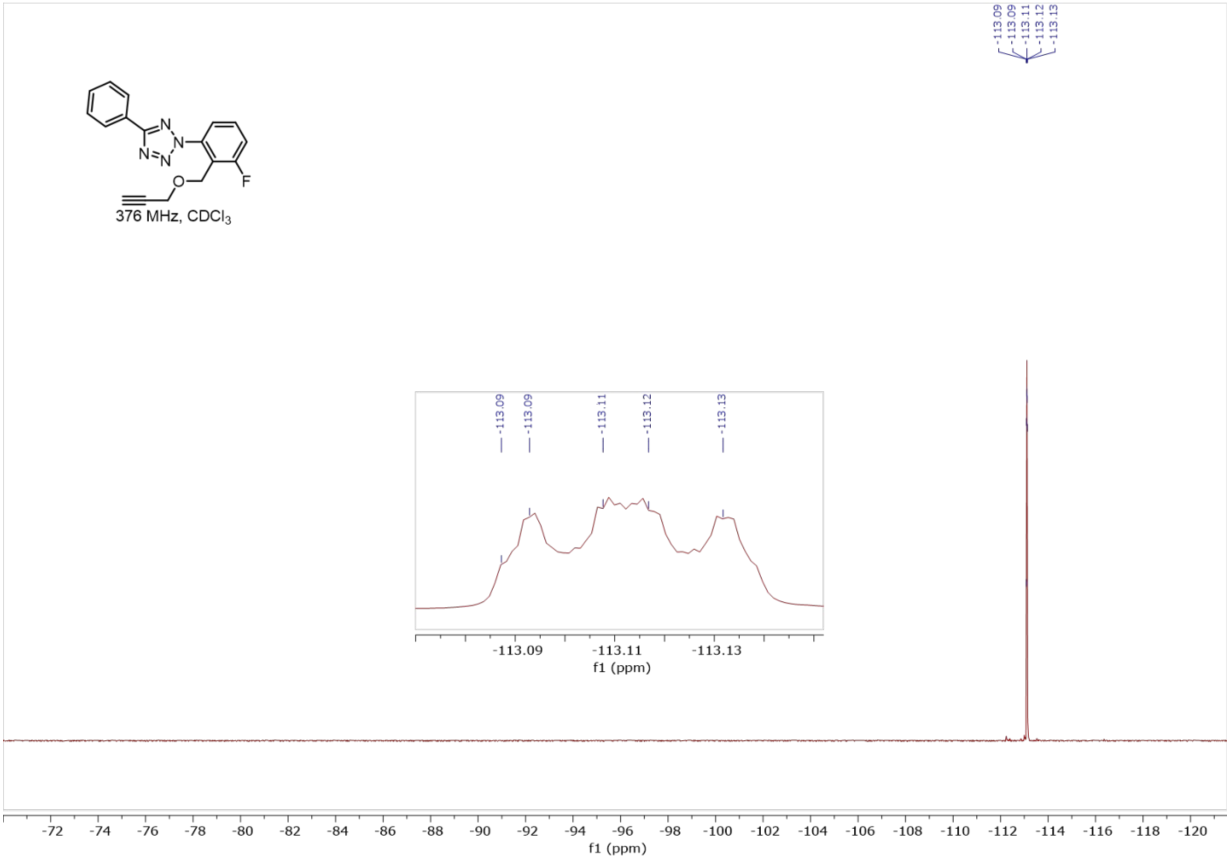
-113.09
-113.09
-113.11
-113.12
-113.13
376 MHz, CDCl3
f1 (ppm)

2-(2-Methyl-6-((prop-2-yn-1-yloxy)methyl)phenyl)-5-phenyl-2*H*-tetrazole (**3b**)

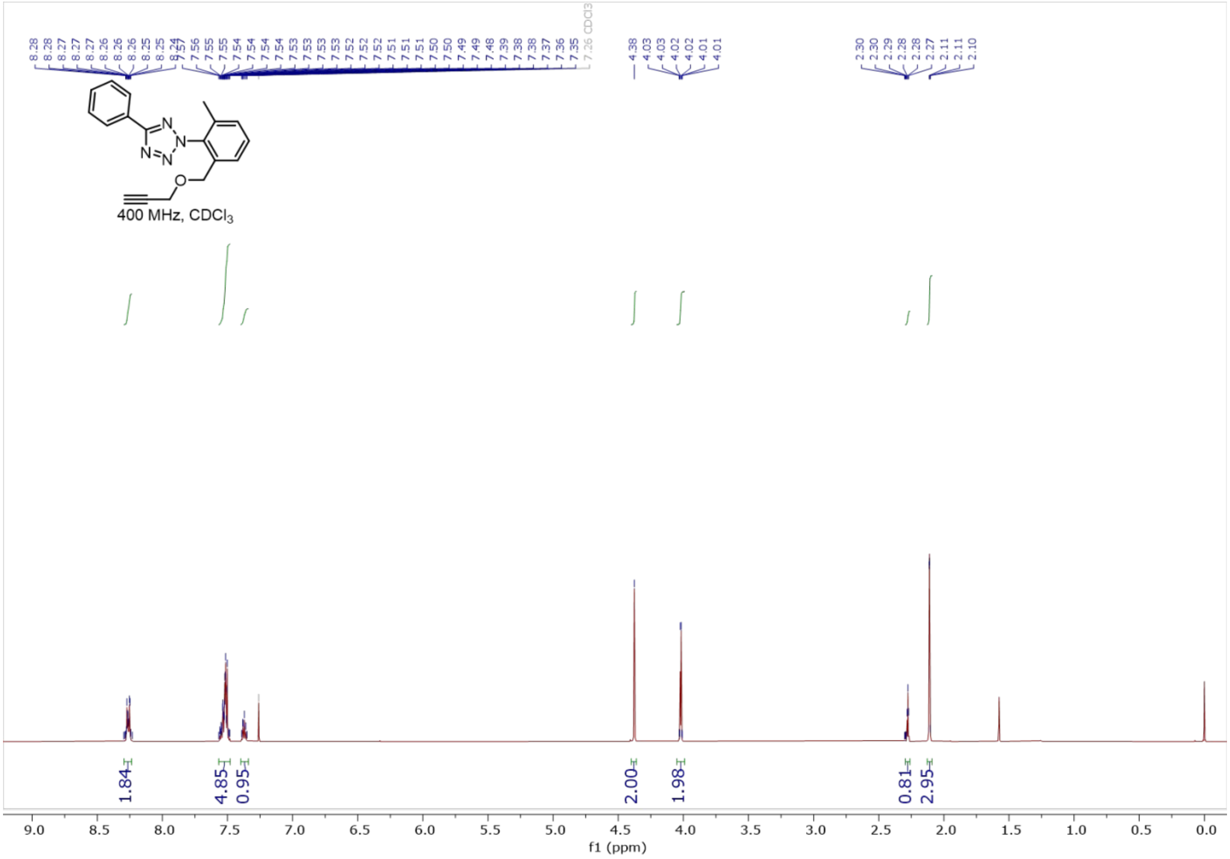

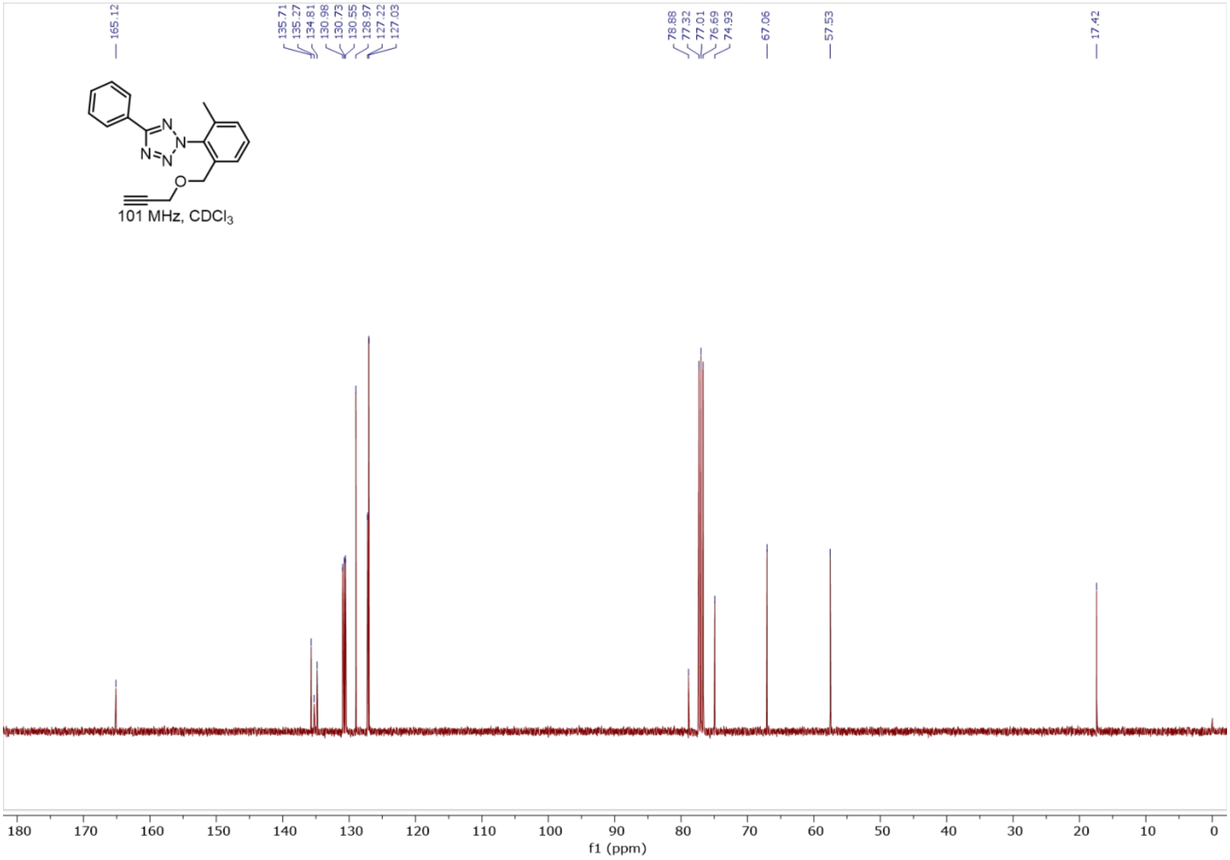

2-(5-Fluoro-2-((prop-2-yn-1-yloxy)methyl)phenyl)-5-phenyl-2*H*-tetrazole (**3c**)

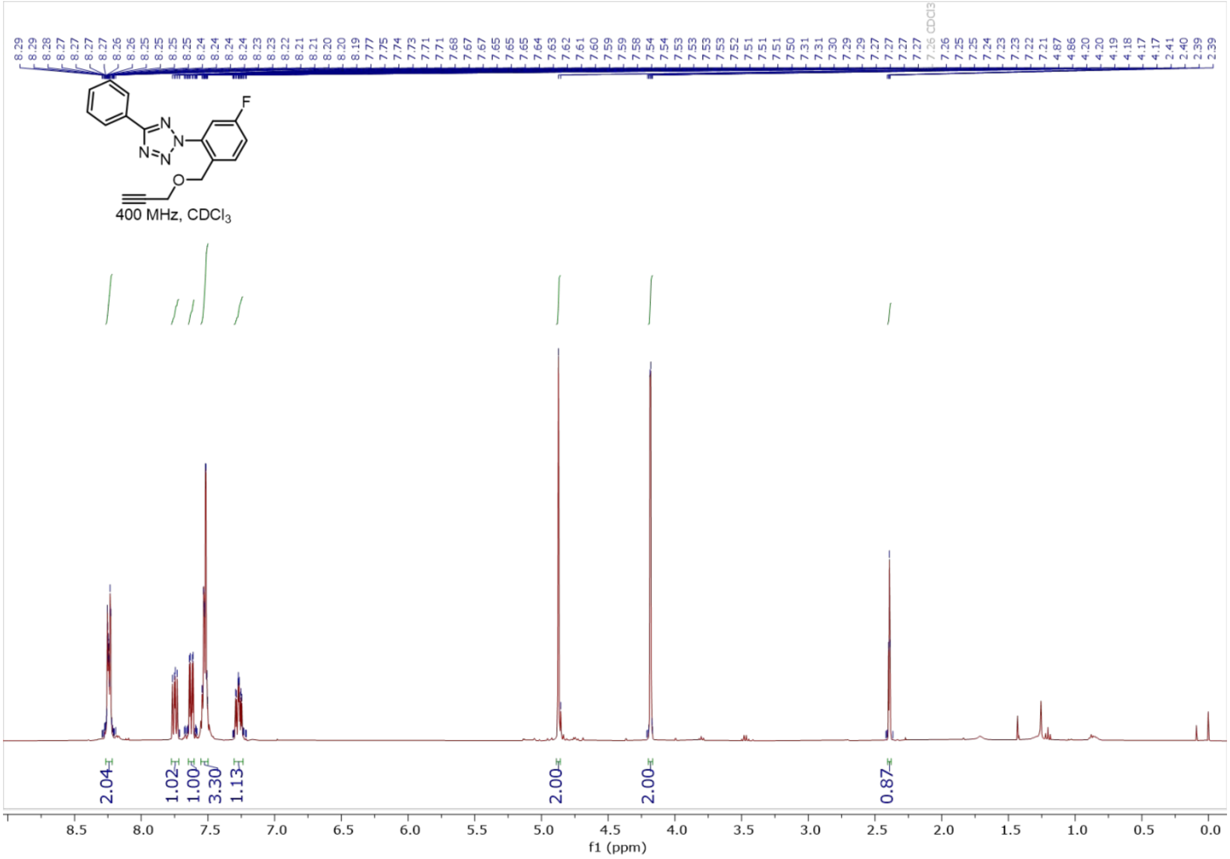

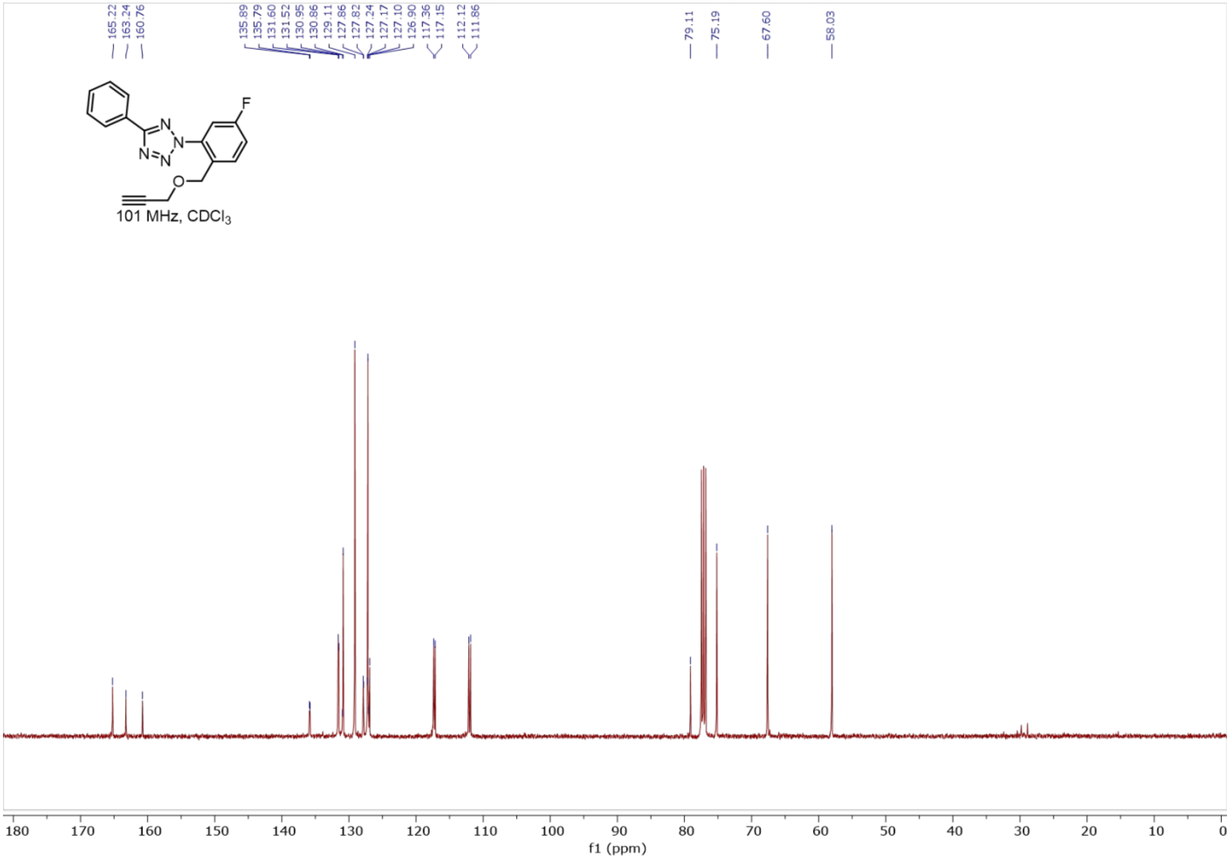

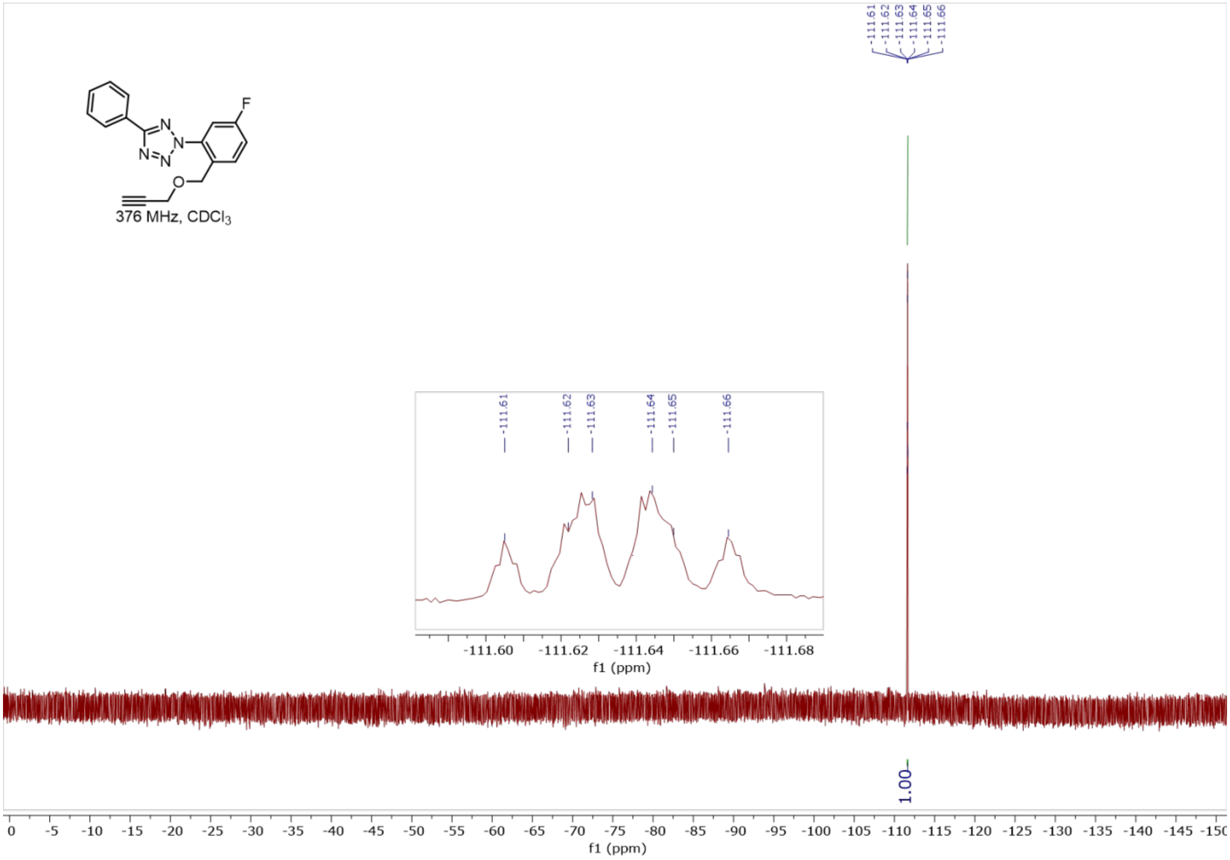

2-(3-Fluoro-2-(((3-phenylprop-2-yn-1-yl)oxy)methyl)phenyl)-5-phenyl-2*H*-tetrazole (**3d**)

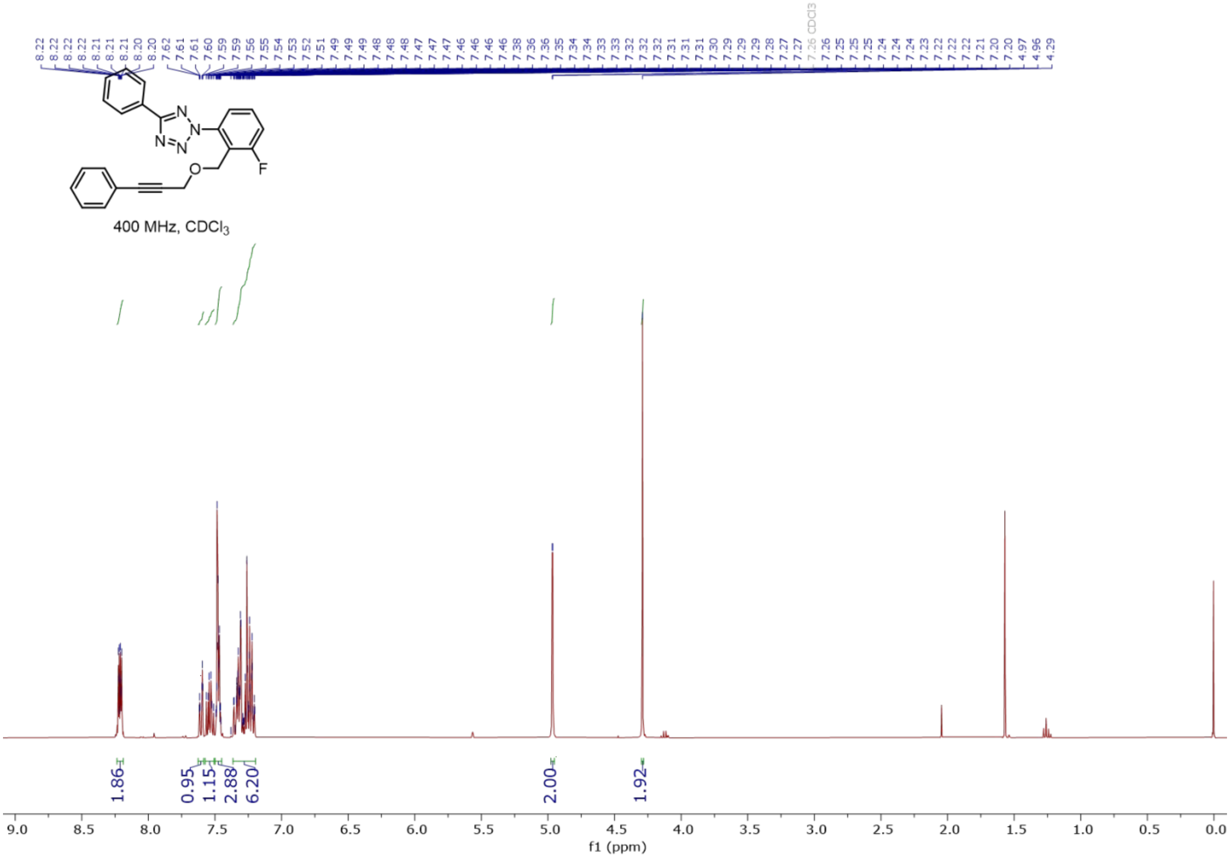

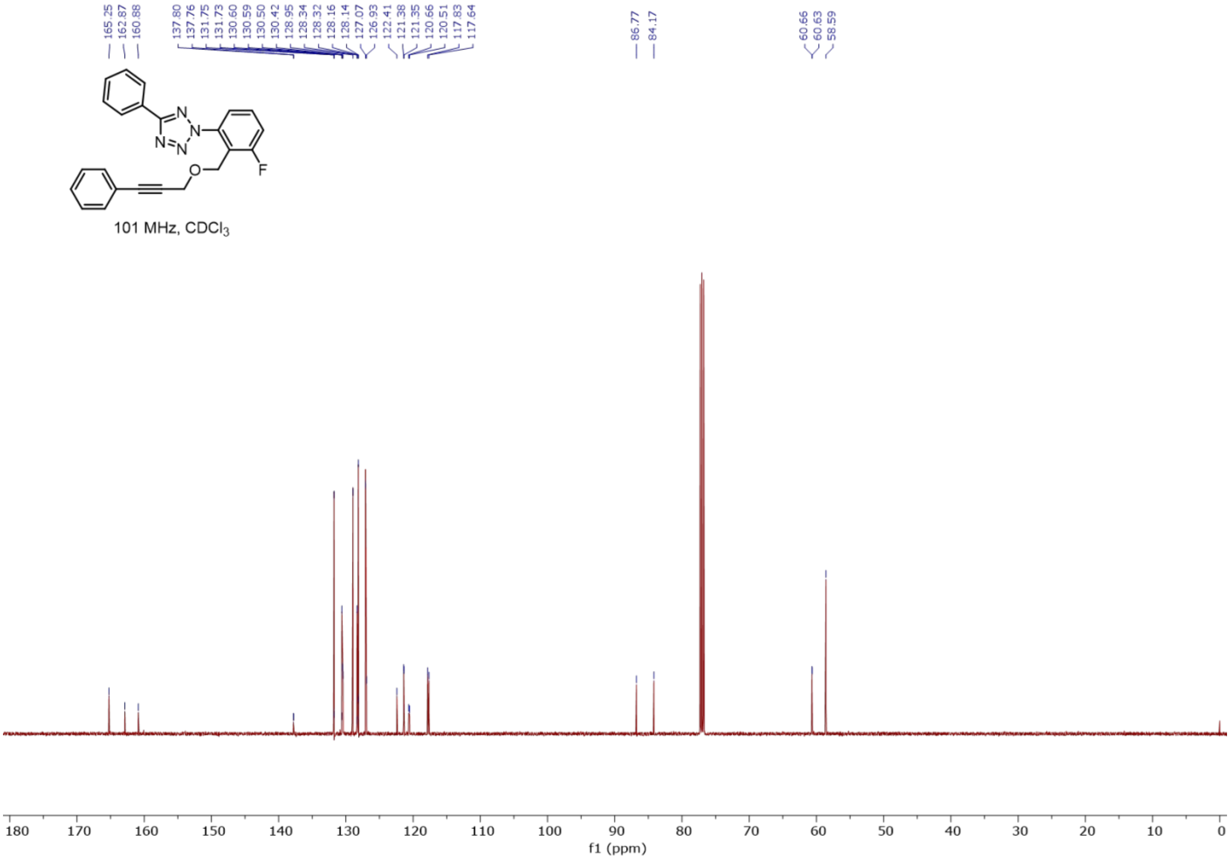
165.25
162.87
160.88
137.80
137.76
131.75
131.73
130.60
130.59
130.50
130.42
128.95
128.34
128.32
128.16
128.14
127.07
126.93
122.41
121.38
121.35
120.66
120.51
117.83
117.64
86.77
84.17
60.66
60.63
58.59
101 MHz, CDCl3
f1 (ppm)

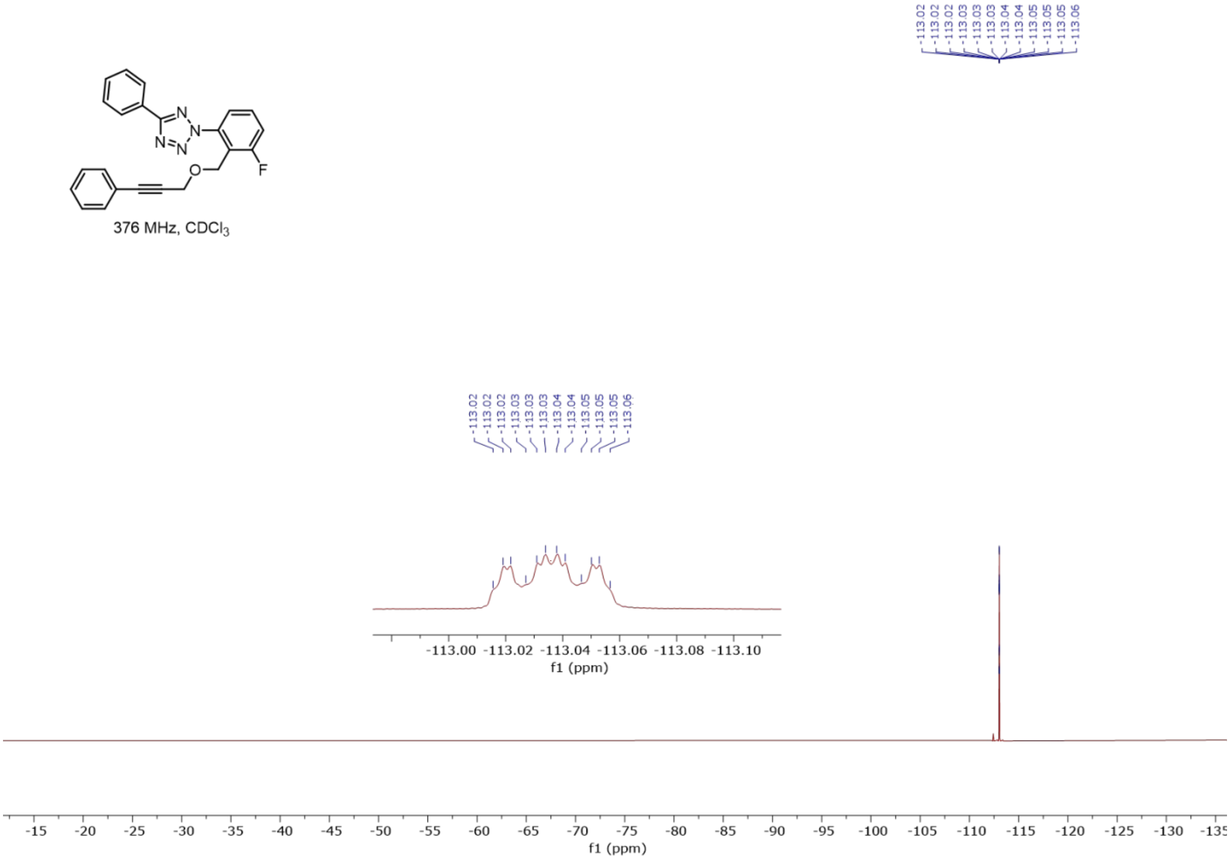
-113.02
-113.02
-113.02
-113.03
-113.03
-113.03
-113.04
-113.04
-113.05
-113.05
-113.06
376 MHz, CDCl3
f1 (ppm)

2-(2-((But-3-en-1-yloxy)methyl)-3-fluorophenyl)-5-phenyl-2*H*-tetrazole (**3e**)

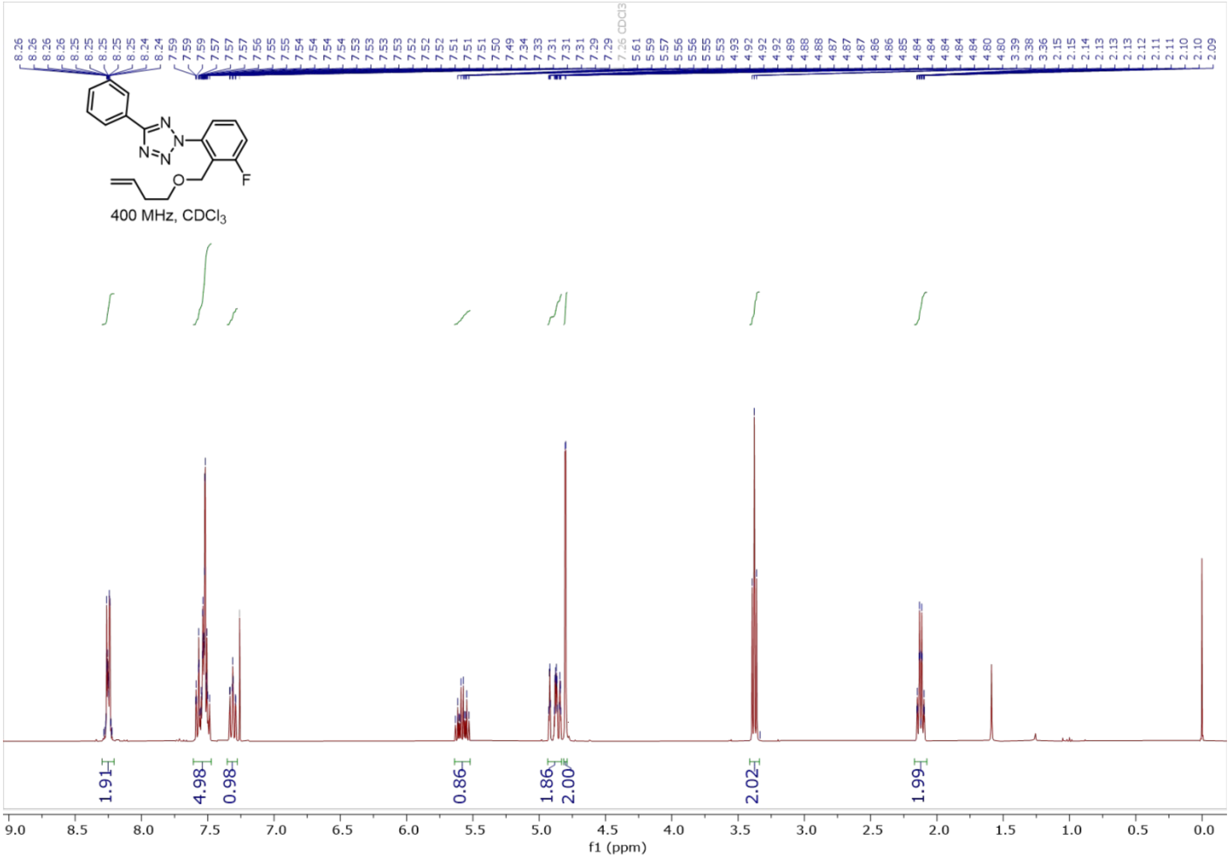

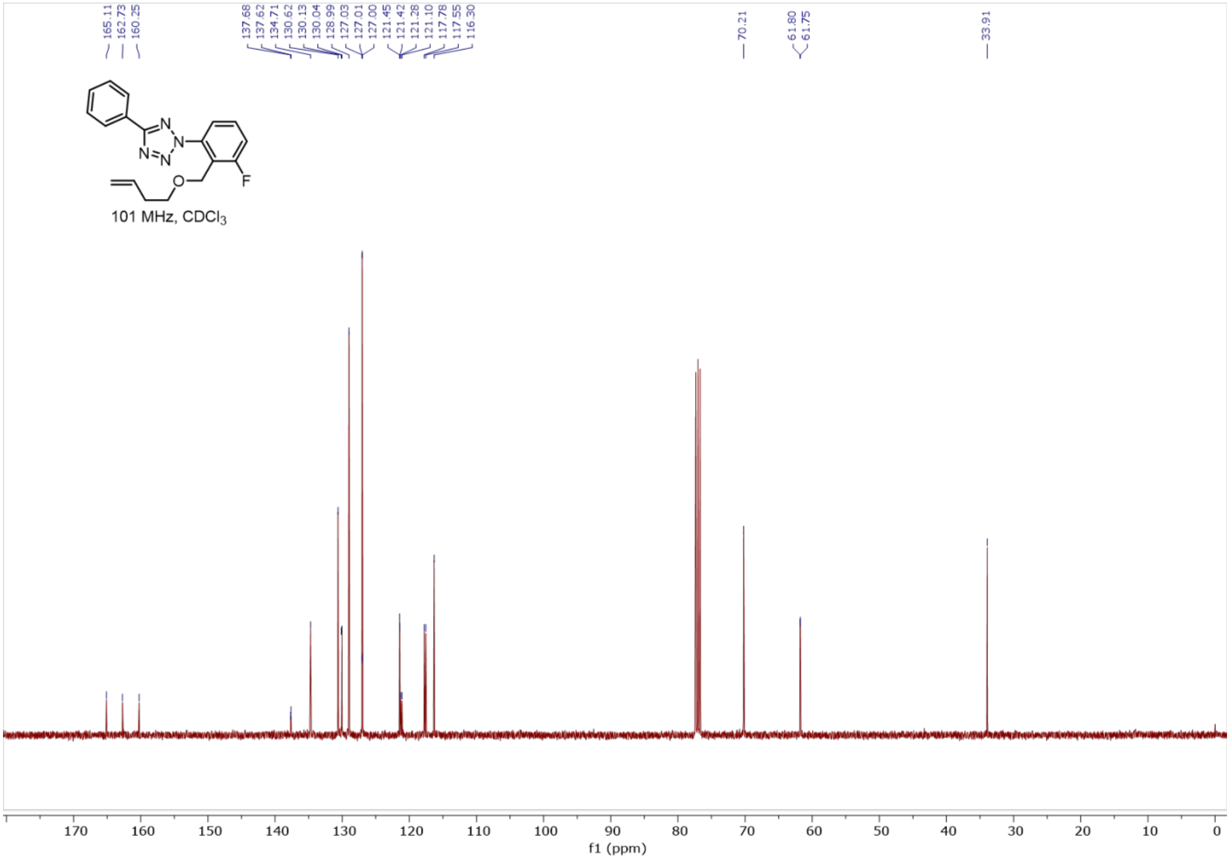

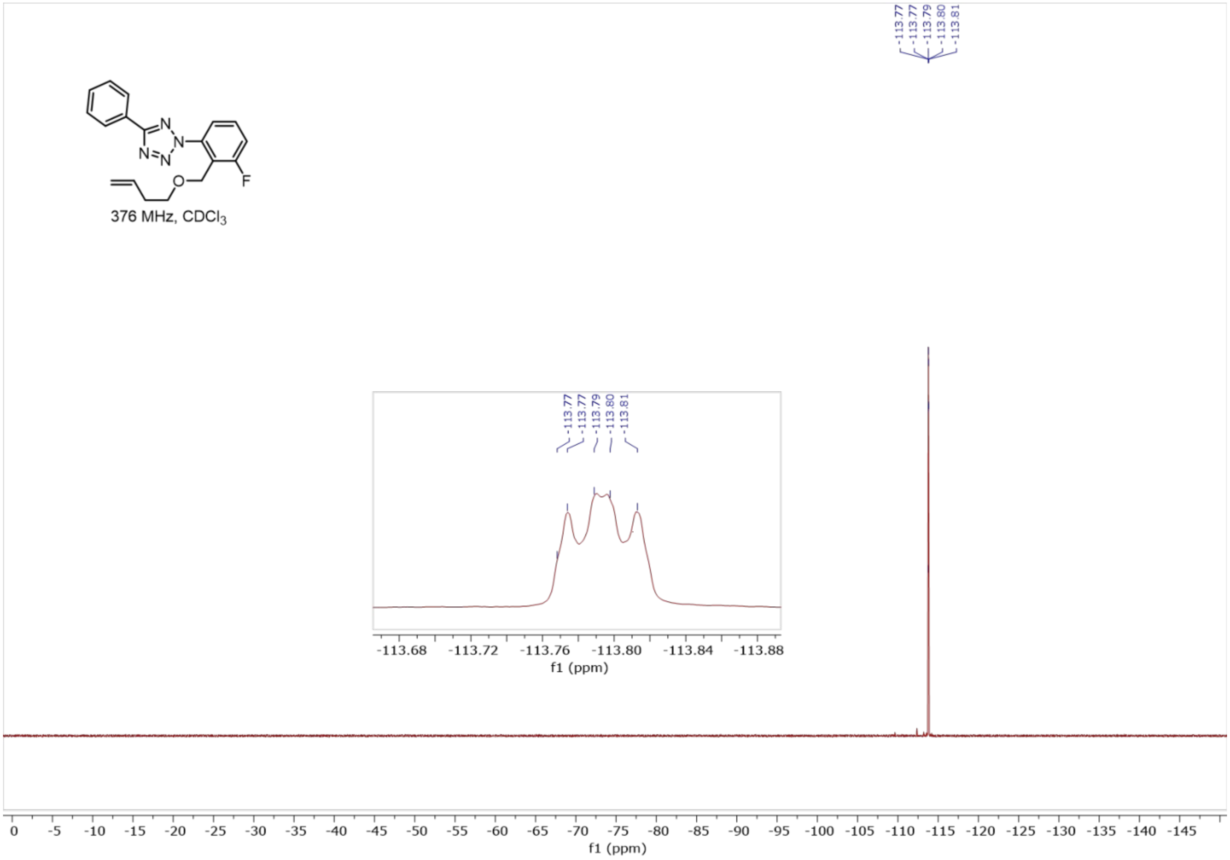

2-(2-((Allyloxy)methyl)-3-fluorophenyl)-5-phenyl-2*H*-tetrazole (**3f**)

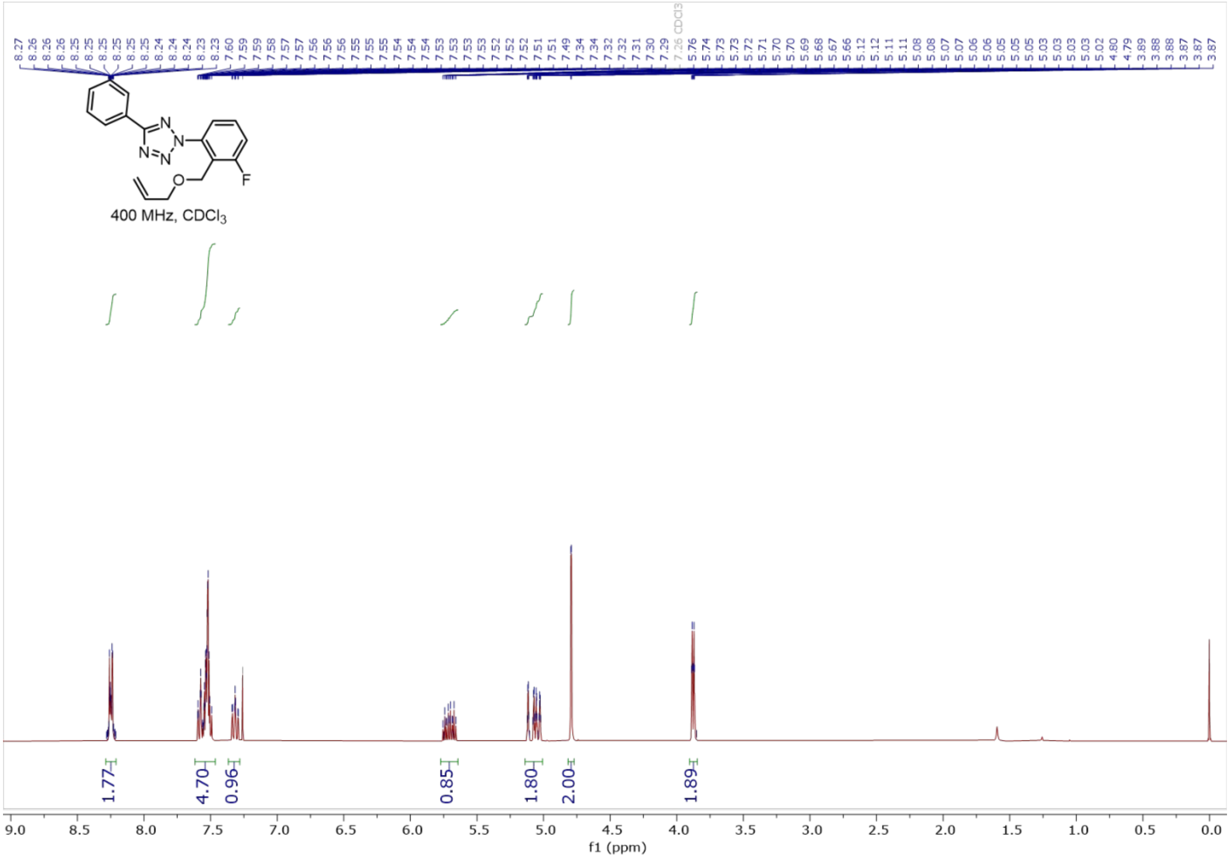

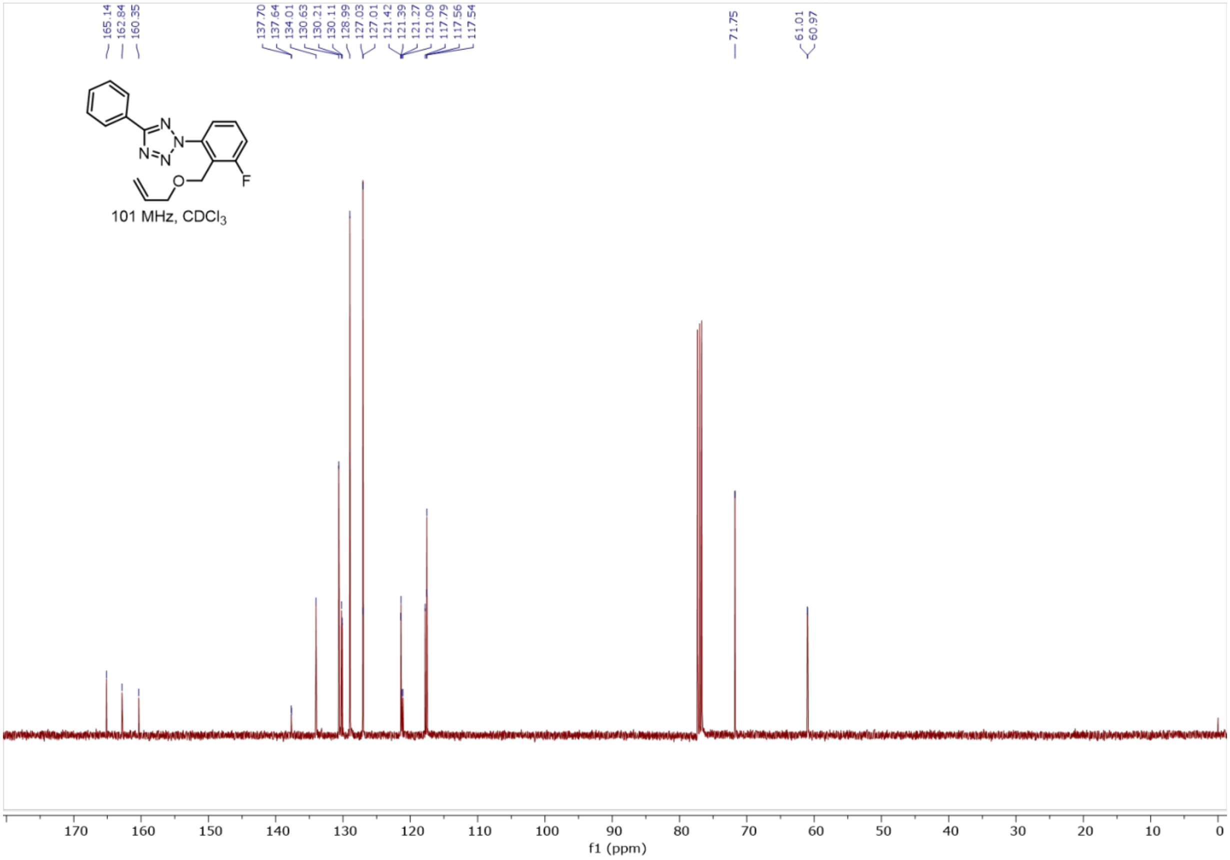
165.14
162.84
160.35
137.70
137.64
134.01
130.63
130.21
130.11
128.99
127.03
127.01
121.42
121.39
121.27
121.09
117.79
117.56
117.54
71.75
61.01
60.97
101 MHz, CDCl3
170 160 150 140 130 120 110 100 90 80 70 60 50 40 30 20 10 0
f1 (ppm)

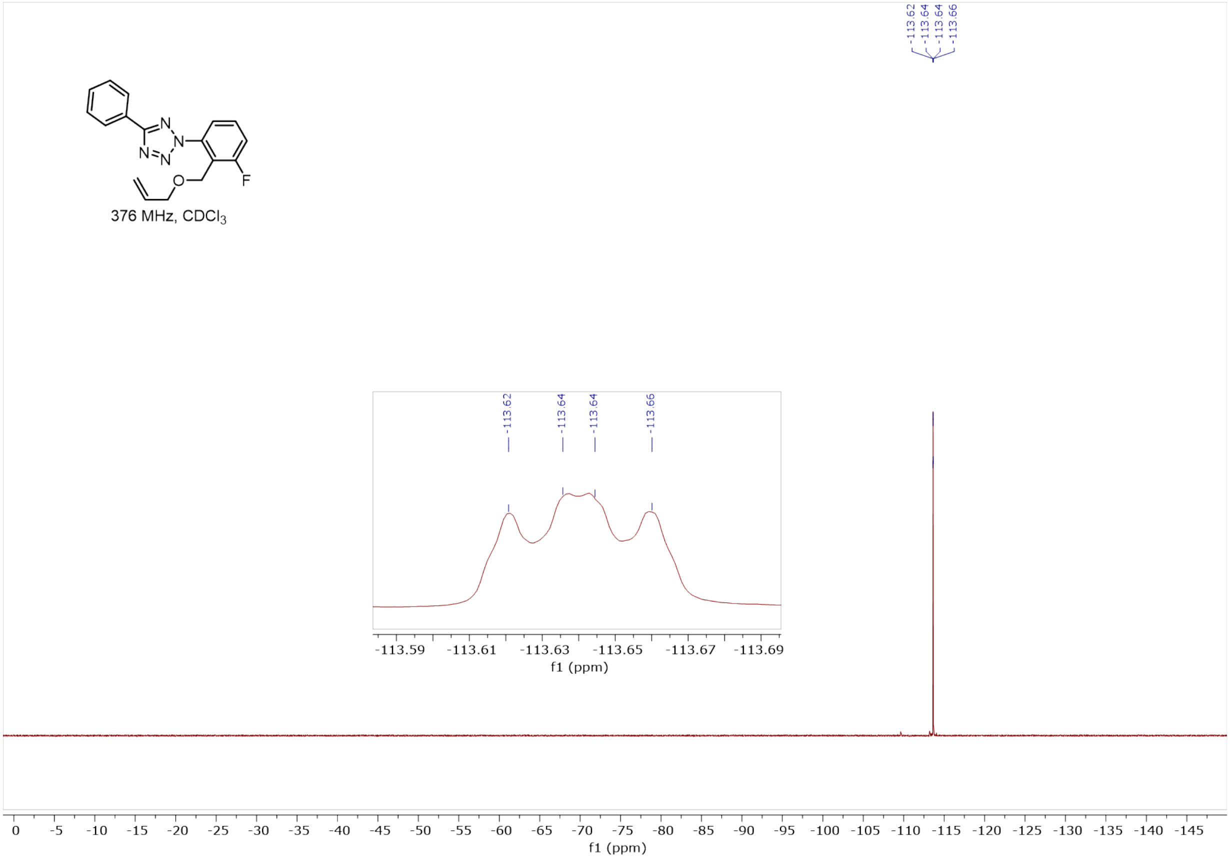
-113.62
-113.64
-113.64
-113.66
376 MHz, CDCl3
-113.59 -113.61 -113.63 -113.65 -113.67 -113.69
f1 (ppm)
0 -5 -10 -15 -20 -25 -30 -35 -40 -45 -50 -55 -60 -65 -70 -75 -80 -85 -90 -95 -100 -105 -110 -115 -120 -125 -130 -135 -140 -145
f1 (ppm)

N-(2-Fluoro-6-(5-phenyl-2*H*-tetrazol-2-yl)benzyl)prop-2-en-1-amine (**3g**)

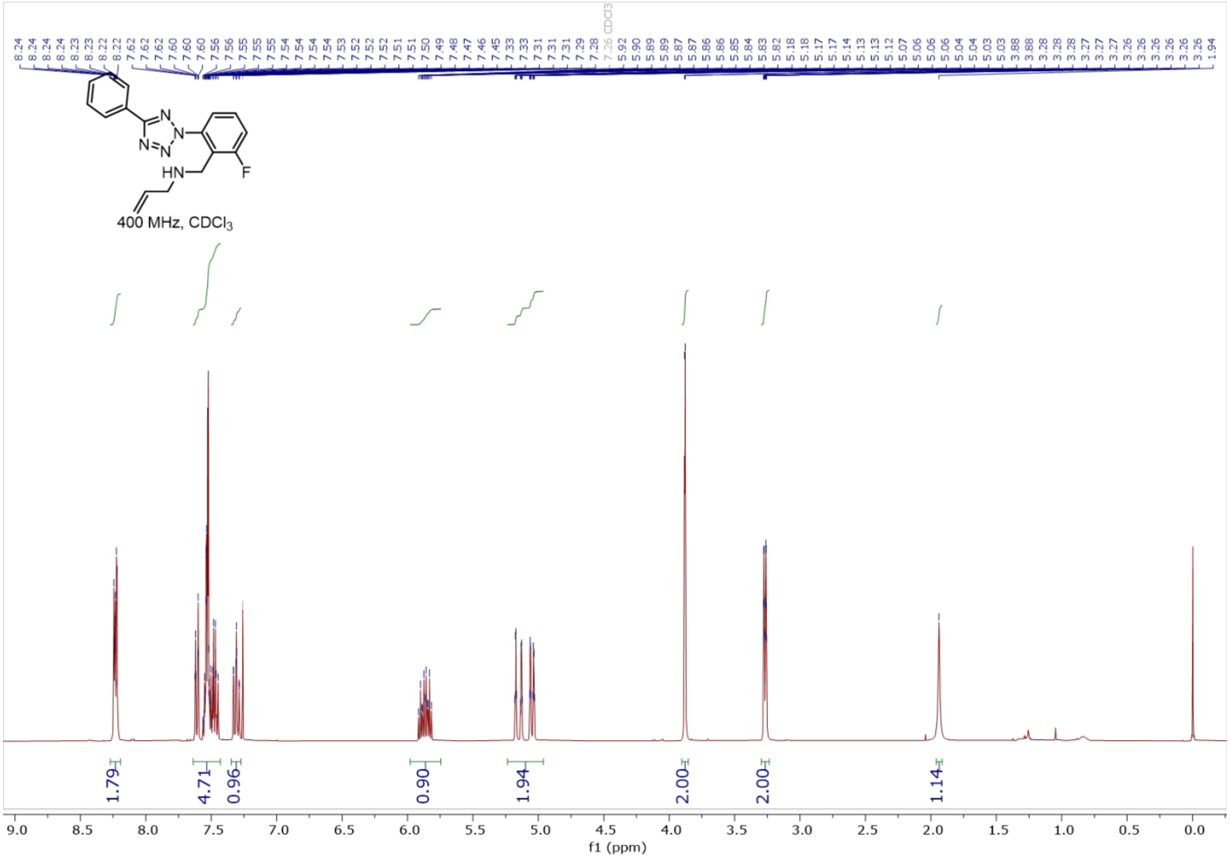

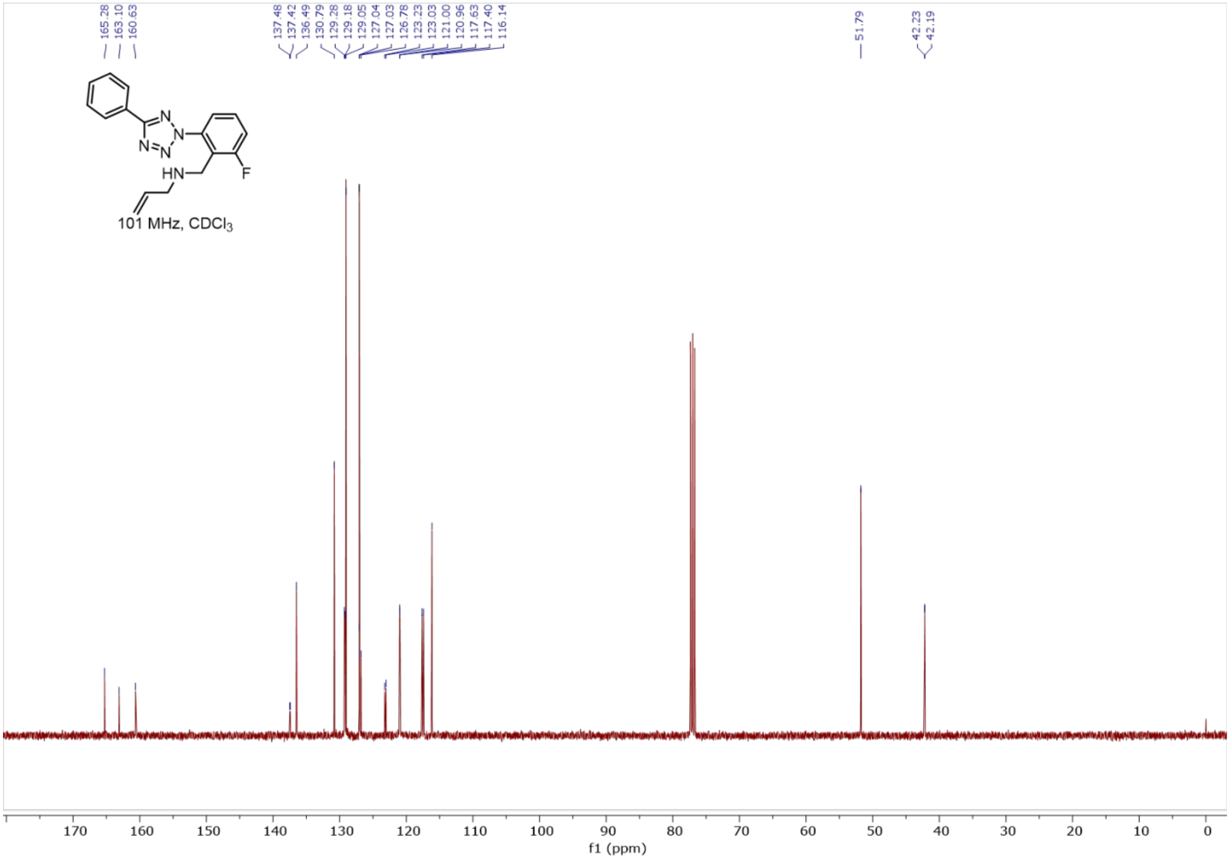

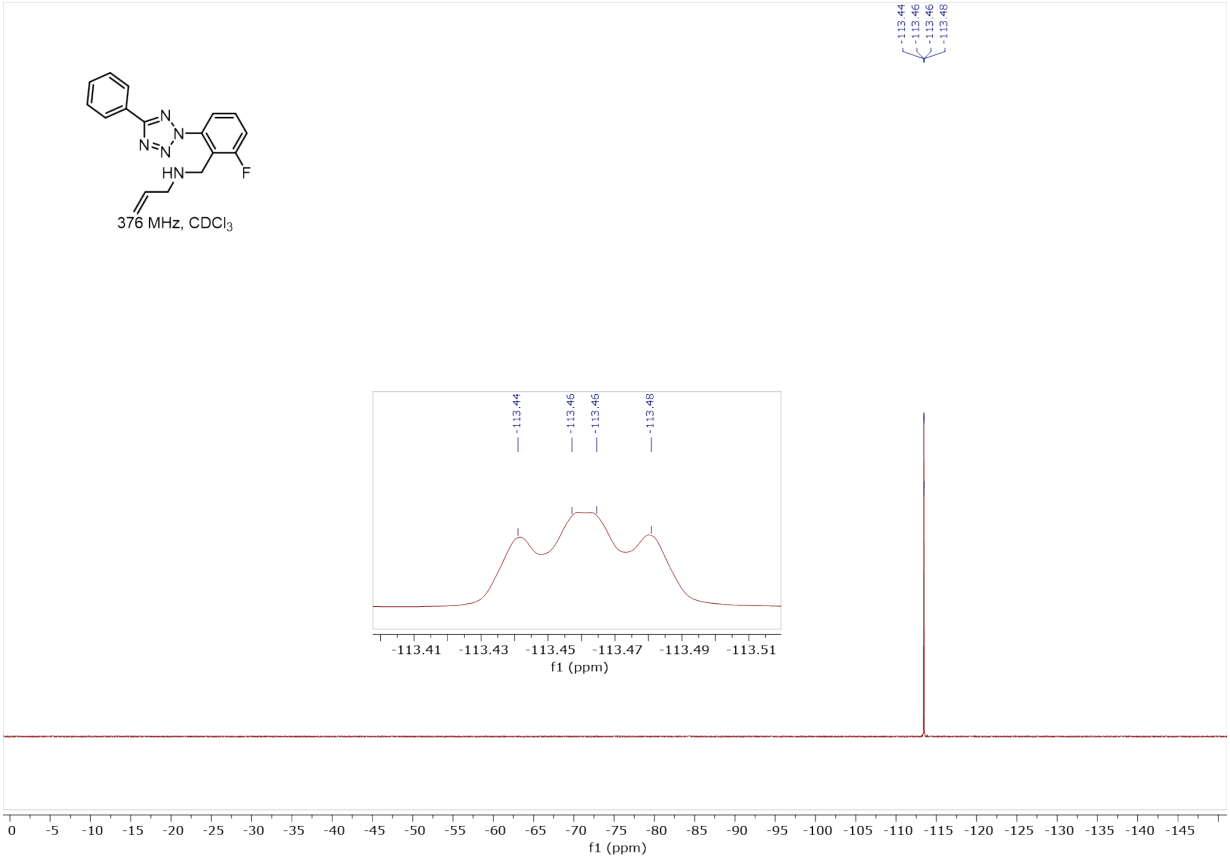

2-(2-((Cinnamyloxy)methyl)-3-fluorophenyl)-5-phenyl-2*H*-tetrazole (**3h**)

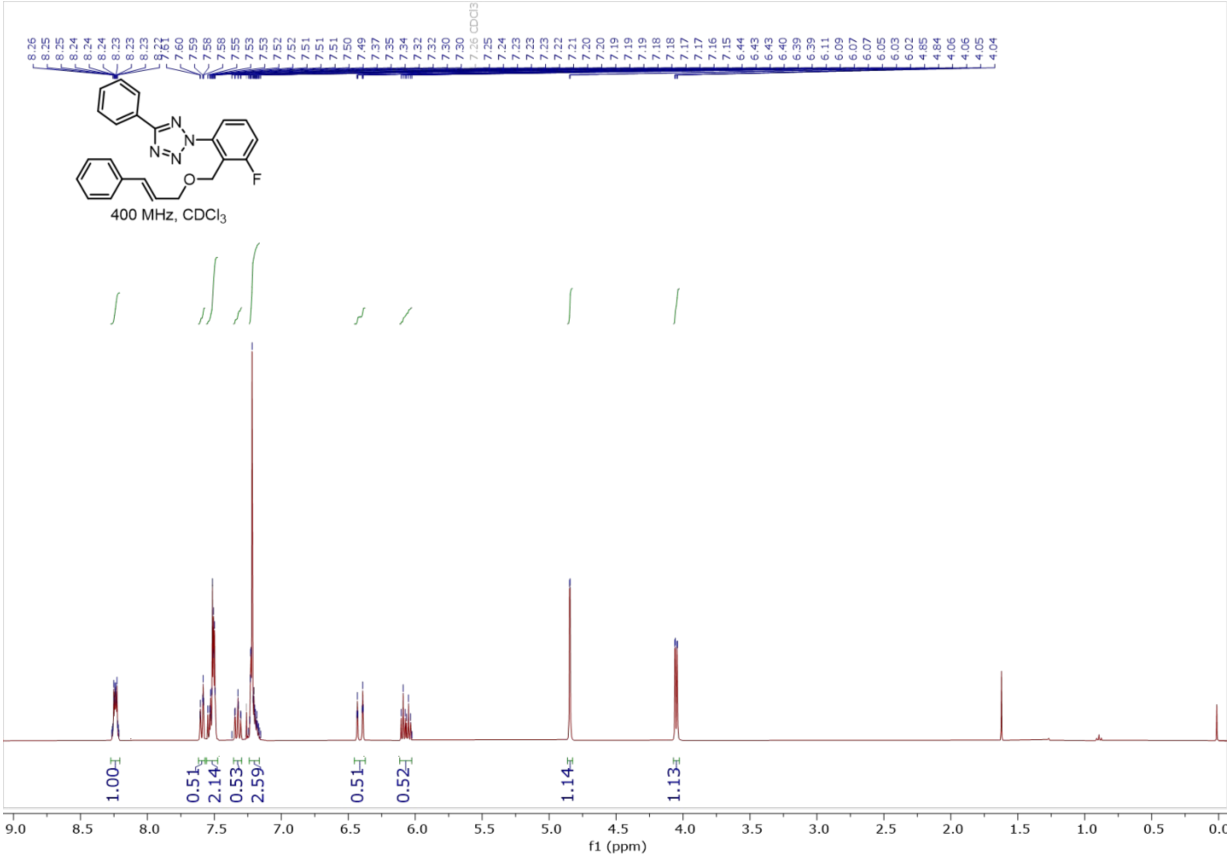

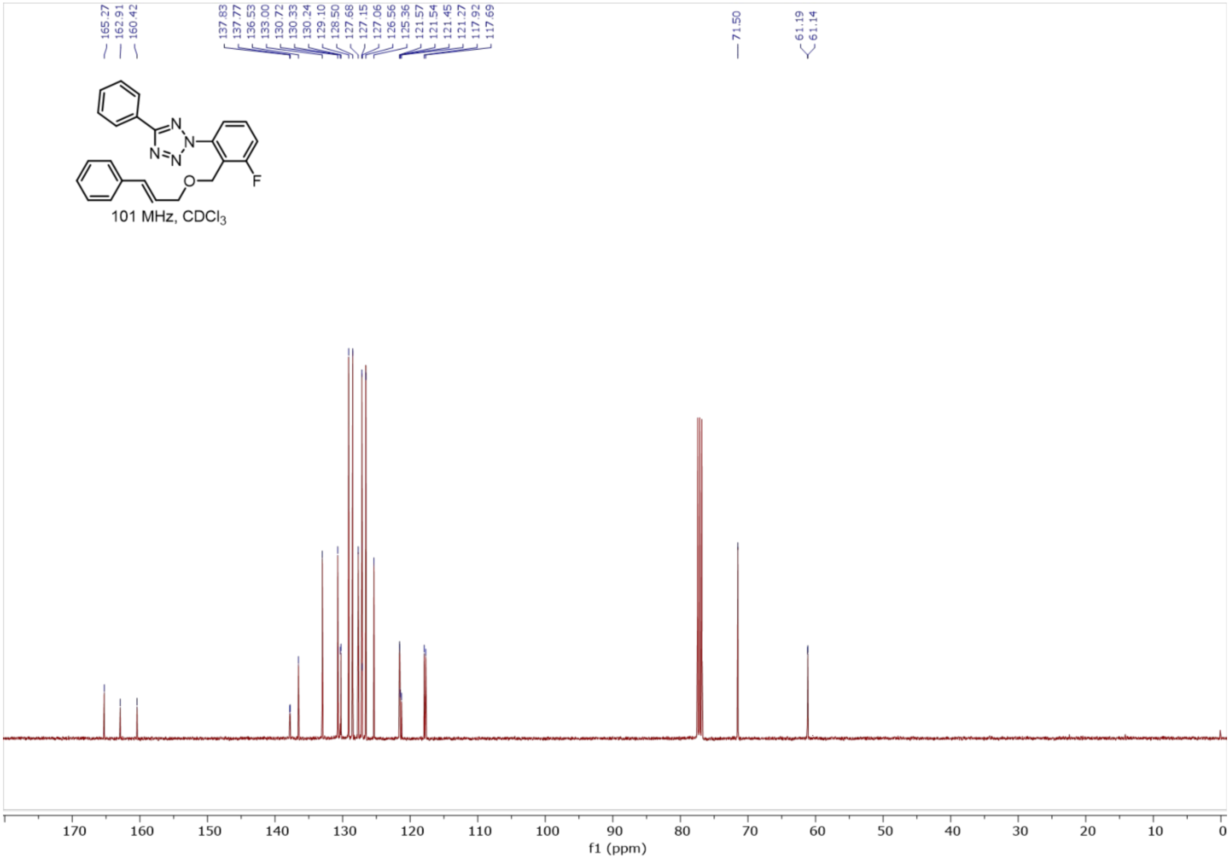
165.27
162.91
160.42
137.83
137.77
136.53
133.00
130.72
130.33
130.24
129.10
128.50
127.68
127.15
127.06
126.56
125.36
121.57
121.54
121.45
121.27
117.92
117.69
71.50
61.19
61.14
101 MHz, CDCl3
f1 (ppm)

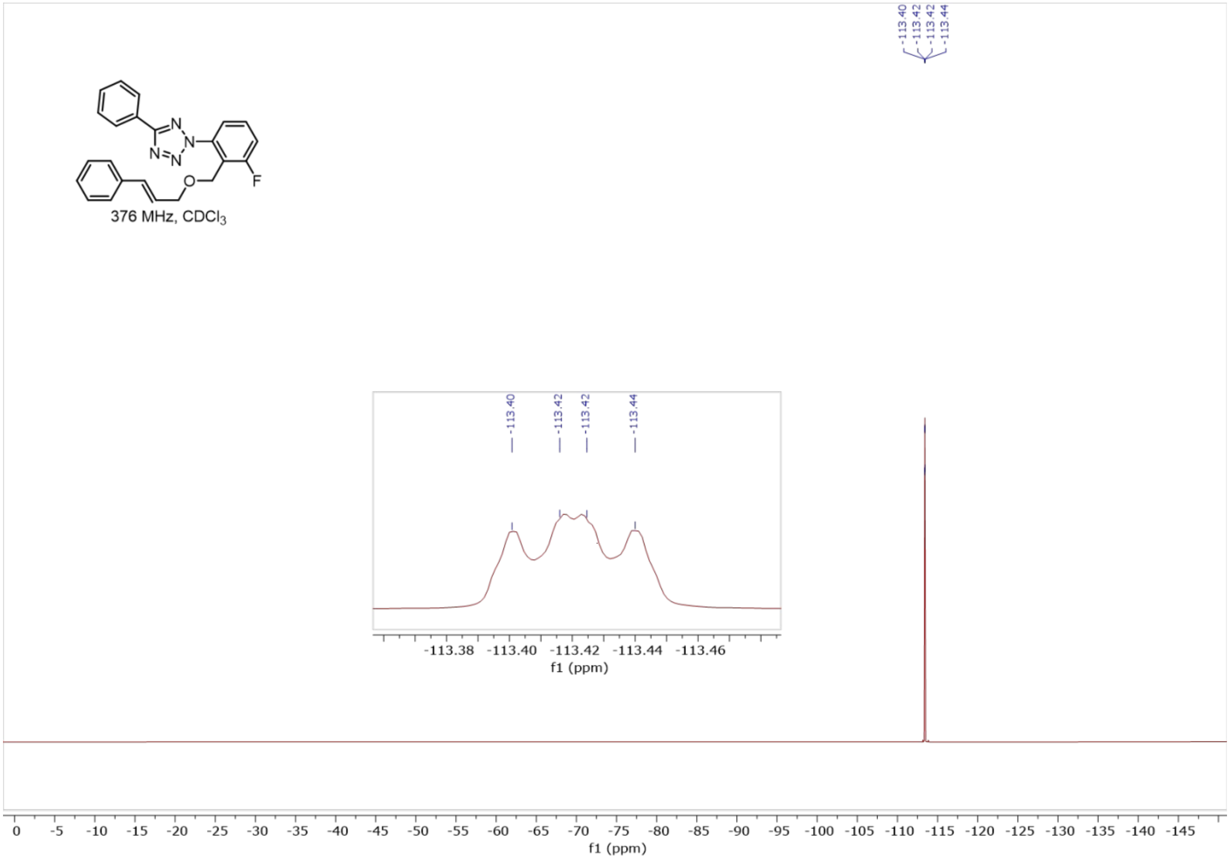
-113.40
-113.42
-113.42
-113.44
376 MHz, CDCl3
f1 (ppm)

N-(2-Fluoro-6-(5-phenyl-2*H*-tetrazol-2-yl)benzyl)-2-methylpropan-1-amine (**3i**)

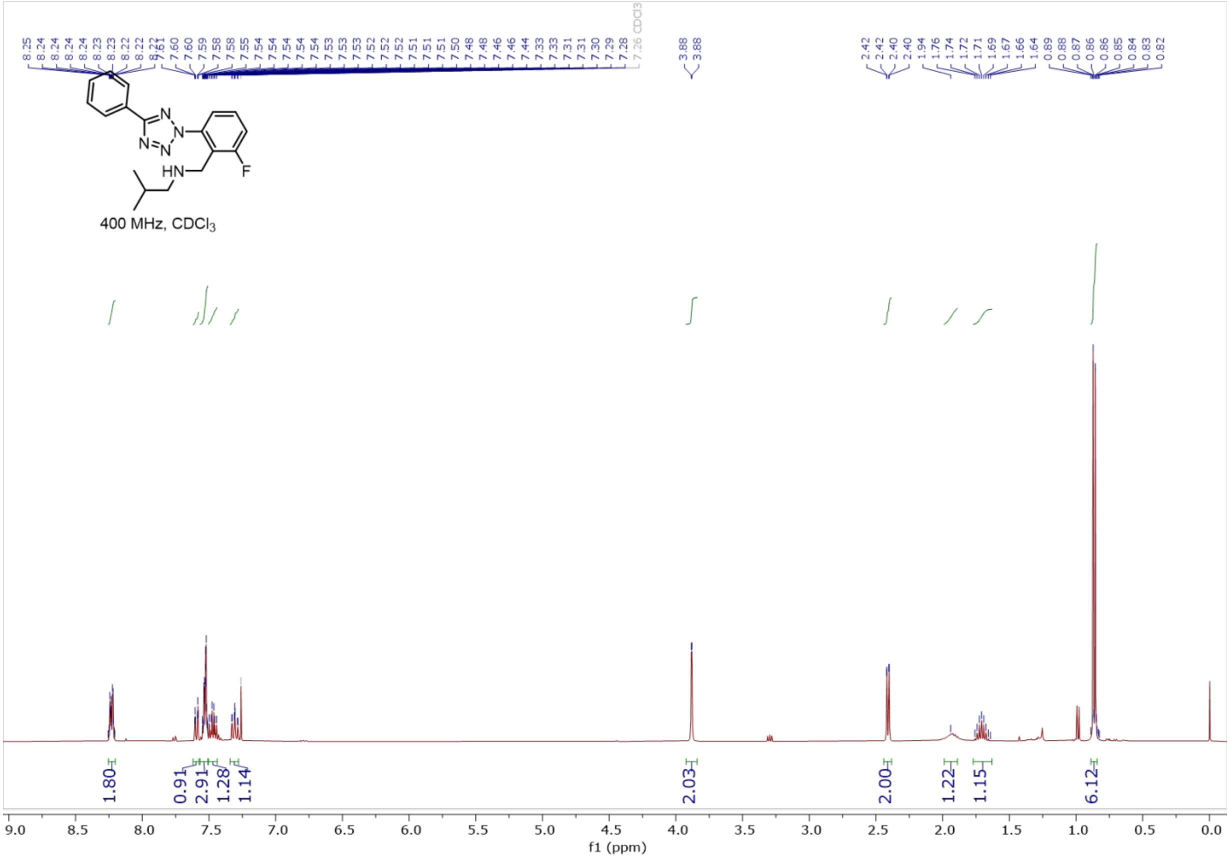

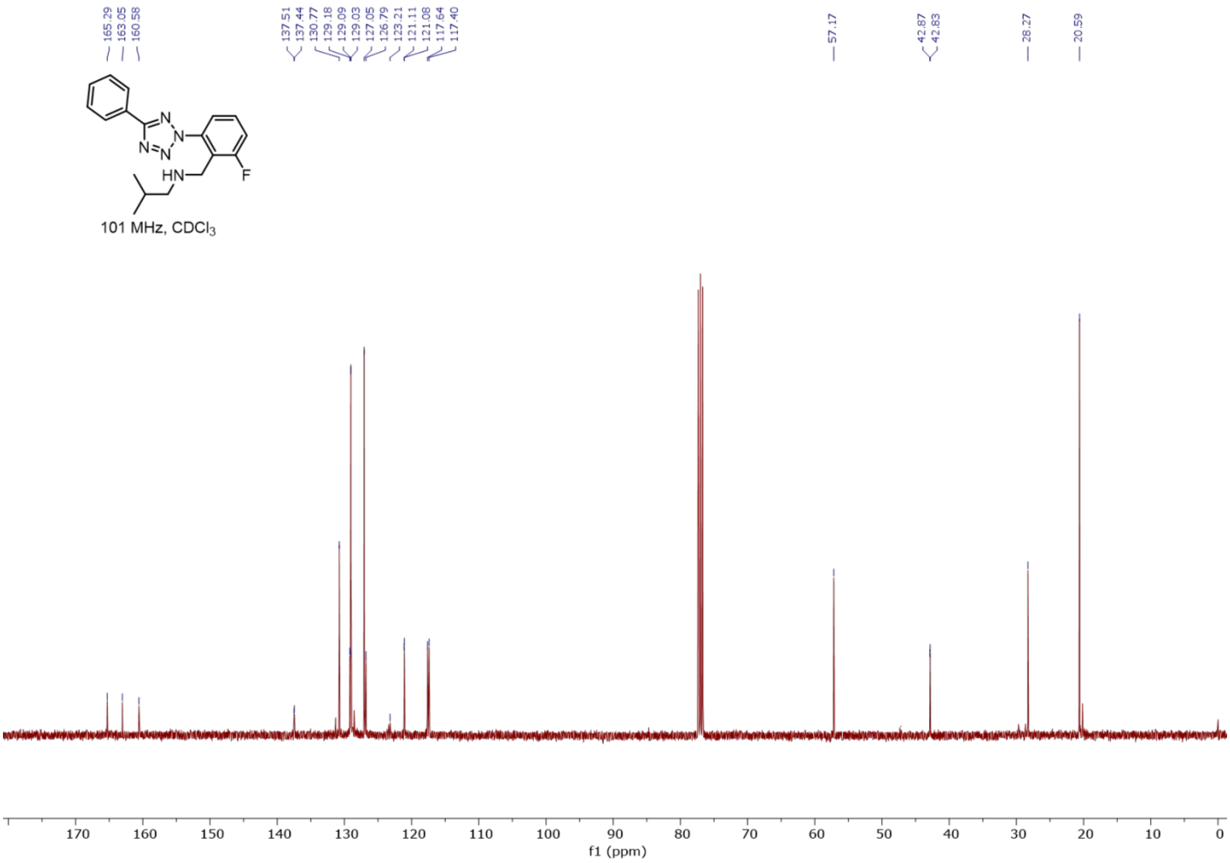

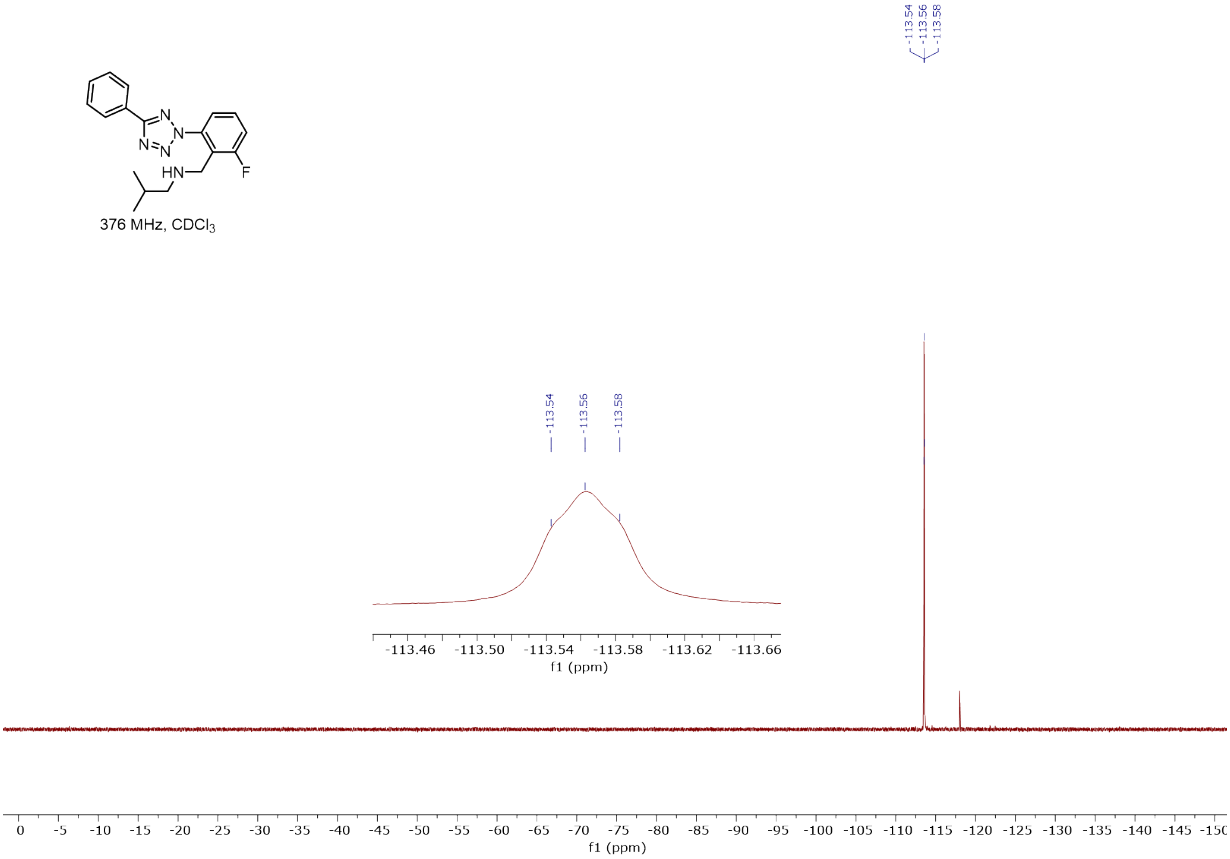

Methyl (2-fluoro-6-(5-phenyl-2*H*-tetrazol-2-yl)benzyl)glycinate (**3j**)

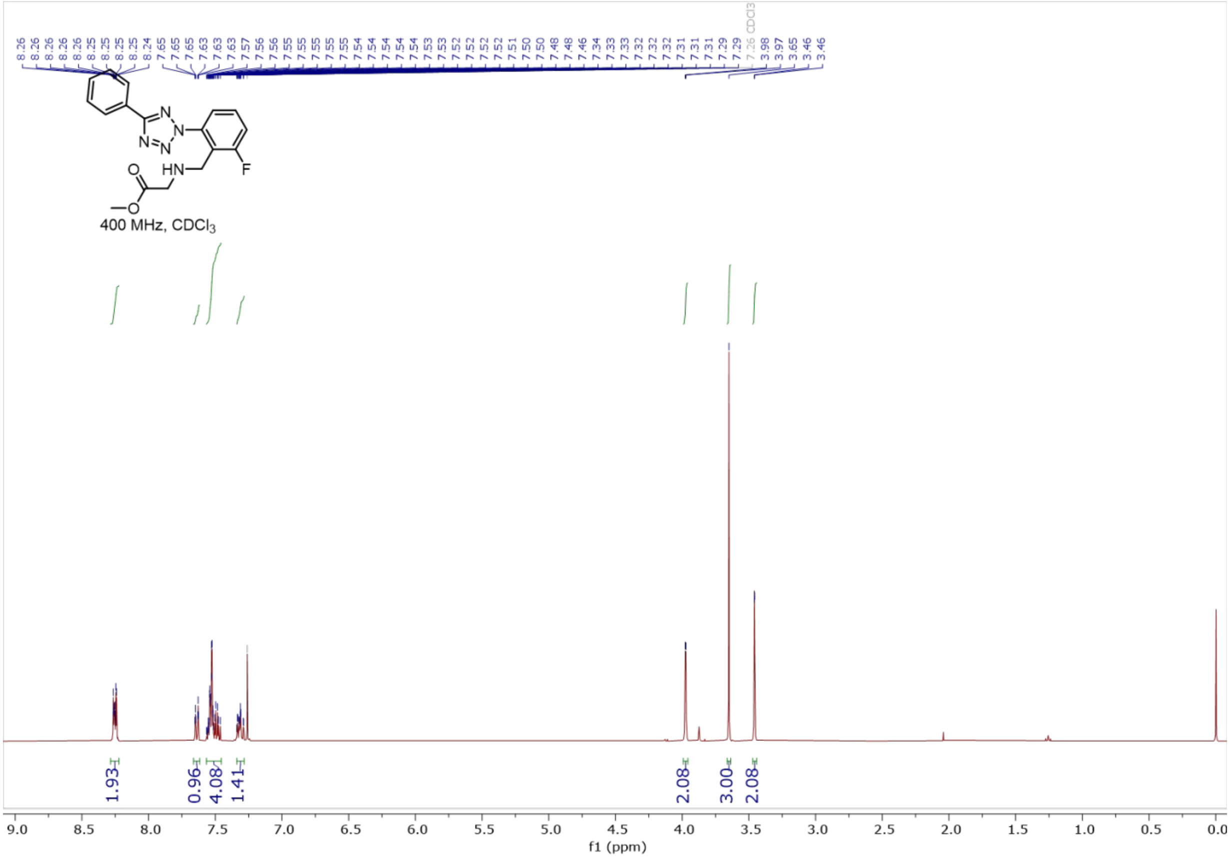

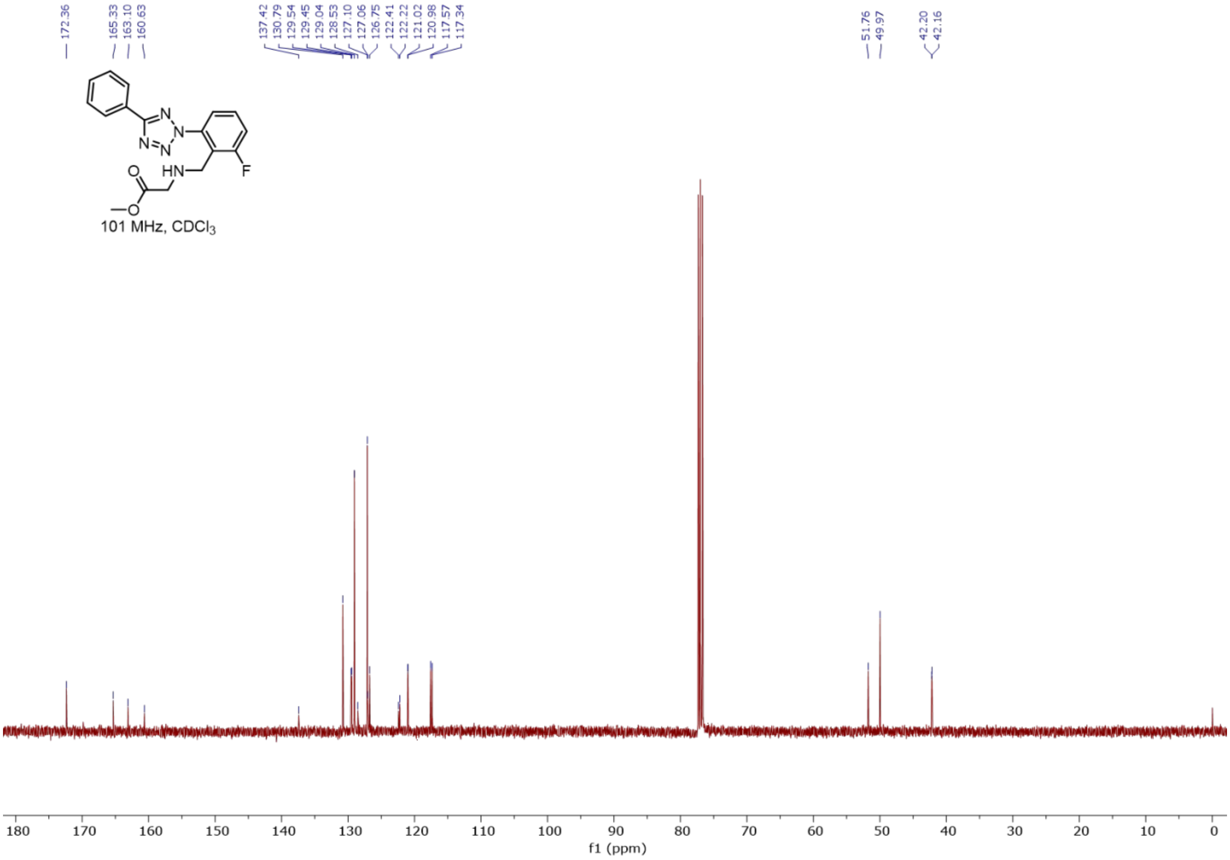
172.36
165.33
163.10
160.63
137.42
130.79
129.54
129.45
129.04
128.53
127.10
127.06
126.75
122.41
122.22
121.02
120.98
117.57
117.34
51.76
49.97
42.20
42.16
N
N
N
N
HN
F
O
O
101 MHz, CDCl3
180 170 160 150 140 130 120 110 100 90 80 70 60 50 40 30 20 10 0
f1 (ppm)

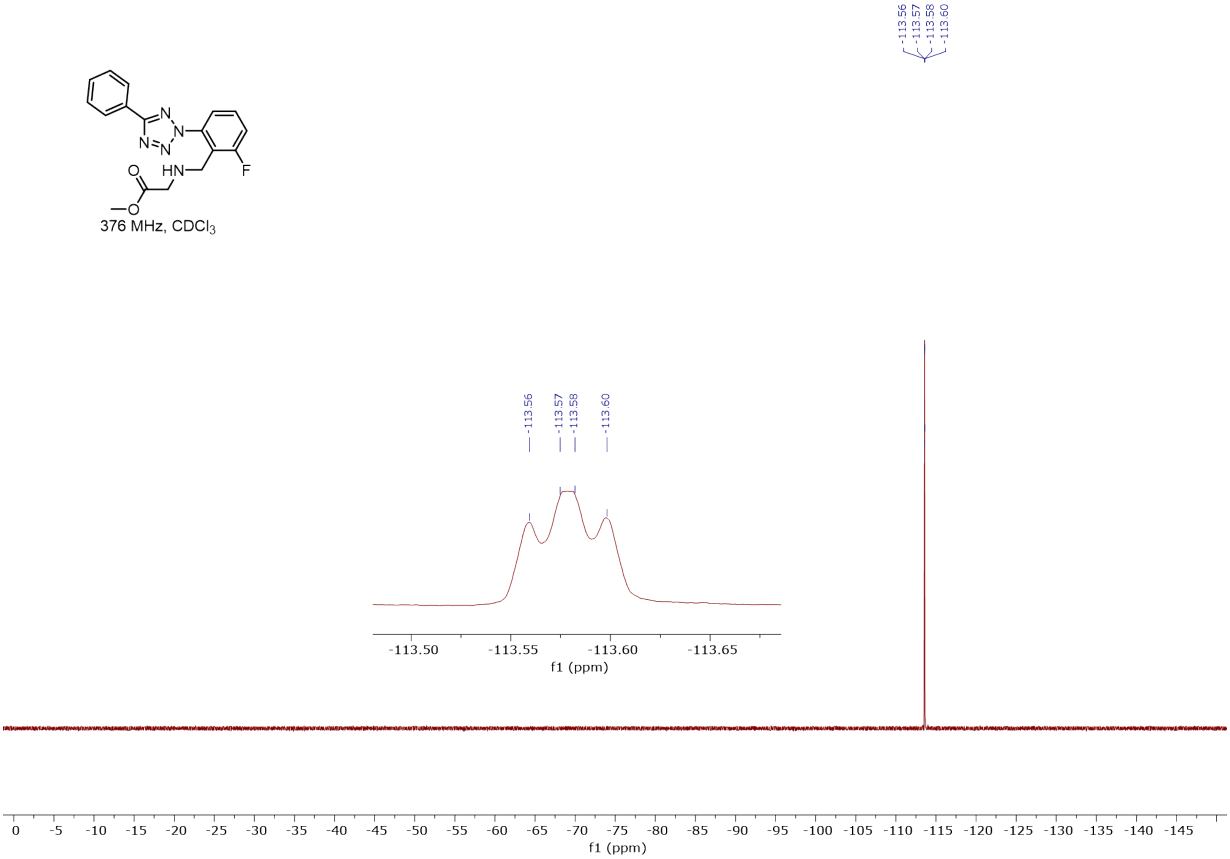
-113.56
-113.57
-113.58
-113.60
N
N
N
N
HN
F
O
O
376 MHz, CDCl3
-113.56
-113.57
-113.58
-113.60
-113.50 -113.55 -113.60 -113.65
f1 (ppm)
0 -5 -10 -15 -20 -25 -30 -35 -40 -45 -50 -55 -60 -65 -70 -75 -80 -85 -90 -95 -100 -105 -110 -115 -120 -125 -130 -135 -140 -145
f1 (ppm)

N-(2-Fluoro-6-(5-phenyl-2*H*-tetrazol-2-yl)benzyl)cyclopentanamine (**3k**)

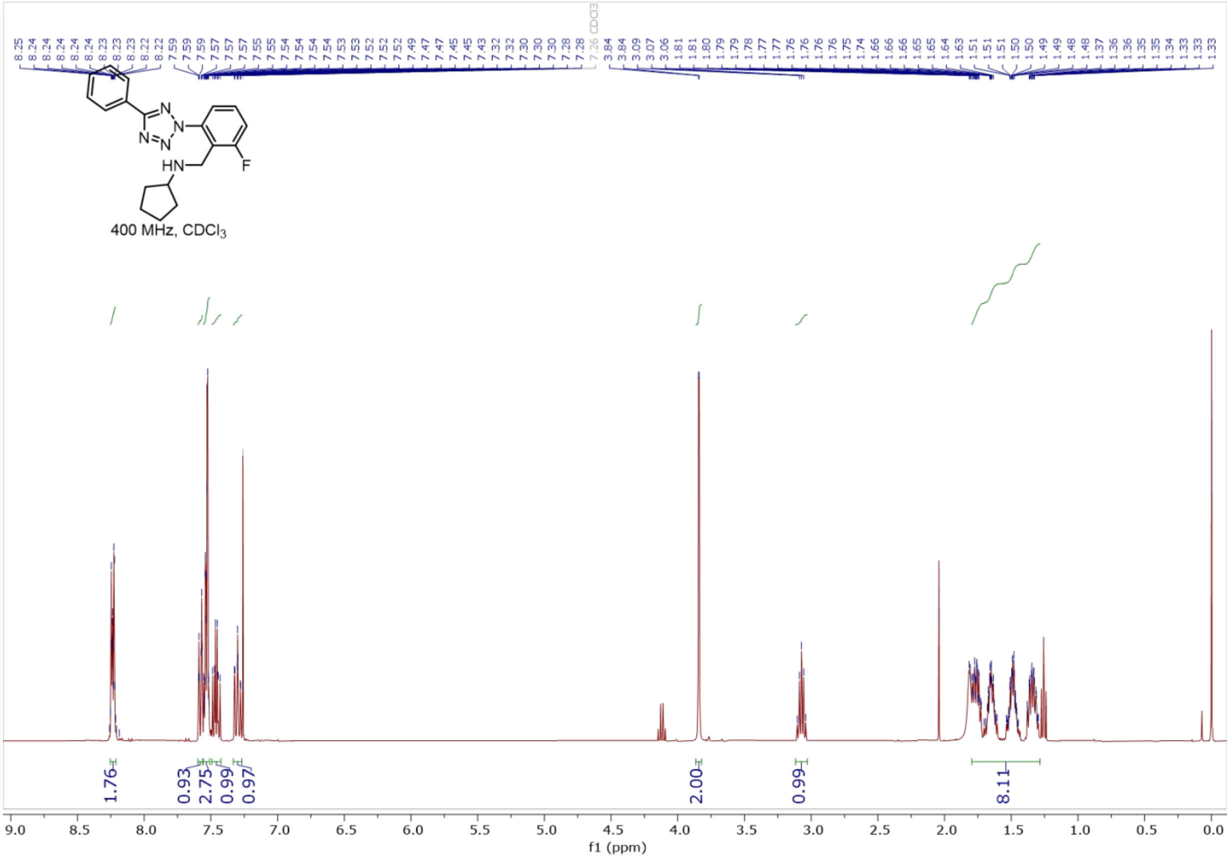

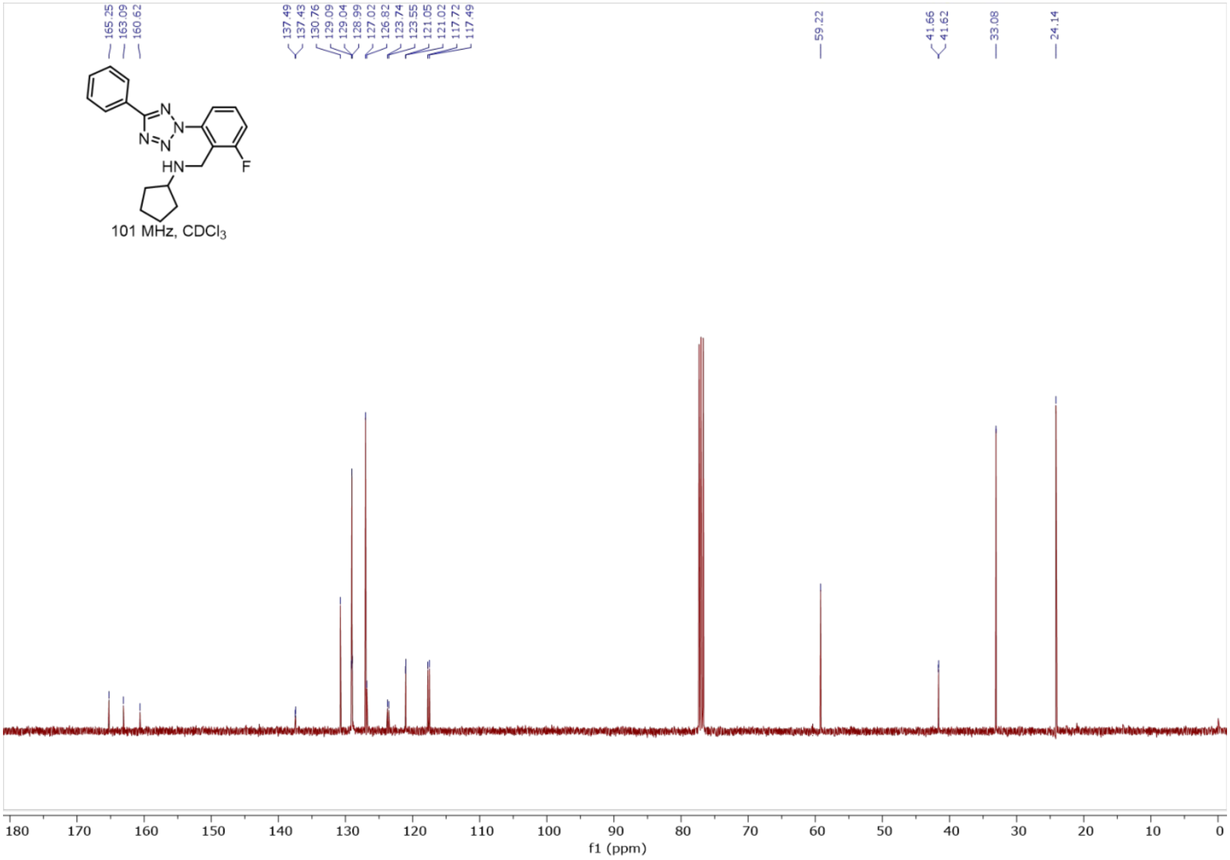

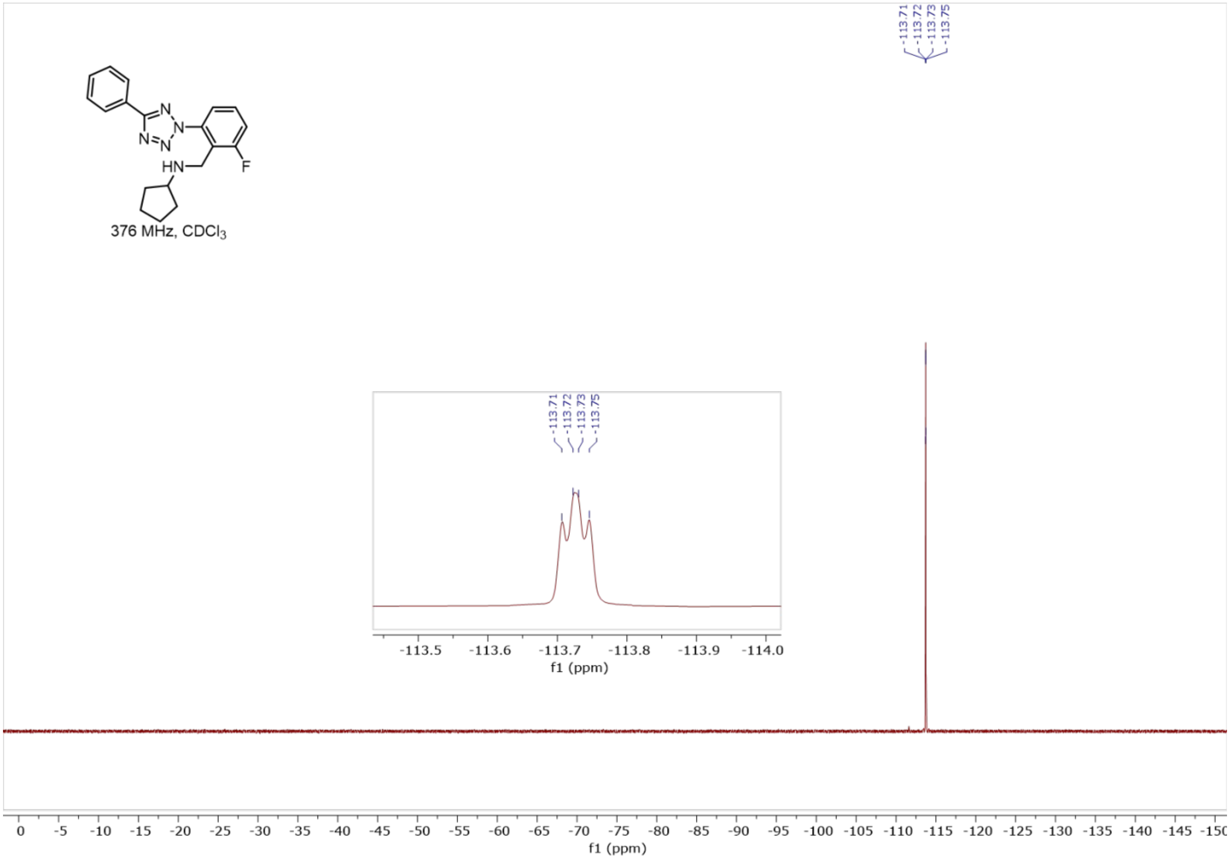

N-(2-Fluoro-6-(5-phenyl-2*H*-tetrazol-2-yl)benzyl)-4-(trifluoromethoxy)aniline (**3l**)

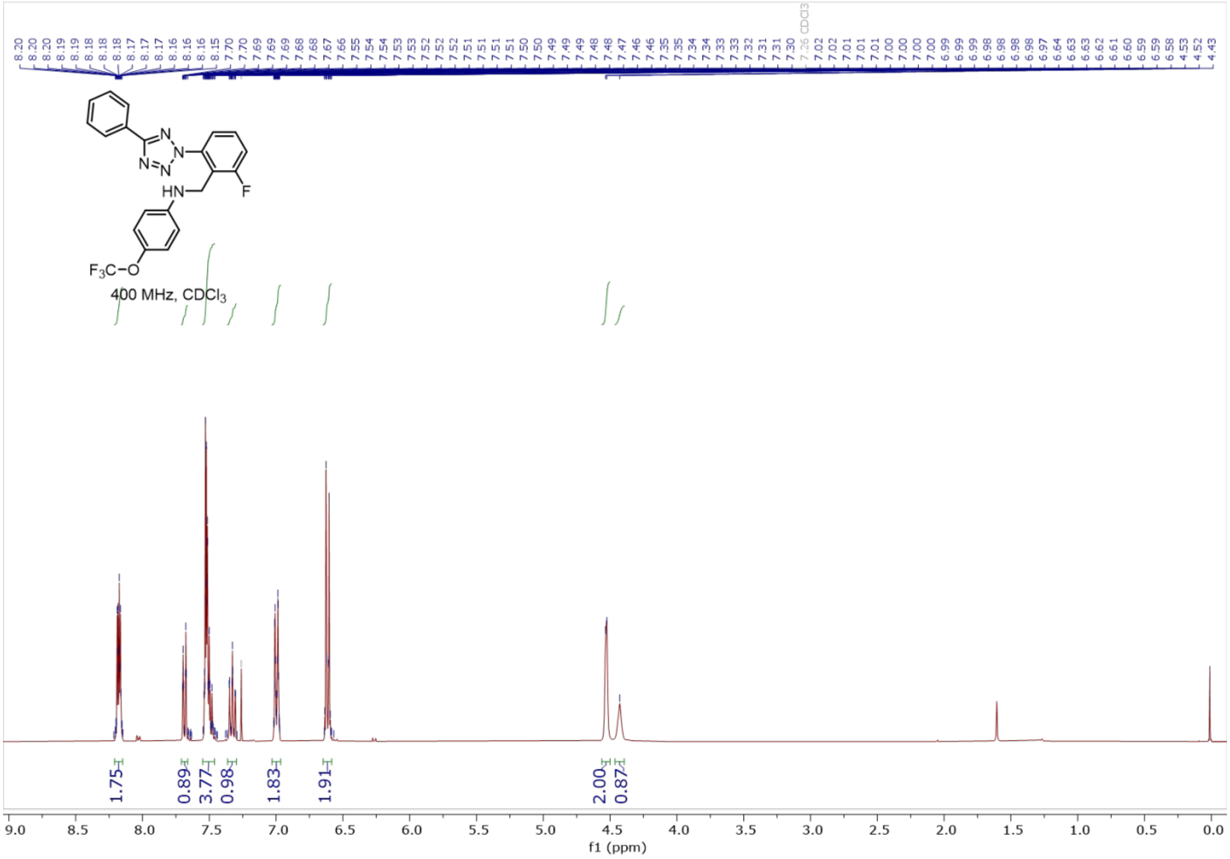

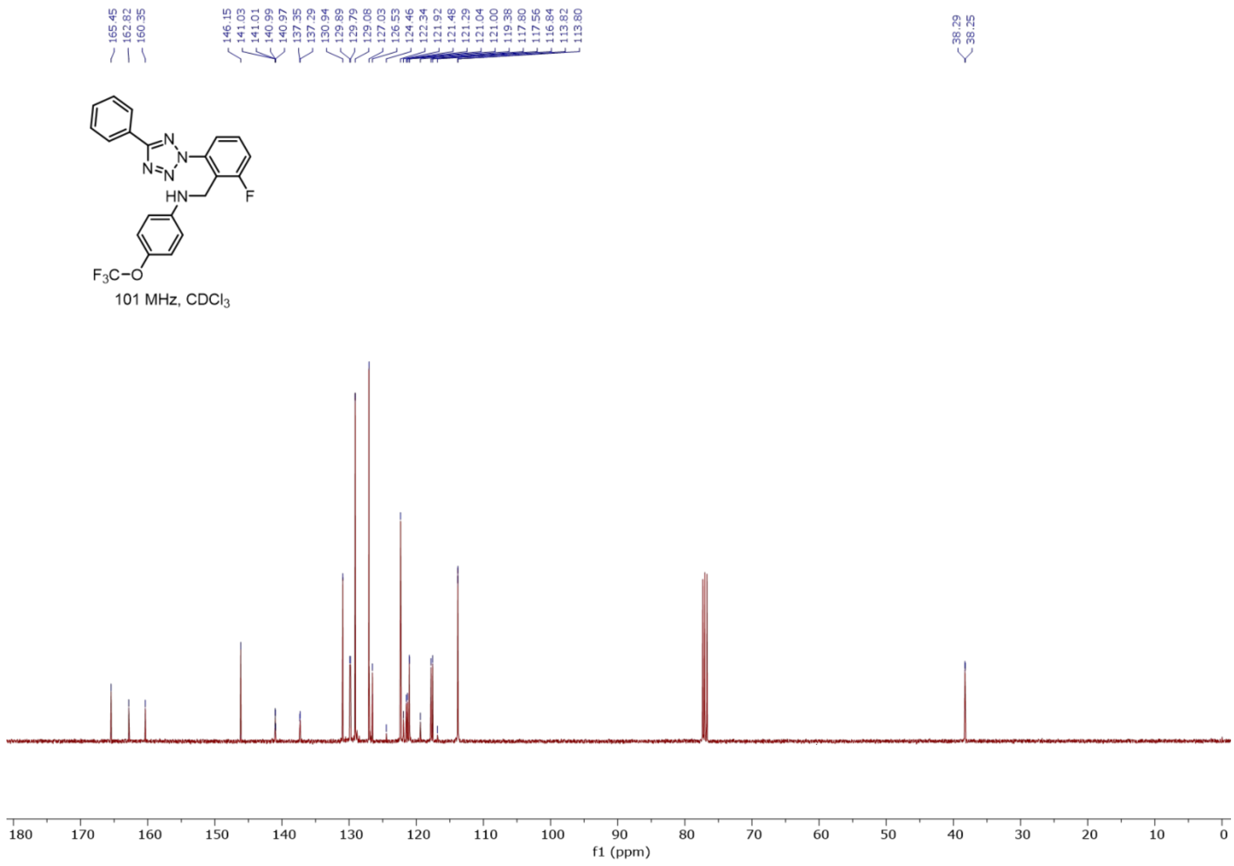
101 MHz, CDCl3
f1 (ppm)

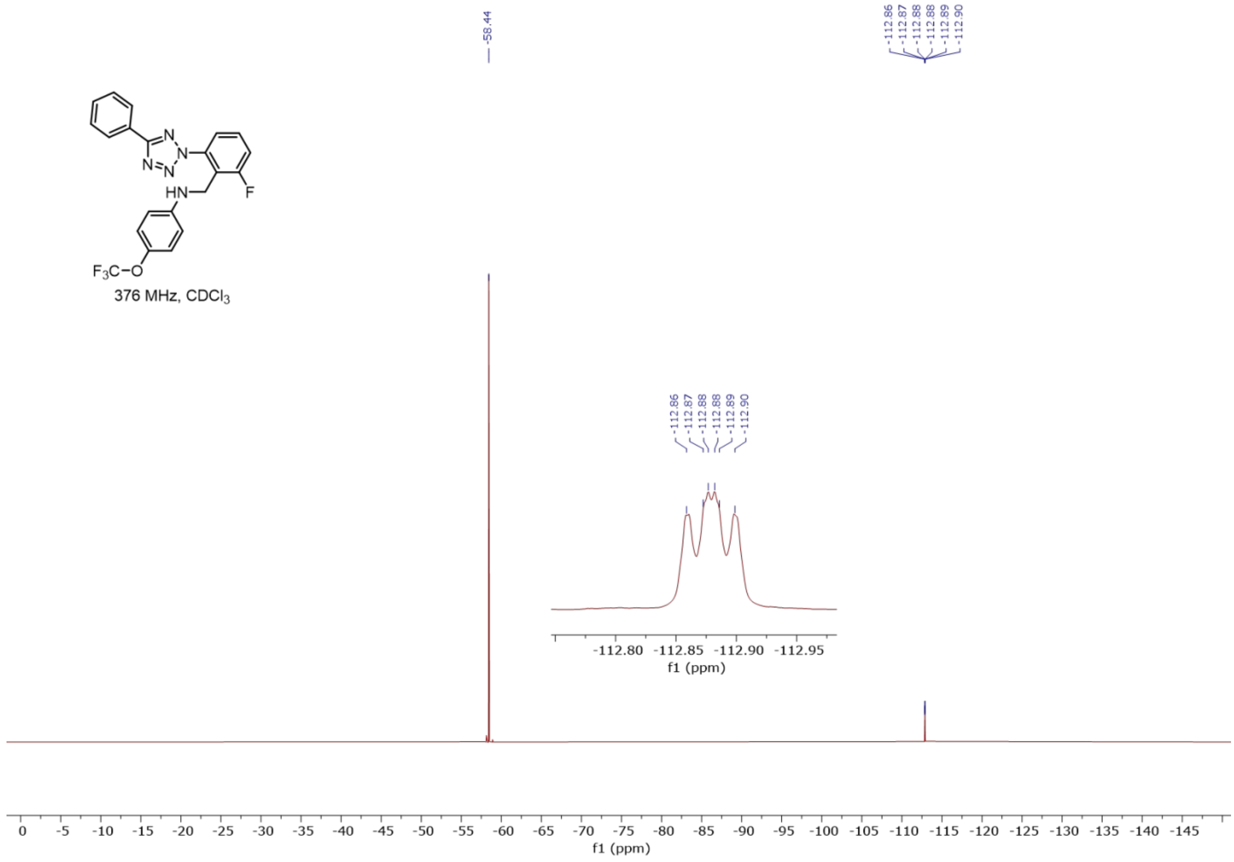
376 MHz, CDCl3
f1 (ppm)

N-(2-Fluoro-6-(5-phenyl-2H-tetrazol-2-yl)benzyl)aniline (**3m**)

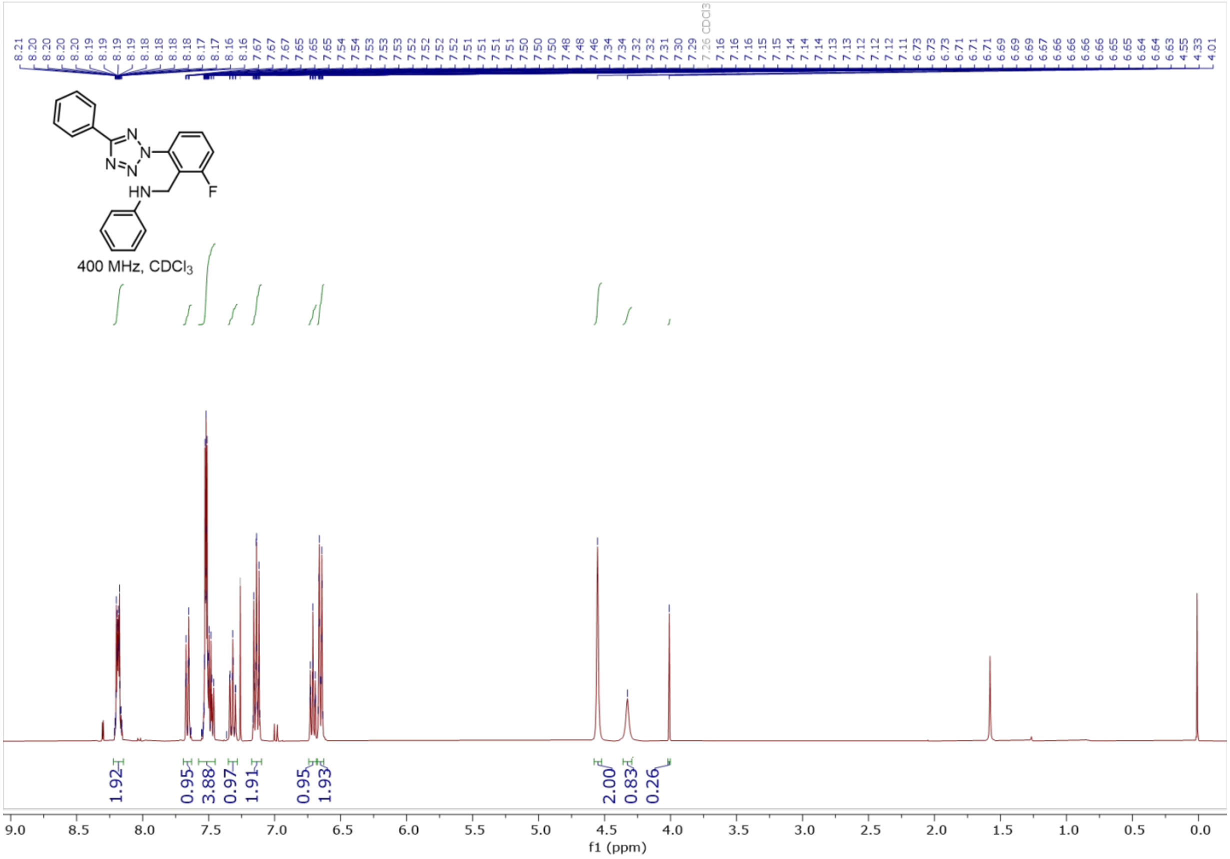

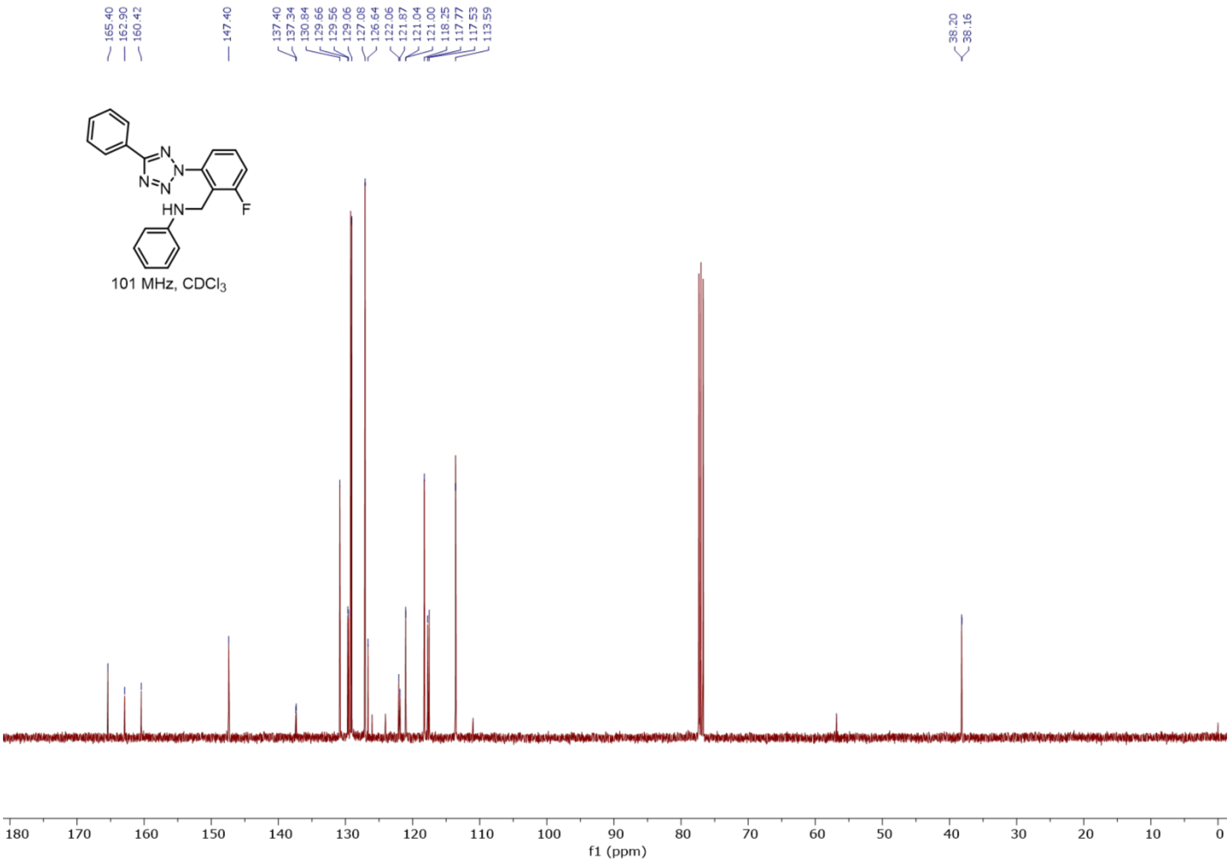

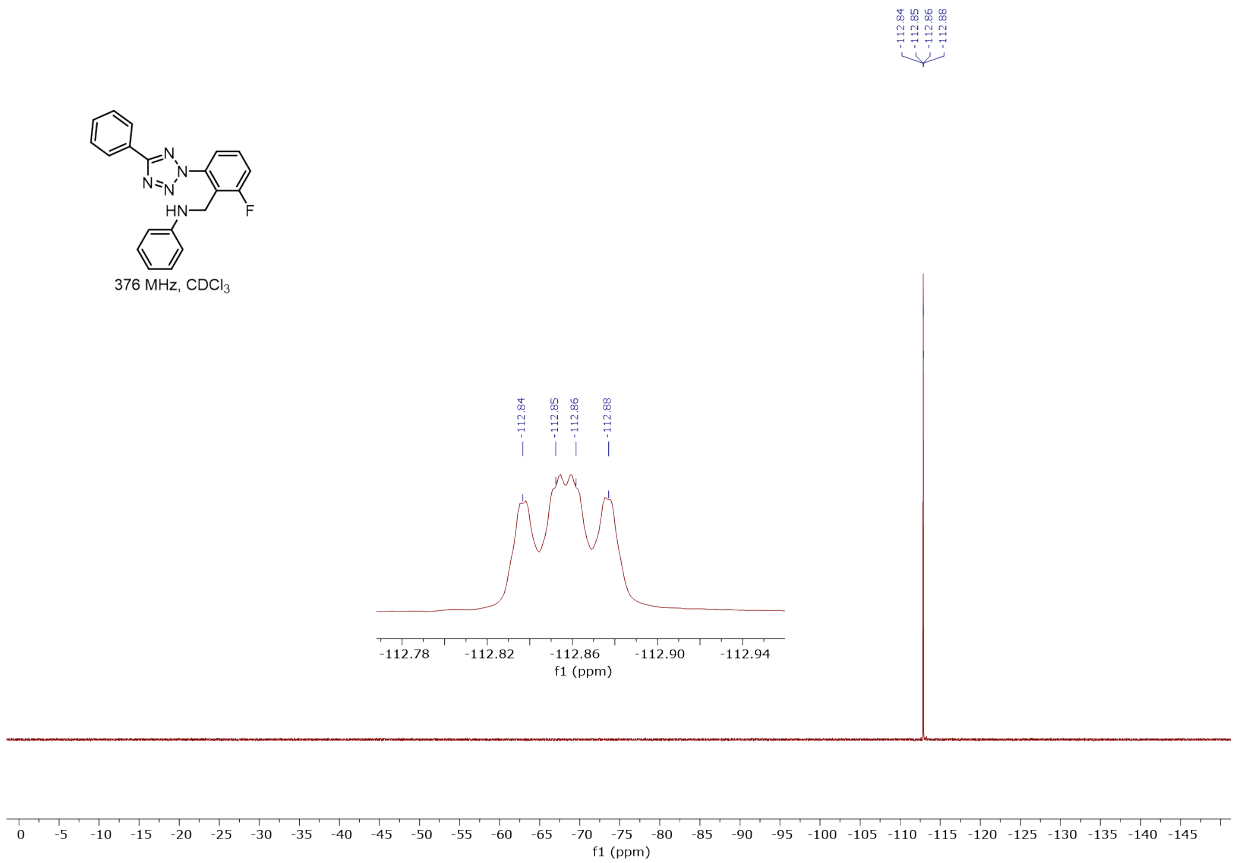

N-(2-Fluoro-6-(5-phenyl-2*H*-tetrazol-2-yl)benzyl)-1-(4-methoxyphenyl)methanamine (**3n**)

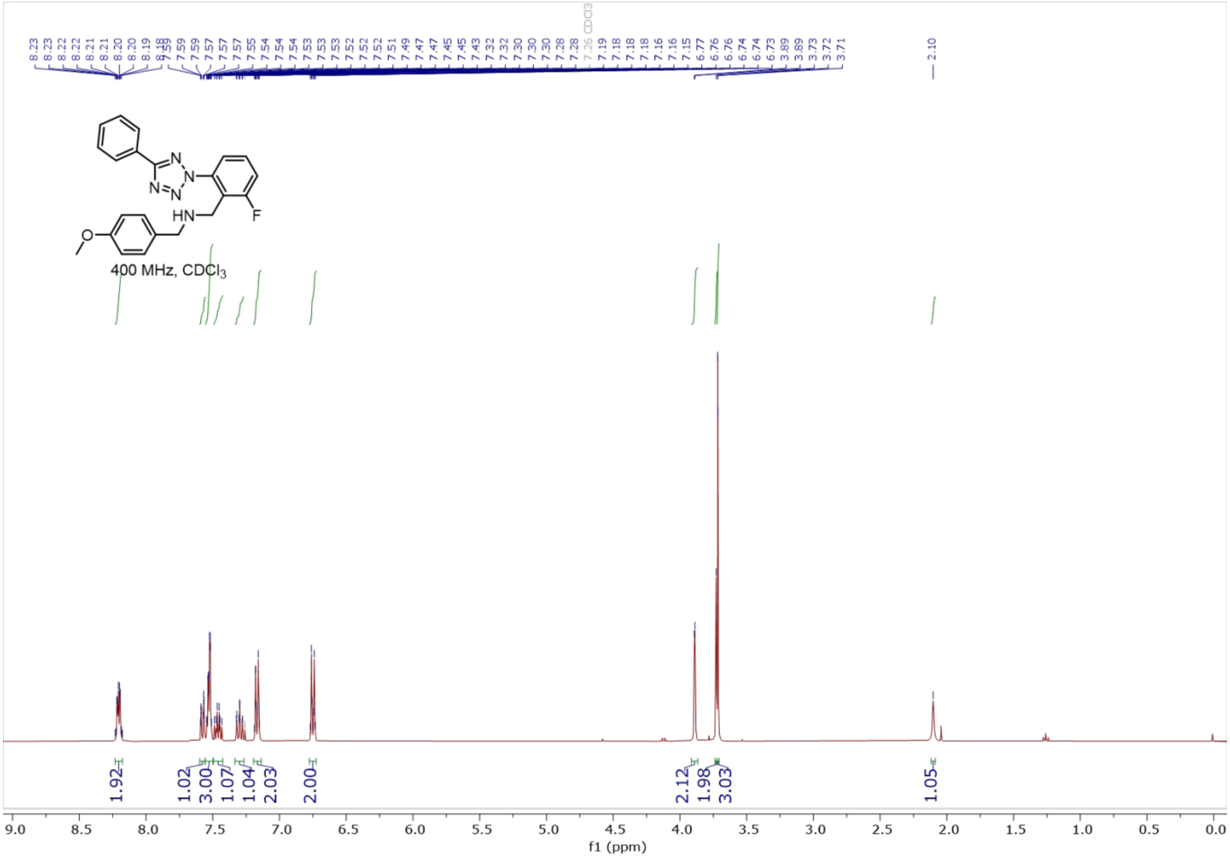

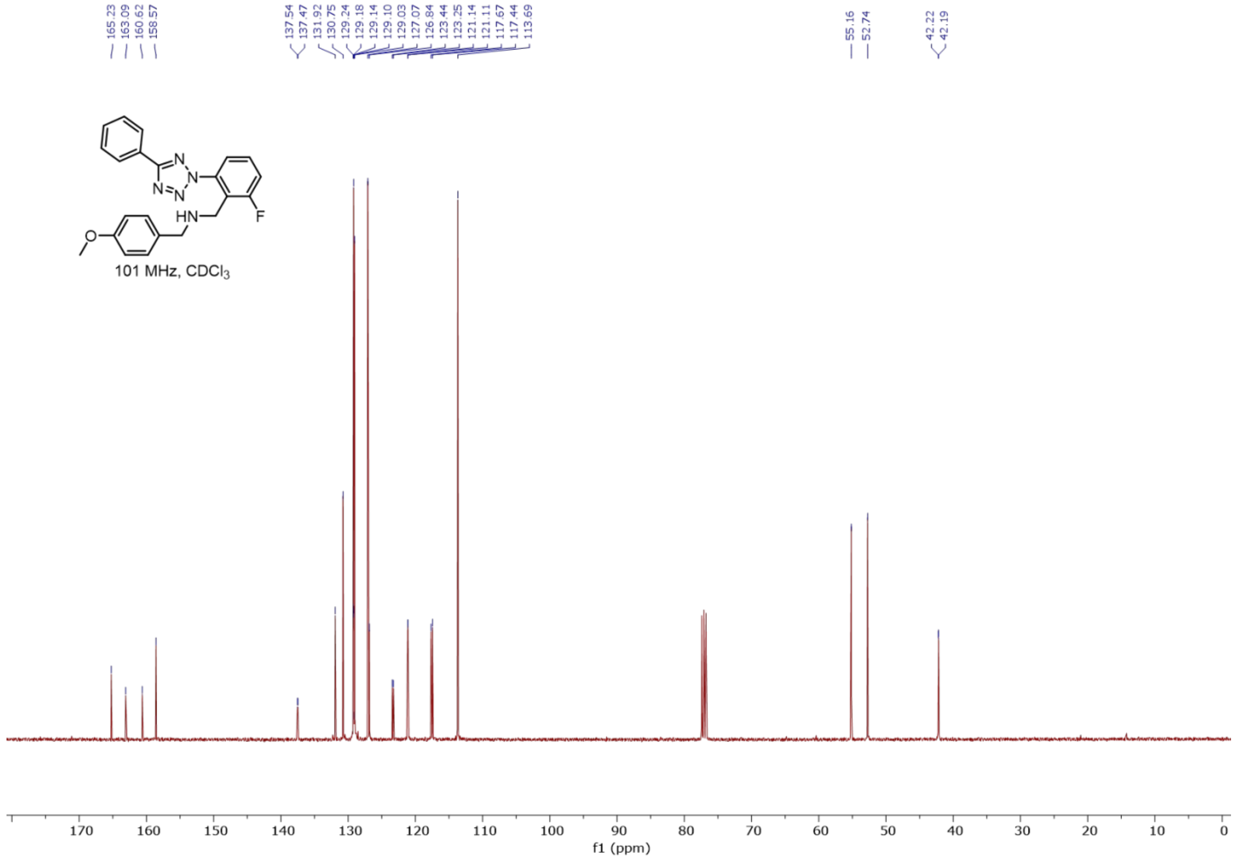
165.23
163.09
160.62
158.57
137.54
137.47
131.92
130.75
129.24
129.18
129.14
129.10
129.03
127.07
126.84
123.44
123.25
121.14
121.11
117.67
117.44
113.69
55.16
52.74
42.22
42.19
101 MHz, CDCl3
f1 (ppm)

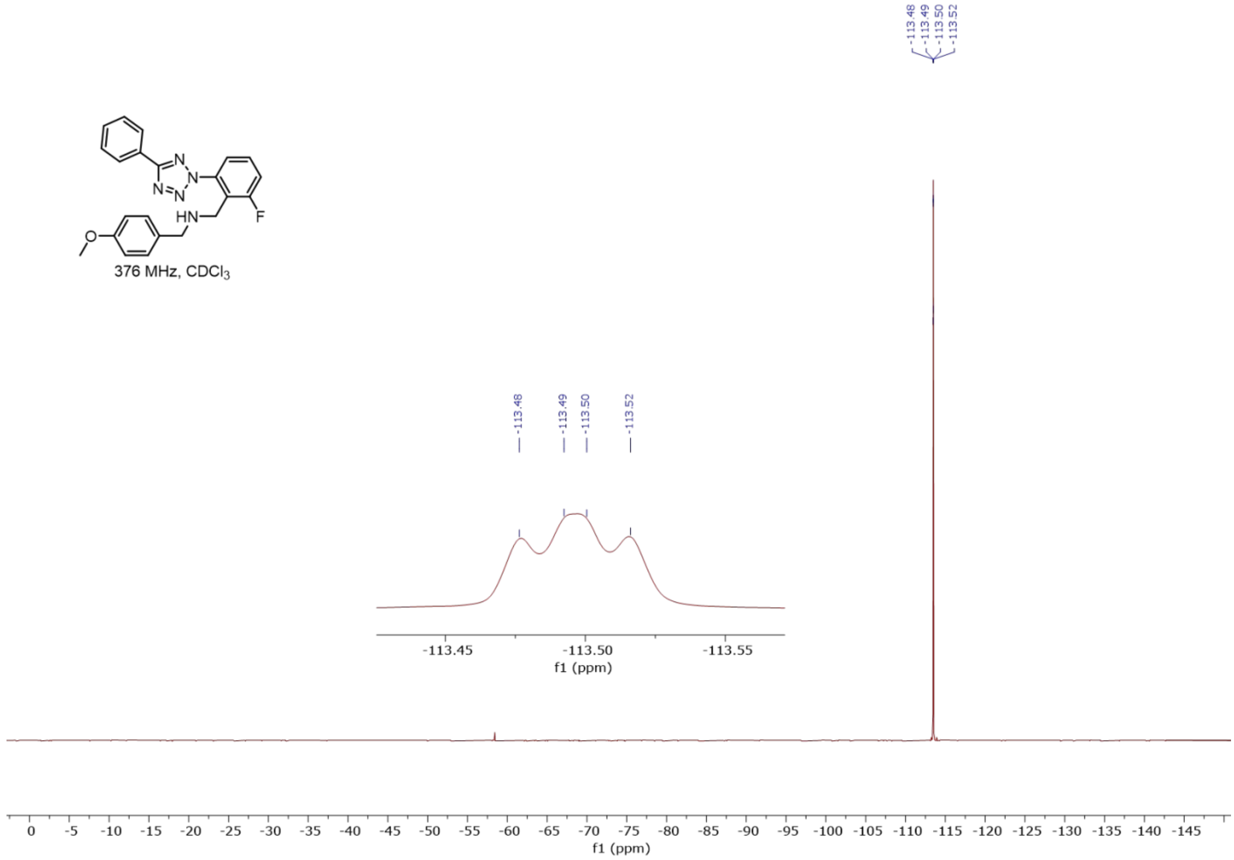
-113.48
-113.49
-113.50
-113.52
376 MHz, CDCl3
f1 (ppm)

N-ethyl-*N*-(2-fluoro-6-(5-phenyl-2*H*-tetrazol-2-yl)benzyl)ethanamine (**3o**)

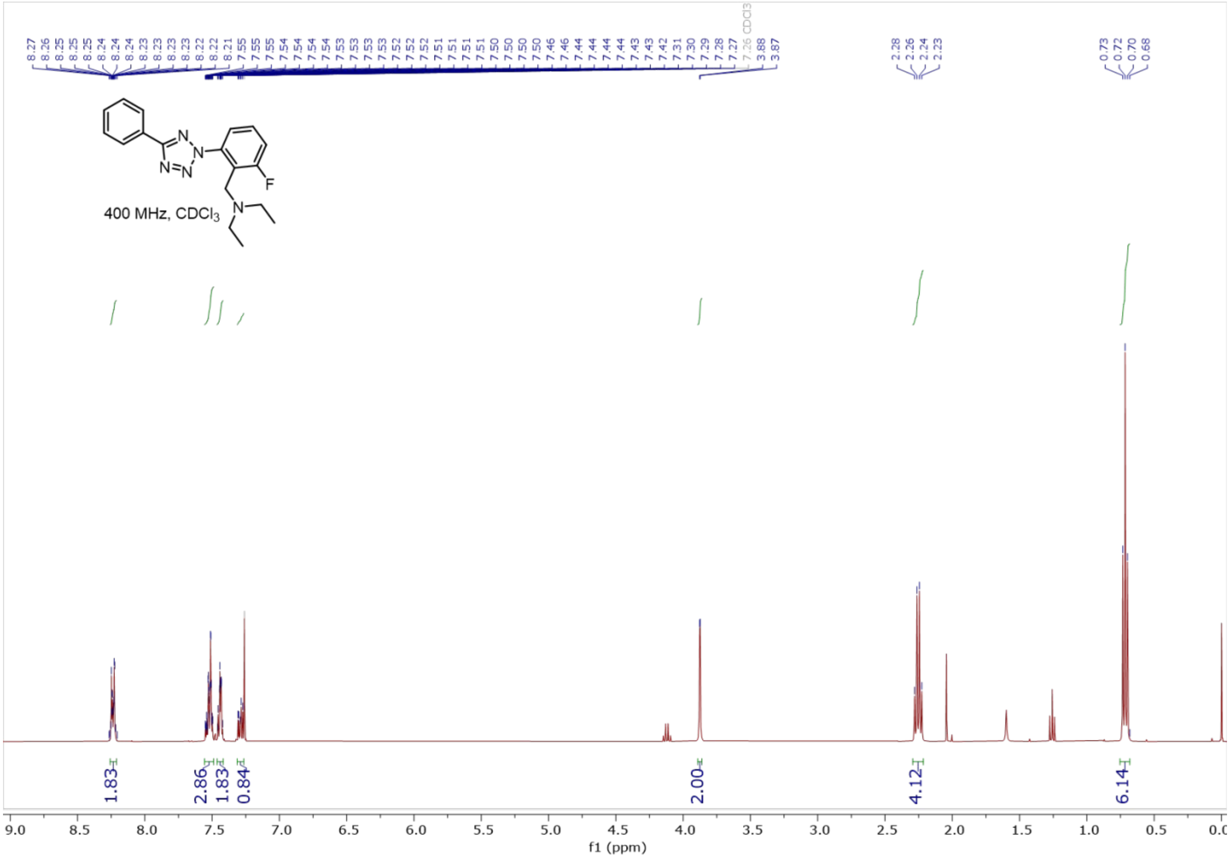

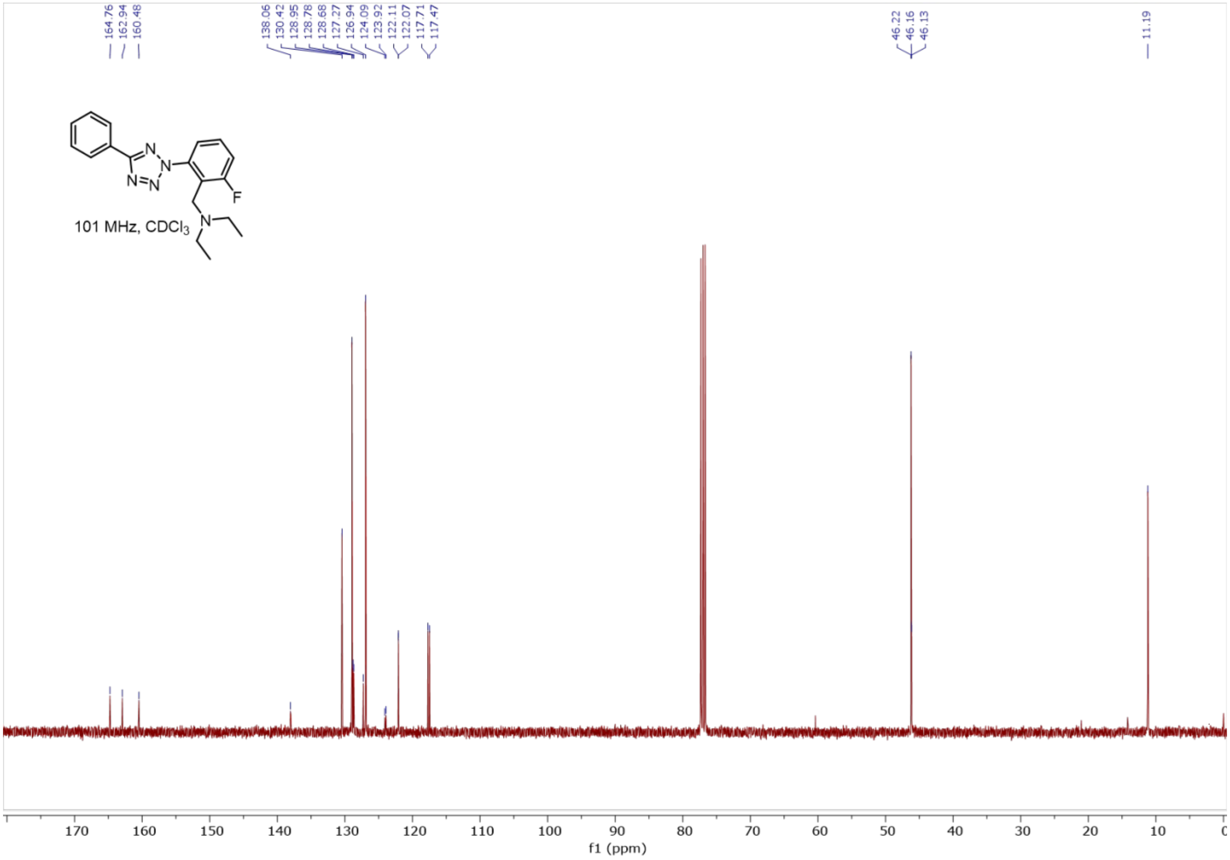

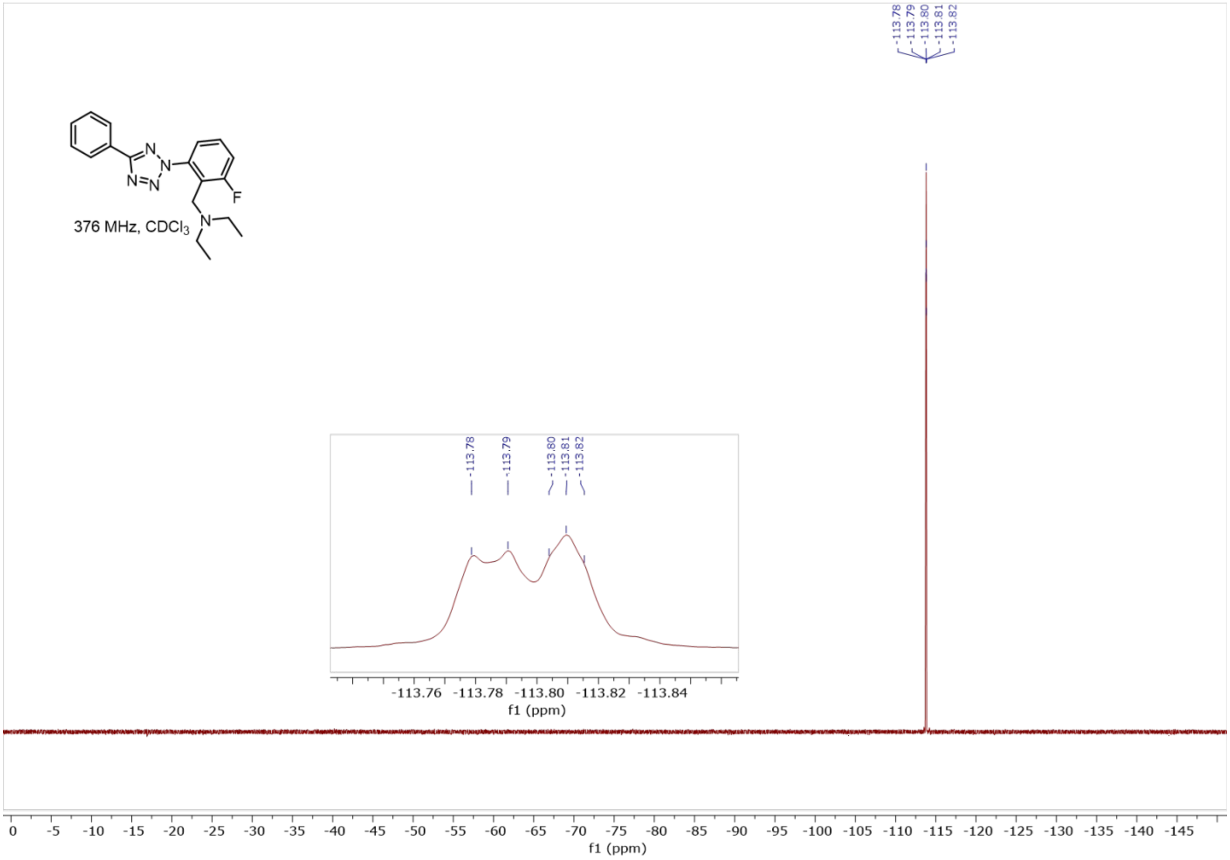

7-Fluoro-2-phenyl-4*H*,6*H*-benzo[e]pyrazolo[5,1-c][1,4]oxazepine (**4a**)

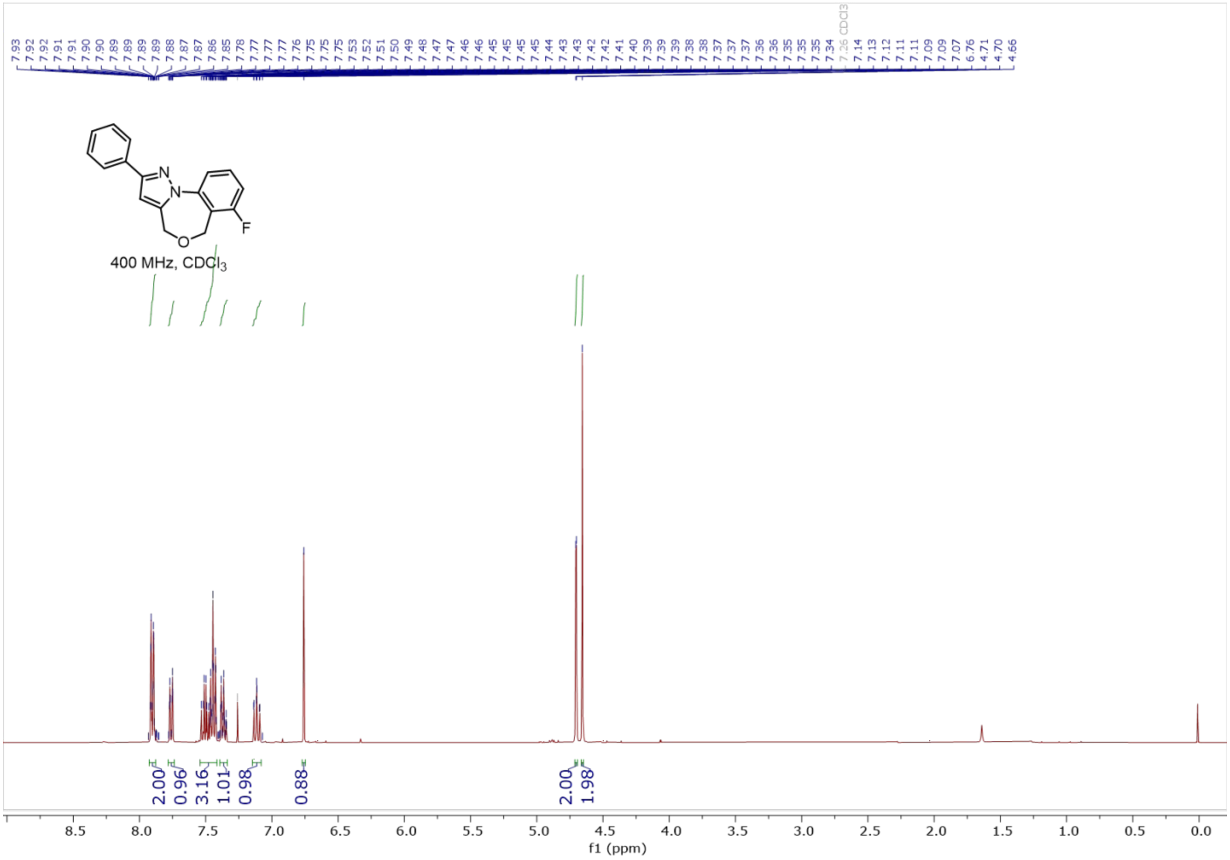

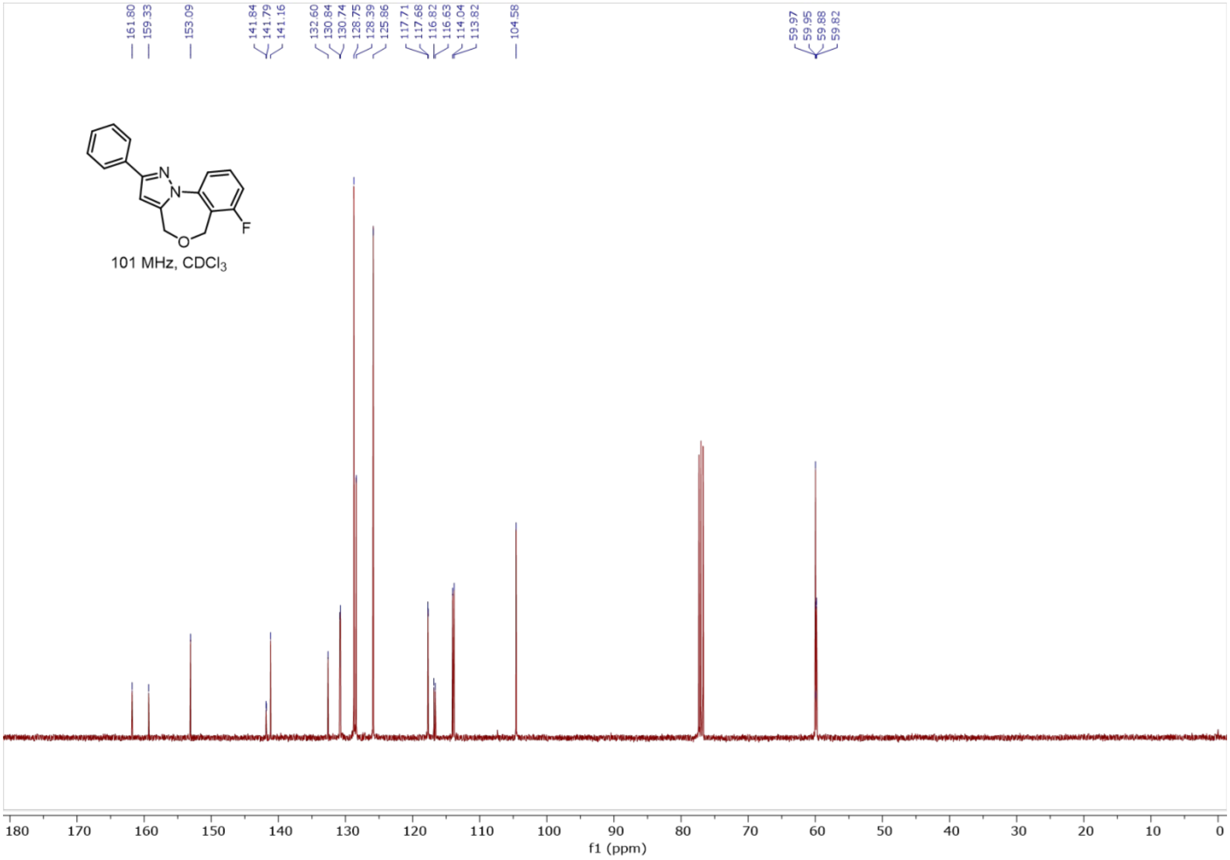
161.80
159.33
153.09
141.84
141.79
141.16
132.60
130.84
130.74
128.75
128.39
125.86
117.71
117.68
116.82
116.63
114.04
113.82
104.58
59.97
59.95
59.88
59.82
N
N
O
F
101 MHz, CDCl3
f1 (ppm)

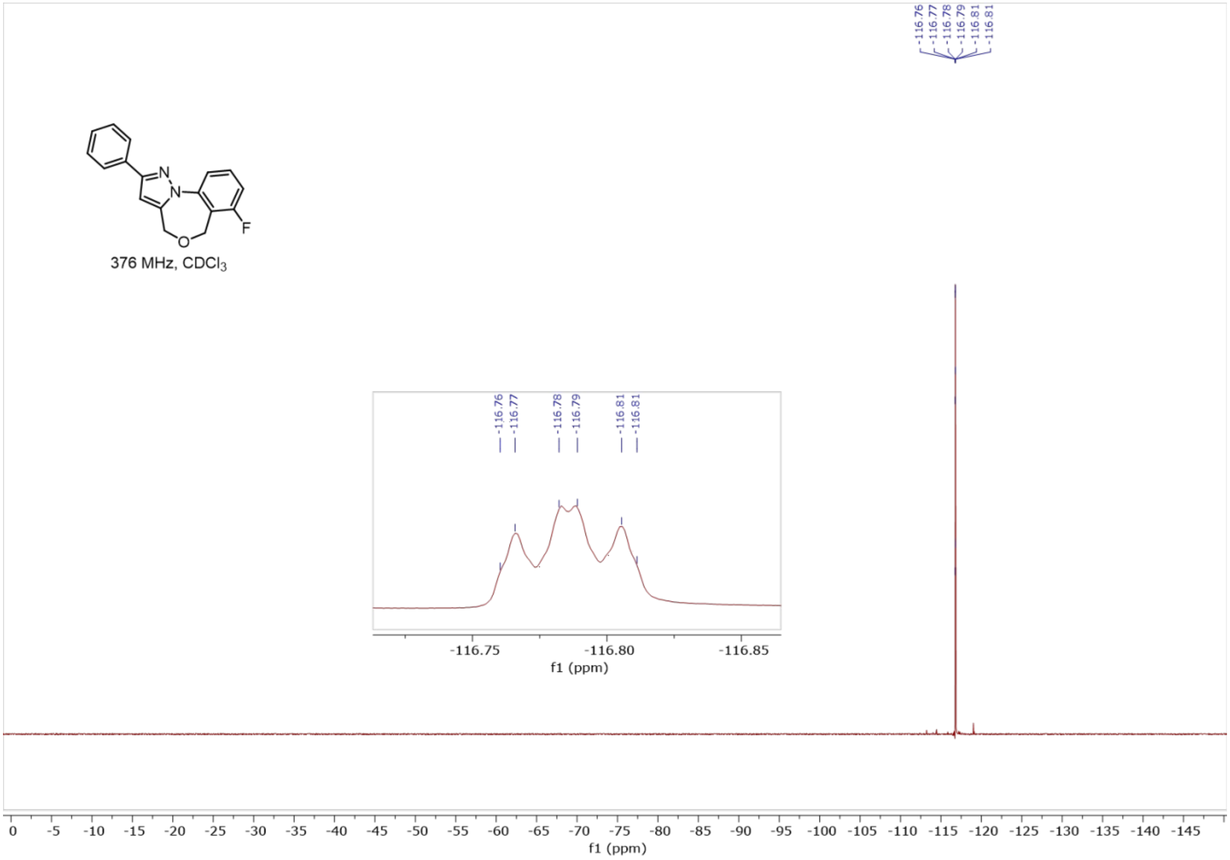
-116.76
-116.77
-116.78
-116.79
-116.81
-116.81
N
N
O
F
376 MHz, CDCl3
-116.75
-116.80
-116.85
f1 (ppm)
f1 (ppm)

10-Methyl-2-phenyl-4*H*,6*H*-benzo[e]pyrazolo[5,1-c][1,4]oxazepine (**4b**)

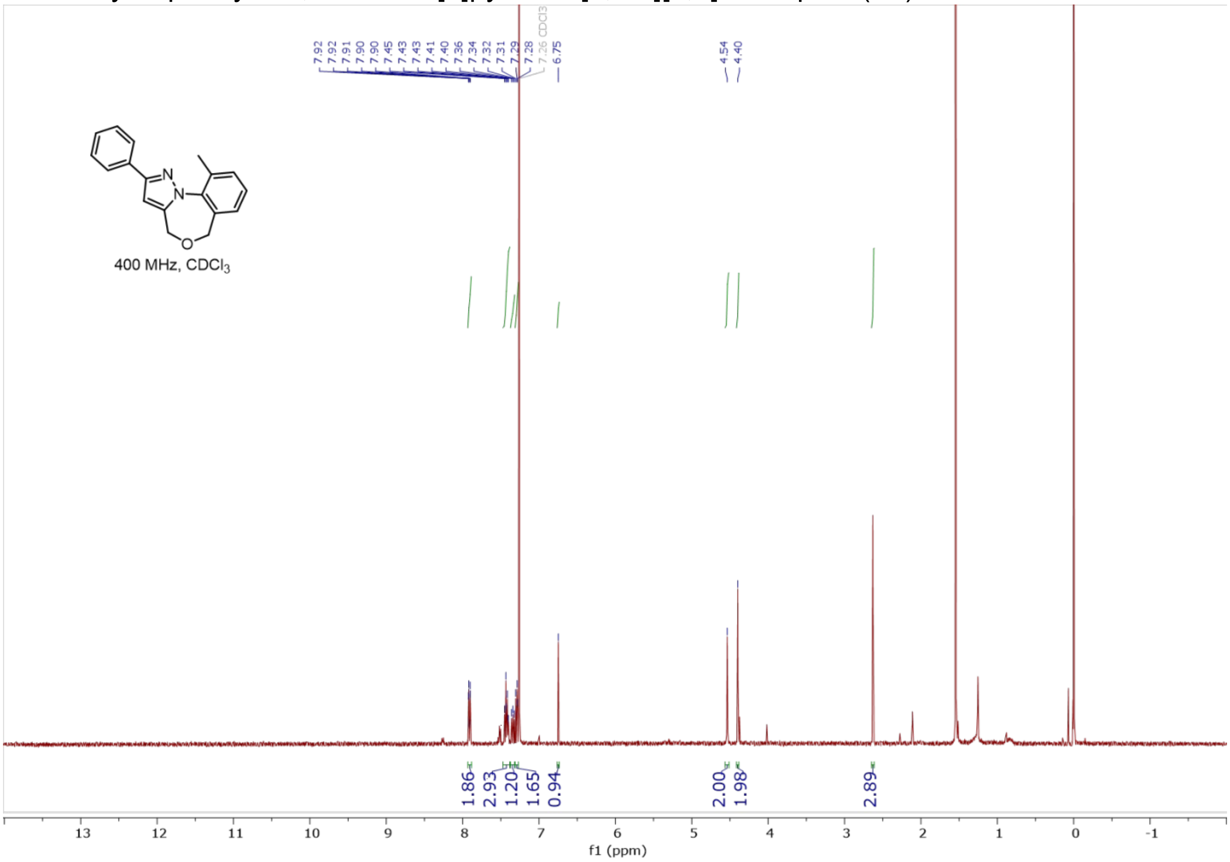

9-Fluoro-2-phenyl-4H,6*H*-benzo[e]pyrazolo[5,1-c][1,4]oxazepine (**4c**)

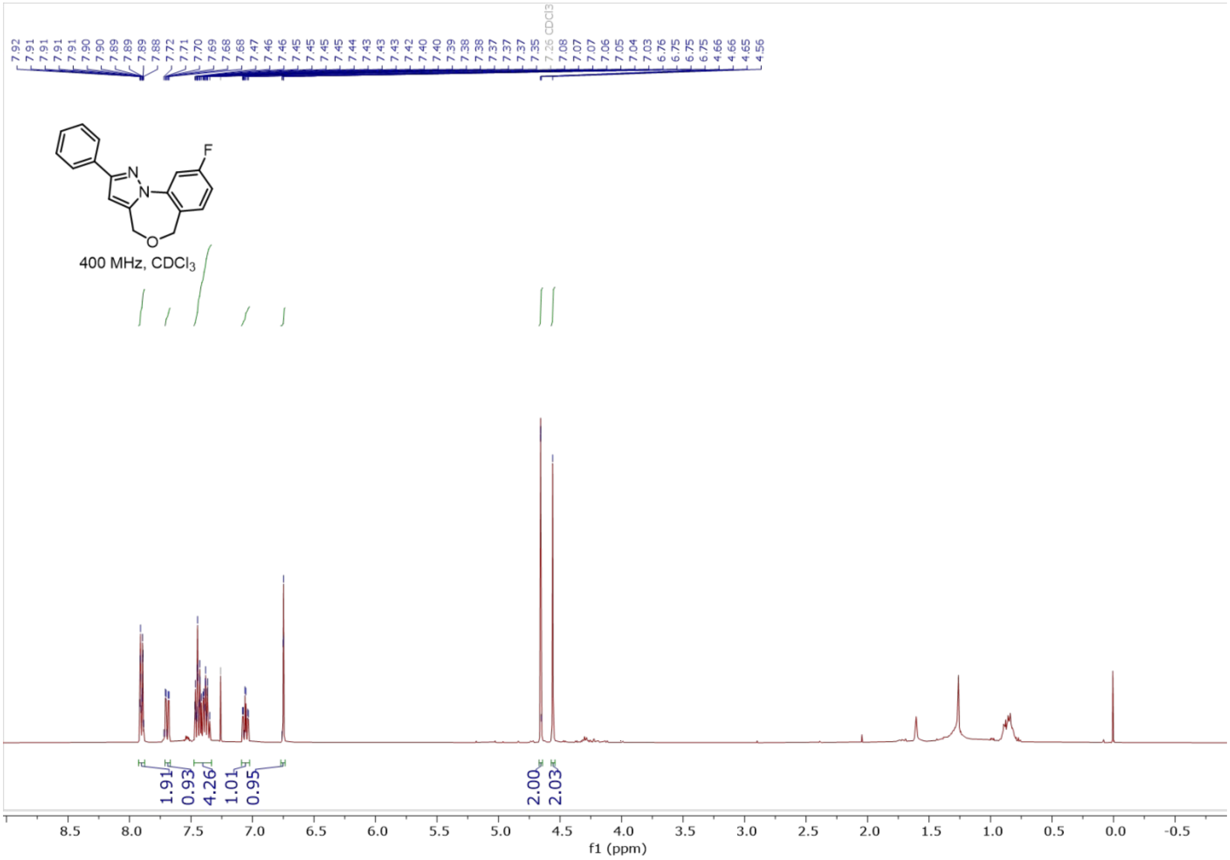

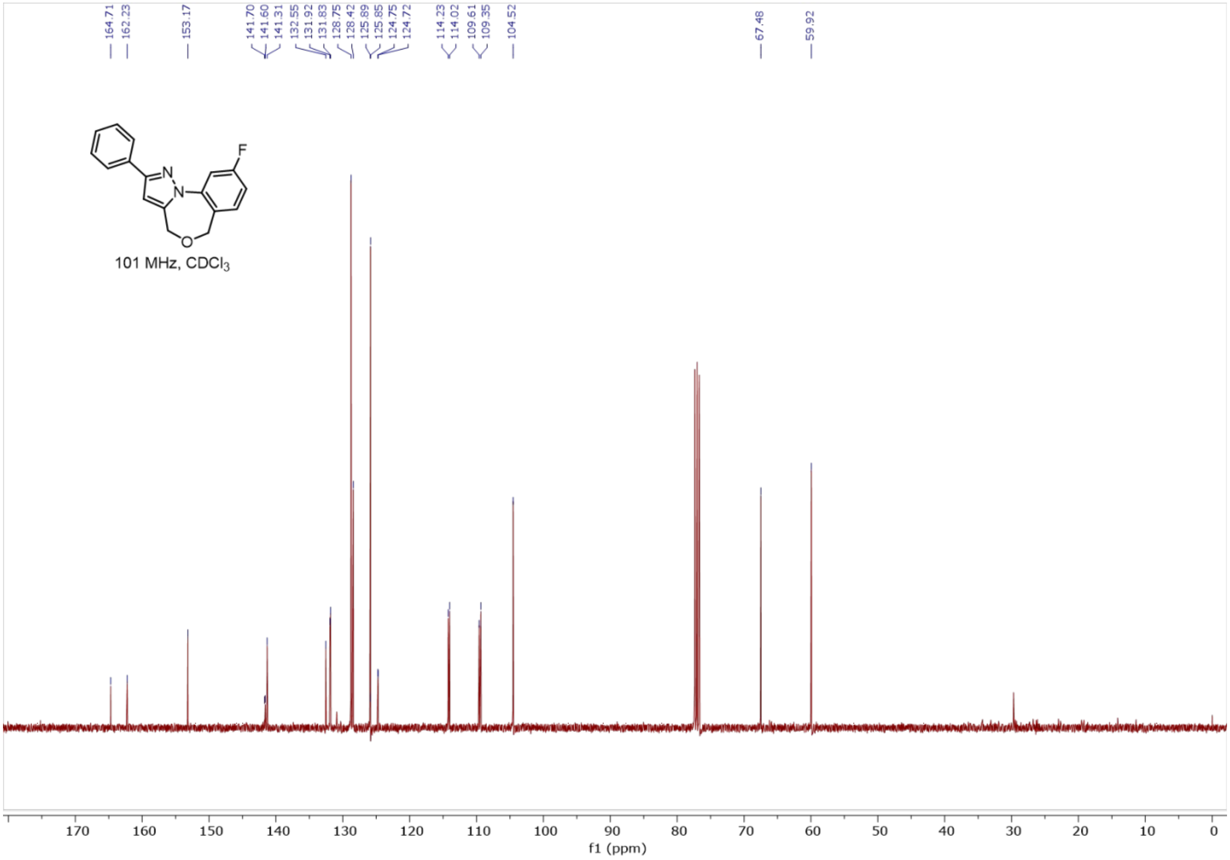
164.71
162.23
153.17
141.70
141.60
141.31
132.55
131.92
131.83
128.75
128.42
125.89
125.85
124.75
124.72
114.23
114.02
109.61
109.35
104.52
67.48
59.92
F
N
N
O
101 MHz, CDCl3
f1 (ppm)

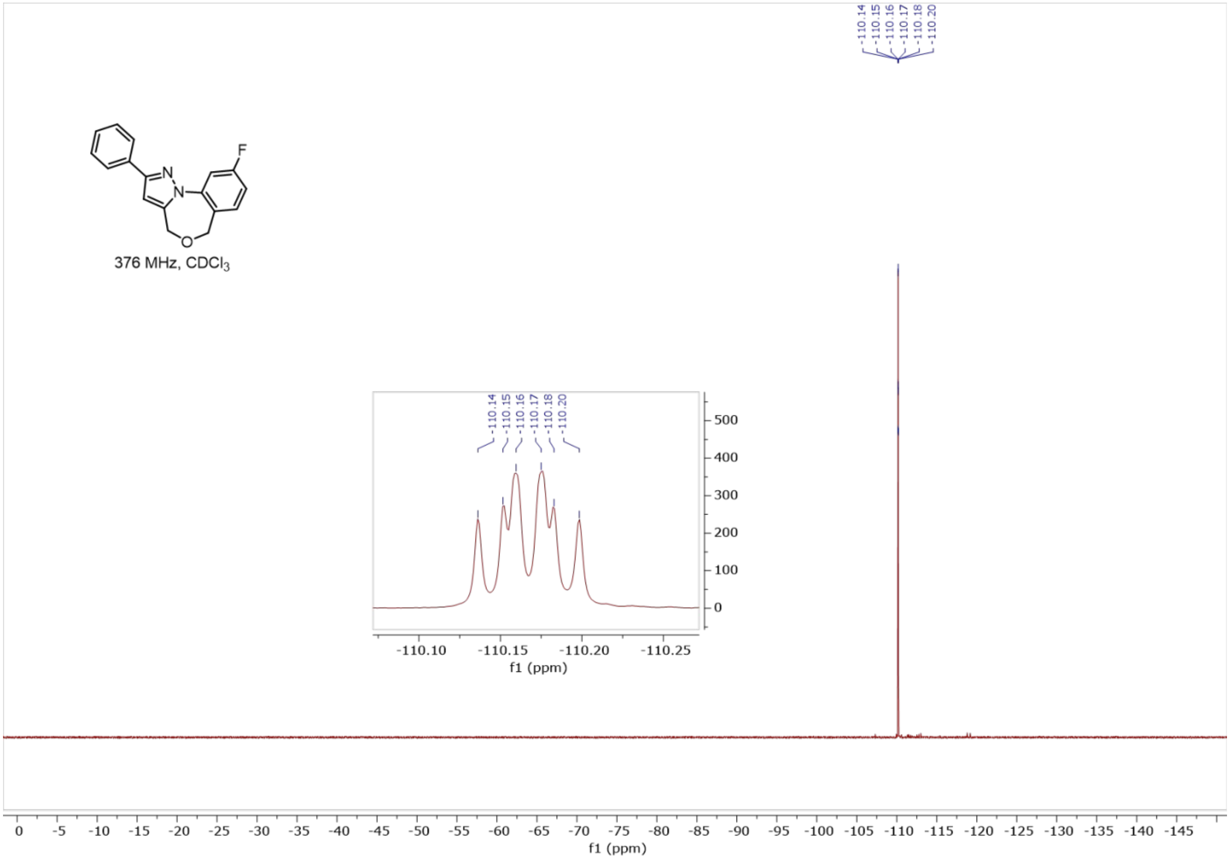
-110.14
-110.15
-110.16
-110.17
-110.18
-110.20
F
N
N
O
376 MHz, CDCl3
f1 (ppm)

7-Fluoro-2,3-diphenyl-4*H*,6*H*-benzo[e]pyrazolo[5,1-c][1,4]oxazepine (**4d**)

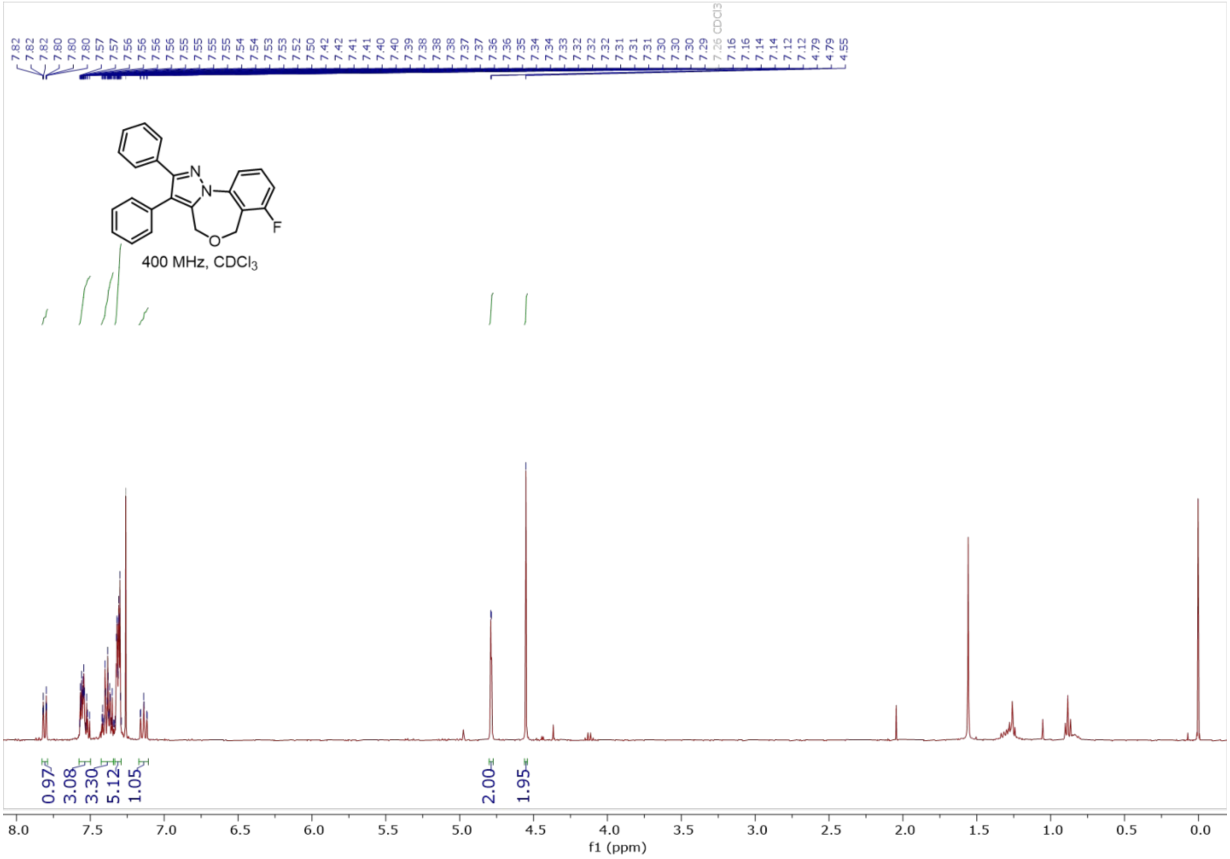

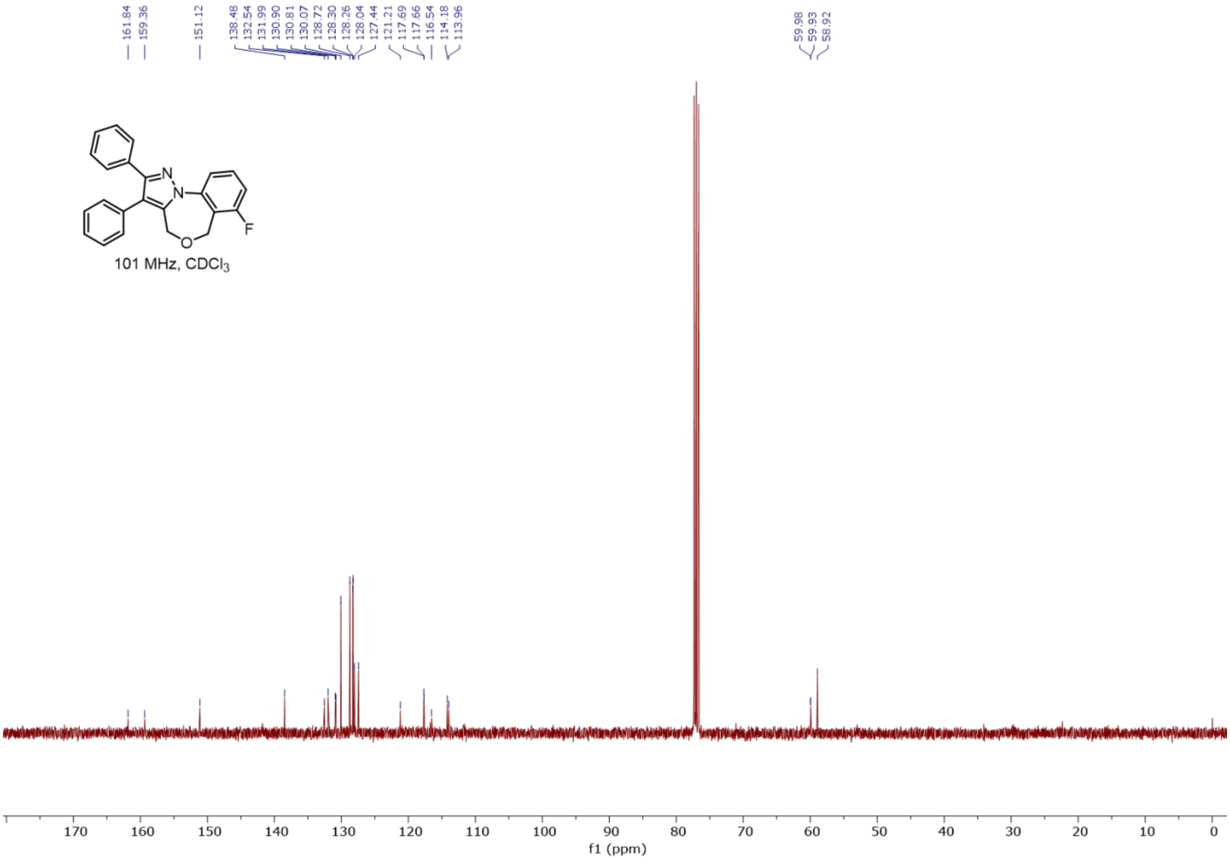

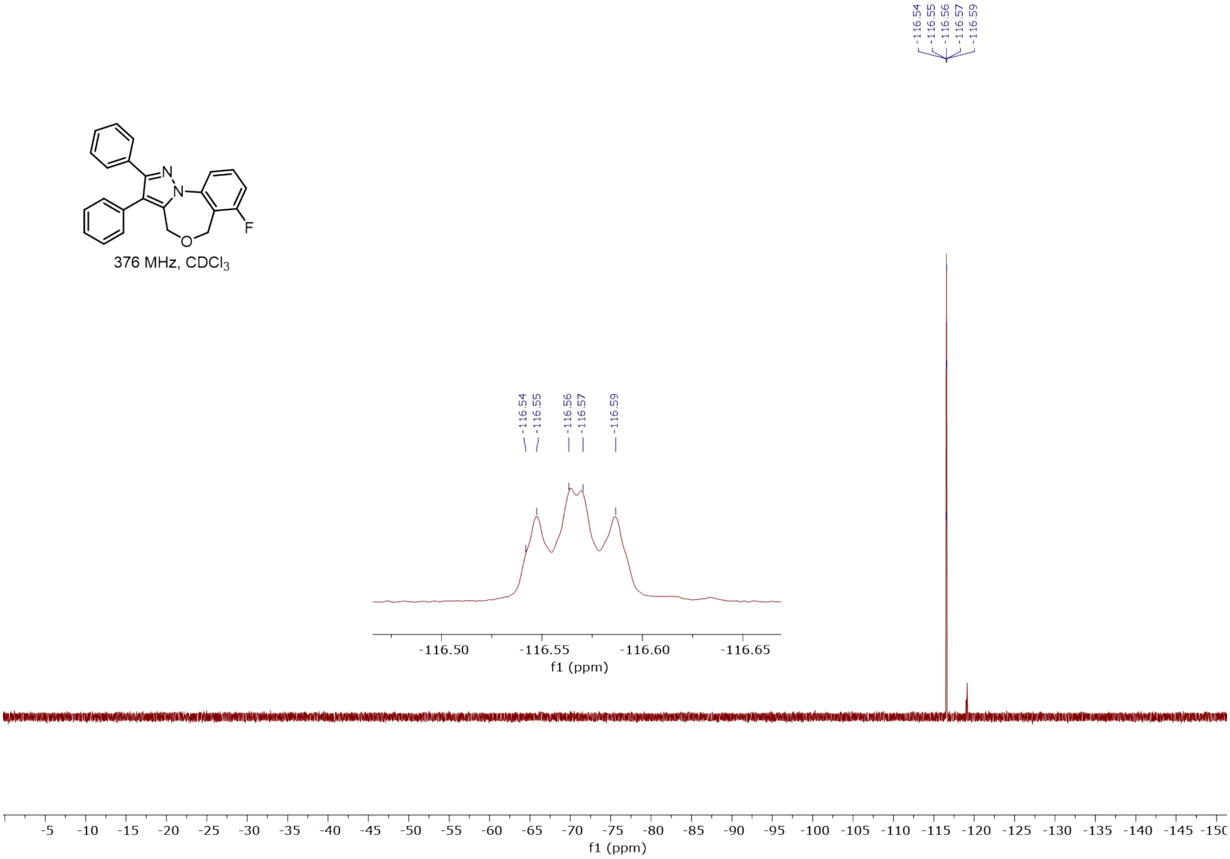

8-Fluoro-2-phenyl-3,3a,4,5-tetrahydro-7*H*-benzo[c]pyrazolo[1,5-e][1,5]oxazocine (**4e**)

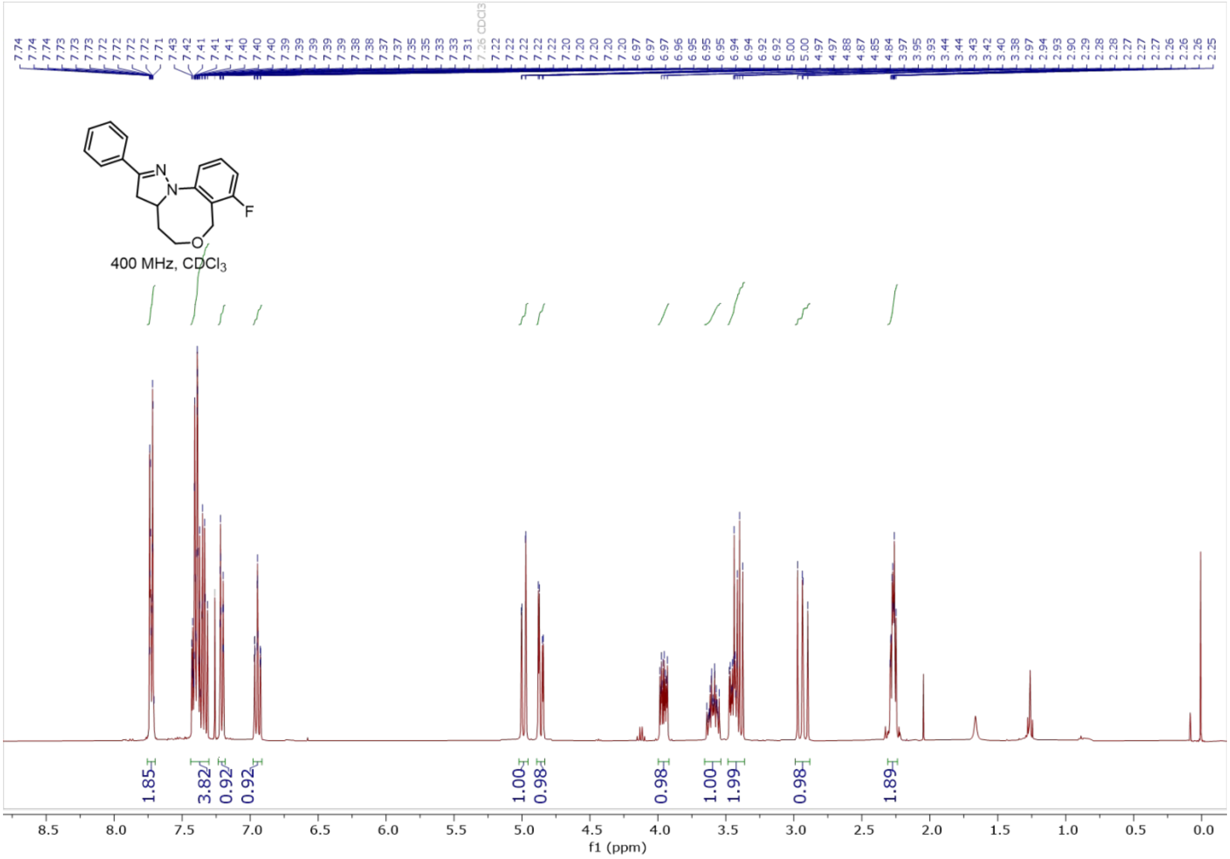

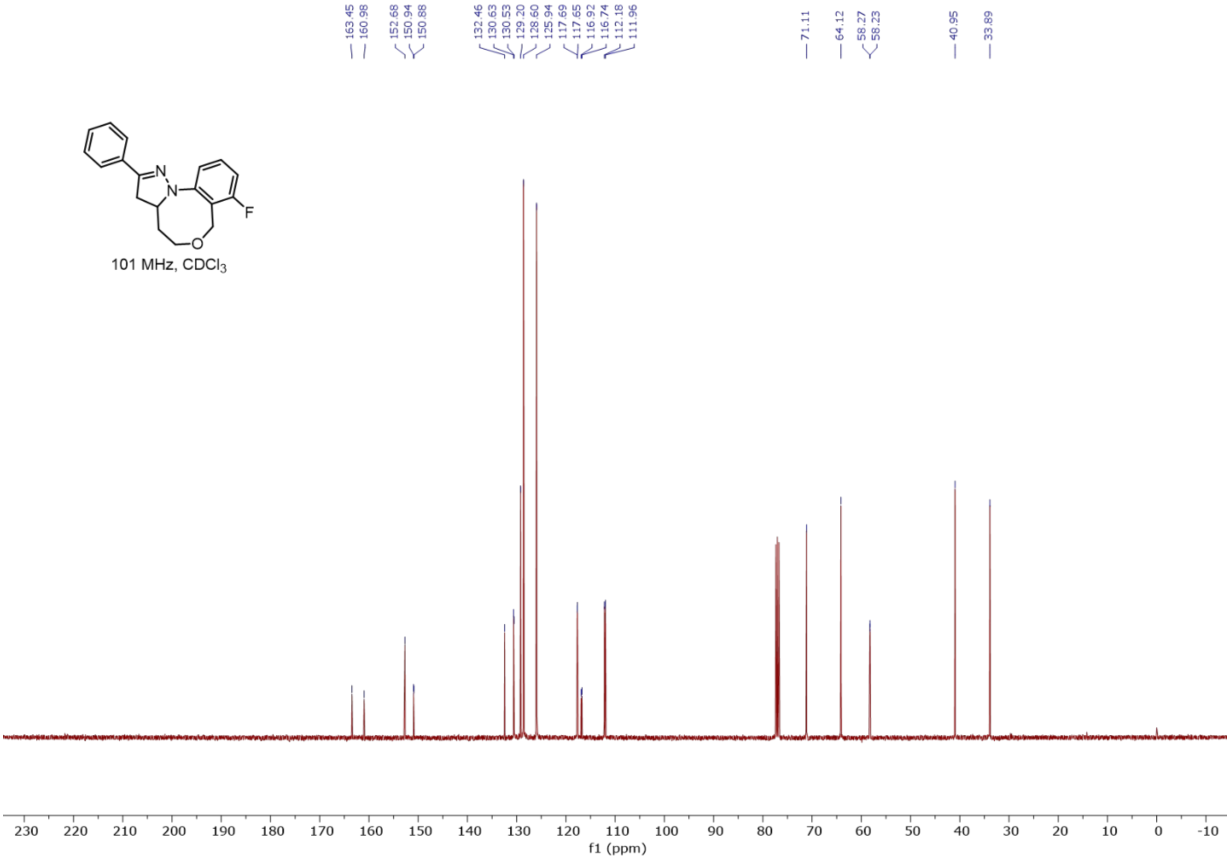
163.45
160.98
152.68
150.94
150.88
132.46
130.63
130.53
129.20
128.60
125.94
117.69
117.65
116.92
116.74
112.18
111.96
71.11
64.12
58.27
58.23
40.95
33.89
N
N
F
O
101 MHz, CDCl3
230 220 210 200 190 180 170 160 150 140 130 120 110 100 90 80 70 60 50 40 30 20 10 0 -10
f1 (ppm)

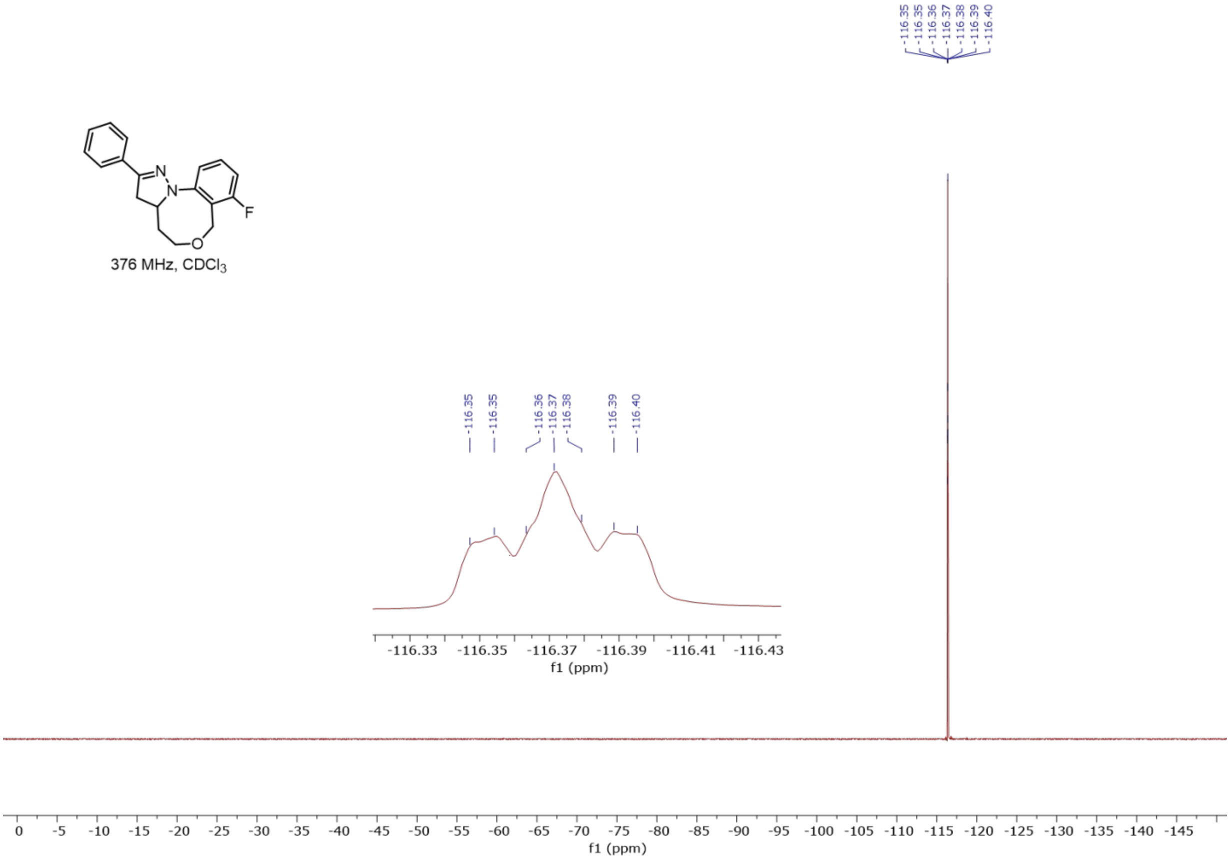
-116.35
-116.35
-116.36
-116.37
-116.38
-116.39
-116.40
N
N
F
O
376 MHz, CDCl3
-116.33 -116.35 -116.37 -116.39 -116.41 -116.43
f1 (ppm)
0 -5 -10 -15 -20 -25 -30 -35 -40 -45 -50 -55 -60 -65 -70 -75 -80 -85 -90 -95 -100 -105 -110 -115 -120 -125 -130 -135 -140 -145
f1 (ppm)

7-Fluoro-2-phenyl-3a,4-dihydro-3*H*,6*H*-benzo[e]pyrazolo[5,1-c][1,4]oxazepane (**4f**)

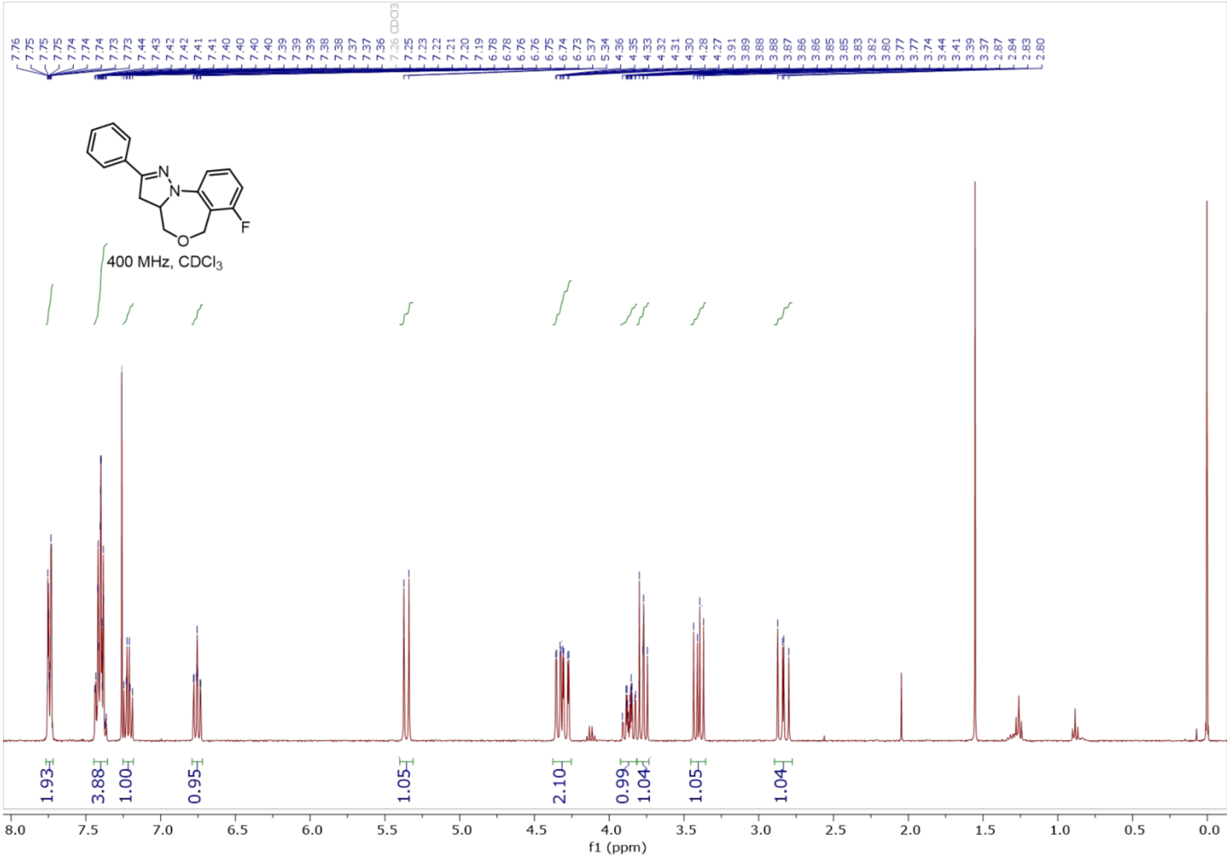

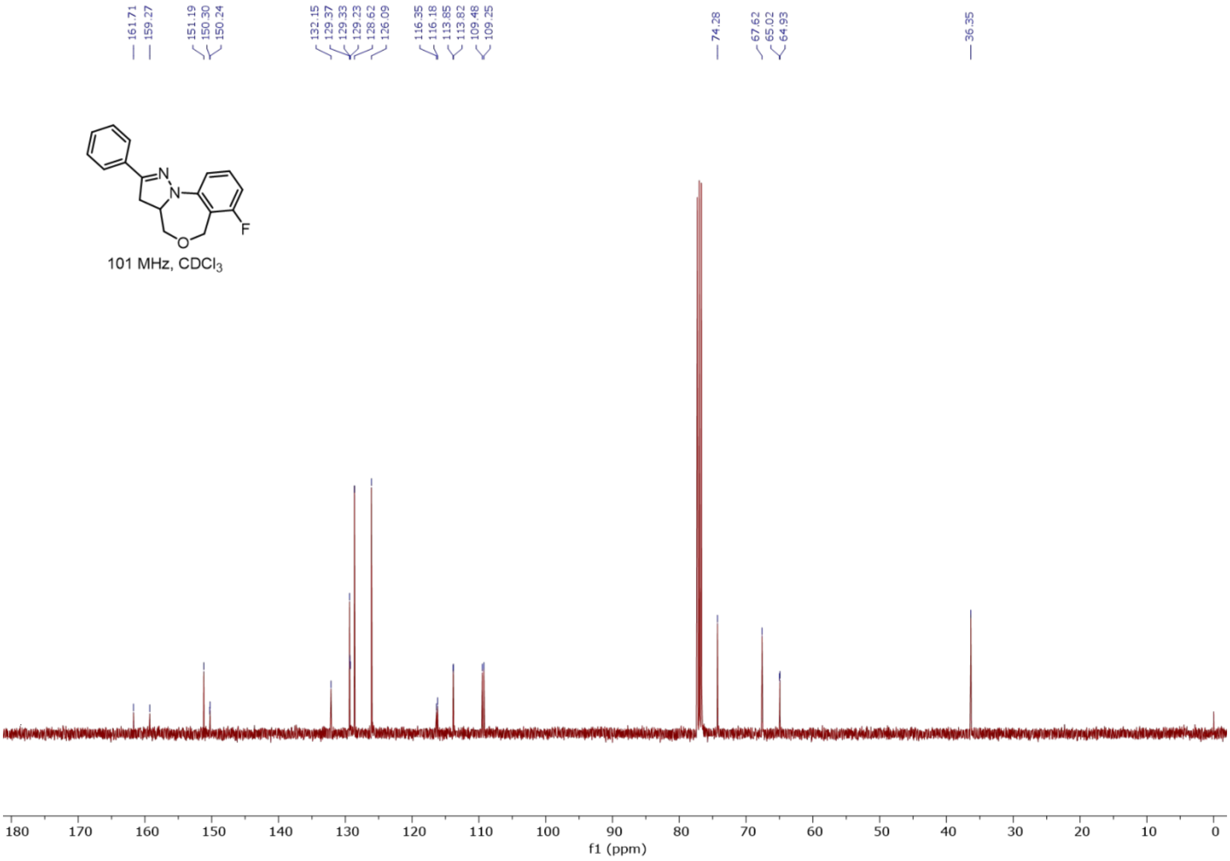

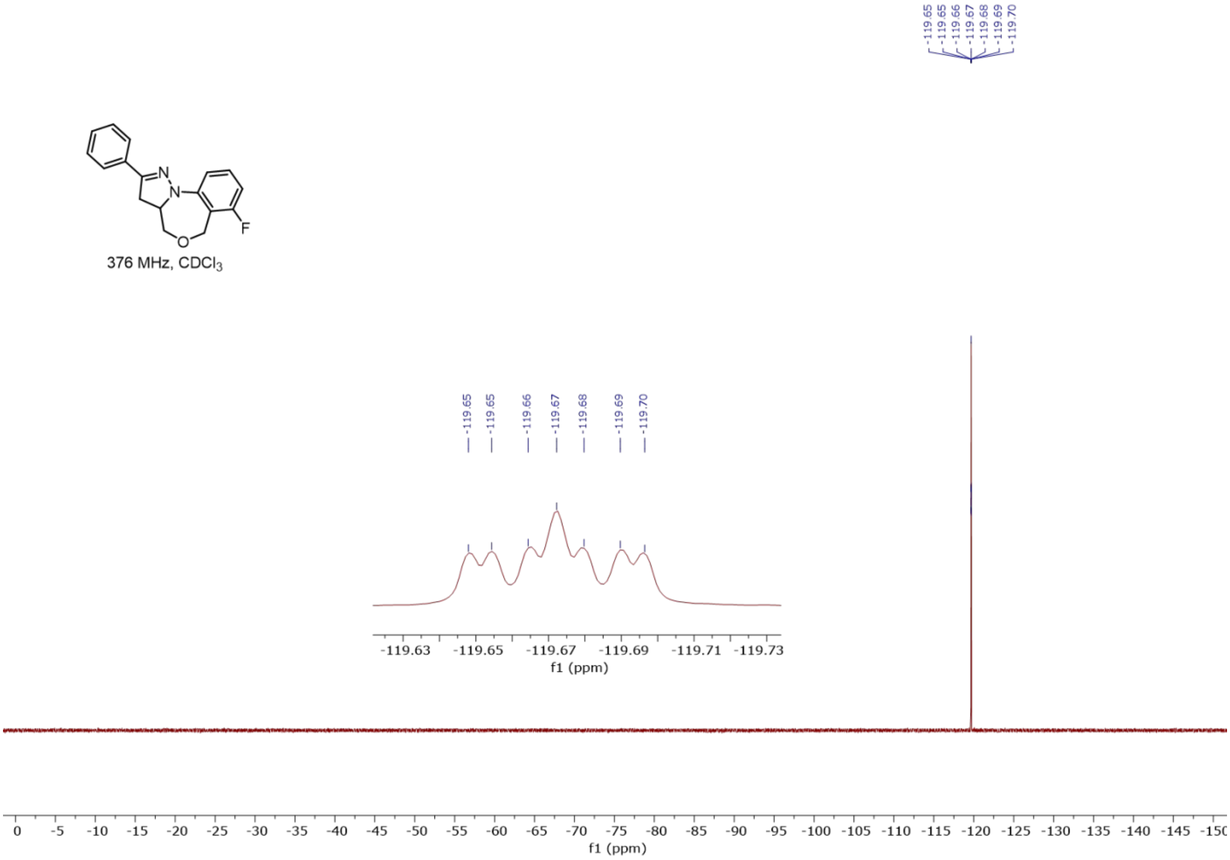

7-Fluoro-2-phenyl-3a,4,5,6-tetrahydro-3*H*-benzo[f]pyrazolo[1,5-a][1,4]diazepine (**4g**)

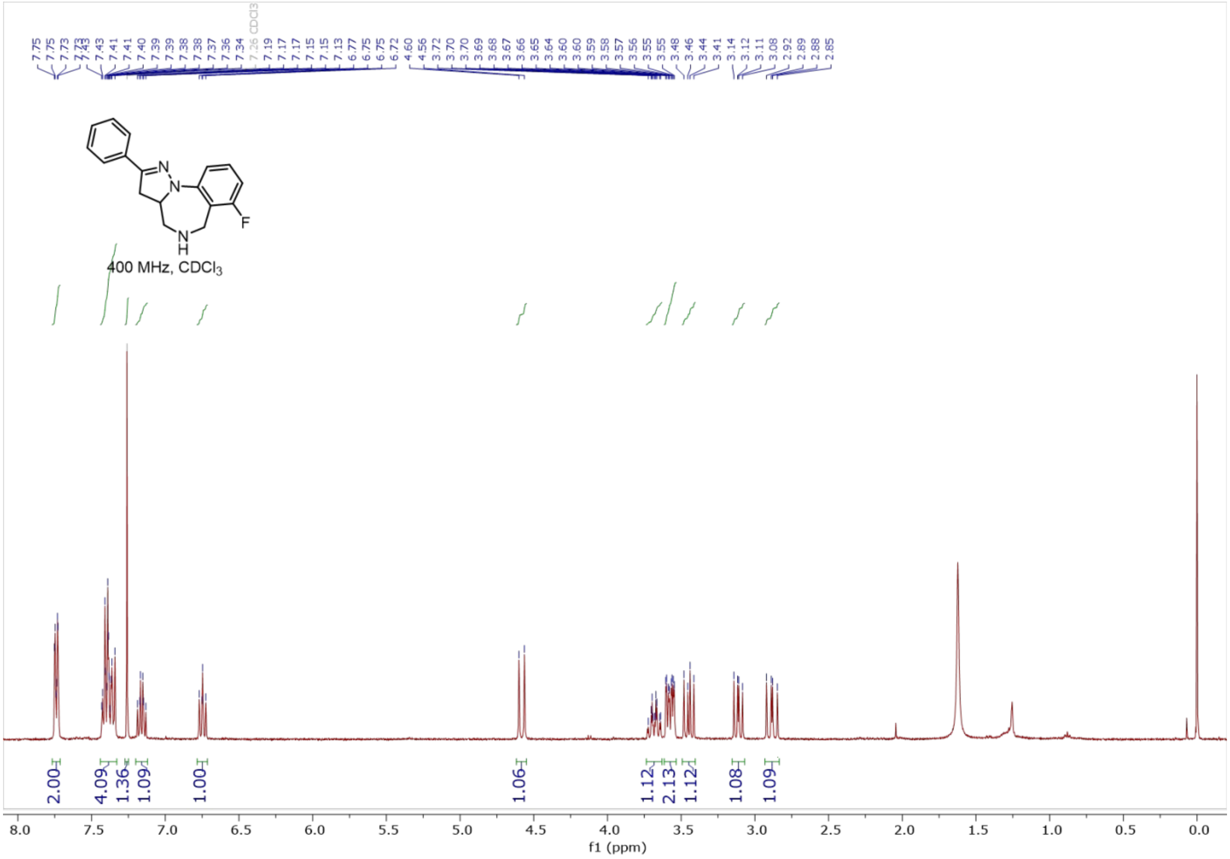

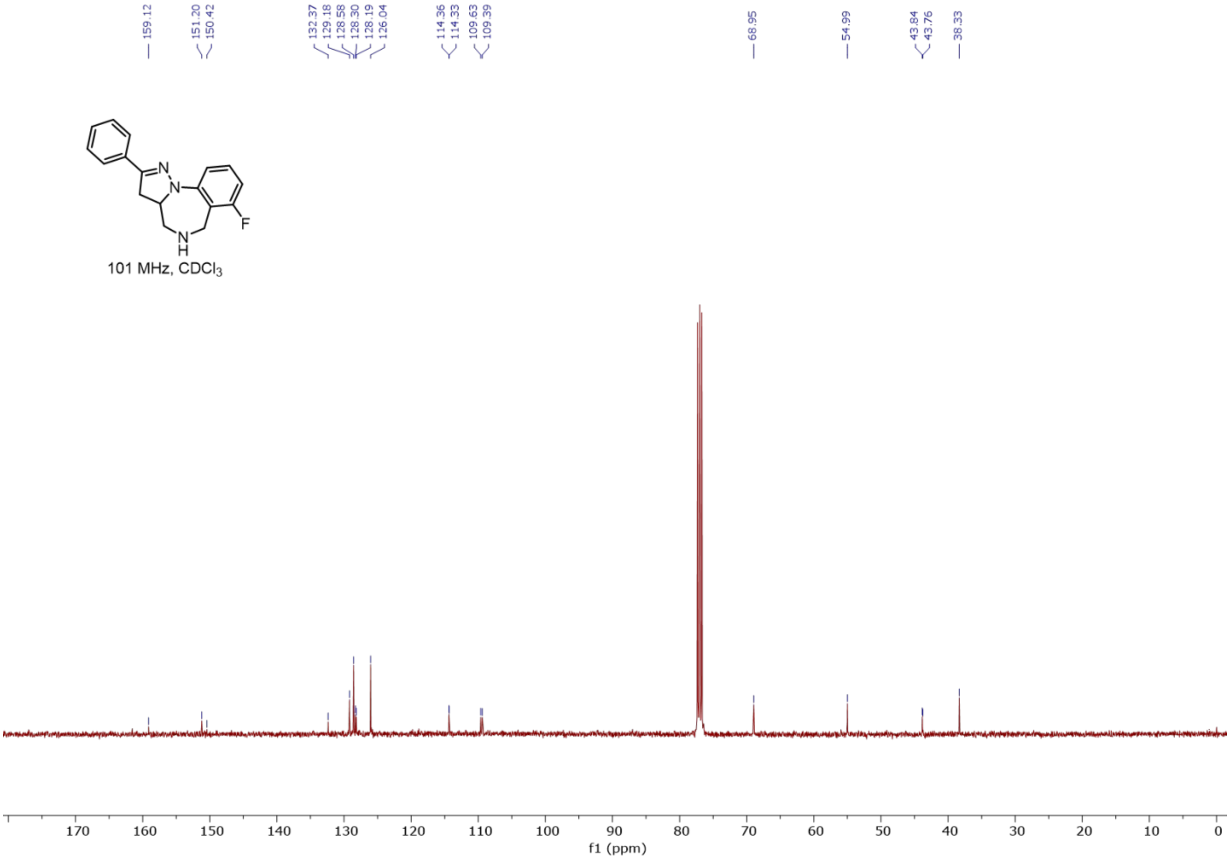

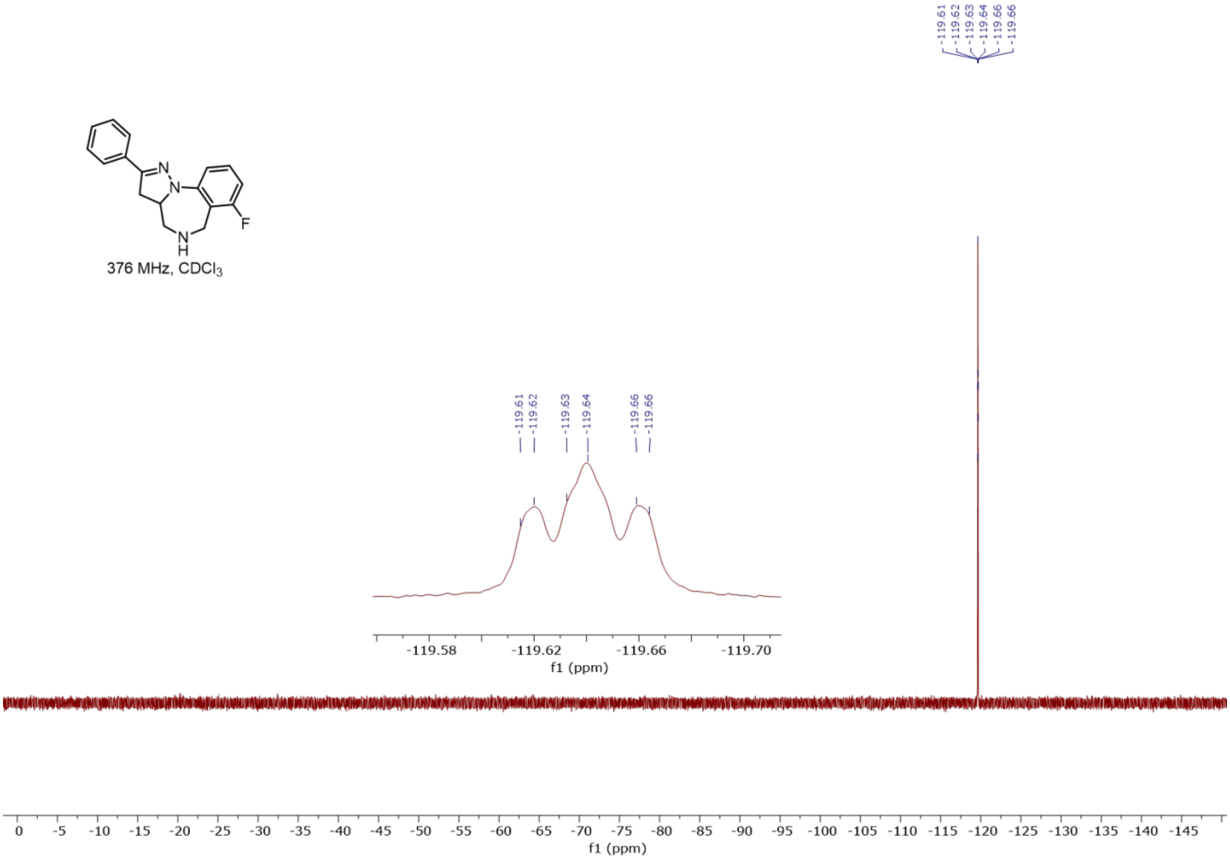

7-Fluoro-2,3-diphenyl-3a,4-dihydro-3*H*,6*H*-benzo[e]pyrazolo[5,1-c][1,4]oxazepine (**4h**)

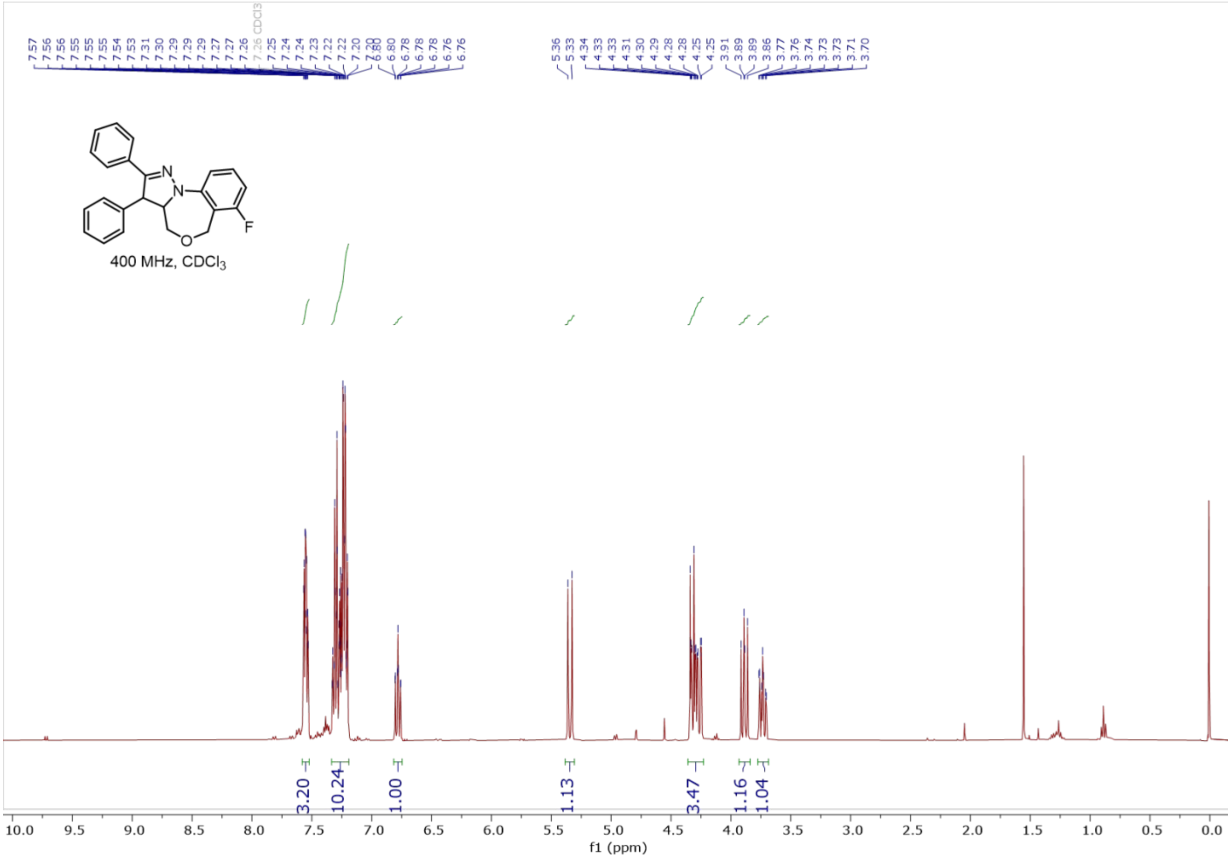
400 MHz, CDCl3
3.20
10.24
1.00
1.13
3.47
1.16
1.04
f1 (ppm)

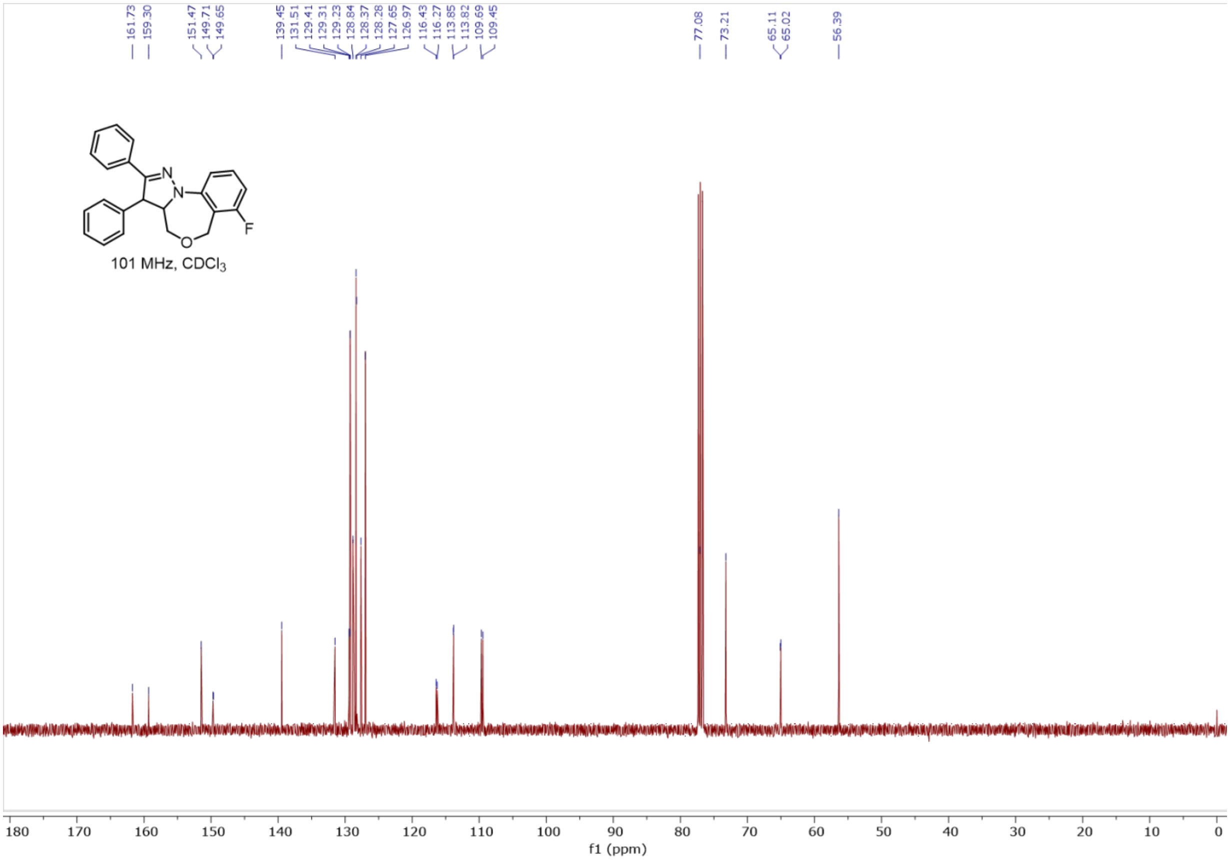
101 MHz, CDCl3
161.73
159.30
151.47
149.71
149.65
139.45
77.08
73.21
65.11
65.02
56.39
f1 (ppm)

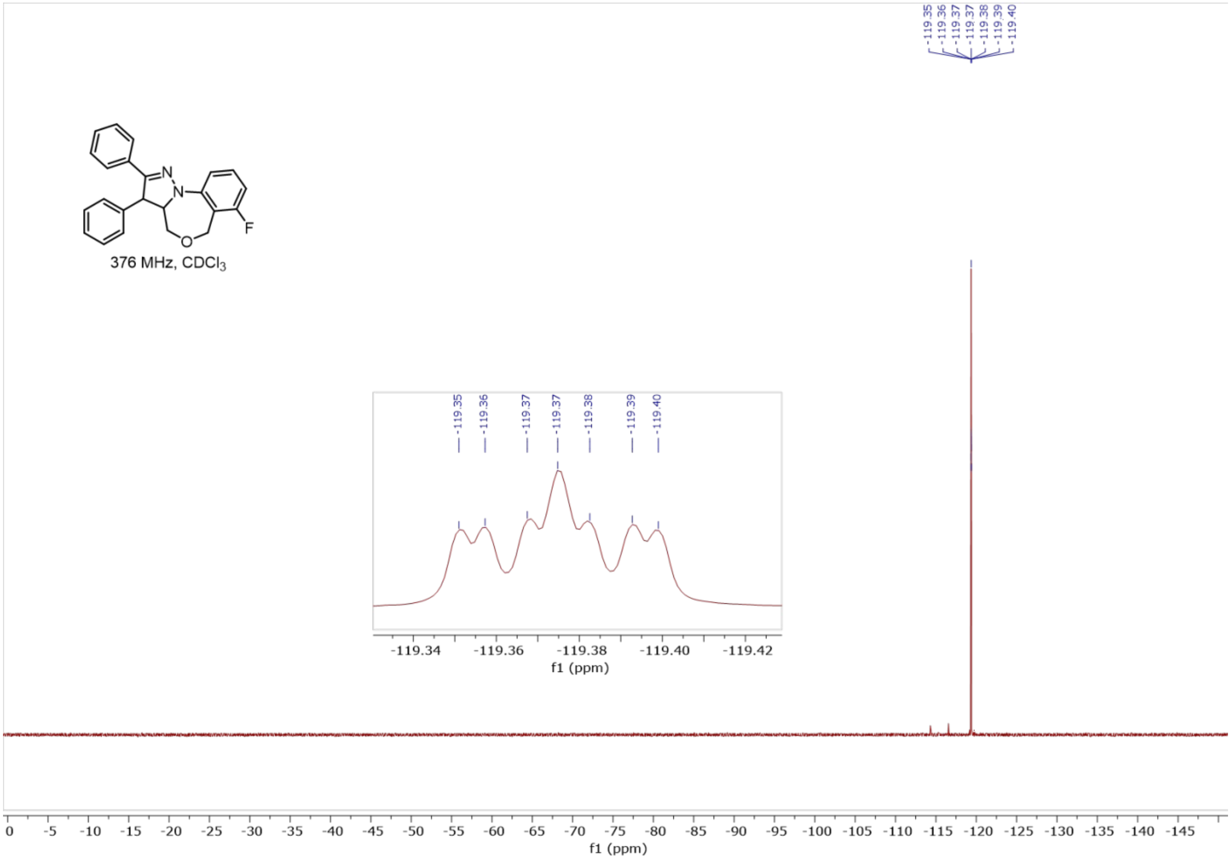

4-Allyl-6-fluoro-3-phenyl-4,5-dihydro-1*H*-benzo[f][1,2,4]triazepine (**5a**)

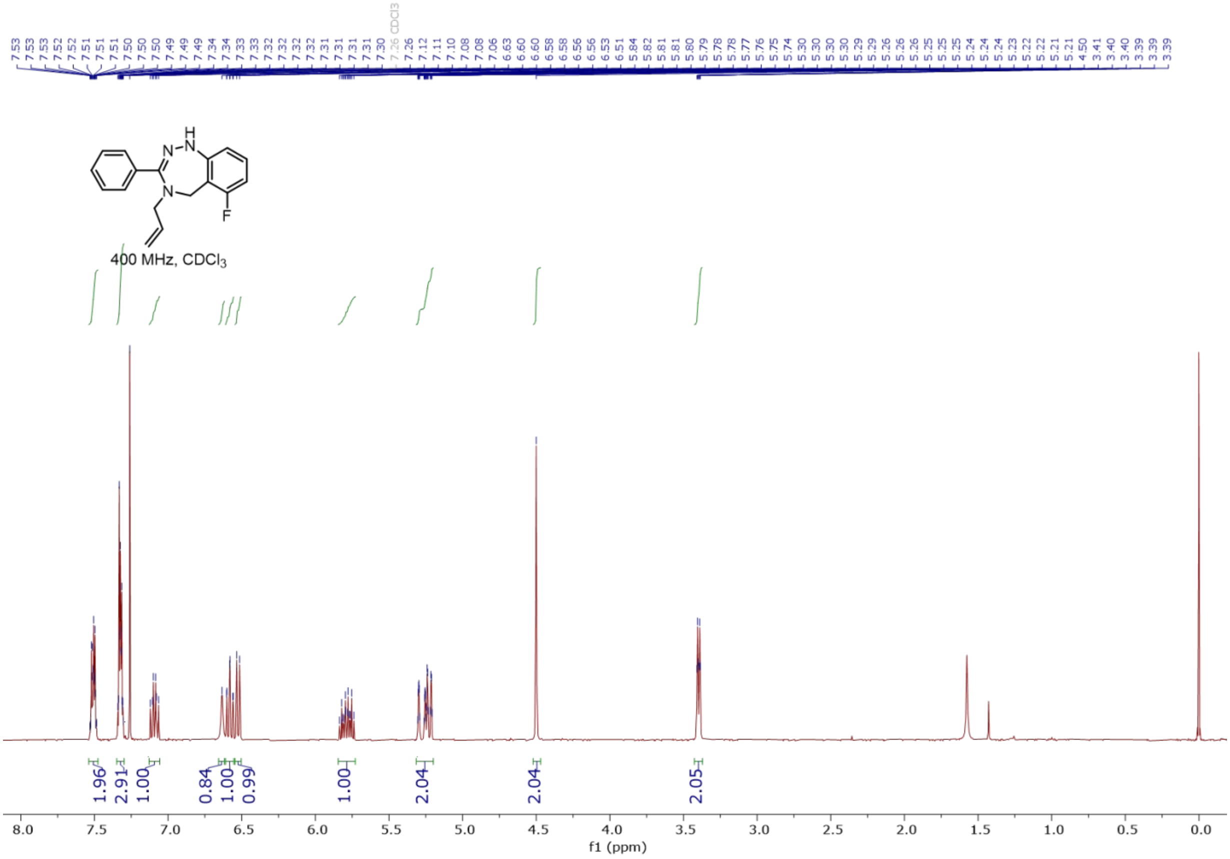

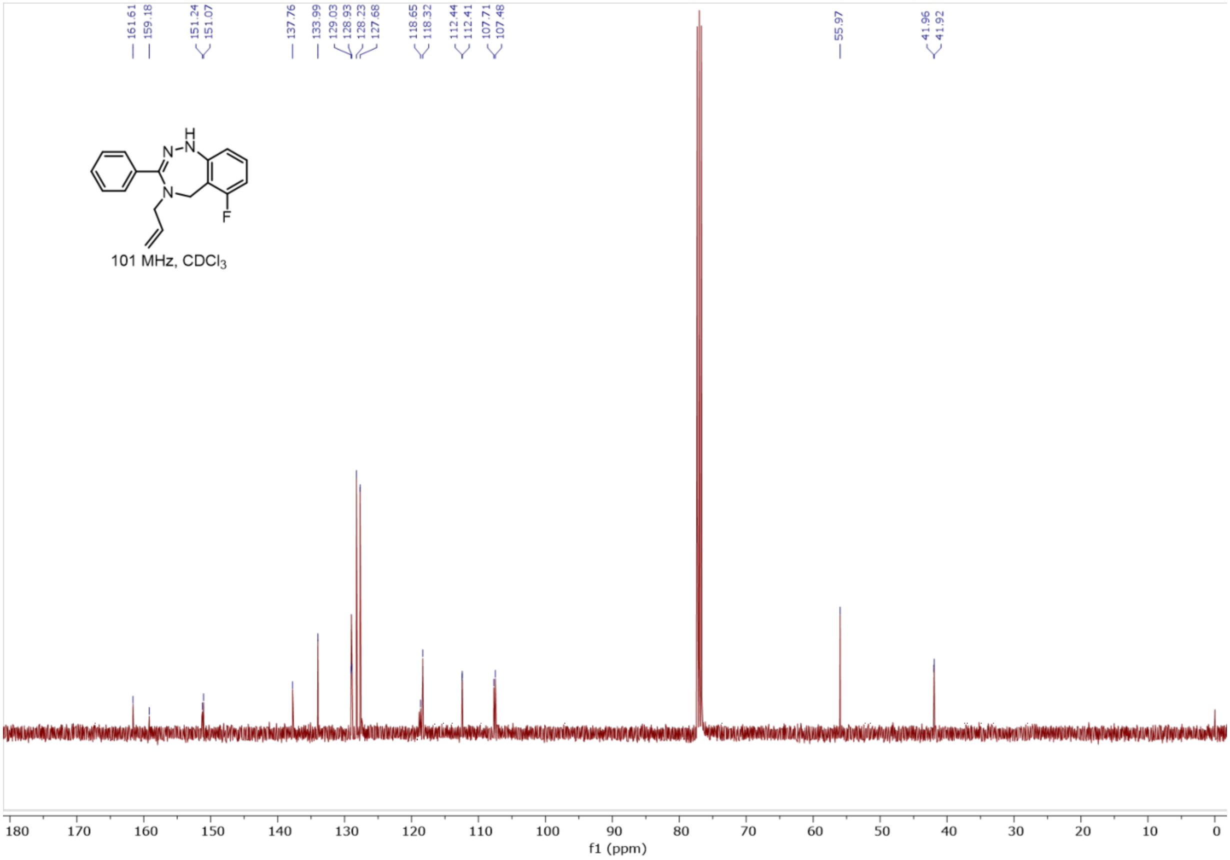

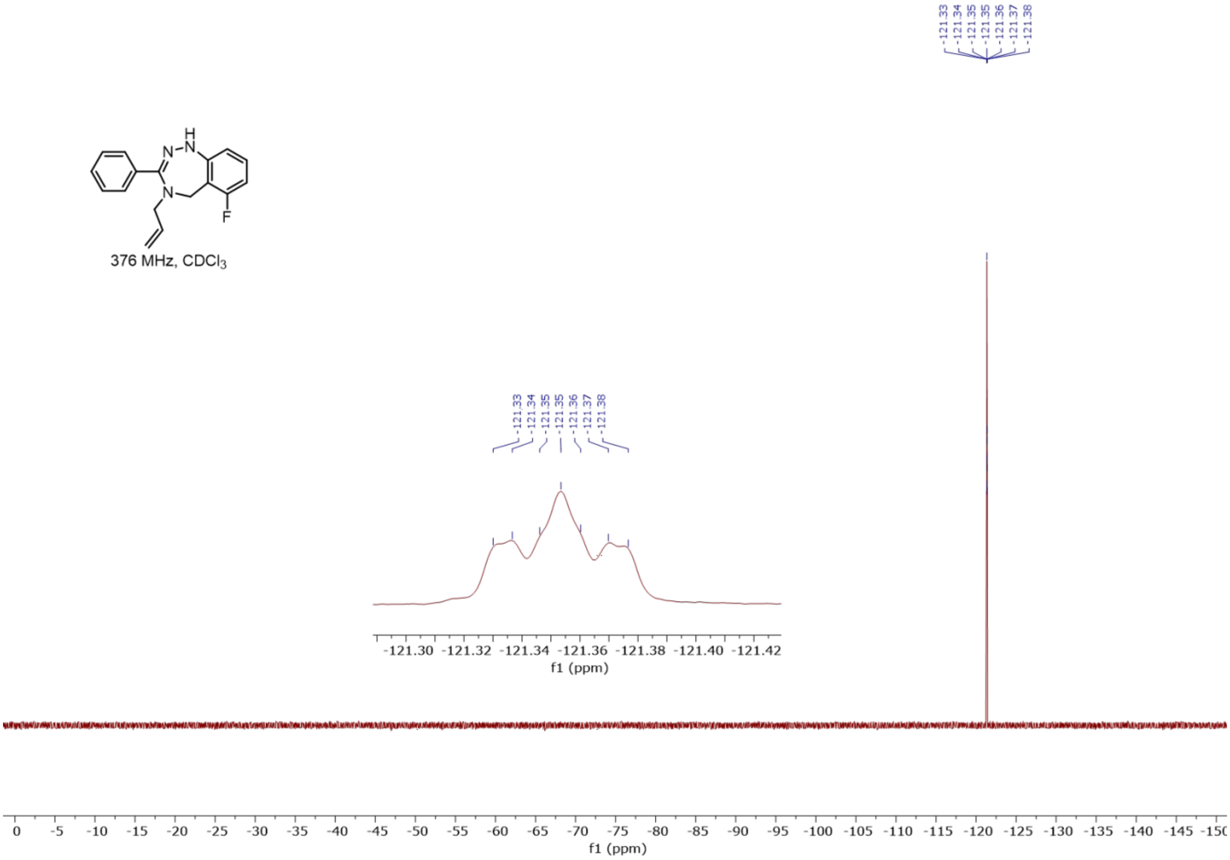

6-Fluoro-4-isobutyl-3-phenyl-4,5-dihydro-1*H*-benzo[*f*][1,2,4]triazepine (**5b**)

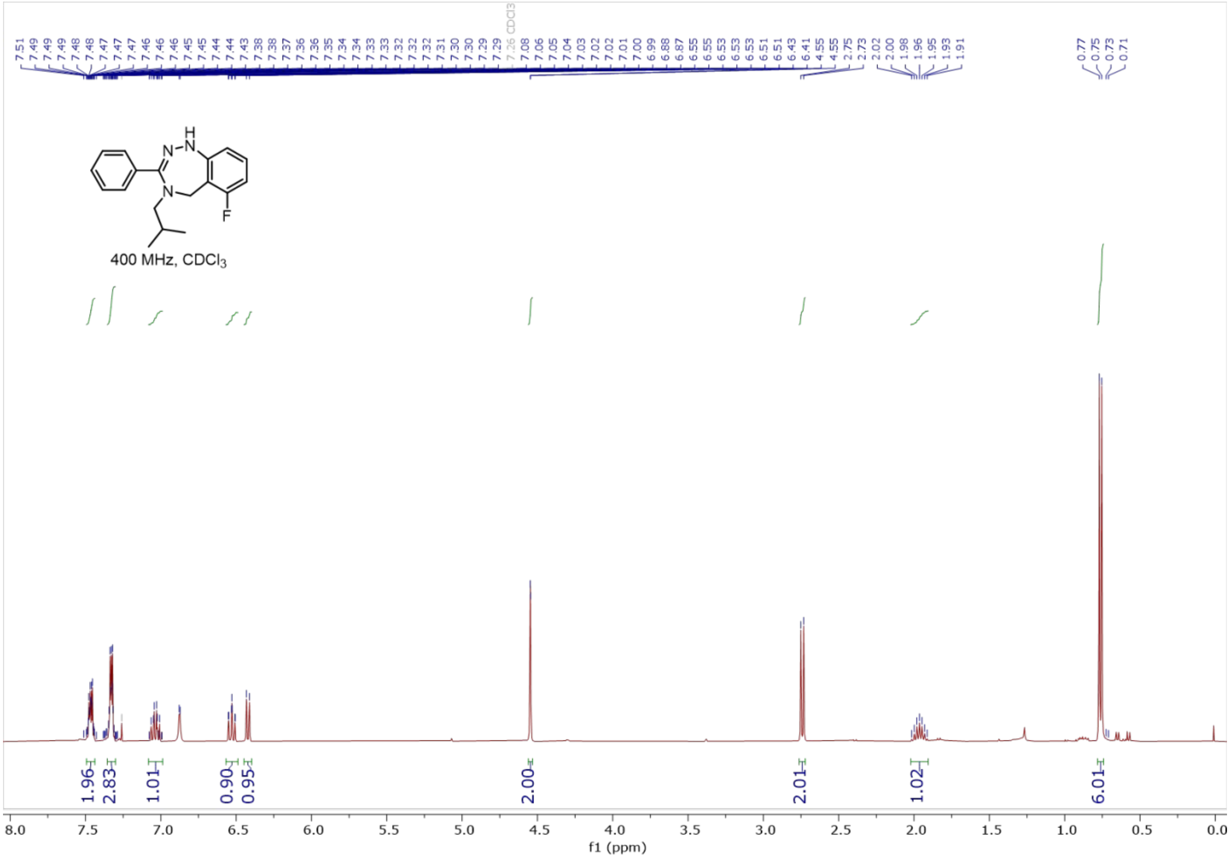

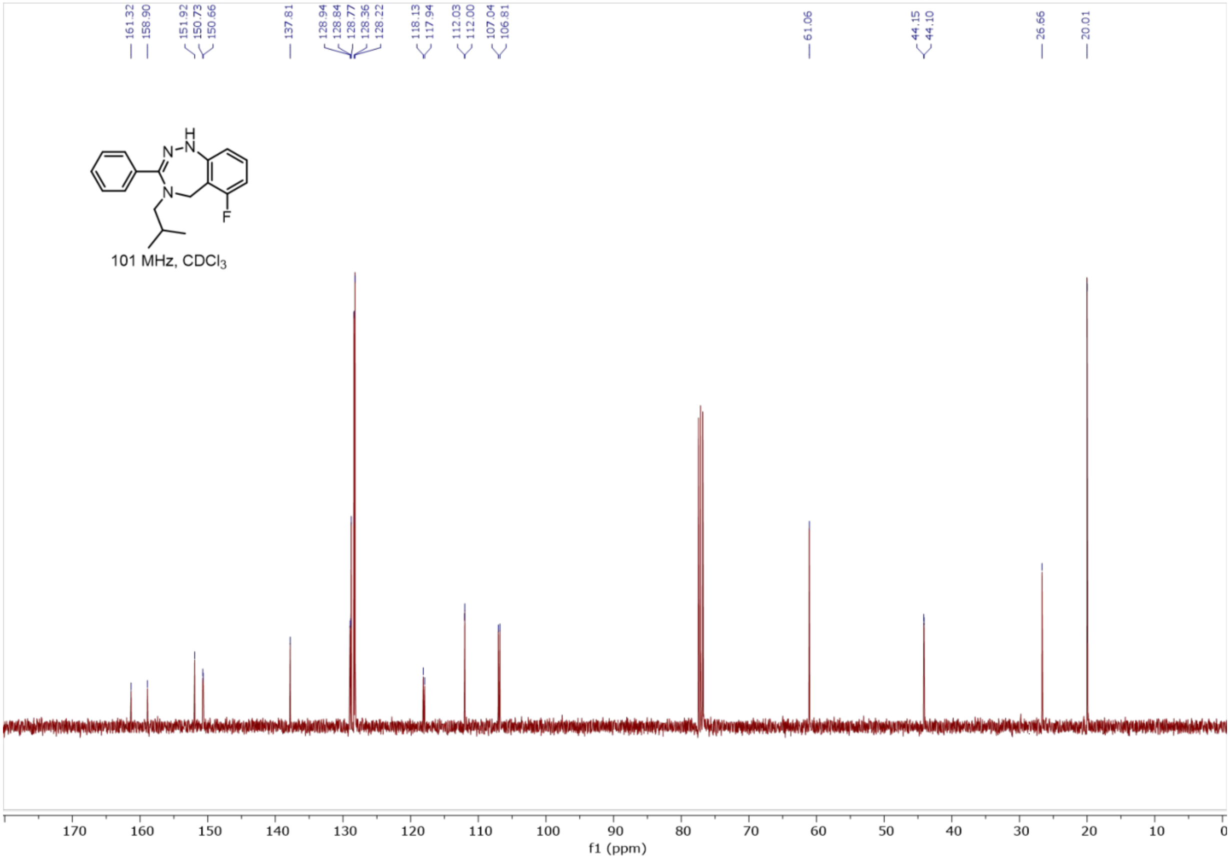

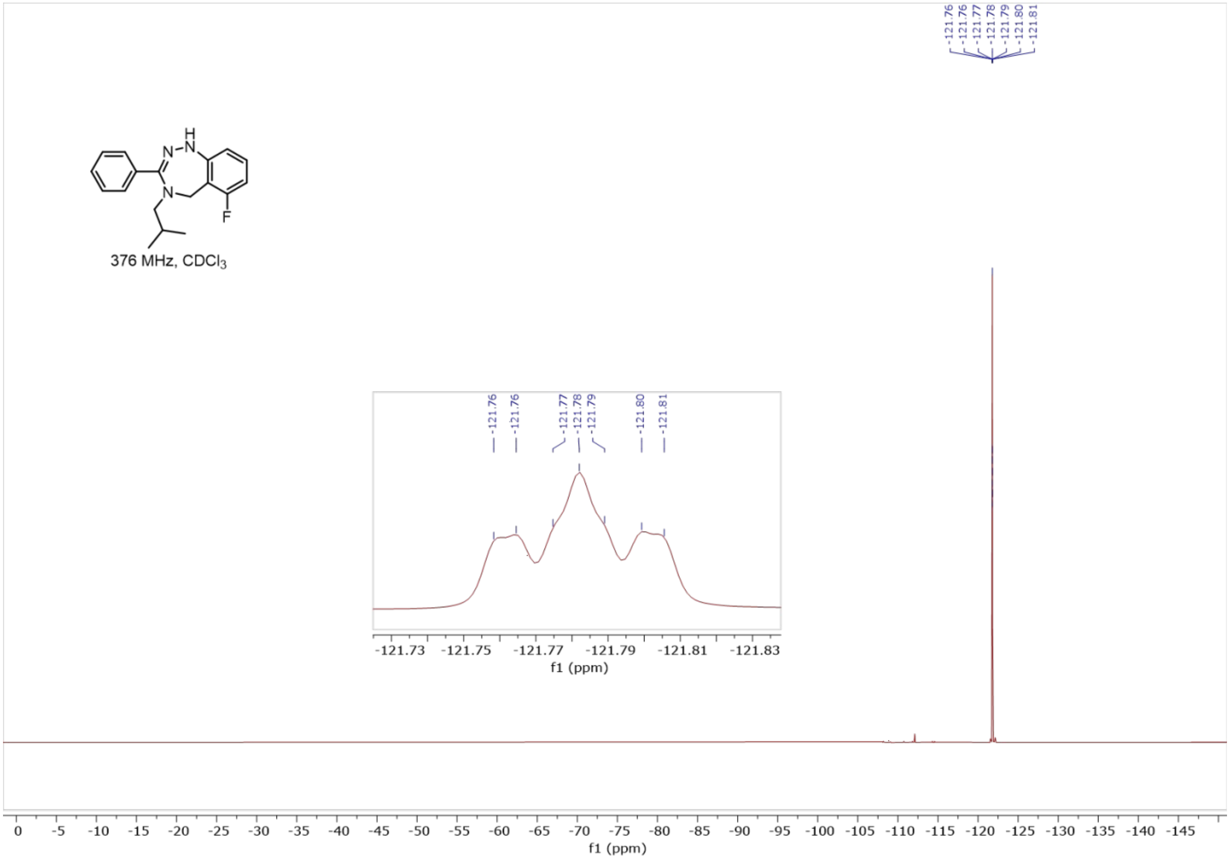

Methyl 2-(6-fluoro-3-phenyl-1,5-dihydro-4*H*-benzo[f][1,2,4]triazepin-4-yl)acetate (**5c**)

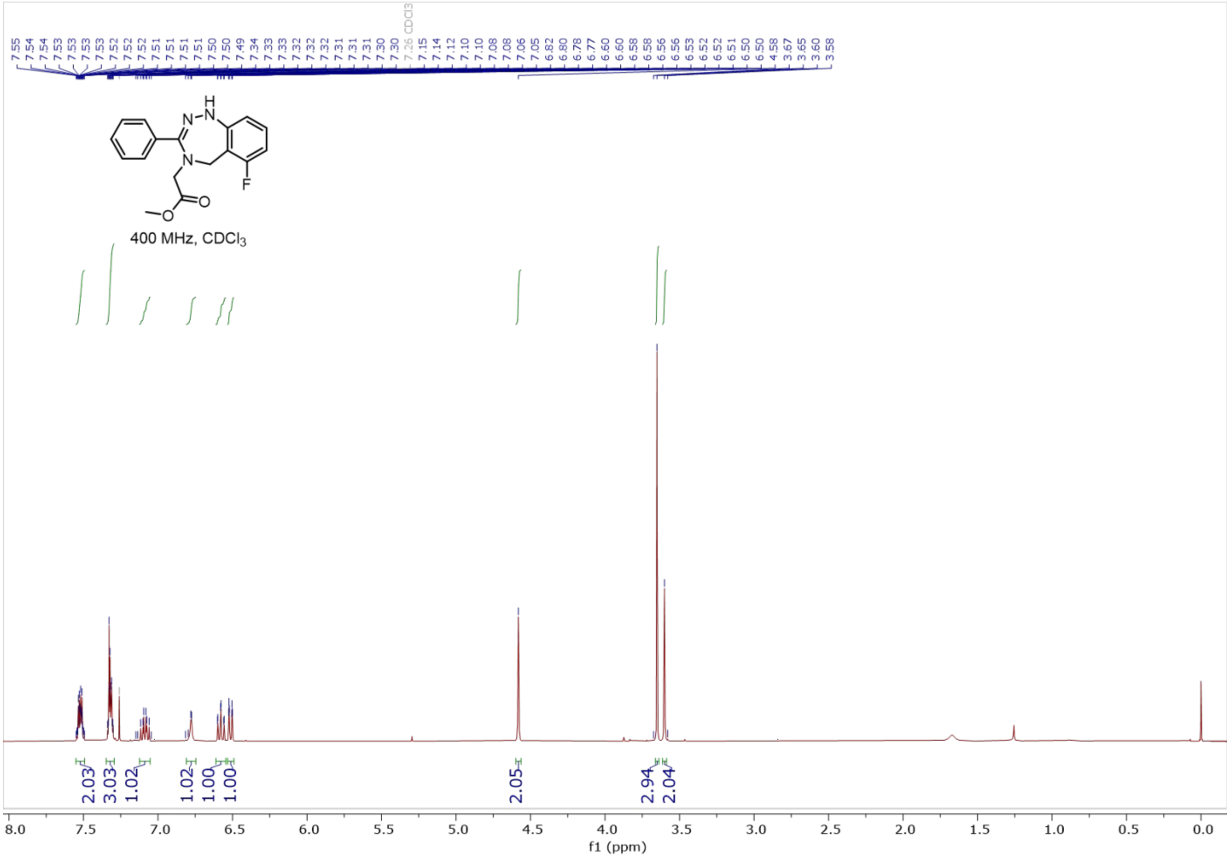

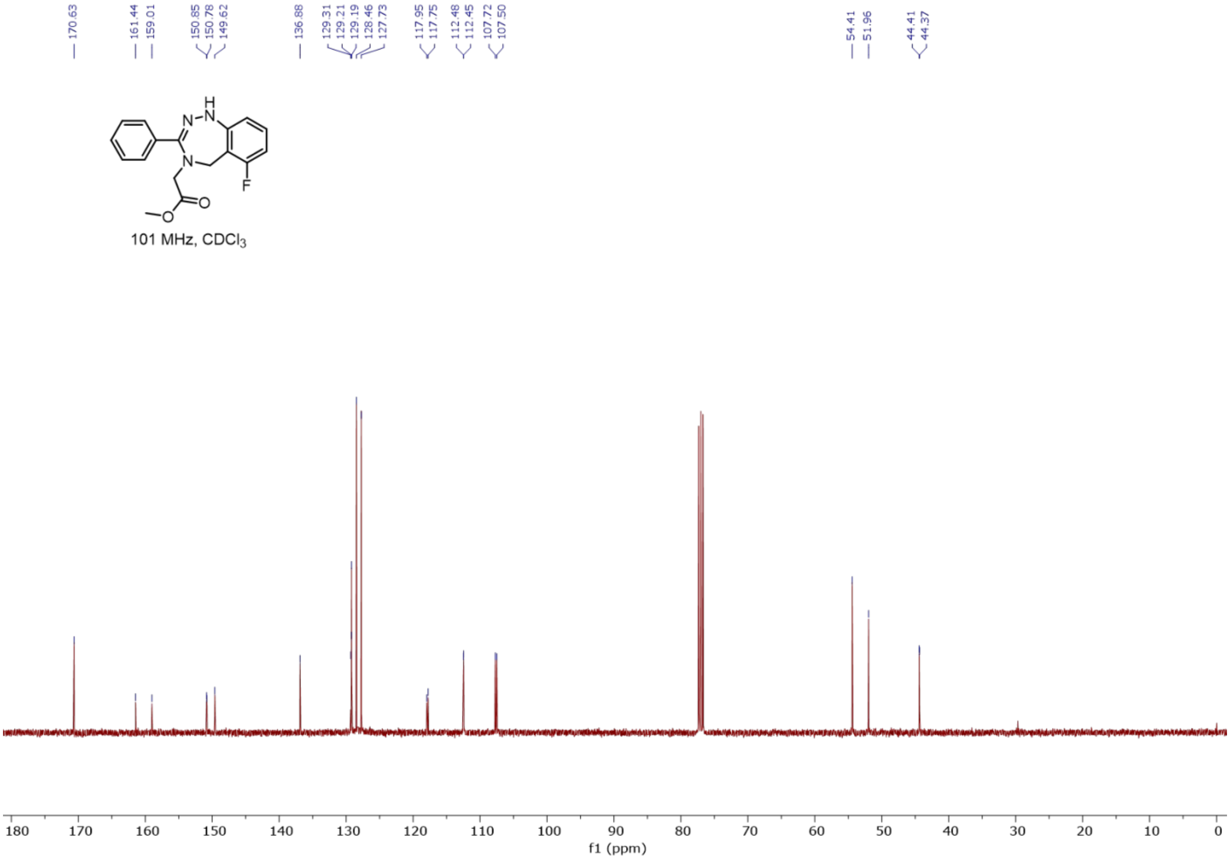

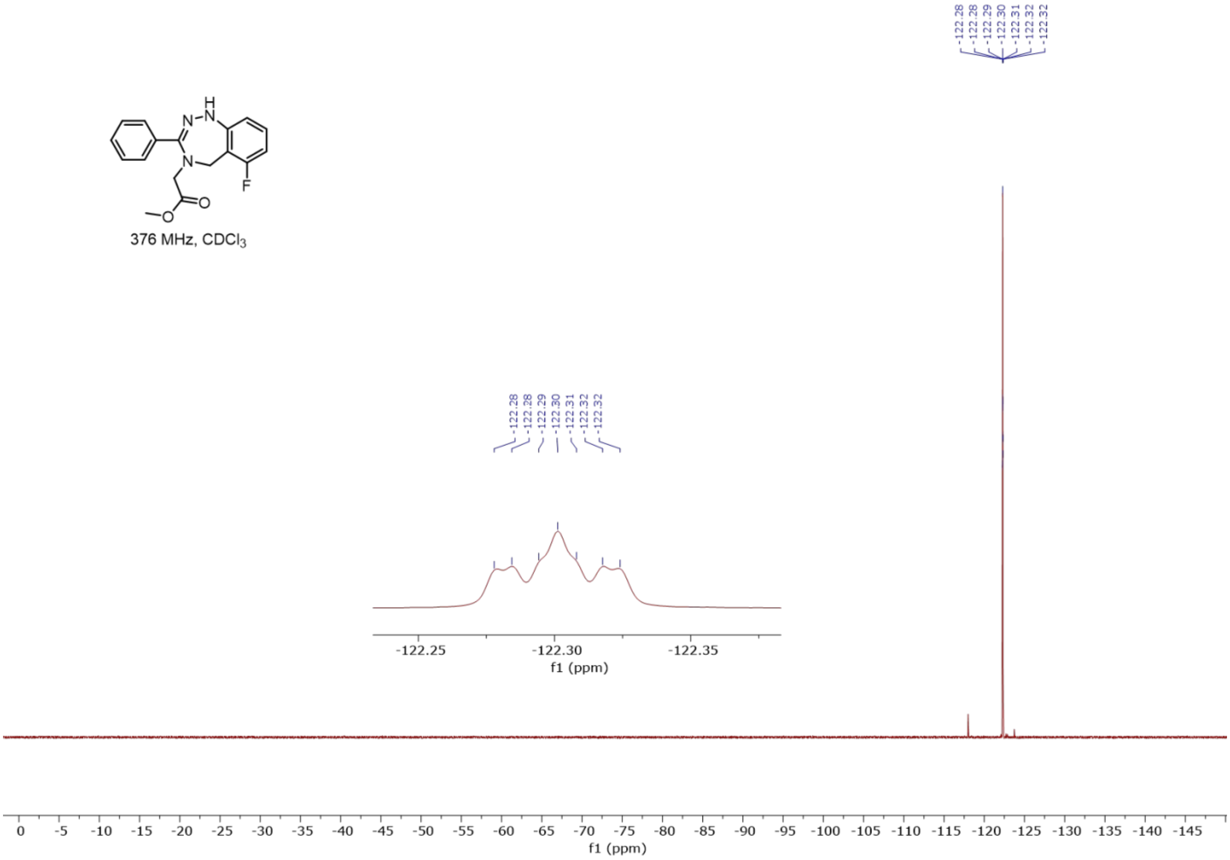

4-Cyclopentyl-6-fluoro-3-phenyl-4,5-dihydro-1*H*-benzo[*f*][1,2,4]triazepine (**5d**)

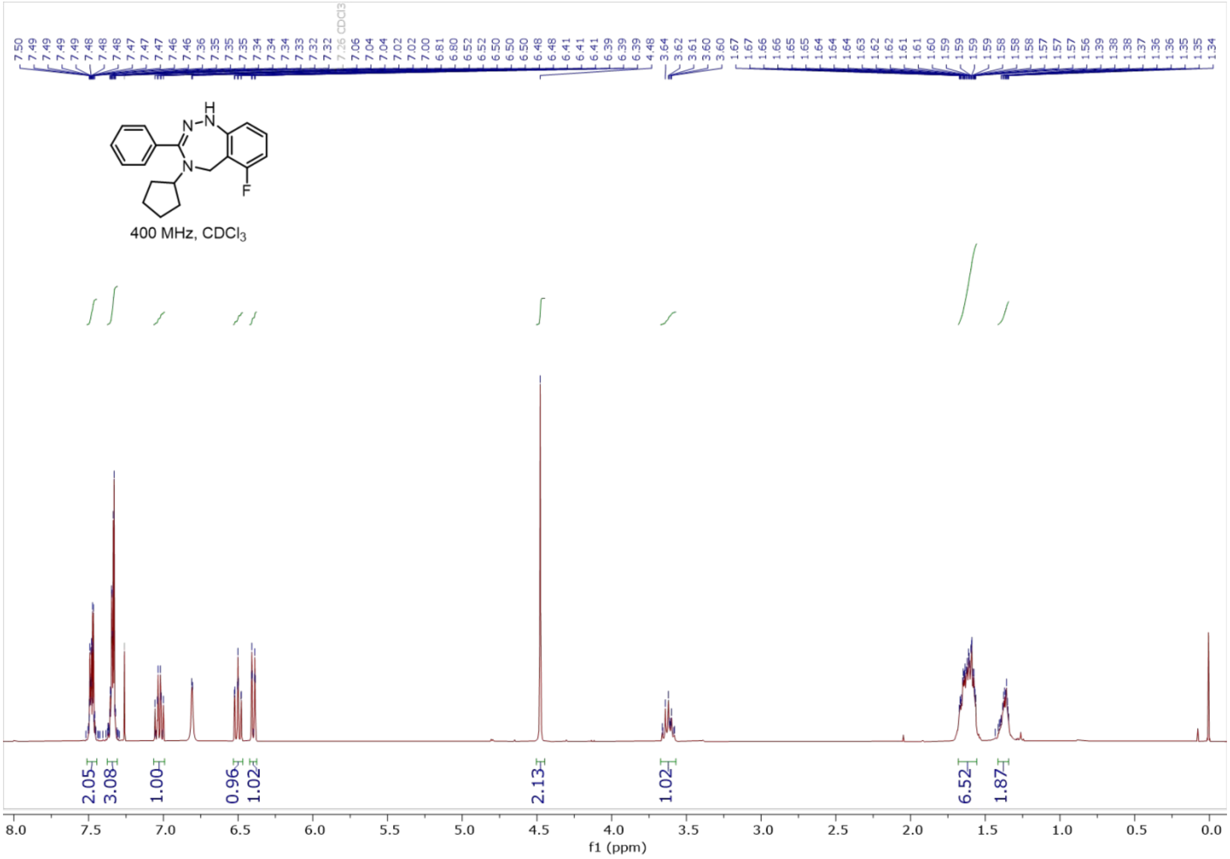
400 MHz, CDCl3
f1 (ppm)

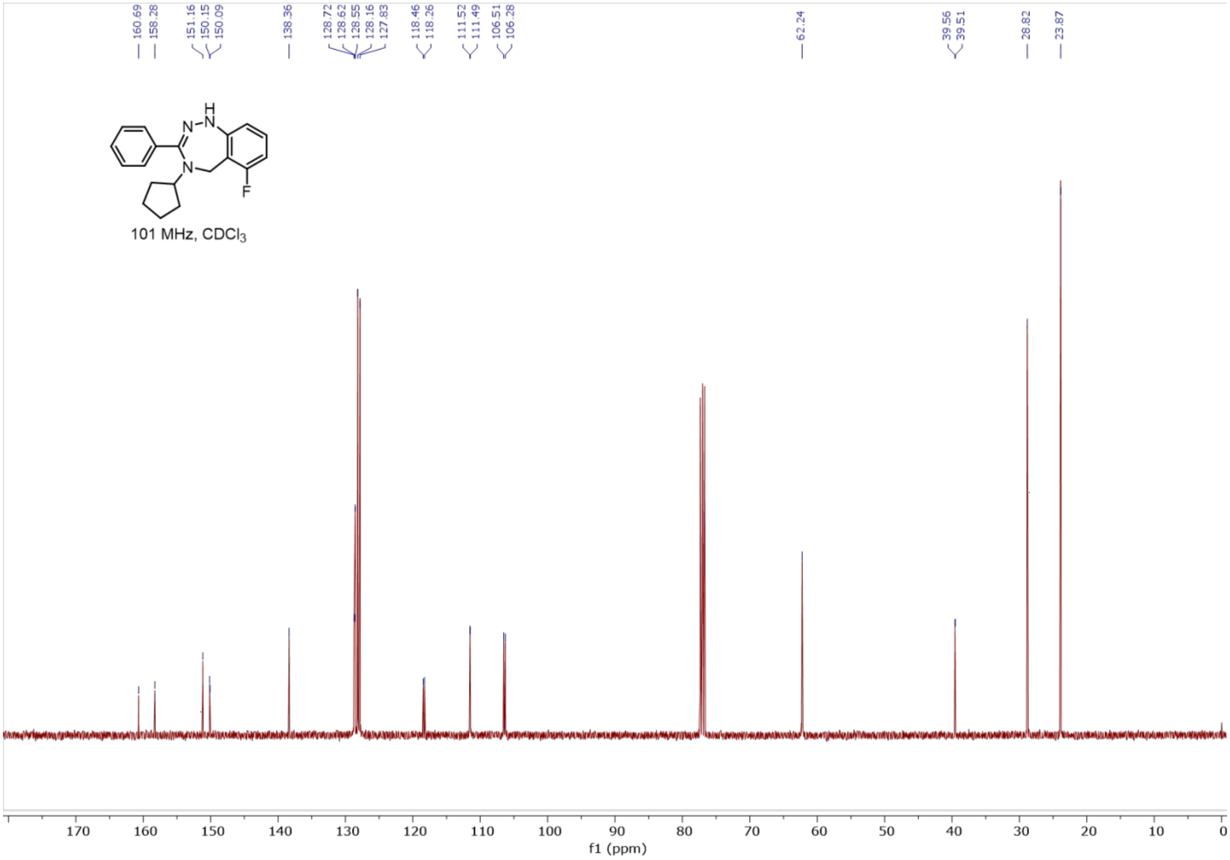
101 MHz, CDCl3
f1 (ppm)

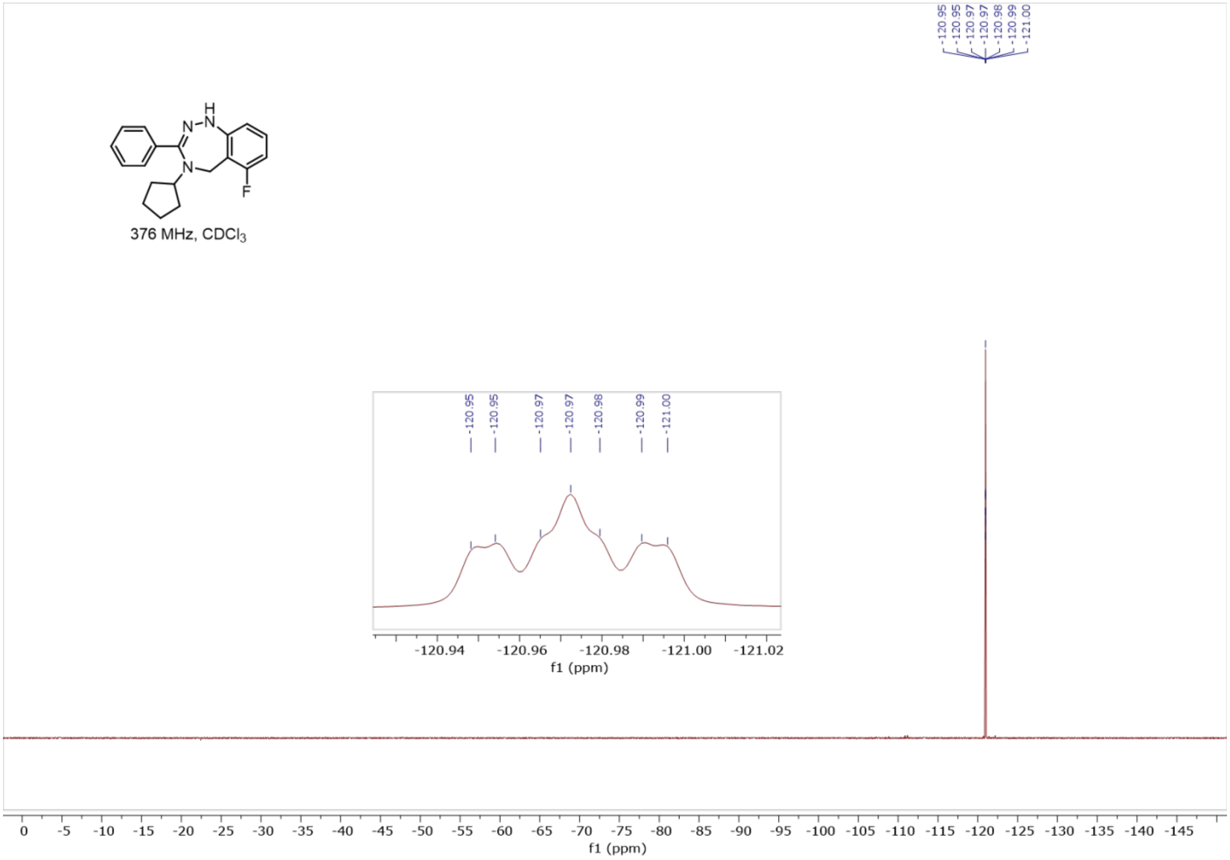

6-Fluoro-4-(4-methoxybenzyl)-3-phenyl-4,5-dihydro-1*H*-benzo[*f*][1,2,4]triazepine (**5g**)

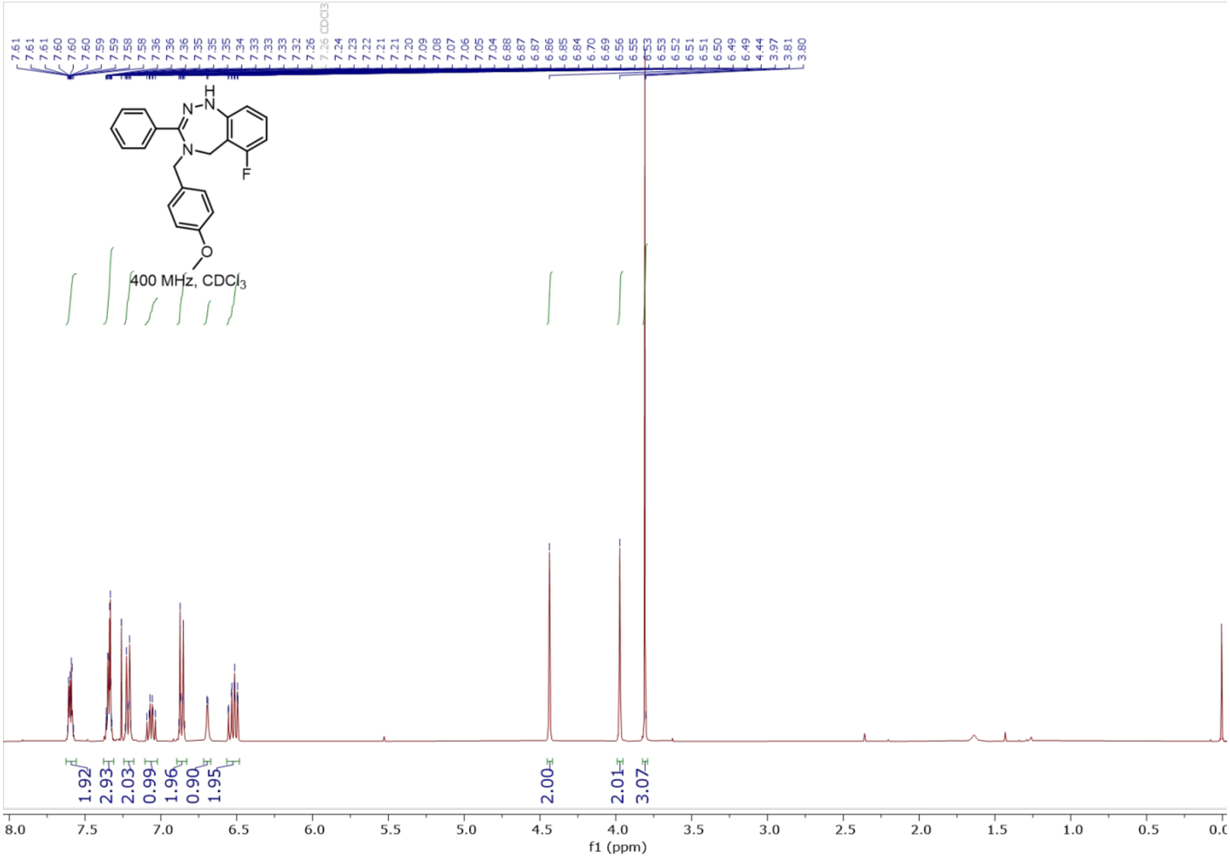

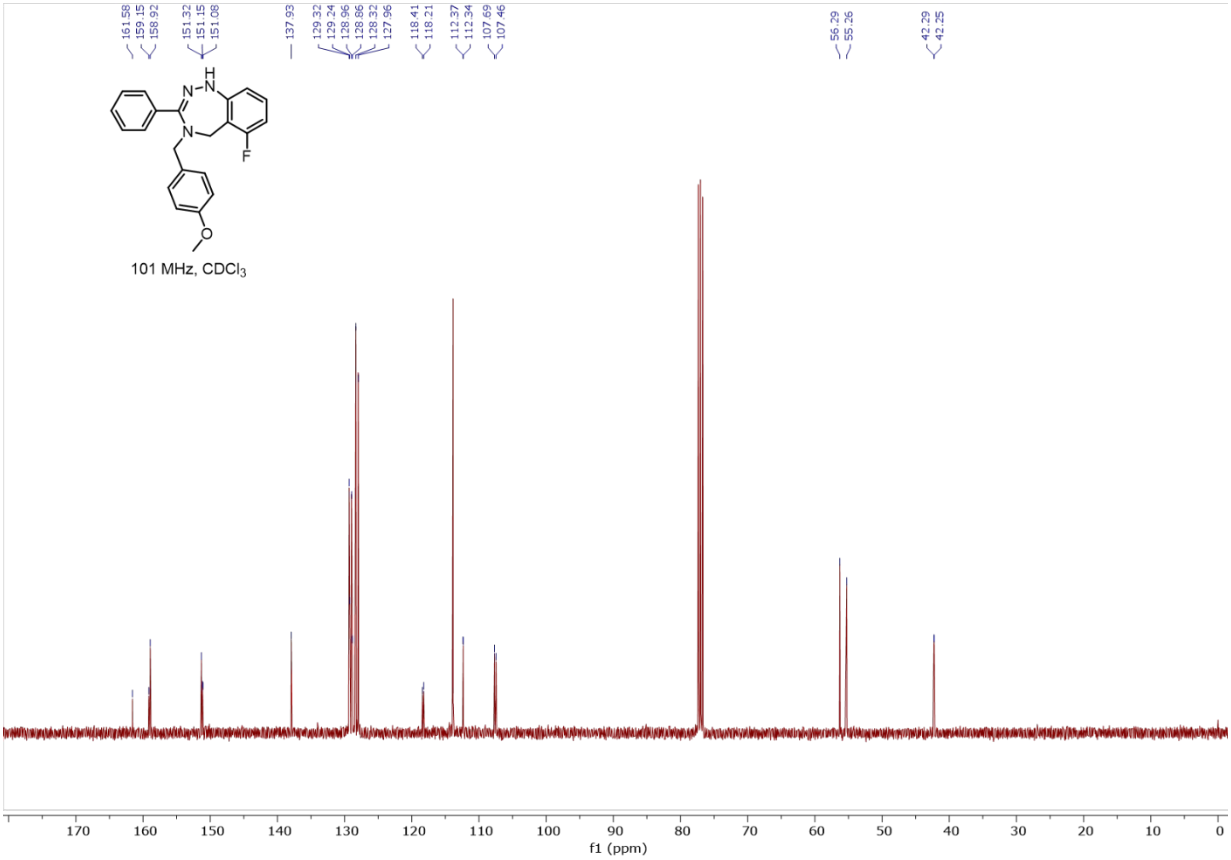

161.58
159.15
158.92
151.32
151.15
151.08
137.93
129.32
129.24
128.96
128.86
128.32
127.96
118.41
118.21
112.37
112.34
107.69
107.46
56.29
55.26
42.29
42.25
101 MHz, CDCl3
f1 (ppm)

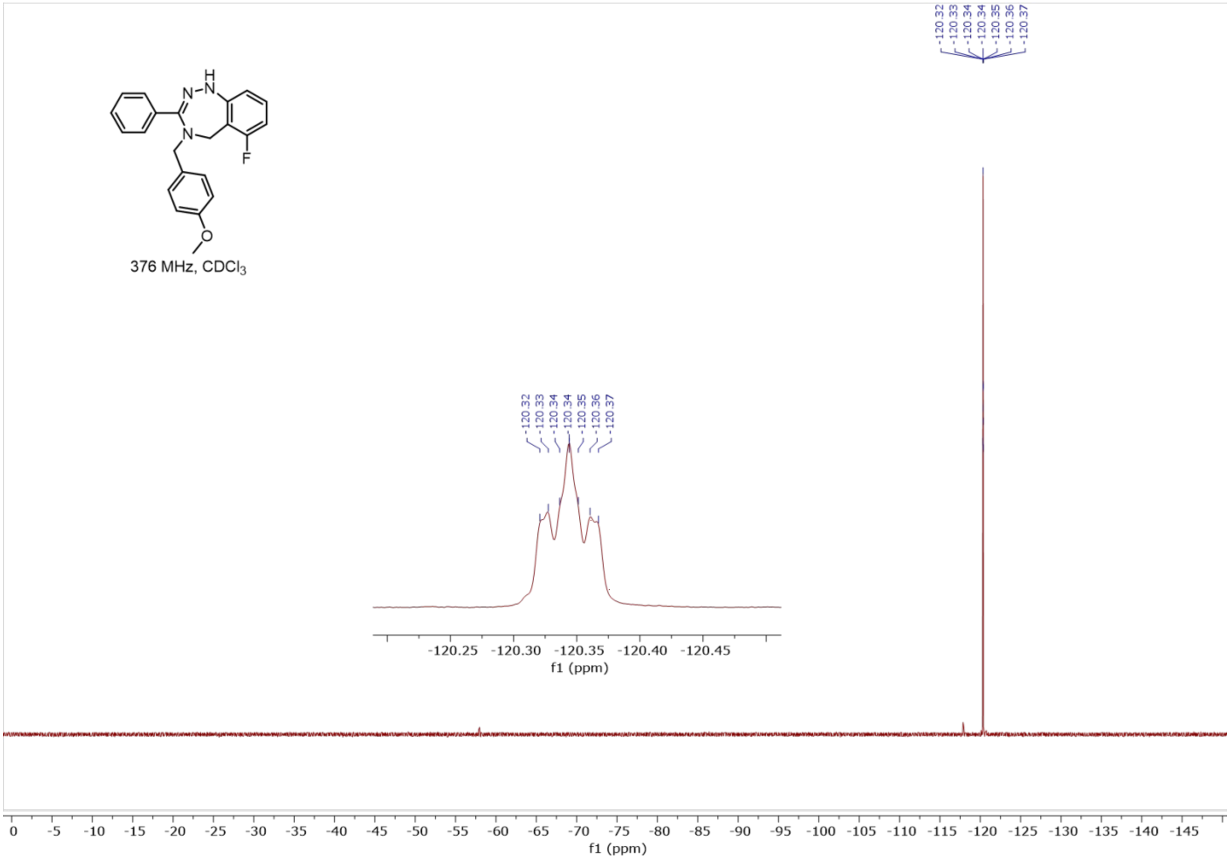

-120.32
-120.33
-120.34
-120.34
-120.35
-120.36
-120.37
376 MHz, CDCl3
f1 (ppm)

References.

(1) Baumann, M.; Burke, A.; Spiccio, S.; Di Filippo, M. Photochemical Synthesis of Pyrazolines from Tetrazoles in Flow. *SynOpen* **2022**, *07* (01), 69-75.
(2) Ramanathan, M.; Wang, Y. H.; Liu, S. T. One-Pot Reactions for Synthesis of 2,5-Substituted Tetrazoles from Aryldiazonium Salts and Amidines. *Org. Lett.* **2015**, *17* (23), 5886-5889.
(3) In Su Kim, G. R. D. a. Y. H. J. Palladium(II)-Catalyzed Isomerization of Olefins with Tributyltin Hydride. *J. Org. Chem.* **2007**, *72* (14), 5424 - 5426.
